# Chelating chloride using binuclear lanthanide complexes in water†‡

**DOI:** 10.1039/d2sc05417e

**Published:** 2022-12-27

**Authors:** Carlson Alexander, James A. Thom, Alan M. Kenwright, Kirsten E. Christensen, Thomas Just Sørensen, Stephen Faulkner

**Affiliations:** a Chemistry Research Laboratory, Department of Chemistry, University of Oxford 12 Mansfield Road Oxford OX1 3TA UK stephen.faulkner@chem.ox.ac.uk; b Department of Chemistry, University of Durham South Road Durham DH1 3LE UK; c Nano-Science Centre and Department of Chemistry, University of Copenhagen 2100 København Ø Denmark

## Abstract

Halide recognition by supramolecular receptors and coordination complexes in water is a long-standing challenge. In this work, we report chloride binding in water and in competing media by pre-organised binuclear kinetically inert lanthanide complexes, bridged by flexible –(CH_2_)_2_– and –(CH_2_)_3_– spacers, forming [Ln_2_(DO3A)_2_C-2] and [Ln_2_(DO3A)_2_C-3], respectively. These hydrophilic, neutral lanthanide coordination complexes are shown to bind chloride with apparent association constants of up to 10^5^ M^−1^ in water and in buffered systems. Hydroxide bridging was observed in these complexes at basic pH, which was proven to be overcome by chloride. Thus, these lanthanide complexes show promise towards chloride recognition in biology and beyond. The results described here have clearly identified a new area of anion coordination chemistry that is ripe for detailed exploration.

## Abstract 2

A series of binuclear lanthanide complexes address the long-standing challenge of binding chloride ions in aqueous solution. Binding of both chloride and fluoride is signalled by changes in the luminescence and NMR behaviour of the complexes.

## Graphical abstract

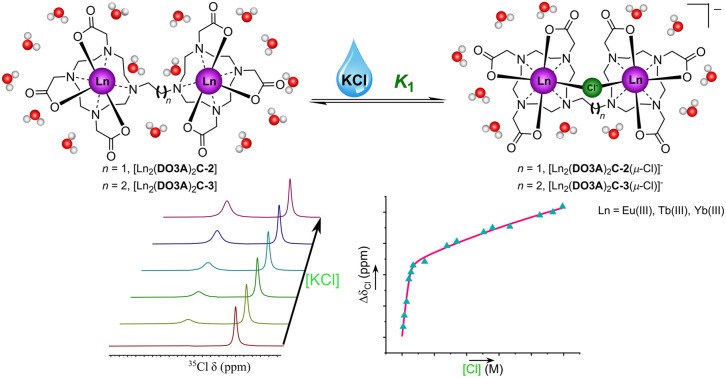

## 1 Introduction

Anions, including DNA, halides, carbonates, phosphates, sulfates, and nitrates are everywhere in the natural world, and play a key role in defining the biology and the environment.^1,2^ Despite this, the discussion of coordination chemistry was framed from the perspective of the metal ion for many years; it is only more recently that anions have been given the attention they deserve.^3,4^ Anion recognition relies on bonding of the guest anions to a host which exploits coulombic or supramolecular forces in general. In solution, solvent molecules can also bind to both guest and host, meaning that anion binding can be hard to achieve in water.^1,5^ While a host can almost invariably bind to a variety of guests, selectivity for a single type of anion is difficult to achieve.^1,3–5^ Selective anion recognition requires careful design to match host and guest, so some of these supramolecular systems can be challenging to synthesise and often involve intricate architectures of considerable structural complexity, yet minimal practical applicability.^4*a*^

Nature provides inspiration towards anion binding: protein-based interactions include bacterial sulfate binding protein and phosphatase enzymes, which selectively recognise sulfate and phosphate under physiological conditions *via* a network of hydrogen bonds from neutral donors. In these proteins, anion affinity is increased by a hydrophobic environment providing complementarity of charge and a shape that minimises the receptor desolvation energy.^6^ These interactions have inspired biomimetic receptors that integrate additional binding sites within the synthetic host, creating a structured binding pocket with high geometric complementarity.^1,3–5^ A range of interactions such as the macrocyclic effect,^1,2^ and conventional ion-pairing have been exploited in anion recognition;^5*a*^ the latter based on electrostatics,^1^ and supported by hydrogen bonding,^2^ halogen bonding,^7^ anion–π interactions,^8^ and coordinate bond using Lewis acids such as metals.^3,4^

Here, we focus on chloride binding. Chloride is the most abundant physiological anion^9^ and plays important roles in neuronal growth (104–115 mM in extracellular fluid),^10^ and development of the central nervous system (115–130 mM in cerebrospinal fluid) through the functions of chloride channels. The intracellular chloride concentration (up to 70 mM in eukaryotic cell types) is crucial in moderating neuronal excitability and neurotransmission.^11^ Tracking chloride in living systems is vital if we are to understand diseases related to chloride transport defects such as cystic fibrosis and epilepsy.^9,11,12^ Apart from its importance as the counterion of life,^9,12^ sequestering salt from sea water (559 mM of chloride in surface sea water)^13^ for clean drinking water is an ongoing challenge.^14^ However, developing suitably selective receptors for chloride in water continues to be a challenge.^1,4,5,15^

The large enthalpy and free energy of hydration of halides (Table 1) make chloride recognition challenging in water.^16*d*^ Halide anions exhibit different levels of hydration in different solvents as reflected by the Hofmeister series of anion hydrophobicity.^17^ Across the series,§§Hofmeister series of anion hydrophobicity (taken from ref. 17): CO_3_^2−^ > SO_4_^2−^ > S_2_O_3_^2−^ > H_2_PO_4_^−^ > HO^−^ > F^−^ > HCO_2_^−^ > CH_3_CO_2_^−^ > Cl^−^ > Br^−^ > NO_3_^−^ > I^−^ > CIO_4_^−^ > SCN^−^. the decrease in charge density results in increasing lipophilicity and weaker hydration.^7^ However, the solvation of the host must also be considered in designing an effective receptor for anion recognition.^1,3–5^

**Table 1:** Size, radius, hydration sphere, free energy of hydration and enthalpy of hydration for hydroxide and halides taken from ref. 16

Anion	Radius (Å)	*n*_hyd_	−Δ*G*^°^_hyd_ (kJ mol^−1^)	−Δ*H*^°^_hyd_ (kJ mol^−1^)
HO^−^	1.33	2.7	430	520
F^−^	1.33	2.7	465	510
Cl^−^	1.81	2	340	367
Br^−^	1.96	1.8	315	336
I^−^	2.2	1.6	275	291

The extent of solvation of the host, guest, and supramolecular assemblies depends on the solvent. Polar protic solvents such as water and methanol strongly solvate charged species, forming hydrogen bonds with both the anion and the host molecule.^1,5^ By contrast, in less polar aprotic solvents such as dichloromethane, the anion is weakly solvated and neutral receptors are able to function.^5,17^ Strong electrostatic or metal–ligand interactions offer the potential to overcome the anion hydration energy and allow binding in biological media.^3,4^

**Fig. 1:**
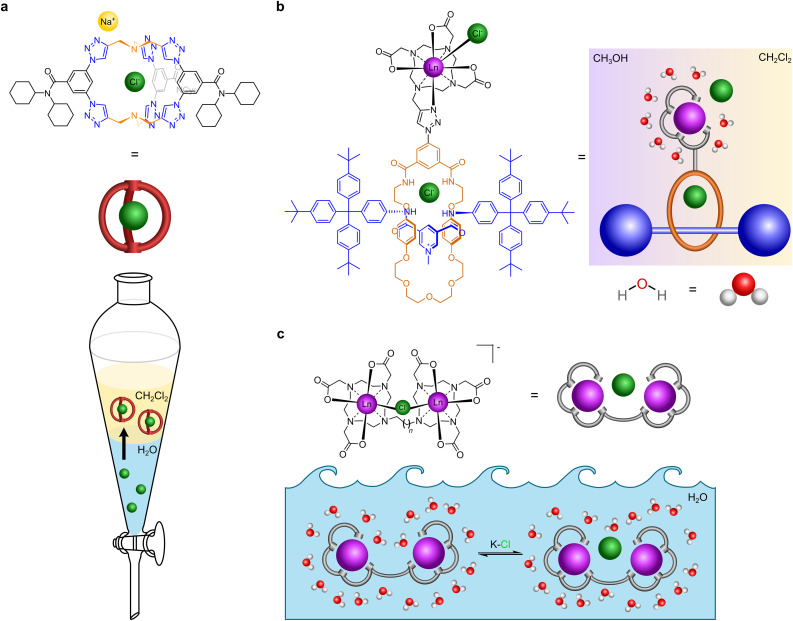
Cartoon illustration of receptors that recognise chloride. (a) Receptor that extracts chloride from water to dichloromethane (adapted from ref. 20). (b) Receptor that binds to chloride in mixed media (dichloromethane and methanol) (adapted from ref. 18). (c) Our receptor that binds to chloride in water.

We previously reported a lanthanide tethered rotaxane that binds to chloride in apolar media (Fig. 1b). In this, the first binding of chloride involves chelation to the metal, a second binding event occurs by encapsulation within the rotaxane.^18^ Gale, Davis *et al.* have reported cholapods made of a steroidal framework containing squaramide groups in axial positions which bind to chloride and other anions of tetraethylammonium salts with ∼10^14^ M^−1^ affinity in wet chloroform, employing a variation of Cram's extraction procedure.^19^ Flood *et al.* have reported a triazolo cage which binds to chloride with ∼10^17^ M^−1^ affinity in dichloromethane, which is very effective in sequestering chloride from water and has been shown to prevent brine-accelerated corrosion when coated onto mild steel (Fig. 1a).^20^ Beer *et al.* have designed rotaxane hosts based on iodotriazole halogen bonding and amide hydrogen bonding with the halogens, but it recognises iodide (*K*_a_ = 2200 M^−1^) better than chloride (*K*_a_ = 55 M^−1^) and bromide in water.^21^ Other chloride binding supramolecular agents have been extensively reviewed elsewhere.^1,5,15^

Lanthanide complexes are an attractive choice for developing anion receptors due to their ability to report anion binding by MRI, luminescence, and optical imaging.^22^ The luminescence applications use lanthanide complexes as luminescent tags, exploiting stable complexes in which their luminescence signal is enhanced *via* the antenna principle where chromophores sensitise lanthanide luminescence and overcome the inherently low molar absorption coefficients associated with f–f transitions.^22,23^ Furthermore, the necessity for kinetic stability in clinical use ensures that macrocyclic ligands related to DOTA are well understood in competitive media with high anion concentrations.^4,22,23^ In such systems, anion chelation is achievable on the metal site without the dissociation of the lanthanide complex.^4^

Anion recognition by lanthanide complexes can reflect either collisional quenching of excited states by anions,^24^ or anion binding.^4^ While luminescence can be modulated both by collision and anion binding, the latter generates a ‘turn-off’ event in MRI since the functioning of MRI contrast agents requires the presence of water molecules in the inner-coordination sphere which exchange with the bulk water.^24^ Thus, isostructural MRI and luminescent probes can be constructed with suitable lanthanide ions for targeted anion binding thereby allowing one design platform to exploit bio-imaging combining paramagnetism and luminescence, an exclusive feature offered by the lanthanides.^4,22^ Halides are strong Lewis bases which coordinate to metals that are hard Lewis acids.^25^ Using this HSAB approach, several mononuclear Ln(iii) complexes comprising cryptands,^26^ and cyclen derived *C*_2_ (ref. 27) and *C*_4_ symmetric^28,29^ ligands have been reported to chelate fluoride at the metal centre, but none have been reported to bind chloride in water.

Binuclear lanthanide(iii) complexes of two heptadentate DO3A-based binding domains tethered by aryl spacers were studied for binding organic dicarboxylate anions at physiological pH;^30^ sensing biologically relevant anions such as phosphate, methylphosphate, double-stranded DNA and a DNA hairpin loop;^31^ and as a pH sensor.^32^ We have explored this binuclear approach by incorporating kinetically inert charge neutral lanthanide DO3A centres bridged by different *m*-xylyl scaffolds to achieve reversible binding of dinicotinate and isophthalate guests with high affinity at physiological conditions, but this requires methanol to improve the solvation of these guests.^33^

In this work, we report how binuclear complexes can be tailored to bind chloride ions in water under conditions where mononuclear complexes^4,26–30^ and free lanthanide ions^34^ do not interact with chloride. We hypothesised that coordinatively unsaturated neutral binuclear lanthanide(iii) complexes of DO3A ligands bridged by ethane and propane linkers ([Ln_2_(DO3A)_2_C-2] and [Ln_2_(DO3A)_2_C-3]) would create a pre-organised pocket between the metal centres capable of hosting halides coordinated to the lanthanide metal centres. Furthermore, having a short and flexible spacer between the coordinatively unsaturated hard Lewis acids, which act as acceptors would increase the steric strain thereby limiting their conformational freedom,^35^ where subsequent halide binding would lead to a smaller entropy loss in the host. Both binuclear complexes respond to halides under physiological conditions and the binding can be followed by luminescence and NMR spectroscopy. A neutral mononuclear lanthanide propargyl DO3A complex [Ln(pDO3A)] was^23^ used to compare the relative effectiveness of mononuclear complexes.

## 2 Results and discussion

### 2.1 Synthesis and characterisation

**Scheme 1:**
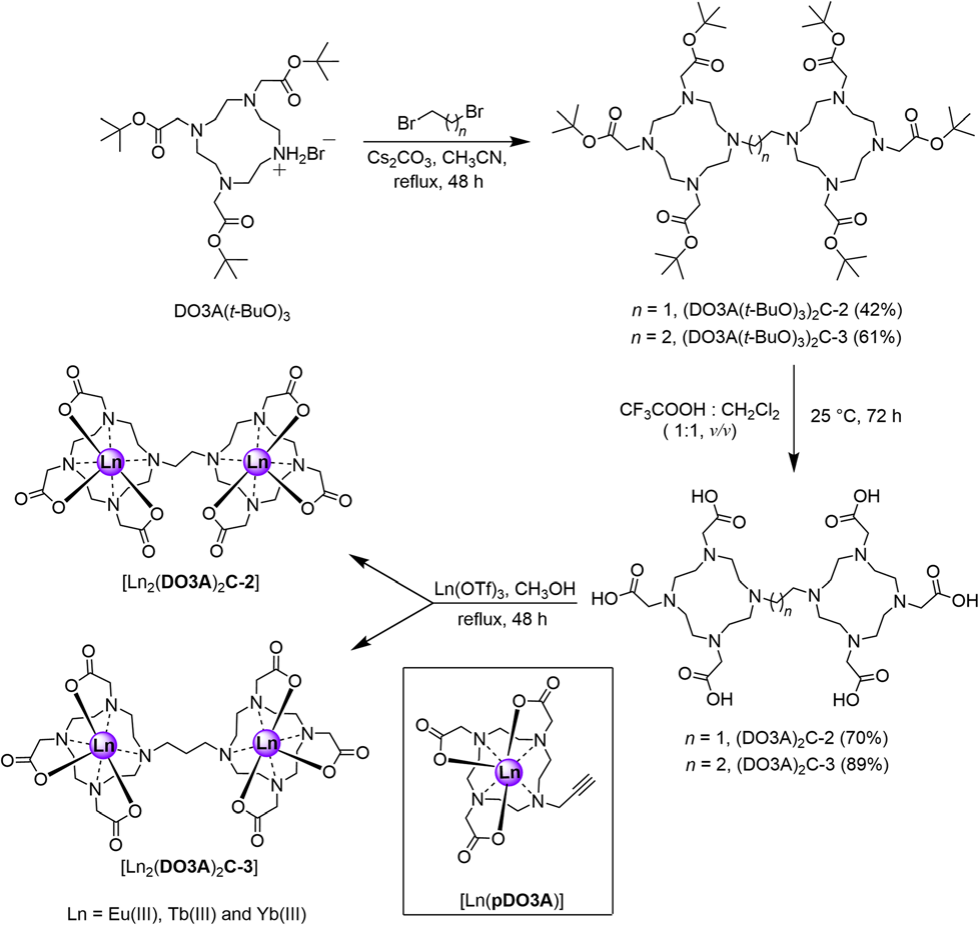
Synthetic scheme of proposed binuclear complexes. The mononuclear complex used as a model system is shown inside the black box.

Synthesis of the proposed complexes is shown in Scheme 1. Detailed synthetic protocol, purification, and characterisation is reported in the ESI.‡ Cyclen was tri-*N*-alkylated using *t*-butyl bromoacetate to form the triester, DO3A(*t*-BuO)_3_. Subsequent alkylation with 1,2-dibromoethane and 1,3-dibromopropane yielded the ethane and propane bridged bis-macrocycles, (DO3A(*t*-BuO)_3_)_2_C-2 or (DO3A(*t*-BuO)_3_)_2_C-3. Following purification and deprotection with trifluoroacetic acid, the pro-ligands (DO3A)_2_C-2 and (DO3A)_2_C-3 were obtained. These were reacted with the appropriate lanthanide triflate salt in methanolic solution under reflux and worked-up to afford the complexes [Ln_2_(DO3A)_2_C-2] and [Ln_2_(DO3A)_2_C-3]. Single crystal X-ray structures of (DO3A(*t*-BuO)_3_)_2_C-2 (Fig. S122; Table S9‡), pDO3A(*t*-BuO)_3_ (Fig. S123; Table S10‡), [Ln_2_(DO3A)_2_C-3] and [Ln(pDO3A)] (Ln = Eu(iii) and Yb(iii)) were obtained. The binuclear complexes crystallised as a cluster containing 12 metal centres *via* μ-oxo bridges from the carbonyl oxygens, maintaining the connectivity and neutrality of the complexes (Fig. S124; Table S11‡). A crystal structure of [Lu(pDO3A)] has been reported as a dimer, linked by a carbonate.^18^ In our case, [Ln(pDO3A)] (Ln = Eu(iii) and Yb(iii)) crystallised without any anion bound to the metal centre (Fig. S126; Table S13‡).

These kinetically robust binuclear complexes^23^ exhibit variations in behaviour with pH. At neutral pH, the ^1^H NMR spectra of the paramagnetic Eu(iii) and Yb(iii) complexes consist of broad lines; however, at pD 10.12, the lines are sharp and well defined (Fig. S28, S31, S34, and S36‡). This observation can be explained by the rapid inter-conversion between diastereomeric forms of the compound, in which the coordination environment at the lanthanide centre varies between Square Antiprism (SAP) and Twisted Square Antiprism (TSAP) geometries. At high pH, interchange between these species was found to be slow on the NMR timescale, resulting in sharp spectral lines. Furthermore, luminescence spectra of [Eu_2_(DO3A)_2_C-2] and [Eu_2_(DO3A)_2_C-3] at pH 4 and 7 showed no significant change (Fig. S62–S65‡), but at pH 9.9, the form and shape of the emission spectra was changed, which implies hydroxide binding to the metal centres. A decrease in the hydration number of the complexes can be inferred from luminescence lifetime measurements at increasing pH (Fig. S112 and S115; Tables S1 and S4‡). These results complement the NMR observations that chelation to hydroxide is observed at basic pH.

### 2.2 Effect of chloride on the mono- and binuclear Ln(iii) complexes

No fluoride chelating Ln(iii) complexes have been observed to bind chloride at the metal centre.^26–29^ Since the binuclear complexes in this work are neutral species, a neutral monometallic Ln(iii) complex [Ln(pDO3A)] was used as a model system to study halide binding. Steady-state luminescence titration of [Eu(pDO3A)] with potassium chloride (KCl) salt in deionised water did not result in any binding event (Fig. S108 and S109‡).

**Fig. 2:**
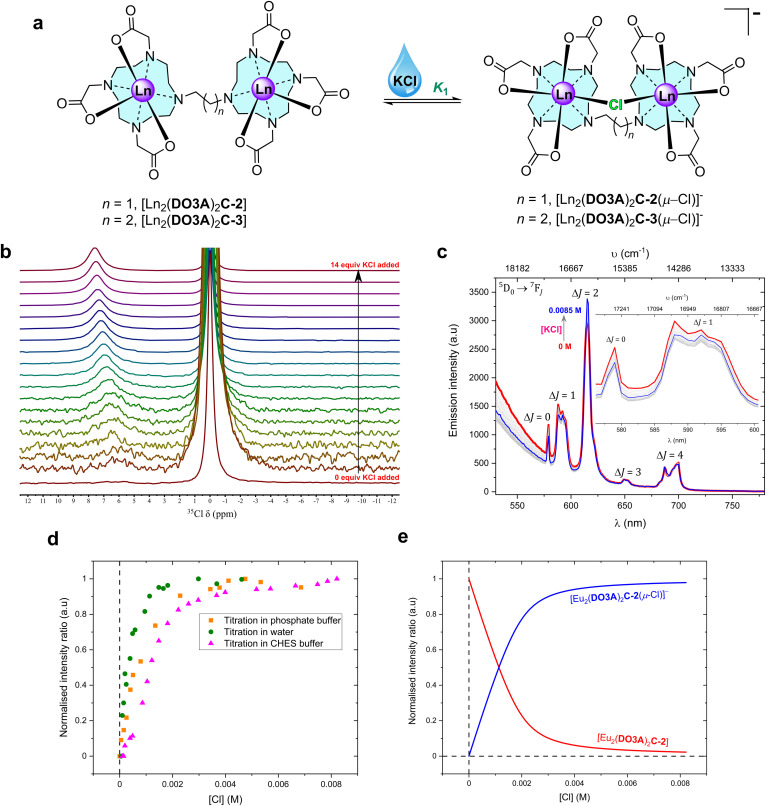
Chloride chelation by the binuclear complexes in water. (a) Structural representation of the binding events in water. (b) 49 MHz ^35^Cl NMR titration spectra of 0.035 M [Tb_2_(DO3A)_2_C-3] with increasing additions of KCl (stock concentration, 1.4 M) in D_2_O at 298 K (Fig. S52 for binding isotherm‡). Each spectrum was recorded with a capillary tube insert containing saturated KCl in D_2_O (*δ* = 0 ppm) (non-dilution method used). (c) Steady-state luminescence titration spectra of 1 mM [Eu_2_(DO3A)_2_C-2] (*λ*_ex_ = 393 nm) against KCl (stock concentration, 0.02 M) in 0.01 M CHES buffer (pH 9.98) at 22 °C; spectrum of neat complex solution (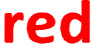), spectra upon the additions of KCl (grey), spectrum upon the final addition of KCl (
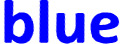
) (non-dilution method used). The inset expands the Δ*J* = *0* and Δ*J* = 1 emission bands (Fig. S78 for binding isotherm‡). (d) Normalised emission trend of Δ*J* = 2/Δ*J* = 1 obtained from steady-state emission titrations for 1 mM [Eu_2_(DO3A)_2_C-2] against KCl in deionised water, 0.01 M phosphate buffer (pH 7.4), and 0.01 M CHES buffer (pH 9.9) at 22 °C. (e) Normalised model of speciation obtained from Δ*J* = 2/Δ*J* = 1 bands from steady-state emission titration spectra of [Eu_2_(DO3A)_2_C-2] against KCl in 0.01 M CHES buffer (pH 9.9) at 22 °C.

Chloride binding by the binuclear Eu(iii) complexes were then explored. Steady-state luminescence titrations were performed in deionised water, phosphate buffer at pH 7.4, and in CHES buffer at pH 9.9. In the case of deionised water and in phosphate buffer, the emission intensity decreased with increasing KCl addition, but the opposite was the case in CHES buffer (Fig. 2, S74–S78 and S94–S99‡). It should be noted that chloride can act as a PeT quencher, reducing the observed lanthanide centred emission intensity.^24^ However, a single binding event (*K*_1_) was observed in all three cases and the binding strength increased as we move into buffered systems (Table 2). These results could be suggestive of competitive binding of phosphate: indeed, the changes to the form of the spectra in phosphate buffer (in comparison with the spectra in water) are strongly suggestive of phosphate binding in line with other mono and binuclear lanthanide complexes derived from DO3A.^4,31,37^

Apparent binding constants for binuclear and mononuclear Eu(iii) complexes in aqueous media^a^

**Table:** 

Halide	Media^b^	Binding constant (*K* in M^−1^)^c^^,^^d^	
		[Eu_2_(DO3A)_2_C-2]	[Eu_2_(DO3A)_2_C-2]
Cl^−^	Water	*K*_1_ = 2800 (±7%)	*K*_1_ = 4550 (±3.9%)
	PB	*K*_1_ = 9400 (±12.2%)	*K*_1_ = 6640 (±7.3%)
	CHES	*K*_1_ = 7460 (±11.2%)	*K*_1_ = 12 210 (±7%)

F^−^	Water	*K*_1_ = 600 000 (±39.7%)	*K*_1_ = 10 480 (±5.9%)
		*K*_2_ = 144 000 (±33.5%)	*K*_2_ = 10 950 (±4%)
		*K*_3_ = 394.7 (±2.3%)	*K*_3_ = 6570 (±3.6%)

	Methanol	*K*_1_ = 54 800 (±11.8%)	*K*_1_ = 179 000 (±13.2%)
		*K*_2_ = 20 200 (±10.6%)	*K*_2_ = 32 000 (±13%)
		*K*_3_ = 0.002843 (±0.6%)	*K*_3_ = 728 (±7.5%)

	PBS	*K*_1_ = 4030 (±2.9%)	*K*_1_ = 5350 (±6.9%)
			*K*_2_ = 18.57 (±1%)

	Tris–HCl	*K*_1_ = 106 000 (±38.9%)	*K*_1_ = 1670 (±14.7%)
		*K*_2_ = 219.1 (±0.8%)	*K*_2_ = 116.4 (±1.7%)

	CHES	*K*_1_ = 13 600 (±9.9%)	*K*_1_ = 3840 (±9.6%)
			*K*_2_ = 3494 (±2.8%)

a The binding of halides with the complexes were studied by steady-state luminescence titrations with varying concentration of potassium halides (non-dilution method used) and binding isotherm generated using DYNAFIT®.

b All buffers were maintained at 0.01 M in deionised water: PBS = phosphate buffer saline at pH 7.4; PB = phosphate buffer at pH 7.4; CHES = *N*-cyclohexyl-2-aminoethanesulfonic acid at pH 9.98; Tris–HCl = tris(hydroxymethyl)aminomethane hydrochloride at pH 7.4.

c *K*_1_ = first binding event; *K*_2_ = second binding event; *K*_3_ = third binding event – deduced from binding isotherm generated using DYNAFIT®.

d ± is the coefficient of variation in percentage for each binding event obtained from binding isotherm plotted in DYNAFIT® by employing trust-region algorithm in confidence interval at 95% probability level. Confidence intervals for all binding constants are given in the ESI under each binding isotherm.

**Table:** 

Halide	Media	Binding constant (K in M − 1)^c^^,^^d^ for [Eu(pDO3A)]
F^−^	Water	*K*_1_ = 693 (±3.2%)
	Methanol	*K*_1_ = 5720 (±6.4%); *K*_2_ = 0.00068 (±1.2%)

NMR studies of chloride binding at lanthanide centres present difficulties due to the interference of the quadrupolar relaxation from the low symmetry ^35^Cl nucleus with the paramagnetic metal centre resulting in broad ^1^H and ^35^Cl NMR signals. However, dipolar ^35^Cl NMR chemical shifts induced by the addition of chloride to axially symmetric [Ln(DOTA)]^−^ complexes give well-resolved ^35^Cl NMR spectra that can be interpreted as evidence of interaction with chloride.^36^ By contrast, when [Tb(pDO3A)] was added to a solution of KCl in deuterium oxide, no new peaks were observed in ^35^Cl NMR, and the chemical shift of the chloride resonance was unchanged, suggesting that chloride does not bind in our model system (Fig. S45‡).

The ^35^Cl NMR spectra of [Tb_2_(DO3A)_2_C-3] in deuterium oxide display chemical shift upon the addition of KCl (Fig. 2b). This shift can be ascribed to fast exchange between bound and free chloride; this is borne out by the fact that increasing addition of KCl increases the observed paramagnetic shift. Therefore, ^35^Cl NMR titrations were pursued with [Tb_2_(DO3A)_2_C-3] against KCl in D_2_O. The resulting chemical shifts were fitted to generate a binding isotherm. A strong first binding event followed by a very weak second binding event was observed (Fig. S47, S49, S51 and S52‡). Titrations were performed at 4 different temperatures in D_2_O in order to understand the thermodynamics of chloride binding to [Tb_2_(DO3A)_2_C-3] (Fig. S46–S52‡). The resulting van't Hoff plot suggested this chelation to be exothermic with high negative enthalpy (Fig. S53‡). By comparison with the results reported for [Tb(DOTA)]^−^ where chloride binding is axial and a negative shift results (Fig. S45‡),^36^ this suggests that chloride ions bind at the equatorial position on the metal centres in [Tb_2_(DO3A)_2_C-3] (Fig. 2). Similarly, ^35^Cl NMR titrations were performed with [Tb_2_(DO3A)_2_C-2] against KCl in D_2_O at 4 different temperatures. Although chlorine bound chemical shifts were observed, the resulting binding isotherms were sigmoidal in shape which was difficult in deducing meaningful binding events. However, they suggest the likely competition between chloride and hydroxide in occupying the binding site between the metal centres in [Tb_2_(DO3A)_2_C-2].

After establishing chloride binding by the binuclear Eu(iii) and Tb(iii) complexes in water, the association was further evaluated by high resolution ESI-Mass spectrometry. The mass spectral peak of a chloride bound to the Eu(iii) binuclear complexes in deionised water were found at *m*/*z* 1051.1486 and *m*/*z* 1065.1640 for C-2 and C-3 complexes, respectively, and confirmed by the calculated isotopic distribution pattern (Fig. S54 and S55‡), thus further supporting the formation of the ternary complexes [Ln_2_(DO3A)_2_C-2(μ-Cl)]^−^ and [Ln_2_(DO3A)_2_C-3(μ-Cl)]^−^.

### 2.3 Exploring chloride binding mode using fluoride

To investigate the mechanism of halide binding in binuclear lanthanide complexes, fluoride binding was explored by titrating the complexes with solutions of potassium fluoride (KF) salt. Fluoride is known to bind strongly to a range of complexes,^26–29^ though it can be neglected as a potential interferent in biological applications as the concentration of free fluoride is very low (70 μM in surface sea water,^13^ and 20–210 μM for human consumption as recommended by WHO^38^). However, we can use the fluoride interaction to further investigate the mode of chloride binding.

**Fig. 3:**
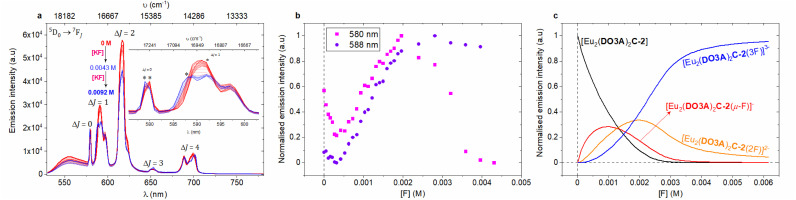
(a) Steady-state emission titration spectra of 1 mM [Eu_2_(DO3A)_2_C-2] against KF (stock concentration, 0.02 M) in deionised water at 22 °C. Spectrum of neat complex solution (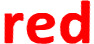 in bold), spectra upon the additions of KF (
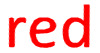
 to 
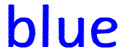
), spectrum upon the final addition of KF (
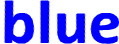
 in bold) (non-dilution method used). The inset expands the Δ*J* = 0 and Δ*J* = 1 emission bands and the asterisks highlight the emission maxima used in quantifying the binding of fluoride to the complex (Fig. S79 for binding isotherm‡). (b) Normalised trend of selected emission wavelengths in Δ*J* = 0 and Δ*J* = 1 transitions obtained from the titration of [Eu_2_(DO3A)_2_C-2] against KF in deionised water at 22 °C used in studying and quantifying binding. (c) Normalised model of speciation obtained from 580 nm emission in the steady-state emission titration spectra of [Eu_2_(DO3A)_2_C-2] against KF in deionised water at 22 °C.

Steady-state emission titrations revealed that three fluoride ions are successively bound to the binuclear lanthanide complexes as the fluoride concentration was increased in deionised water. These three binding events were also observed in methanol, where the experimental observables at each event were better resolved, presumably due to better solvation of KF, and the lack of competing hydroxides.^39^ The binding isotherms were fitted to three binding events; all three were needed to generate an acceptable fit (Fig. 3).

**Fig. 4:**
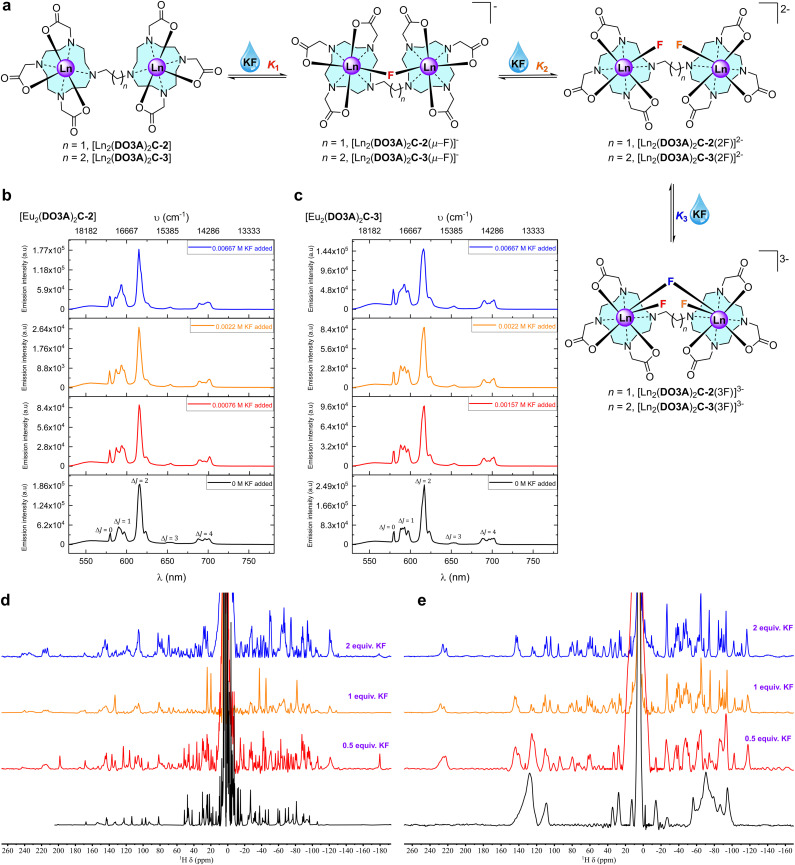
Fluoride chelation by the binuclear complexes in aqueous media. (a) Structural representation of binding events in aqueous media. (b) Stacked steady-state luminescence of 1 mM [Eu_2_(DO3A)_2_C-2] with KF in methanol at 22 °C. The spectra are arranged as per the binding events deduced from the luminescence titration spectra (Fig. S72‡) and binding isotherm (Fig. S73‡). (c) Stacked steady-state luminescence of 1 mM [Eu_2_(DO3A)_2_C-3] with KF in methanol at 22 °C. The spectra are arranged as per the binding events deduced from the luminescence titration spectra (Fig. S92‡) and binding isotherm (Fig. S93‡). (d) 500 MHz stacked paramagnetic ^1^H NMR spectra of [Yb_2_(DO3A)_2_C-2] with increasing KF in deuterium oxide at 298 K. (e) 500 MHz stacked paramagnetic ^1^H NMR spectra of [Yb_2_(DO3A)_2_C-3] with increasing KF in deuterium oxide at 298 K.

From the luminescence titrations (Fig. 3, S72, S73 and S79‡ for [Eu_2_(DO3A)_2_C-2]; Fig. S84, S85, S92, and S93‡ for [Eu_2_(DO3A)_2_C-3]), it can be hypothesised that the speciation involves binding of first fluoride (*K*_1_) by chelation between the metal centres. Excess addition of KF generates the second binding event (*K*_2_) which involves chelation of fluoride to each metal, while the third binding event (*K*_3_) forms a bridging fluoride in addition to the fluoride chelated to each metal (Fig. 4).

**Fig. 5:**
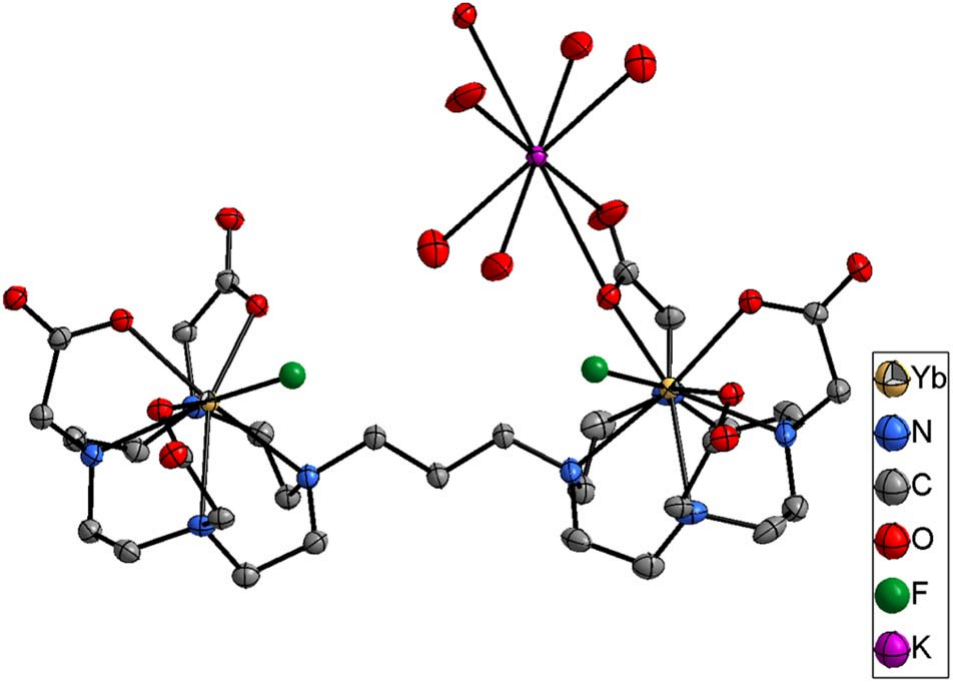
Single crystal X-ray of [Yb_2_(DO3A)_2_C-3(2F)]^2−^. H atoms and water omitted for clarity. Thermal ellipsoid drawn at 30% probability level.

When titrations with the binuclear Eu(iii) complexes and KF were performed in competing (phosphate buffered saline at pH 7.4), non-competing (Tris–HCl buffer at pH 7.4), and basic (CHES buffer at pH 9.98) media, the binding strength as well as the number of fluoride bound to the binuclear Eu(iii) complexes were lowered and no more than two binding events were observed (Fig. S66–S71‡ for [Eu_2_(DO3A)_2_C-2] and Fig. S84–S91‡ for [Eu_2_(DO3A)_2_C-3]). A single crystal X-ray structure of [Yb_2_(DO3A)_2_C-3(2F)]^2−^ was obtained (Fig. 5) which verifies our hypothesis on *K*_2_ mode of fluoride binding to the binuclear complexes. In luminescence titrations, the emission spectral change caused by fluoride is so high due to its strong influence on the crystal-filed of the metal centre as a result of its small-size with highly dense charge and strong electrostatic interaction in comparison with chloride.^16*d*,29*a–c*^ It is noteworthy from the above observations that although fluoride chelation by the binuclear systems suffers from competing ions in buffers, chloride binding is enhanced amidst competition from other ions (Table 2).

As expected, in luminescence lifetime measurements on the binuclear Eu(iii) complexes, upon increasing the concentration of fluoride in deionised water, methanol and their deuterated counterparts, a decrease in the hydration number accounting for the displacement of inner-sphere water by the coordination of fluoride ions was observed (Fig. S110, S111, S113 and S114; Tables S2, S3, S5 and S6‡). Surprisingly, no change in the luminescence lifetime was observed during the addition of chloride with Eu(iii) complexes which we hypothesise due to a counteracting interference between Photoinduced electron transfer (PeT) from chloride and intermetallic luminescence quenching.

The ^1^H NMR spectra of Eu(iii) and Yb(iii) binuclear complexes sharpen with increasing addition of fluoride due to its chelation involving both the metal centres which slows the rate of interconversion between the SAP and TSAP isomers (Fig. 4d, e, S27, S29, S30, S32, S33, S35 and S37‡). New signals are observed that extend the NMR spectral range in both the positive and negative chemical shift regions suggesting equatorial binding of fluoride with respect to the metal centre: axial binding of fluoride commonly results in a decrease or inversion of the local crystal field by destabilising axially oriented m_*j*_ states, while equatorial binding destabilises other m_*j*_ states.^27–29^^19^F NMR spectra of binuclear Eu(iii) complexes in deuterium oxide contain a small peak at around −450 ppm for the fluoride bound to the complexes (Fig. S42 and S43‡) which is similar to the chemical shift reported for fluoride binding in mononuclear complexes.^27*d*,28,29^

### 2.4 Control experiments with mononuclear complexes

In [Eu(pDO3A)], no chloride chelation was observed (Fig. S45, S108, and S109‡). However, when this complex was titrated against KF in deionised water and methanol, a single binding event was observed (Table 2, Fig. S104–107‡). Results from time-resolved lifetime, paramagnetic ^1^H NMR, and ^19^F NMR are similar to the interaction of fluoride to the binuclear systems (Fig. S38–S40, S43, S116 and S117; Tables S7 and S8‡). Although fluoride chelation was observed in these binuclear systems, it does not need the binuclear cavity which chloride demands.^26–29^

### 2.5 Chloride selectivity over other halides on binuclear Ln(iii) complexes

In contrast to fluoride, significant interference for a chloride receptor can be envisioned from the heavier halides. Titration of the binuclear Eu(iii) complexes against bromide and iodide solutions in deuterium oxide and deionised water gave broadened NMR spectra and no observable changes to the fine structure in the luminescence spectra (Fig. S80–S83‡ for [Eu_2_(DO3A)_2_C-2]; Fig. S100–S103‡ for [Eu_2_(DO3A)_2_C-3]) indicating that these ions bind very weakly, if at all. Thus, we have a highly selective receptor for chloride.

## 3 Conclusion

Binuclear lanthanide complexes can bind to chloride ions in aqueous solution provided that two lanthanide ions can bind to chloride – reducing intermetallic repulsions. This offers new scope for the development of chloride specific/selective molecular probes that can be exploited in competitive biological media. Much remains to be done, but the results described here clearly identify a new area of anion coordination chemistry that is ripe for detailed exploration. With the current need for chloride binding receptors in water for aquifers and *in vivo*, these hydrophilic binuclear complexes are a good beginning. This work is a possible solution to the decades old challenge of chloride recognition in water, which opens up new avenues to various applications from biology to sensors and beyond.

## Data availability

ESI is available which contains detailed methods and materials, detailed synthesis and characterization, titrations (NMR and luminescence), speciation models, luminescence lifetimes and single crystal X-ray data. Crystallographic information for the structures reported in this article have been deposited at the Cambridge Crystallographic Data Centre, under deposition numbers CCDC 2201928 for (DO3A(*t*-BuO)_3_)_2_C-2, CCDC 2201929 for pDO3A(*t*-BuO)_3_, CCDC 2201930 for [Eu_2_(DO3A)_2_C-3], CCDC 2201931 for [Yb_2_(DO3A)_2_C-3], CCDC 2201932 for [Yb_2_(DO3A)_2_C-3(2F)]^2−^, CCDC 2201933 for [Eu(pDO3A)], and CCDC 2201934 for [Yb(pDO3A)].

## Author contributions

Conceptualisation, S. F.; supervision, S. F., A. M. K. and T. J. S.; methodology, C. A., S. F., A. M. K., T. J. S. and K. E. C.; investigation, C. A., K. E. C. and J. A. T.; validation, C. A.; formal analysis, C. A., T. J. S., K. E. C. and S. F.; data curation, C. A. and K. E. C.; visualisation, C. A.; writing – original draft, C. A., T. J. S. and S. F.; writing – review & editing, C. A., T. J. S., A. M. K., S. F., K. E. C. and J. A. T.; resources, S. F. and K. E. C.; funding acquisition, S. F. and C. A.

## Conflicts of interest

There are no conflicts to declare.

## Supplementary Material

SC-014-D2SC05417E-s001

SC-014-D2SC05417E-s002

## References

[cit1] (b) Steed J. W. and Atwood J. L., Supramolecular chemistry, John Wiley and sons, New York, 3rd edn, 2022

[cit2] Dalkara D., Zuber G., Behr J. P. (2004). Intracytoplasmic delivery of anionic proteins. Mol. Ther..

[cit3] Steed J. W. (2009). Coordination and organometallic compounds as anion receptors and sensors. Chem. Soc. Rev..

[cit4] Butler S. J., Parker D. (2013). Anion binding in water at lanthanide centres: from structure and selectivity to signalling and sensing. Chem. Soc. Rev..

[cit5] Park C. H., Simmons H. E. (1968). Macrobicyclic amines. III. Encapsulation of halide ions by in,in-1, (*k* + 2)-diazabicyclo[*k.l.m*]alkaneammonium ions. J. Am. Chem. Soc..

[cit6] Pflugrath J. W., Quiocho F. A. (1985). Sulphate sequestered in the sulphate-binding protein of Salmonella typhimurium is bound solely by hydrogen bonds. Nature.

[cit7] Gilday L. C., Robinson S. W., Barendt T. A., Langton M. J., Mullaney B. R., Beer P. D. (2015). Halogen bonding in supramolecular chemistry. Chem. Rev..

[cit8] Chifotides H. T., Dunbar K. R. (2013). Anion–π interactions in supramolecular
architectures. Acc. Chem. Res..

[cit9] Dodani S. C. , in Building better chloride sensors, ed. E. G. Berg, Chem. Eng. News, 2018, (Dec 28)

[cit10] Cole D. E. C., Shafai J., Scriver C. R. (1982). Inorganic sulfate in cerebrospinal fluid from infants and children. Clin. Chim. Acta.

[cit11] Reuter D., Zierold K., Schröder W. H., Frings S. (1998). A depolarizing chloride current contributes to chemoelectrical transduction in olfactory sensory neurons in situ. J. Neurosci..

[cit12] Tutol J. N., Peng W., Dodani S. C. (2019). Discovery and characterization of a naturally occurring, turn-on yellow fluorescent protein sensor for chloride. Biochemistry.

[cit13] Pilson M. E. Q. , An Introduction to the Chemistry of the Sea, Cambridge University Press, New York, 2^nd^ edn, 2012

[cit14] Guo Y., Compton R. G. (2021). A bespoke chloride sensor for seawater: Simple and fast with a silver electrode. Talanta.

[cit15] Wu X., Gilchrist A. M., Gale P. A. (2020). Prospects and challenges in anion recognition and transport. Chem.

[cit16] Shannon R. D. (1976). Revised effective ionic radii and systematic studies of interatomic distances in halides and chalcogenides. Acta Crystallogr., Sect. A: Found. Adv..

[cit17] Hofmeister F. (1888). Zur Lehre von der Wirkung der Salze (About the Science of the Effect of Salts). Arch. Exp. Pathol. Pharmakol..

[cit18] Allain C., Beer P. D., Faulkner S., Jones M. W., Kenwright A. M., Kilah N. L., Knighton R. C., Sørensen T. J., Tropiano M. (2013). Lanthanide appended rotaxanes respond to changing chloride concentration. Chem. Sci..

[cit19] Edwards S. J., Valkenier H., Busschaert N., Gale P. A., Davis A. P. (2015). High-affinity anion binding by steroidal squaramide receptors. Angew. Chem., Int. Ed..

[cit20] Liu Y., Zhao W., Chen C.-H., Flood A. H. (2019). Chloride capture using a C–H hydrogen-bonding cage. Science.

[cit21] Langton M. J., Robinson S. W., Marques I., Félix V., Beer P. D. (2014). Halogen bonding in water results in enhanced anion recognition in acyclic and rotaxane hosts. Nat. Chem..

[cit22] (b) de Bettencourt-Dias A. . Luminescence of Lanthanide Ions in Coordination Compounds and Nanomaterials, John Wiley and Sons, New York, 2014

[cit23] Sørensen T. J., Faulkner S. (2018). Multimetallic lanthanide complexes: Using kinetic control to define complex multimetallic arrays. Acc. Chem. Res..

[cit24] Parker D., Senanyake K., Williams J. A. G. (1998). Luminescent sensors for pH, pO_2_, halide and hydroxide ions using phenanthridine as a photosensitiser in macrocyclic europium and terbium complexes. J. Chem. Soc., Perkin Trans. 2.

[cit25] Pearson R. G. (1963). Hard and soft acids and bases. J. Am. Chem. Soc..

[cit26] Scarborough Jr R. M., Smith III A. B. (1977). Synthesis and chemical properties of lanthanide cryptates. J. Am. Chem. Soc..

[cit27] Tripier R., Platas-Iglesias C., Boos A., Morfin J.-F., Charbonnière L. J. (2010). Towards fluoride sensing with positively charged lanthanide complexes. Eur. J. Inorg. Chem..

[cit28] Aime S., Botta M., Fasano M., Marques M. P. M., Geraldes C. F. G. C., Pubanz D., Merbach A. E. (1997). Conformational and coordination equilibria on DOTA complexes of lanthanide metal ions in aqueous solution studied by ^1^H-NMR spectroscopy. Inorg. Chem..

[cit29] Blackburn O. A., Chilton N. F., Keller K., Tait C. E., Myers W. K., McInnes E. J. L., Kenwright A. M., Beer P. D., Timmel C. R., Faulkner S. (2015). Spectroscopic and crystal field consequences of fluoride binding by [Yb·DTMA]^3+^ in aqueous solution. Angew. Chem., Int. Ed..

[cit30] Harte A. J., Jensen P., Plush S. E., Kruger P. E., Gunnlaugsson T. (2006). A dinuclear lanthanide complex for the recognition of bis(carboxylates): formation of Terbium(III) luminescent self-assembly ternary complexes in aqueous solution. Inorg. Chem..

[cit31] Andolina C. M., Morrow J. R. (2011). Luminescence resonance energy transfer in heterodinuclear Ln^III^ complexes for sensing biologically relevant anions. Eur. J. Inorg. Chem..

[cit32] Moore J. D., Lord R. L., Cisneros G. A., Allen M. J. (2012). Concentration-independent pH detection with a luminescent dimetallic Eu(III)-based probe. J. Am. Chem. Soc..

[cit33] Tilney J. A., Sørensen T. J., Burton-Pye B. P., Faulkner S. (2011). Self-assembly between dicarboxylate ions and a binuclear europium complex: Formation of stable adducts and heterometallic lanthanide complexes. Dalton Trans..

[cit34] Kofod N., Thomsen M. S., Nawrocki P., Sørensen T. J. (2022). Revisiting the assignment of innocent and non-innocent counterions in lanthanide(III) solution chemistry. Dalton Trans..

[cit35] Tropiano M., Blackburn O. A., Tilney J. A., Hill L. R., Placidi M. P., Aarons R. J., Sykes D., Jones M. W., Kenwright A. M., Snaith J. S., Sørensen T. J., Faulkner S. (2013). Using remote substituents to control solution structure and anion binding in lanthanide complexes. Chem. – Eur. J..

[cit36] Bryden C. C., Reilley C. N., Desreux J. F. (1981). Multinuclear nuclear magnetic resonance study of three aqueous lanthanide shift reagents: complexes with EDTA and axially symmetric macrocyclic polyamino polyacetate ligands. Anal. Chem..

[cit37] Tear L. R., Maguire M. L., Tropiano M., Yao K., Farrer N. J., Faulkner S., Schneider J. E. (2020). Enhancing 31P NMR relaxation rates with a kinetically inert gadolinium complex. Dalton Trans..

[cit38] Fawell J. , Bailey K., Chilton E., Dahi E., Fewtrell L. and Magara Y., Fluoride in drinking-water, World Health Organization, IWA Publishing, London, 2006

[cit39] Hefter G. T. (1989). Solvation of fluoride ions. 3. A review of fluoride solvation thermodynamics in nonaqueous and mixed solvents. Rev. Inorg. Chem..

