# Supplementary material for: Chelating chloride using binuclear lanthanide complexes in water
Source: Chem Sci. 2022 Dec 27;14(5):1194–204. doi: 10.1039/d2sc05417e (PMC9891377; doi:10.1039/d2sc05417e)
Supplement: SC-014-D2SC05417E-s001 [file SC-014-D2SC05417E-s001.pdf]

Electronic Supplementary Material (ESI) for Chemical Science.
This journal is © The Royal Society of Chemistry 2023

Chelating chloride using binuclear lanthanide complexes in water

Carlson Alexander,[a] James A. Thom,[a] Alan M. Kenwright,[b] Kirsten E. Christensen,[a] Thomas Just Sørensen[a,c] and Stephen Faulkner.*[a]

[a] Chemistry Research Laboratory, Department of Chemistry, University of Oxford, Mansfield Road, Oxford, OX1 3TA, UK.
[b] Department of Chemistry, University of Durham, South Road, Durham DH1 3LE, UK.
[c] Nano-Science Centre and Department of Chemistry, University of Copenhagen, 2100 København Ø, Denmark.
*Correspondence to: stephen.faulkner@keble.ox.ac.uk

Contents to supporting information

1. Abbreviations ---------- S5
2. Experimental procedures ---------- S6
2.1 Reagents and solvents ---------- S6
2.2 Vacuum processing, dialysis, centrifugation, TLC and column chromatography S7
2.3 Preparation of buffer and standard solutions ---------- S7
2.4 Physical methods ---------- S8
2.4.1 NMR spectra ---------- S8
2.4.2 Mass spectrometry ---------- S9
2.4.3 Elemental analysis ---------- S9
2.4.4 X-ray crystal structures ---------- S9
2.4.5 Electronic absorption and luminescence spectra ---------- S10
2.4.6 pH meter ---------- S11
2.5 Luminescence titration of host Eu(III) complexes with the guest halide ions S11
2.5.1 Halide binding isotherms ---------- S12
2.6 Chlorine NMR titration and determination of thermodynamic parameters ----- S13
2.7 Softwares used ---------- S13
3. Synthesis of Ligands and Complexes ---------- S14
3.1 Synthesis and characterisation of ligands $(DO3A)_2C$-2 and $(DO3A)_2C$-3 ---------- S14
3.1.1 Synthesis of DO3A(*t*-BuO)$_3$ ---------- S14
3.1.2 Synthesis of (DO3A(*t*-BuO)$_3$)$_2$C-2 and (DO3A(*t*-BuO)$_3$)$_2$C-3 ---------- S15
3.1.3 Synthesis of $(DO3A)_2C$-2 and $(DO3A)_2C$-3 ---------- S17
3.2 Synthesis and characterisation of binuclear lanthanide(III) complexes ---------- S18
3.2.1 Synthesis of binuclear lanthanide(III) complexes ---------- S18
[Eu$_2$(**DO3A)$_2$C-2**] ---------- S19
[Tb$_2$(**DO3A)$_2$C-2**] ---------- S19
[Yb$_2$(**DO3A)$_2$C-2**] ---------- S19
[Eu$_2$(**DO3A)$_2$C-3**] ---------- S20
[Tb$_2$(**DO3A)$_2$C-3**] ---------- S20
[Yb$_2$(**DO3A)$_2$C-3**] ---------- S20
3.3 Synthesis and characterisation of ligand and complexes for [Ln(**pDO3A**)] ----- S21
Figure S1: Synthesis of [Ln(**pDO3A**)] ---------- S21
3.3.1 Synthesis of pDO3A(*t*-BuO)$_3$ ---------- S21
3.3.2 Synthesis of pDO3A ---------- S22
3.3.3 Synthesis of [Ln(**pDO3A**)] ---------- S22
[Eu(**pDO3A**)] ---------- S23
[Tb(**pDO3A**)] ---------- S23

[Yb(**pDO3A**)] -- S23
[Tb(**DOTA**)]$^-$ -- S24

4. Mass and NMR Spectra of Ligands and Complexes ------------------------- S25

Figure S2: ESI-Mass spectrum of (DO3A(*t*-BuO)$_3$)$_2$C-2 ------------------------- S25
Figure S3: ^{1}H NMR spectrum of (DO3A(*t*-BuO)$_3$)$_2$C-2 in CD_2Cl_2 at 203 K ---------- S26
Figure S4: ^{13}C NMR spectrum of (DO3A(*t*-BuO)$_3$)$_2$C-2 in CD_2Cl_2 at 203 K --------- S26
Figure S5: ESI-Mass spectrum of (DO3A(*t*-BuO)$_3$)$_2$C-3 ------------------------- S27
Figure S6: ^{1}H NMR spectrum of (DO3A(*t*-BuO)$_3$)$_2$C-3 in CD_2Cl_2 at 193 K ---------- S28
Figure S7: ^{13}C NMR spectrum of (DO3A(*t*-BuO)$_3$)$_2$C-3 in CD_2Cl_2 at 203 K --------- S28
Figure S8: ESI-Mass spectrum of (DO3A)$_2$C-2 -------------------------------- S29
Figure S9: ^{1}H NMR spectrum of (DO3A)$_2$C-2 in DMSO-d_6 at 403 K ----------------- S30
Figure S10: ^{13}C NMR spectrum of (DO3A)$_2$C-2 in DMSO-d_6 at 403 K ---------------- S30
Figure S11: ESI-Mass spectrum of (DO3A)$_2$C-3 -------------------------------- S31
Figure S12: ^{1}H NMR spectrum of (DO3A)$_2$C-3 in DMSO-d_6 at 403 K ---------------- S32
Figure S13: ^{13}C NMR spectrum of (DO3A)$_2$C-3 in DMSO-d_6 at 403 K ---------------- S32
Figure S14: ^{19}F NMR spectrum of (DO3A)$_2$C-2 in DMSO-d_6 at 298 K ---------------- S33
Figure S15: ^{19}F NMR spectrum of (DO3A)$_2$C-3 in DMSO-d_6 at 298 K ---------------- S33
Figure S16: MALDI-ToF Mass spectrum of [Eu_2(**DO3A)$_2$C-2**] ----------------- S34
Figure S17: MALDI-ToF Mass spectrum of [Tb_2(**DO3A)$_2$C-2**] ----------------- S34
Figure S18: ^{1}H NMR spectrum of [Eu_2(**DO3A)$_2$C-2**] in D_2O at 298 K ---------------- S35
Figure S19: ^{19}F NMR spectrum of [Eu_2(**DO3A)$_2$C-2**] in D_2O at 298 K ---------------- S35
Figure S20: ^{1}H NMR spectrum of [Yb_2(**DO3A)$_2$C-2**] in D_2O at 298 K ---------------- S36
Figure S21: MALDI-ToF Mass spectrum of [Eu_2(**DO3A)$_2$C-3**] ---------------- S36
Figure S22: ESI-Mass spectrum of [Tb_2(**DO3A)$_2$C-3**] ------------------------ S37
Figure S23: MALDI-ToF Mass spectrum of [Yb_2(**DO3A)$_2$C-3**] ---------------- S38
Figure S24: ^{1}H NMR spectrum of [Eu_2(**DO3A)$_2$C-3**] in D_2O at 298 K ---------------- S38
Figure S25: ^{19}F NMR spectrum of [Eu_2(**DO3A)$_2$C-3**] in D_2O at 298 K -------------- S39
Figure S26: ^{1}H NMR spectrum of [Eu_2(**DO3A)$_2$C-3**] in D_2O at 298 K -------------- S39

5. Paramagnetic ^{1}H NMR Spectra of Mono- and Binuclear Ln(III) Complexes S40

Figure S27: Stacked ^{1}H NMR spectra of [Eu_2(**DO3A)$_2$C-2**] with and without KF in D_2O S40
Figure S28: Stacked ^{1}H NMR spectra of [Eu_2(**DO3A)$_2$C-2**] in D_2O, pD 10.12 -------- S40
Figure S29: Stacked ^{1}H NMR spectra of [Eu_2(**DO3A)$_2$C-2**] with and without KF, pD 10.12 S41
Figure S30: Stacked ^{1}H NMR spectra of [Yb_2(**DO3A)$_2$C-2**] with and without KF in CD_3OD S41
Figure S31: Stacked ^{1}H NMR spectra of [Yb_2(**DO3A)$_2$C-2**] in D_2O, pD 10.12 -------- S42
Figure S32: Stacked ^{1}H NMR spectra of [Yb_2(**DO3A)$_2$C-2**] with and without KF, pD 10.12 S42
Figure S33: Stacked ^{1}H NMR spectra of [Eu_2(**DO3A)$_2$C-3**] with and without KF in D_2O S43
Figure S34: Stacked ^{1}H NMR spectra of [Eu_2(**DO3A)$_2$C-3**] in D_2O, pD 10.12 ------- S43
Figure S35: Stacked ^{1}H NMR spectra of [Eu_2(**DO3A)$_2$C-3**] with and without KF, pD 10.12 S44
Figure S36: Stacked ^{1}H NMR spectra of [Yb_2(**DO3A)$_2$C-3**] in D_2O, pD 10.12 -------- S44
Figure S37: Stacked ^{1}H NMR spectra of [Yb_2(**DO3A)$_2$C-3**] with and without KF, pD 10.12 S45
Figure S38: Stacked ^{1}H NMR spectra of [Eu(**pDO3A**)] with increasing KF in D_2O ------ S45
Figure S39: Stacked ^{1}H NMR spectra of [Yb(**pDO3A**)] with increasing KF in D_2O ----- S46
Figure S40: Stacked ^{1}H NMR spectra of [Yb(**pDO3A**)] with increasing KF in CD_3OD --- S46
Figure S41: Stacked ^{1}H NMR spectra of [Tb(**DOTA**)]$^-$ with and without KCl in D_2O --- S47

6. ^{19}F and ^{35}Cl NMR Spectra of Complexes Bound to Fluoride and Chloride, binding isotherms, and van't Hoff plot --- S48

Figure S42: ^{19}F NMR spectrum of [Eu_2(**DO3A**)$_2$**C-2**] with 5 equiv. KF in D_2O ------- S48
Figure S43: ^{19}F NMR spectrum of [Eu_2(**DO3A**)$_2$**C-3**] with 5 equiv. KF in D_2O ------- S48
Figure S44: ^{19}F NMR spectrum of [Eu(**pDO3A**)] with 5 equiv. KF in D_2O ---------- S49
Figure S45: Stacked ^{1}H NMR spectra of [Tb(**pDO3A**)] and [Tb(**DOTA**)]$^-$ with KCl in D_2O S49
Figure S46: [35]Cl NMR titration spectra of [Tb_2(**DO3A**)$_2$**C-3**] with KCl in D_2O at 304 K S50
Figure S47: Binding isotherm for the binding of Cl^- to [Tb_2(**DO3A**)$_2$**C-3**] in D_2O at 304 K S50
Figure S48: [35]Cl NMR titration spectra of [Tb_2(**DO3A**)$_2$**C-3**] with KCl in D_2O at 311 K S51
Figure S49: Binding isotherm for the binding of Cl^- to [Tb_2(**DO3A**)$_2$**C-3**] in D_2O at 311 K S51
Figure S50: [35]Cl NMR titration spectra of [Tb_2(**DO3A**)$_2$**C-3**] with KCl in D_2O at 317 K S52
Figure S51: Binding isotherm for the binding of Cl^- to [Tb_2(**DO3A**)$_2$**C-3**] in D_2O at 317 K S52
Figure S52: Binding isotherm for the binding of Cl^- to [Tb_2(**DO3A**)$_2$**C-3**] in D_2O at 298 K S53
Figure S53: van't Hoff plot for the binding of Cl^- to [Tb_2(**DO3A**)$_2$**C-3**] in D_2O --------- S53
7. Mass Spectra of Eu(III) Complexes Bound to Fluoride and Chloride in Water S54
Figure S54: ESI-Mass spectrum of [Eu_2(**DO3A**)$_2$**C-2**(μ-Cl)]$^-$ recorded in H_2O --------- S54
Figure S55: ESI-Mass spectrum of [Eu_2(**DO3A**)$_2$**C-3**(μ-Cl)]$^-$ recorded in H_2O --------- S55
Figure S56: ESI-Mass spectrum of [Eu(**pDO3A**)F]$^-$ recorded in H_2O -------------- S56
8. Photophysical Studies of Mono- and Binuclear Eu(III) Complexes ----------------- S57
8.1 Electronic absorption spectra -- S57
Figure S57: Electronic absorption spectrum of [Eu_2(**DO3A**)$_2$**C-2**] in water ---------- S57
Figure S58: Electronic absorption spectrum of [Eu_2(**DO3A**)$_2$**C-3**] in water ---------- S58
Figure S59: Electronic absorption spectrum of [Eu(**pDO3A**)] in water --------------- S58
8.2 Excitation and emission spectra -- S59
Figure S60: Excitation & steady-state emission spectra of [Eu_2(**DO3A**)$_2$**C-2**] in H_2O & CH_3OH S59
Figure S61: Excitation & steady-state emission spectra of [Eu_2(**DO3A**)$_2$**C-3**] in H_2O & CH_3OH S60
8.3 Steady-state emission spectra at different pH and pD ------------------------------- S61
Figure S62: Steady-state emission spectra of [Eu_2(**DO3A**)$_2$**C-2**] at different pH ------ S61
Figure S63: Steady-state emission spectra of [Eu_2(**DO3A**)$_2$**C-3**] at different pD ------- S61
Figure S64: Steady-state emission spectra of [Eu_2(**DO3A**)$_2$**C-2**] at different pH ------- S62
Figure S65: Steady-state emission spectra of [Eu_2(**DO3A**)$_2$**C-3**] at different pD ------- S62
8.4. Luminescence Titration with Halides: Spectra, Binding Isotherms, and Speciation Plots S63
Figure S66: Luminescence titration spectra of [Eu_2(**DO3A**)$_2$**C-2**] with KF in PBS ----- S63
Figure S67: Binding isotherm for the binding of F^- with [Eu_2(**DO3A**)$_2$**C-2**] in PBS ----- S63
Figure S68: Luminescence titration spectra of [Eu_2(**DO3A**)$_2$**C-2**] with KF in Tris-HCl buffer S64
Figure S69: Binding isotherm and speciation model for the binding of F^- with [Eu_2(**DO3A**)$_2$**C-2**] in Tris-HCl buffer ------------------------------- S64
Figure S70: Luminescence titration spectra of [Eu_2(**DO3A**)$_2$**C-2**] with KF in CHES buffer S65
Figure S71: Binding isotherm for the binding of F^- with [Eu_2(**DO3A**)$_2$**C-2**] in CHES buffer S65
Figure S72: Luminescence titration spectra of [Eu_2(**DO3A**)$_2$**C-2**] with KF in methanol S66
Figure S73: Binding isotherm and speciation model for the binding of F^- with [Eu_2(**DO3A**)$_2$**C-2**] in methanol --- S66
Figure S74: Luminescence titration spectra of [Eu_2(**DO3A**)$_2$**C-2**] with KCl in water ----- S67
Figure S75: Binding isotherm for the binding of Cl^- with [Eu_2(**DO3A**)$_2$**C-2**] in water ----- S67
Figure S76: Luminescence titration spectra of [Eu_2(**DO3A**)$_2$**C-2**] with KCl in phosphate buffer S68
Figure S77: Binding isotherm for the binding of Cl^- with [Eu_2(**DO3A**)$_2$**C-2**] in phosphate buffer S68
Figure S78: Binding isotherm for the binding of Cl^- with [Eu_2(**DO3A**)$_2$**C-2**] in CHES buffer S69
Figure S79: Binding isotherm for the binding of F^- with [Eu_2(**DO3A**)$_2$**C-2**] in water --- S69

Figure S80: Luminescence titration spectra of [Eu_2(**DO3A**)$_2$**C-2**] with KBr in water --- S70
Figure S81: Emission trend for the interaction of Br^- to [Eu_2(**DO3A**)$_2$**C-2**] in water --- S70
Figure S82: Luminescence titration spectra of [Eu_2(**DO3A**)$_2$**C-2**] with KI in water --- S71
Figure S83: Emission trend for the interaction of I^- to [Eu_2(**DO3A**)$_2$**C-2**] in water --- S71
Figure S84: Luminescence titration spectra of [Eu_2(**DO3A**)$_2$**C-3**] with KF in water ---- S72
Figure S85: Binding isotherm and speciation model for the binding of F^- with [Eu_2(**DO3A**)$_2$**C-3**] in water -- S72
Figure S86: Luminescence titration spectra of [Eu_2(**DO3A**)$_2$**C-3**] with KF in PBS ---- S73
Figure S87: Binding isotherm and speciation model for the binding of F^- with [Eu_2(**DO3A**)$_2$**C-3**] in PBS -- S73
Figure S88: Luminescence titration spectra of [Eu_2(**DO3A**)$_2$**C-3**] with KF in Tris-HCl buffer S74
Figure S89: Binding isotherm and speciation model for the binding of F^- with [Eu_2(**DO3A**)$_2$**C-3**] in Tris-HCl buffer -------------------------------- S74
Figure S90: Luminescence titration spectra of [Eu_2(**DO3A**)$_2$**C-3**] with KF in CHES buffer S75
Figure S91: Binding isotherm and speciation model for the binding of F^- with [Eu_2(**DO3A**)$_2$**C-3**] in CHES buffer -------------------------------- S75
Figure S92: Luminescence titration spectra of [Eu_2(**DO3A**)$_2$**C-3**] with KF in methanol S76
Figure S93: Binding isotherm and speciation model for the binding of F^- with [Eu_2(**DO3A**)$_2$**C-3**] in methanol --------------------------------------- S76
Figure S94: Luminescence titration spectra of [Eu_2(**DO3A**)$_2$**C-3**] with KCl in water ---- S77
Figure S95: Binding isotherm for the binding of Cl^- with [Eu_2(**DO3A**)$_2$**C-3**] in water -- S77
Figure S96: Luminescence titration spectra of [Eu_2(**DO3A**)$_2$**C-3**] with KCl in phosphate buffer S78
Figure S97: Binding isotherm for the binding of Cl^- with [Eu_2(**DO3A**)$_2$**C-3**] in phosphate buffer S78
Figure S98: Luminescence titration spectra of [Eu_2(**DO3A**)$_2$**C-3**] with KCl in CHES buffer S79
Figure S99: Binding isotherm and speciation model for the binding of Cl^- with [Eu_2(**DO3A**)$_2$**C-3**] in CHES buffer -------------------------------- S79
Figure S100: Luminescence titration spectra of [Eu_2(**DO3A**)$_2$**C-3**] with KBr in water --- S80
Figure S101: Emission trend for the interaction of Br^- to [Eu_2(**DO3A**)$_2$**C-3**] in water --- S80
Figure S102: Luminescence titration spectra of [Eu_2(**DO3A**)$_2$**C-3**] with KI in water --- S81
Figure S103: Emission trend for the interaction of I^- to [Eu_2(**DO3A**)$_2$**C-3**] in water --- S81
Figure S104: Luminescence titration spectra of [Eu(**pDO3A**)] with KF in water ------ S82
Figure S105: Binding isotherm for the binding of F^- with [Eu(**pDO3A**)] in water ------ S82
Figure S106: Luminescence titration spectra of F^- with [Eu(**pDO3A**)] in methanol ------ S83
Figure S107: Binding isotherm and speciation model for the binding of F^- with [Eu(**pDO3A**)] in methanol -- S83
Figure S108: Luminescence titration spectra of [Eu(**pDO3A**)] with KCl in water ------ S84
Figure S109: Emission trend for the interaction of Cl^- with [Eu(**pDO3A**)] in water ------ S84
8.5. Time-resolved Luminescence Lifetime Data for Eu(III) Complexes ------------------ S85
Figure S110: Time-resolved luminescence lifetime of [Eu_2(**DO3A**)$_2$**C-2**] with increasing KF in H_2O and D_2O -- S85
Figure S111: Time-resolved luminescence lifetime of [Eu_2(**DO3A**)$_2$**C-2**] with increasing KF in CH_3OH and CD_3OD -- S86
Figure S112: Time-resolved luminescence lifetime of [Eu_2(**DO3A**)$_2$**C-2**] at different pH and pD S87
Table S1: Luminescence lifetimes for [Eu_2(**DO3A**)$_2$**C-2**] at different pH and pD S88
Table S2: Luminescence lifetimes for [Eu_2(**DO3A**)$_2$**C-2**] with increasing KF in H_2O and D_2O S88
Table S3: Luminescence lifetimes for [Eu_2(**DO3A**)$_2$**C-2**] with increasing KF in

CH$_3$OH and CD$_3$OD -- S88
Figure S113: Time-resolved luminescence lifetime of [Eu$_2$(**DO3A**)$_2$**C-3**] with increasing KF in H_2O and D_2O -- S89
Figure S114: Time-resolved luminescence lifetime of [Eu$_2$(**DO3A**)$_2$**C-3**] with increasing KF in CH$_3$OH and CD$_3$OD -- S90
Figure S115: Time-resolved luminescence lifetime of [Eu$_2$(**DO3A**)$_2$**C-3**] at different pH and pD S91
Table S4: Luminescence lifetimes for [Eu$_2$(**DO3A**)$_2$**C-3**] at different pH and pD S92
Table S5: Luminescence lifetimes for [Eu$_2$(**DO3A**)$_2$**C-3**] with increasing KF in H_2O and D_2O S92
Table S6: Luminescence lifetimes for [Eu$_2$(**DO3A**)$_2$**C-3**] with increasing KF in CH$_3$OH and CD$_3$OD -- S92
Figure S116: Time-resolved luminescence lifetime of [Eu(**pDO3A**)] with increasing KF in H_2O and D_2O -- S93
Figure S117: Time-resolved luminescence lifetime of [Eu(**pDO3A**)] with increasing KF in CH$_3$OH and CD$_3$OD -- S94
Table S7: Luminescence lifetimes for [Eu(**pDO3A**)] with increasing KF in H_2O and D_2O S95
Table S8: Luminescence lifetimes for [Eu(**pDO3A**)] with increasing KF in CH$_3$OH and CD$_3$OD -- S95
9. Limits of Detection and Quantification with KCl in Water ------------------------ S96
Figure S118: LOD and LOQ plots for [Eu$_2$(**DO3A**)$_2$**C-2**] with KCl in water ---------- S96
Figure S119: LOD and LOQ plots for [Eu$_2$(**DO3A**)$_2$**C-3**] with KCl in water ---------- S96
10. DYNAFIT Script -- S97
10.1 A typical DYNAFIT script for a single binding event -------------------------- S97
Figure S120: A typical binding isotherm generated from DYNAFIT® for a one binding event S97
10.2 A typical DYNAFIT script for a two-binding event ------------------------ S98
Figure S121: A typical binding isotherm generated from DYNAFIT® for a two-binding event S98
10.3. Speciation modelling -- S98
11. X-ray Crystal Structures of Ligands and Complexes ------------------------ S99
Figure S122: Single crystal X-ray structure of (DO3A(*t*-BuO$_3$))$_2$C-2 ------------ S99
Table S9: Crystal data and structure refinement for (DO3A(*t*-BuO)$_3$)$_2$C-2 ------- S100
Figure S123: Single crystal X-ray structure of pDO3A(*t*-BuO$_3$) ------------------------ S101
Table S10: Crystal data and structure refinement for pDO3A(*t*-BuO)$_3$ ----------- S102
Figure S124: X-ray crystal structure of [Eu$_2$(**DO3A**)$_2$**C-3**] and [Yb$_2$(**DO3A**)$_2$**C-3**]------- S103
Table S11: Crystal data and structure refinement for [Ln$_2$(**DO3A**)$_2$**C-3**] [Ln = Eu(III), Yb(III)] S104
Figure S125: Single crystal X-ray structure of [Yb$_2$(**DO3A**)$_2$**C-3**(2F)]$^{2-}$ ----------------- S105
Table S12: Crystal data and structure refinement for [Yb$_2$(**DO3A**)$_2$**C-3**(2F)]$^{2-}$ -------- S106
Figure S126: Single crystal X-ray structure of [Eu(**pDO3A**)] and [Yb(**pDO3A**)] ---- S107
Table S13: Crystal data and structure refinement for [Ln(**pDO3A**)] [Ln = Eu(III), Yb(III)] S108
12. References -- S109

1. Abbreviations

Ln	lanthanide
cyclen	1,4,7,10-tetraazacyclododecane
cycl.	cyclen
DO3A(*t*-BuO)$_3$	*tert*-butyl-2-{4,7-bis[2-(*tert*-butoxy)-2-oxoethyl]-1,4,7,10-tetraazacyclododecan-1-yl}acetate
DO3A	1,4,7,10-tetraazacyclododecane-1,4,7-triacetic acid

DOTA	1,4,7,10-tetraazacyclododecane-1,4,7,10-tetraacetic acid
C-2	ethane bridge
C-3	propane bridge
pDO3A	2-[4,7-bis(carboxymethyl)-10-(prop-2-yn-1-yl)-1,4,7,10-tetraazacyclododecan-1-yl]acetic acid
DCTB	*trans*-2-[3-(4-*tert*-butylphenyl)-2-methyl-2-propenylidene]malononitrile
PBS	phosphate buffered saline
Tris-HCl	*tris*(hydroxymethyl)aminomethane hydrochloride
HEPES	4-(2-hydroxyethyl)-1-piperazineethanesulfonic acid
CHES	2-(cyclohexylamino)ethanesulfonic acid
mp	melting point
br	broad
s	singlet
m	multiplet
MHz	megahertz
DMSO-d_6	deuterated dimethyl sulfoxide
CD_2Cl_2	deuterated dichloromethane
CD_3OD	deuterated methanol
Calc.	calculated
KF	potassium fluoride
KCl	potassium chloride
CI	confidence interval
equiv	equivalent
eq	equation

2. Experimental procedures

2.1 Reagents and solvents

1,2-dibromoethane (99%), 1,3-dibromopropane (99%), sodium bicarbonate (99.7%), sodium hydroxide (98%), propargyl bromide solution (80 wt. % in toluene, 0.3% MgO stabiliser), sodium dihydrogen phosphate (anhydrous, 99%), sodium hydrogen phosphate (anhydrous, 99%), acetonitrile, chloroform, methanol, pentane, ethanol, diethyl ether, toluene, Tris-HCl (Trizma® hydrochloride) (99%), HEPES sodium salt (≥ 99.5%, titration), PBS tablet, CHES sodium salt, europium(III) trifluoromethanesulfonate hydrate (98%), ytterbium(III) trifluoromethanesulfonate (98%) and silica gel 60 (Sigma Aldrich); cyclen, DOTA (CheMatech, Dijon, France); caesium carbonate (Fluorochem); trifluoroacetic acid (99%) (Acros Organics); dichloromethane (Honeywell); hydrochloric acid and potassium iodide (Fisher Scientific); anhydrous sodium acetate (99.0%), anhydrous sodium sulphate (99.99%), neutral alumina (activated, Brockmann grade II), ethylene glycol dimethyl ether (glyme, 99%, stabilised with BHT), *tert*-butylbromoacetate (98%), terbium(III) trifluoromethanesulfonate (98%) (Alfa Aesar); CD_3OD ≥ 99.8 atom % D, CD_2Cl_2 99.8 atom % D, DMSO-d_6 99.9 atom % D, D_2O 99.9 atom % D, trifluoroacetic acid-*d* (99.5 atom % D) and NaOD (40 wt. % solution in D_2O, 99+ atom % D) (Sigma Aldrich); potassium fluoride (Riedel-de Haën®); potassium chloride and potassium bromide (BDH®) were used as received. Deionised water was used

throughout the study which was obtained from Elga PURELAB® Chorus 1 complete with conductivity of 18.2 MΩ.cm (Type I^+/I).

2.2 Vacuum processing, dialysis, centrifugation, TLC and column chromatography

Buchi Rotavapor® R-200 and Heidolph Laborota 4000-efficient Rotary Evaporator were used to evaporate solvents under reduced pressure and compounds were dried in high-vac Schlenk line with an Edwards RV3 Vacuum Pump. Dialysis of the complexes were performed using Spectra-Por® Float-A-Lyzer® G2 dialysis membrane tubing made from cellulose ester (Spectrum Labs) with a molecular weight cut-off of 500-1000 Da. Centrifugations were performed using Beckman Coulter® Allegra® X-12R Benchtop Centrifuge. All centrifugations were performed at 3750 RPM for 6 min at 4 °C. Column chromatography was performed using Geduran silica gel 60 (0.040-0.063 mm mesh, Sigma Aldrich) and neutral alumina (activated, Brockmann grade II). Analytical thin layer chromatography (TLC) was performed on aluminium sheet supported silica gel plates coated with silica gel 60 F_{254} (0.2 mm, Merck) and aluminium sheet supported plates coated with neutral alumina 60 F_{254} (0.2 mm, Merck) using different solvent systems as mobile phase. The compounds were visualised in TLC by potassium permanganate stain prepared as per literature protocol.[1]

2.3 Preparation of buffer and standard solutions

In water

PBS buffer (0.01 M, pH 7.4) was prepared by dissolving one PBS tablet in 200 mL deionised water.

Tris-HCl buffer (0.01 M, pH 7.4) was prepared by dissolving 242.28 mg of Trizma base in 100 mL deionised water followed by the dropwise addition of 138 μL of concentrated HCl (37%, 12.1 M), and made up to 200 mL using deionised water.

Phosphate buffer (0.01 M, pH 7.4) was prepared by dissolving sodium hydrogen phosphate (505 mg) and sodium dihydrogen phosphate (85 mg) in 250 mL deionised water. pH adjusted using 0.01M aqueous NaOH.

Acetate buffer (0.01 M, pH 3.98) was prepared by dissolving 7 mg of sodium acetate in 20 mL deionised water followed by the addition of 18.12 μL of glacial acetic acid under stirring and adjusting the pH to 3.98 using 0.01 M aqueous CF_3COOH. The final volume was adjusted to 40 mL using deionised water.

HEPES buffer (0.01 M, pH 6.97) was prepared by dissolving 100 mg of HEPES sodium salt in 20 mL deionised water and adjusting the pH using 0.01 M aqueous NaOH. The final volume was adjusted to 40 mL using deionised water.

CHES buffer (0.01 M, pH 9.98) was prepared by dissolving 80 mg of CHES sodium salt in 20 mL deionised water and adjusting the pH using 0.01 M aqueous NaOH. The final volume was adjusted to 40 mL using deionised water.

Aqueous NaOH (0.01 M) was prepared by dissolving 40 mg of NaOH in 50 mL deionised water and making up to 100 mL in a standard flask. Aqueous CF_3COOH (0.01 M) was prepared by the dropwise addition of 76.58 μL CF_3COOH in 50 mL deionised water and making up to 100 mL in a standard flask.

In deuterium oxide

Acetate buffer (0.01 M, pD 4.1) was prepared by dissolving 3 mg of sodium acetate in 4 mL D_2O followed by the addition of 3.8 μL glacial acetic acid under stirring and adjusting the pH using 0.01 M aqueous CF_3COOD. The final volume was adjusted to 10 mL using D_2O.

HEPES buffer (0.01 M, pD 7.05) was prepared by dissolving 20 mg of HEPES sodium salt in 4 mL D_2O and adjusting the pH using 0.01 M aqueous NaOD. The final volume was adjusted to 10 mL using D_2O.

CHES buffer (0.01 M, pD 10.12) was prepared by dissolving 20 mg of CHES sodium salt in 4 mL D_2O and adjusting the pH using 0.1 M aqueous NaOD and 0.01 M NaOD. The final volume was adjusted to 10 mL using D_2O.

Solution of NaOD (0.1 M) was prepared by dissolving 102.4 μL of NaOD in 5 mL D_2O and diluted to 10 mL. Solution of NaOD (0.01 M) was prepared by dissolving 10.2 μL of NaOD in 5 mL D_2O and diluted to 10 mL. Solution of CF_3COOD (0.01 M) was prepared by dissolving 7.8 μL of CF_3COOD in 5 mL D_2O and diluted to 10 mL.

2.4 Physical Methods

2.4.1 NMR spectra

NMR spectra were recorded on a Bruker Biospin AG Avance III HD Nanobay 400 MHz NMR Spectrometer equipped with a 9.4 T magnet (^{1}H 400.2 MHz, ^{13}C 100.6 MHz, ^{19}F 376.5 MHz) or Bruker Avance III 500 MHz NMR equipped with a 11.75 T magnet (^{1}H 500 MHz, ^{13}C 125.7 MHz, ^{19}F 470.4 MHz, ^{35}Cl 49 MHz). All NMR spectra were recorded in deuterated solvents (Sigma Aldrich). Chemical shifts were assigned by comparison with the

residual proton and carbon resonances of the solvents.[2] The NMR spectrometers were set with $(CH_3)_4Si$ in $CDCl_3$ as the internal reference for ^1H and ^{13}C; $CFCl_3$ in $CDCl_3$ for ^{19}F; and NaCl in D_2O for ^{35}Cl NMR, ($\delta = 0$ ppm). NMR samples were placed in Norrell® standard series™ 5 mm NMR tubes (600 MHz frequency, Sigma Aldrich) and the spectra were recorded.

2.4.2 Mass spectrometry

ESI mass spectra were recorded on the Thermofisher Exactive™ Plus Mass Spectrometer with a Waters Acuity UPLC system. LRMS was recorded on a Waters LCT Premier XE bench-top orthogonal acceleration time-of-flight ESI mass spectrometer and data processed in Mestrenova. MALDI-TOF mass spectra were recorded on a Bruker AutoFlex Mass Spectrometer. All MALDI-TOF MS were run on a DCTB matrix.

2.4.3 Elemental analysis

CHN microanalyses were carried out on Thermo Scientific™ FLASH 2000 CHNS/O Analyzer at London Metropolitan University, London. All samples were submitted in pre-scored long stem Vacule® cryogenic glass ampules (Sigma Aldrich) sealed using a blow torch.

2.4.4 X-Ray crystal structures

Low temperature single crystal X-ray diffraction data were collected using a (Rigaku) Oxford Diffraction SuperNova A diffractometer. Raw frame data were reduced using CrysAlisPro and the structure was solved from the integrated intensities with charge-flipping using ‘Superflip’.[3a] The structure was refined using full-matrix least squares on F^2 using the CRYSTALS suite[3b,3c] as per the SI (CIF). Crystallographic data have been deposited with the Cambridge Crystallographic Data Centre as supplemental publication number CCDC 2201928 – 2201934 and can be obtained *via* www.ccdc.cam.ac.uk/data_request/cif.

Single crystal X-ray structures reported in this work were obtained as follows: $(DO3A(t\text{-}BuO)_3)_2C$-2 was obtained by vapour diffusion of pentane into concentrated solution of the compound in deuterated chloroform; pDO3A(*t*-BuO)$_3$ obtained from recrystallisation in toluene; [Ln$_2$(**DO3A**)$_2$**C-3**] (Ln = Eu(III) and Yb(III)) obtained by vapour diffusion of glyme into concentrated solution of the complex in deuterium oxide; [Ln(**pDO3A**)] (Ln = Eu(III) and Yb(III)) obtained by vapour diffusion of acetone into concentrated solution of the complex in deionised water; [Yb$_2$(**DO3A**)$_2$**C-3**(2F)]$^{2-}$ obtained by vapour diffusion of glyme into concentrated solution of the complex in deuterium oxide with KF salt added to facilitate crystallisation.

2.4.5 Electronic absorption and luminescence spectra

Electronic absorption measurements were recorded on a Jasco V-770 UV-Visible/NIR spectrophotometer operated under Spectra Manager™ suite. Points were recorded at 0.2 nm interval with UV/Vis bandwidth of 1 nm, UV/Vis response of 0.06 sec in continuous scan mode at the rate of 400 nm/min. Electronic absorption spectra was recorded for solution of compounds taken in a 1 mL Hellma Analytics precision quartz cell cuvette (SUPRASIL®, type: 114F-QS). Steady-state excitation and emission spectra were recorded on a Horiba Jobin Yvon Fluorolog® 3-12 Fluorometer equipped with a Hamamatsu R928 detector and a double-grating emission monochromator. S1/R1 response was used throughout as luminescence output. A 2" square unmounted longpass 550 nm filter (FGL 550S) or 400 nm filter (FGL400S) fabricated using a 2 mm thick Schott® coloured glass from Thor labs was used while recording steady-state emission spectra of Eu(III) complexes with a slit width of 20 nm and a band pass of 1 nm for excitation at 393 nm. Points were recorded at 1 nm intervals with a 0.1 s to 0.5 s integration time. S1/R1 response was used throughout to obtain the steady-state luminescence output. Steady-state emission titrations in CHES buffer with KCl was performed in Horiba Jobin Yvon Fluoromax® 4 Spectrofluorometer using 495 nm (FGL495S) fabricated using a 2 mm thick Schott® coloured glass from Thor labs filter and employing the same conditions as used in Fluorolog® 3-12. Time-resolved lifetime measurements were made on Fluorolog® 3-12 for the Eu(III) complexes with a slit width of 20 nm and band pass of 14.7 nm by exciting at 393 nm and monitoring emission at 616 nm with a max delay of 8 or 12 ms. Luminescence lifetimes were obtained by tail fit for Eu(III) complexes using exponential decay function in Origin 8 operated with the FluorEssence™ software for Windows®. All lifetimes gave satisfactory fitting using a mono exponential decay function; fitting to a double exponential decay did not improve the fit. S1 response was used throughout for obtaining the time-resolved and steady-state excitation output. Luminescence lifetimes were calculated for Eu(III) complexes using the modified Horrock's equation,[4] $q = A\,(kH - kD - B)$ where A = 1.2 in water and 2.4 in methanol, B = 0.25 in water and 0.125 in methanol, kH and kD (in ms^{-1}) correspond to the rate constant of the lifetime decay in the given solvent and the corresponding deuterated solvent.[4] The steady-state and time resolved measurements were carried out for solution of samples taken in a 3500 μL quartz macro fluorescence cuvette with screw cap (Starna Scientific, type: 3/GL14/Q/10). All measurements were performed at ambient temperature (22 °C) and averaged to 3 measurements.

2.4.6 pH meter

Hanna Instrument pH 210 Microprocessor pH Meter with a HI 1131B electrode was used for the pH measurements of complexes in water and deuterium oxide. The pH meter was calibrated using pH 4.01 and 7.01 buffer solutions (Hannah Instruments). The electrodes were stored in inorganic cleaning solution (Hanna Instruments) for 20 min and washed with deionised water before use. In deuterium oxide, the pD of the solution was determined using the equation pD = pH + 0.45 (pH is the "pH meter reading" obtained from the pH meter standardised to read pH in water).[5]

2.5 Luminescence titration of the host Eu(III) complexes with the guest halide ions

The binding of halides to the complexes [Eu_2(**DO3A**)$_2$**C-2**] and [Eu_2(**DO3A**)$_2$**C-3**] was quantified to compute the binding constants (K) by steady-state luminescence titration, employing the non-dilution method,[6] by fixing the concentration of the host complex and varying the concentration of the guest (halides). This was accomplished by carrying out the luminescence titrations by adding aliquots of halide (guest), pre-dissolved in a solution containing the host complex, to the solution of a fixed concentration of the host complex to maintain the concentration of the complex uniform, whilst increasing the concentration of the guest. Stock solution of the host complex (2 mL) was taken in a cuvette and the solution of the guest was added in aliquots using Gilson® micropipettes. 30 – 40 aliquots were added in total with each addition mixed by agitation using 1000 μL micropipette inside the cuvette, left aside for 2 min and the measurements were taken. The stock concentration of the host complex was 1 mM and the stock concentration of the guest was 0.02 M.

Luminescent lanthanide complexes have been used as probes for the detection of anions. Eu(III) complexes have sharp line-like emission spectra, which allows ratiometric analysis by measuring the change in intensity of one emission band relative to the intensity of an almost stationary band.[7] Anion binding occurs directly at the Ln(III) metal centre by the displacement of one or more inner-sphere water molecules with concomitant variations in the Ln(III) coordination environment and the local ligand field, resulting in changes in the emission intensity, spectral form, and lifetime of the complex. Notably, for Eu(III) complexes the changes in emission spectral form induced by anion binding is typically characterised by a large change in the intensity of the electric-dipole allowed, hypersensitive $^5D_0 \rightarrow {}^7F_2$ ($\Delta J = 2$) emission band relative to the intensity of the magnetic-dipole allowed $^5D_0 \rightarrow {}^7F_1$ ($\Delta J = 1$) emission band whose splitting and intensity is relative to the local symmetry at the metal centre

and to the magnetic anisotropy factor, *D*.[7] Therefore, the steady-state emission spectra of the host complexes [Eu_2(**DO3A**)$_2$**C-2**] and [Eu_2(**DO3A**)$_2$**C-3**] were monitored upon the addition of each aliquot portion of the guest and the area under the emission bands corresponding to the $\Delta J = 2$ and $\Delta J = 1$ transitions integrated. This ratiometric change is then plotted as a function of the concentration of the guest added to the host (equivalent guests) and the concentration of the halides is determined alongside a known concentration curve.[7] The resulting titration curve, known as the binding isotherm, is then fitted to a mathematical model corresponding to the postulated chemical equilibria (eq. 1 to 3) to obtain the association constants for one- (K_1) or two- (K_2) or three binding events (K_3) in M^{-1} *via* an iterative least square fitting process using DYNAFIT® software[8] with uncertainty (±) expressed as coefficient of variation in percentage. However, in luminescence titrations involving fluoride binding in neat solvents, the change in form and shape of the emission intensities dramatically change over the increasing addition of fluoride, such that integrating the area under the emissive bands do not account for the changes in the crystal-field splitting.[6] Therefore, selected emission maxima in $\Delta J = 0$ and $\Delta J = 1$ bands are used to quantify binding by plotting them *via* an iterative least square fitting process,[6] similar to the ratiometric method mentioned above.

$$[Host] + [Guest] \underset{K_1}{\Leftrightarrow} [Host - Guest] \qquad K_1 = \frac{[Host-Guest]}{[Host][Guest]} \qquad (1)$$

$$[Host - Guest] + [Guest] \underset{K_2}{\Leftrightarrow} [Host - Guest_2] \qquad K_2 = \frac{[Host-Guest_2]}{[Host-Guest][Guest]} \qquad (2)$$

$$[Host - Guest_2] + [Guest] \underset{K_3}{\Leftrightarrow} [Host - Guest_3] \qquad K_3 = \frac{[Host-Guest_3]}{[Host-Guest_2][Guest]} \qquad (3)$$

2.5.1 Halide binding isotherms

Data on all luminescence titrations of the Eu(III) complexes with the guest ions were processed and plotted using Origin software. For the ratiometric study of binding interactions, the area under the emission bands were integrated by summing over the wavelength range under each band (585 – 603 nm for $\Delta J = 1$ and 610 – 628 nm for $\Delta J = 2$ transitions) and the ratio was performed in Origin. For luminescence titrations involving fluoride binding in neat solvents (i.e., deionised water and methanol), binding isotherms were generated after baseline subtraction from an emission wavelength. Baseline corrections were carried out for the $\Delta J = 0$ and $\Delta J = 1$ transitions by subtracting the intensity of the emission at 582 nm and 604 nm which has no characteristic Eu emission, respectively. Baseline subtractions for other titrations did not show any difference. All binding isotherms were generated from DYNAFIT® version 4.08 using its default setting (Trust region algorithm in confidence intervals at 95% probability level) which was transported to replot in Origin. Few outliers from the dataset were masked to

improve the quality of the iterative fit for binding isotherms. Masking outliers only improved the fit but did not affect the overall result. All speciation plots were generated using DYNAFIT®.

2.6 Chlorine NMR titrations and determination of thermodynamic parameters

^{35}Cl NMR titrations with the binuclear Tb complexes was performed using the non-dilution method,[6] where the concentration of the complex (host) was kept consistent and the concentration of KCl (guest) was increased. This was accomplished by carrying out the NMR titrations by adding aliquots of guest (pre-dissolved in a solution containing the host), to the solution of a fixed concentration of the host to maintain the concentration of the host uniform, whilst increasing the concentration of the guest. 400 μL of guest was taken in the NMR tube containing a capillary tube of saturated KCl in D_2O. ~19 aliquots of KCl was added to the NMR tube in total (using Gilson® micropipettes) with each addition mixed by agitation of the NMR tube for 2 minutes and the spectrum recorded. The stock concentration of the host complex was 0.035 M and the stock concentration of the guest was 1.4 M. Each ^{35}Cl NMR spectrum was referenced to saturated KCl in D_2O (δ = 0 ppm). 64 scans were used to record each spectrum. The bound chloride chemical shift upon the addition of KCl was observed and these chemical shifts were used in generating a binding isotherm using DYNAFIT®. The thermodynamic parameters associated with chloride binding to [Tb_2(**DO3A**)$_2$**C-3**] was determined from the binding constant (K_1) obtained from ^{35}Cl NMR titrations performed at 298 K, 304 K, 311 K, and 317 K. A van't Hoff plot of -Rln(K_1) against T^{-1} from the titrations at 4 different temperatures mentioned above, allowed the determination of enthalpy and entropy contributions associated to the binding as the slope and intercept, respectively.[9]

2.7 Softwares used

Structures were drawn using Chem Draw 20 and IUPAC names were predicted using Marvin Sketch version 21.16. NMR data were processed using Mestrenova software version 14.2. The binding of halides with the complexes is determined *via* an iterative least square fitting process using DYNAFIT® version 4.08 software.[8] Speciation models were generated using DYNAFIT®. All electronic absorption, luminescence, binding isotherms, speciation plots, and fitted data were plotted using Origin software version 2020b. Images were generated using Inkscape. Single crystal X-ray structures were rendered using Diamond software version 3.7.[10]

3. Synthesis of Ligands and Complexes

3.1 Synthesis and characterisation of ligands, $(DO3A)_2C$-2 and $(DO3A)_2C$-3

3.1.1 Synthesis of *tert*-butyl 2-{4,7-bis[2-(*tert*-butoxy)-2-oxoethyl]-1,4,7,10-tetraaza-cyclododecan-1-yl}acetate (DO3A(*t*-BuO)$_3$)

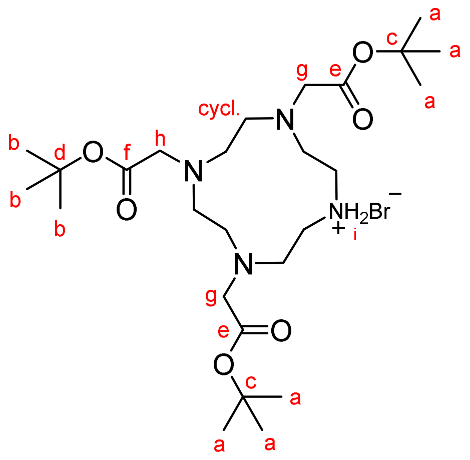

DO3A(*t*-BuO)$_3$

This compound was synthesised by modifying the reported procedure.[11] To a suspension of cyclen (5.00 g, 28.4 mmol, 1.00 equiv) in 250 mL acetonitrile was added sodium bicarbonate (7.36 g, 87.0 mmol, 3.05 equiv) and stirred at 25 °C for 30 min. *tert*-butylbromoacetate (13 mL, 87.0 mmol, 3.05 equiv) in 50 mL acetonitrile was added dropwise over 2 h at 0 °C to this mixture and stirred for 72 h at 25 °C. This reaction mixture was filtered to remove the inorganic solids and the resulting filtrate was reduced under pressure to produce an off-white solid which was washed with 500 mL toluene for 24 h under stirring, followed by filtration to afford the crude product. This process was repeated again to afford the title compound as a white powder (7.50 g, 14.6 mmol, 51%); mp 164–168 °C (lit.[11] 178–180 °C); R_f = 0.42 (silica, CH_2Cl_2/CH_3OH, 95:5); ^{1}H NMR (400 MHz, DMSO-d_6, 298 K) δ (ppm): 9.47 – 8.49 (2 H, br s, H^{i} N*H*.*H*Br), 3.74 – 2.36 (22 H, br m, $H^{g,h,cycl.}$), 1.82 – 1.00 (27 H, br s, $H^{a,b}$); ^{13}C NMR (101 MHz, DMSO-d_6, 298 K) δ (ppm): 170.5 (C^{e}), 169.9 (C^{f}), 80.5 (C^{d}), 80.4 (C^{c}), 56.1 (C^{g}), 51.7 ($C^{cycl.}$), 50.4 (C^{h}), 49.7 – 45.6 ($C^{cycl.}$), 27.81 (C^{a}), 27.79 (C^{b}); HRMS (ESI in CH_3OH) *m*/*z* (% relative intensity): $[M + H]^+$ found, 515.3796 (100%). Calc. for $C_{26}H_{51}N_4O_6$, M_r = 515.3803.

3.1.2 General procedure 1 for the synthesis of *tert*-butyl 2-{4,7-bis[2-(*tert*-butoxy)-2-oxoethyl]-10-(2-{4,7,10-tris[2-(*tert*-butoxy)-2-oxoethyl]-1,4,7,10-tetraazacyclododecan-1-yl}ethyl)-1,4,7,10-tetraazacyclododecan-1-yl}acetate ((DO3A(*t*-BuO)$_3$)$_2$C-2) and *tert*-butyl 2-{4,7-bis[2-(*tert*-butoxy)-2-oxoethyl]-10-(3-{4,7,10-tris[2-(*tert*-butoxy)-2-oxoethyl]-1,4,7,10-tetraazacyclododecan-1-yl}propyl)-1,4,7,10-tetraazacyclododecan-1-yl}acetate ((DO3A(*t*-BuO)$_3$)$_2$C-3)

DO3A(*t*-BuO)$_3$ (2.00 equiv) was dissolved in 100 mL acetonitrile and stirred with caesium carbonate (3.00 equiv) for 30 min at 25 °C. To this, the appropriate dibromoalkane (1.05 equiv) was added dropwise for 1 h and stirred under reflux for 48 h. The reaction mixture was cooled to 25 °C and filtered. The filtrate was evaporated under reduced pressure and the resulting yellow oily residue suspended in 10 mL water and extracted with chloroform (3 × 20 mL). The organic layers were combined, dried over anhydrous sodium sulfate, filtered, and evaporated to dryness. The crude orange product was purified by flash column chromatography to afford the title compound.

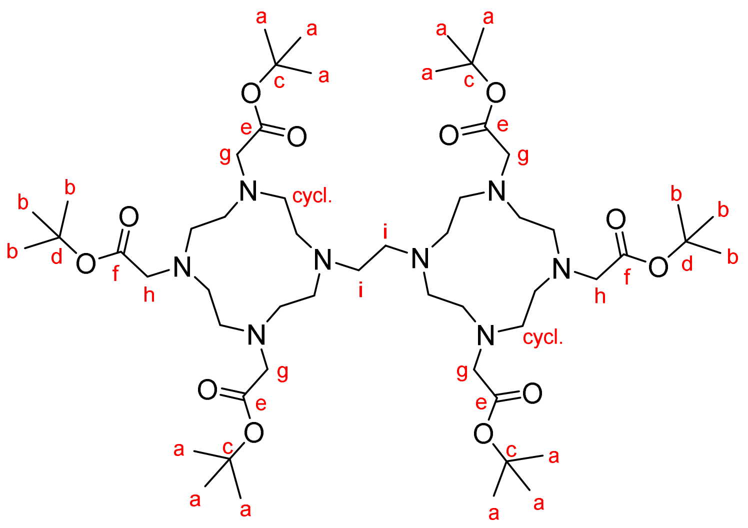

(DO3A(*t*-BuO)$_3$)$_2$C-2

(DO3A(*t*-BuO)$_3$)$_2$C-2: Prepared from **DO3A(*t*-BuO)$_3$** (3.80 g, 6.38 mmol, 2.00 equiv) and 1,2-dibromoethane (289 μL, 3.35 mmol, 1.05 equiv) following general procedure **1**. Flash column chromatography over silica gel by gradient elution with CH_2Cl_2/CH_3CH_2OH (100:0→99:1→98:2→97:3→96:4→94:6→90:10, *v*/*v*) afforded the desired product as a yellow foamy solid (1.50 g, 1.42 mmol, 42%); R_f = 0.67 (silica, CH_2Cl_2/CH_3OH, 9:1); mp 175–180 °C; ^{1}H NMR (500 MHz, CD_2Cl_2, 203 K) δ (ppm): 3.55 – 1.76 (50 H, br m, $H^{g,h,i,cycl.}$ + 2 *H*Br), 1.73 – 1.18 (54 H, br m, $H^{a,b}$); ^{13}C NMR (126 MHz, CD_2Cl_2, 203 K) δ (ppm): 173.5 – 172.2

($C^{e,f}$), 83.1 (C^{d}), 82.2 (C^{c}), 55.5 – 47.0 ($C^{g,h,i,cycl.}$), 27.4 – 27.0 ($C^{a,b}$); HRMS (ESI in CH_3OH) *m/z* (% relative intensity): $[M + H]^+$ found, 1055.7672 (100%). Calc. for $C_{54}H_{103}N_8O_{12}$, M_r = 1055.7690; Found: C, 50.38; H, 8.53; N, 8.76. Calc. for $C_{54}H_{102}N_8O_{12}$•2HBr•3.9H_2O: C, 50.37; H, 8.75; N, 8.70%

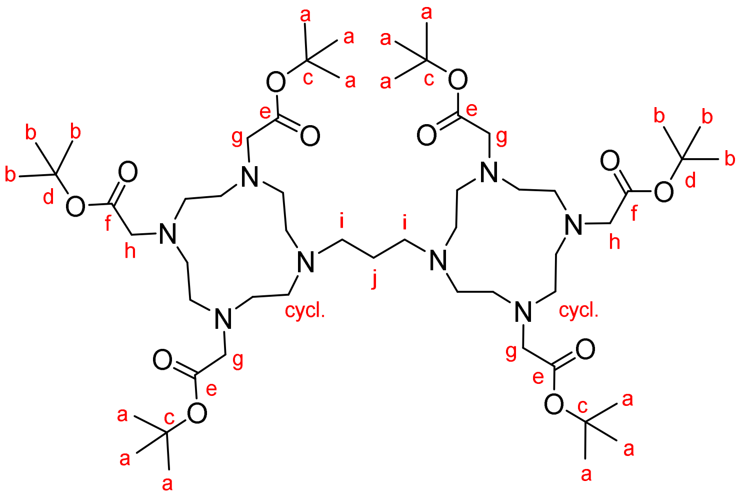

(DO3A(*t*-BuO)$_3$)$_2$C-3

(DO3A(*t*-BuO)$_3$)$_2$C-3: Prepared from **DO3A(*t*-BuO)$_3$** (3.50 g, 5.94 mmol, 2.00 equiv) and 1,3-dibromopropane (316 μL, 3.11 mmol, 1.05 equiv) following general procedure **1**. Flash column chromatography over neutral Al_2O_3 by gradient elution with CH_3CN/H_2O (100:0→99:1→98:2→97:3→96:4→95:5→93:7→90:10→80:20, *v/v*) afforded the desired product as an orange foamy solid (1.42 g, 1.32 mmol, 61%); R_f = 0.62 (neutral alumina, CH_3CN/H_2O, 95:5); mp 160–165 °C; ^{1}H NMR (500 MHz, CD_2Cl_2, 193 K) δ (ppm): 3.72 – 1.86 (52 H, br m, $H^{g,h,i,cycl.}$ + 2*H*Br), 1.74–1.18 (56 H, br m, $H^{a,b,j}$); ^{13}C NMR (126 MHz, CD_2Cl_2, 203 K) δ (ppm): 173.9 – 172.1 ($C^{e,f}$), 82.9 – 81.1 ($C^{c,d}$), 56.5 – 46.9 ($C^{g,h,i,cycl.}$), 27.3 – 27.0 ($C^{a,b}$), 19.7 (C^{j}); HRMS (ESI in CH_3OH) *m/z* (% relative intensity): $[M + H]^+$ found, 1069.7831 (100%). Calc. for $C_{55}H_{105}N_8O_{12}$, M_r = 1069.7846; Found: C, 52.34; H, 8.99; N, 8.97. Calc. for $C_{55}H_{104}N_8O_{12}$•2HBr•1.7H_2O: C, 52.35; H, 8.74; N, 8.88%

3.1.3 General procedure 2 for the synthesis of 2-[4,7-bis(carboxymethyl)-10-{2-[4,7,10-tris(carboxymethyl)-1,4,7,10-tetraazacyclododecan-1-yl]ethyl}-1,4,7,10-tetraazacyclododecan-1-yl]acetic acid ((DO3A)$_2$C-2) and 2-[4,7-bis(carboxymethyl)-10-{3-[4,7,10-tris(carboxy-methyl)-1,4,7,10-tetraazacyclododecan-1-yl]propyl}-1,4,7,10-tetraazacyclo- dodecan-1-yl] acetic acid ((DO3A)$_2$C-3)

The ligands were synthesised by adapting the procedure from the literature.[12] The appropriate bis-macrocyclic ester was dissolved in 10 mL dichloromethane and trifluoroacetic acid (10 mL) added dropwise over 1 h and stirred at 25 °C for 72 h. The reaction mixture was diluted with equal volume of methanol and concentrated to dryness. The resulting oil was dissolved in methanol (~4 mL) and triturated with diethyl ether (~160 mL) to afford a precipitate that was isolated by centrifugation. This trituration protocol was repeated twice and the isolated precipitate dried under vacuum.

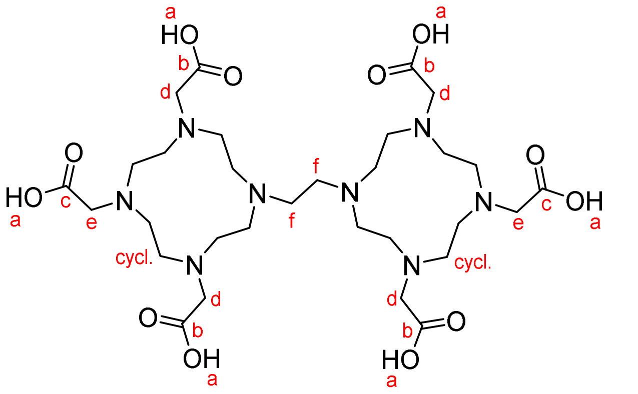

(DO3A)$_2$C-2

(DO3A)$_2$C-2: Prepared from **(DO3A(*t*-BuO)$_3$)$_2$C-2** (400 mg, 0.379 mmol) following general procedure 2. Yellow powder (240 mg, 0.33 mmol, 70%); mp 212–216 °C; ^{1}H NMR (500 MHz, DMSO-d_6, 403 K) δ (ppm): 9.32 – 7.52 (6 H, br s, –COOH^{a}), 4.33 – 2.48 (48 H, br m, $H^{d,e,f,cycl.}$); ^{13}C NMR (126 MHz, DMSO-d_6, 403 K) δ (ppm): 170.7 – 170.6 ($C^{b,c}$), 55.1 (C^{d}), 54.9 (C^{e}), 51.1 – 47.9 ($C^{f,cycl.}$); ^{19}F NMR (565 MHz, DMSO-d_6, 298 K) δ (ppm): -73.36 (CF_3COOH); HRMS (ESI in CH_3OH) *m/z* (% relative intensity): $[M – H]^-$ found, 717.3776 (100%). Calc. for $C_{30}H_{53}N_8O_{12}$, M_r = 717.3788; Found: C, 40.43; H, 6.35; N, 11.25. Calc. for $C_{30}H_{54}N_8O_{12}$•1.8CF_3COOH•4H_2O: C, 40.51; H, 6.46; N, 11.25%

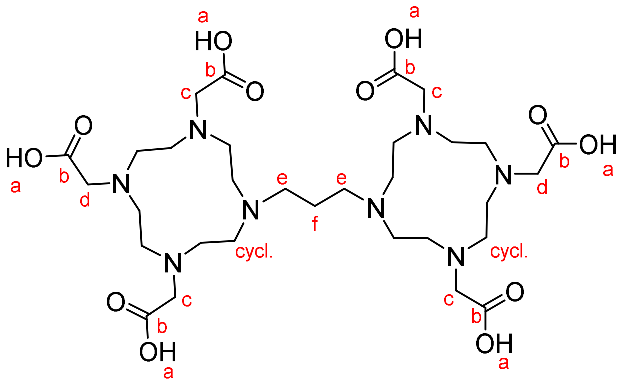

(DO3A)$_2$C-3

(DO3A)$_2$C-3: Prepared from **(DO3A(*t*-BuO)$_3$)$_2$C-3** (1.00 g, 0.935 mmol) following general procedure 2. Orange powder (yield 478 mg, 0.65 mmol, 89%); mp 214–219 °C; ^{1}H NMR (500 MHz, DMSO-d_6, 403 K) δ (ppm): 9.62 – 9.14 (6 H, br s, –COOH^a), 4.61 – 2.13 (48 H, br m, $H^{c,d,cycl.}$), 2.12 – 1.70 (2 H, br s, H^f); ^{13}C NMR (126 MHz, DMSO-d_6, 403 K) δ (ppm): 170.9 (C^b), 55.4 (C^c), 54.8 (C^d), 51.4 – 47.9 ($C^{e,cycl.}$), 19.9 (C^f); ^{19}F NMR (565 MHz, DMSO-d_6, 298 K) δ (ppm): -73.53 (CF_3COOH); HRMS (ESI in CH_3OH) *m/z* (% relative intensity): $[M – H]^-$ found, 731.3932 (100%). Calc. for $C_{31}H_{55}N_8O_{12}$, M_r = 731.3945; Found: C, 43.80; H, 6.58; N, 12.09. Calc. for $C_{31}H_{56}N_8O_{12}$•1.5CF_3COOH•1.6H_2O: C, 43.78; H, 6.56; N, 12.01%

3.2 Synthesis and characterisation of binuclear lanthanide(III) complexes

3.2.1 General procedure 3 for the synthesis of dinuclear lanthanide(III) complexes [Ln$_2$(DO3A)$_2$C-2] and [Ln$_2$(DO3A)$_2$C-3]

The complexes were synthesised by adapting the procedure from the literature.[12,13] The preformed ligands **(DO3A)$_2$C-2** or **(DO3A)$_2$C-3** (100 mg, 1.00 equiv) was added to the respective lanthanide triflate [Ln(OTf)$_3$.xH$_2$O] (2.05 equiv), methanol (2 mL) added, and the mixture sonicated until complete dissolution. The resulting solution was stirred under reflux for 1 h, aqueous NaOH (1 M) added dropwise to maintain the pH 5 - 6 and refluxed for 48 h. The solvent was evaporated under reduced pressure to afford a crude residue, dissolved in 10 mL of water and basified to pH 10 using 1 M aqueous NaOH. The precipitate was removed by centrifugation and the resulting solution neutralised using 1 M HCl, an equal volume of ethanol added, and the solvent was evaporated to dryness. The crude product was redissolved in 3 mL of deionised water and purified by dialysis (5 mL dialysis tube, molecular weight cut-off: 500 – 1000 Da). After 48 h and 6 water changes (4 L), the solvent was removed under reduced pressure to afford the desired complex.

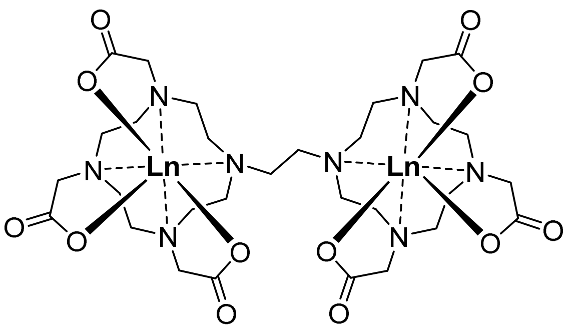

[Ln_2(**DO3A**)$_2$**C-2**] Ln = Eu(III), Tb(III) and Yb(III)]

[Eu_2(DO3A)$_2$C-2]: Prepared from (DO3A)$_2$C-2 and [Eu(OTf)$_3$.$x$$H_2O$] by following general procedure **3**. Yellow hygroscopic crystalline powder (70 mg, 0.067 mmol, 48%); ^{1}H NMR (400 MHz, D_2O, 298 K) δ (ppm): 30.54, 25.62, 22.29, 19.36, 17.95, 14.20, 11.38, -0.44, -1.96, -2.71, -4.99, -6.76, -8.46, -10.26, -12.71, -14.14, -17.76, -18.23, -19.29, -20.28, -21.61 (only resolved peaks outside 0 – 10 ppm reported); ^{19}F NMR (377 MHz, D_2O, 298 K) δ (ppm): -75.58 (CF_3COOH), -78.82 (C$F_3$$SO_3$Na); HRMS (MALDI in H_2O) *m/z*: $[M + Na]^+$ found, 1039.170. Calc. for $Eu_2C_{30}H_{48}N_8O_{12}Na$, M_r = 1039.170; Found: C, 31.44; H, 4.99; N, 9.36. Calc. for $Eu_2C_{30}H_{48}N_8O_{12}$•0.7$CF_3COOH$•0.7$CF_3SO_3Na$•0.5$H_2O$: C, 31.45; H, 4.09; N, 9.14%

[Tb_2(DO3A)$_2$C-2]: Prepared from (DO3A)$_2$C-2 and [Tb(OTf)$_3$.$x$$H_2O$] by following general procedure **3**. Brown hygroscopic crystalline powder (57 mg, 0.054 mmol, 39%); HRMS (MALDI in H_2O) *m/z*: $[M + Na]^+$ found, 1053.035. Calc. for $Tb_2C_{30}H_{48}N_8O_{12}Na$, M_r = 1053.179; Found: C, 24.32; H, 4.40; N, 7.18. Calc. for $Tb_2C_{30}H_{48}N_8O_{12}$•0.8$CF_3COOH$• 1.5$CF_3SO_3Na$•10$H_2O$: C, 25.48; H, 4.45; N, 7.18%

[Yb_2(DO3A)$_2$C-2]: Prepared from (DO3A)$_2$C-2 and [Yb(OTf)$_3$.$x$$H_2O$] by following general procedure **3**. Yellow hygroscopic crystalline powder (60 mg, 0.059 mmol, 42%); ^{1}H NMR (500 MHz, D_2O, 298 K) δ (ppm): 167.38, 154.00, 143.25, 142.39, 132.48, 122.45, 113.25, 101.15, 96.98, 92.79, 81.60, 51.11, 47.50, 46.52, 42.06, 33.38, 29.38, 28.34, 24.92, 24.34, 24.33, 23.60, 23.43, 21.44, 19.40, 17.54, 13.73, 10.86, -11.44, -12.74, -15.12, -16.65, -24.27, -27.07, -30.72, -31.85, -32.97, -37.24, -37.77, -41.24, -44.58, -48.18, -59.36, -61.34, -68.19, -71.89, -77.12, -81.23, -88.69, -89.83, -96.59, -105.96 (only the resolved peaks outside -10 – 10 ppm reported); ^{19}F NMR (470 MHz, D_2O, 298 K) δ (ppm): -75.52 (CF_3COOH), -78.2 (C$F_3$$SO_3$Na).

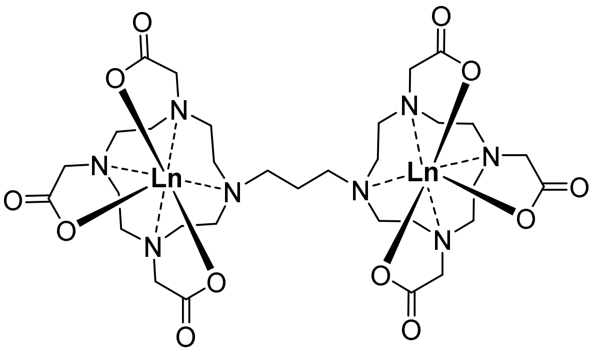

[Ln_2(**DO3A**)$_2$**C-3**] Ln = Eu(III), Tb(III) and Yb(III)]

[Eu_2(DO3A)$_2$C-3]: Prepared from (DO3A)$_2$C-3 and [Eu(OTf)$_3$.$x$$H_2O$] by following general procedure **3**. Orange hygroscopic crystalline powder (60 mg, 0.057 mmol, 42%); ^{1}H NMR (500 MHz, D_2O, 298 K) δ (ppm): 33.59, 31.66, 23.45, 21.01, 20.02, 18.93, 17.11, 16.24, 14.52, 12.88, 11.79, 10.99, 0.16, -1.07, -1.43, -1.76, -4.11, -5.35, -5.68, -6.65, -7.12, -7.90, -8.95, -9.64, -10.67, -11.48, -12.21, -13.73, -14.46, -15.86, -16.93, -17.78, -19.14, -20.22, -20.81, -21.93, -23.24, -24.40, -25.77, -27.18 (only the resolved peaks outside 0 – 10 ppm reported); ^{19}F NMR ((377 MHz, 298 K, D_2O) δ (ppm): -78.48 (C$F_3$$SO_3$Na); HRMS (MALDI in H_2O) *m/z*: [M + Na]$^+$ found, 1053.029. Calc. for $Eu_2C_{31}H_{50}N_8O_{12}Na$, M_r = 1053.185; Found: C, 31.24; H, 4.88; N, 9.08. Calc. for $Eu_2C_{31}H_{50}N_8O_{12}$•1.1$CF_3SO_3Na$•1.5$H_2O$: C, 31.25; H, 4.34; N, 9.11%

[Tb_2(DO3A)$_2$C-3]: Prepared from (DO3A)$_2$C-3 and [Tb(OTf)$_3$.$x$$H_2O$] by following general procedure **3**. Orange hygroscopic crystalline powder (yield 45 mg, 0.043 mmol, 31%); HRMS (ESI in CH_3OH) *m/z* (% relative intensity): [M + H]$^+$ found, 1045.2130 (100%). Calc. for $Tb_2C_{31}H_{51}N_8O_{12}$, M_r = 1045.2128; Found: C, 27.91; H, 5.03; N, 8.15. Calc. for $Tb_2C_{31}H_{50}N_8O_{12}$ •1.6CF_3SO_3Na•3H_2O: C, 28.50; H, 4.11; N, 8.16%

[Yb_2(DO3A)$_2$C-3]: Prepared from (DO3A)$_2$C-3 and [Yb(OTf)$_3$.$x$$H_2O$] by following general procedure **3**. Yellow hygroscopic crystalline powder (50 mg, 0.046 mmol, 33%); ^{1}H NMR (500 MHz, D_2O, 298 K) δ (ppm): 125.83, 109.61, 34.41, 27.48, 13.35, -2.69, -14.15, -55.78, -70.33, -85.56, -94.16 (only resolved peaks outside -10 – 10 ppm reported); ^{19}F NMR (377 MHz, D_2O, 298 K) δ (ppm): -75.84 (CF_3COOH), -78.75 (C$F_3$$SO_3$Na); HRMS (MALDI in H_2O) *m/z*: [M + Na]$^+$ found, 1095.124. Calc. for $Yb_2C_{31}H_{50}N_8O_{12}Na$, M_r = 1095.220; Found: C, 32.93; H, 4.99; N, 9.39. Calc. for $Yb_2C_{31}H_{50}N_8O_{12}$•0.3$CF_3COOH$•0.2$CF_3SO_3Na$•$H_2O$: C, 32.94; H, 4.55; N, 9.66%

3.3 Synthesis and characterisation of pDO3A and complexes [Ln(pDO3A)]

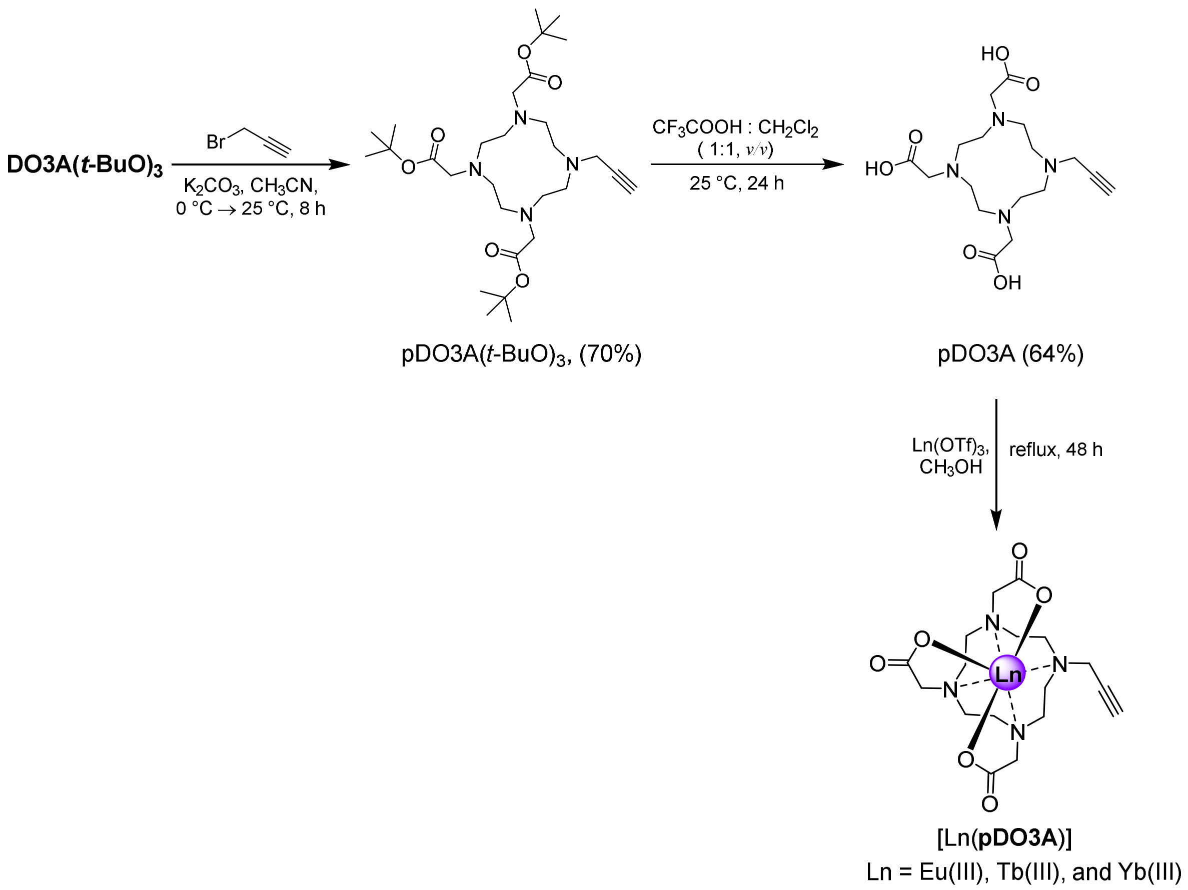

Figure S1: Synthesis of mononuclear lanthanide complexes [Ln(**pDO3A**)].[14]

3.3.1 Synthesis of *tert*-butyl 2-{4,7-bis[2-(*tert*-butoxy)-2-oxoethyl]-10-(prop-2-yn-1-yl)-1,4,7,10-tetraazacyclododecan-1-yl}acetate (pDO3A(*t*-BuO)$_3$)

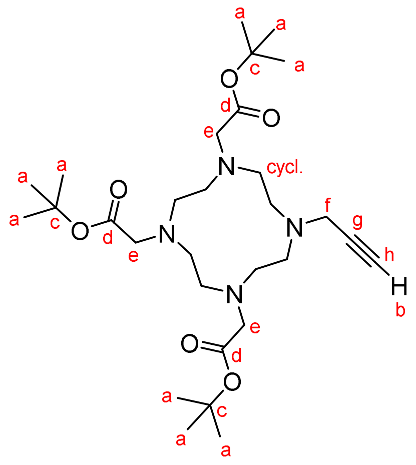

pDO3A(*t*-BuO)$_3$

This compound was synthesised by following the literature procedure.[14] (yield 70%); ^{1}H NMR (500 MHz, $CDCl_3$, 298 K) δ (ppm): 3.42 (2 H, s, H^f), 3.27 (6 H, s, H^e), 2.82 (12 H, s, $H^{cycl.}$), 2.68 (4 H, m, $H^{cycl.}$), 2.14 (1 H, s, H^b), 1.45 (27 H, s, H^a); ^{13}C NMR (126 MHz, D_2O,

298 K) δ (ppm): 171.3 (C^{d}), 80.9 (C^{c}), 79.4 (C^{g}) 72.6 (C^{h}), 56.9 (C^{e}), 52.3 – 51.2 ($C^{cycl.}$), 43.2 (C^{f}), 28.4 (C^{a}); HRMS (ESI in CH_3OH) *m/z* (% relative intensity): $[M + H]^+$ found, 553.3944 (100%). Calc. for $C_{29}H_{53}N_4O_6$, M_r = 553.3960.

3.3.2 Synthesis of 2-[4,7-bis(carboxymethyl)-10-(prop-2-yn-1-yl)-1,4,7,10-tetraazacyclo-dodecan-1-yl]acetic acid (pDO3A)

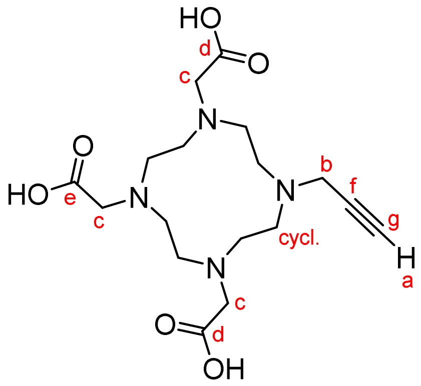

pDO3A

This compound was synthesised by following the literature procedure.[14] Trituration was repeated thrice to afford the title compound as a white solid as reported.[14] (yield 64%); mp 173–178 °C; ^{1}H NMR (500 MHz, D_2O, 298 K) δ (ppm): 3.94 – 3.71 (6 H, m, H^{c}), 3.62 (2 H, s, H^{b}), 3.51 – 3.25 (8 H, m, $H^{cycl.}$), 3.25 – 3.05 (8 H, m, $H^{cycl.}$), 2.78 (1 H, br s, H^{a}); ^{13}C NMR (126 MHz, D_2O, 298 K) δ (ppm): 173.1 (C^{d}), 171.0 (C^{e}), 76.4 (C^{f}) 72.7 (C^{g}), 55.5 (C^{c}), 53.8 – 48.0 (C^{cycl}), 43.0 (C^{b}); ^{19}F NMR (565 MHz, D_2O, 298 K) δ (ppm): -73.24 (CF_3COOH); HRMS (ESI in CH_3OH) *m/z* (% relative intensity): $[M + H]^+$ found, 385.2080 (100%). Calc. for $C_{17}H_{29}N_4O_6$, M_r = 385.2082.

3.3.3 General procedure 4 for the synthesis of mononuclear lanthanide(III) complexes [Ln(pDO3A)] and [Tb(DOTA)]$^-$

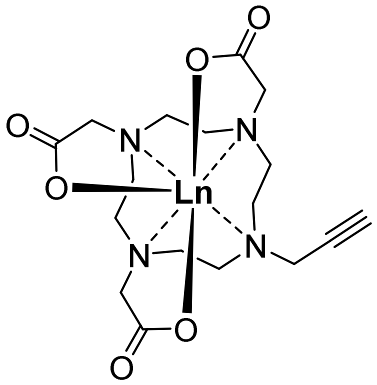

[Ln(**pDO3A**)] Ln = Eu(III), Tb(III), Yb(III)

These complexes were synthesised by following the literature procedure.[13,14] The ligand **pDO3A** (200 mg, 0.4 mmol, 1.00 equiv) was added to the respective lanthanide triflate [Ln(OTf)$_3$.xH$_2$O] (1.05 equiv), methanol (2 mL) added, and the mixture sonicated until complete dissolution. The resulting solution was stirred under reflux for 1 h, before aqueous NaOH (1 M) was added dropwise to pH 5 - 6 and heated under reflux for 48 h. The solvent was evaporated under reduced pressure to afford a crude residue, dissolved in 10 mL of water and basified to pH 10 using 1 M aqueous NaOH. The precipitate was removed by centrifugation and the resulting solution neutralised using 1 M HCl, an equal volume of ethanol added, and the solvent flash evaporated to dryness. The crude product was redissolved in 3 mL of deionised water and purified by dialysis (5 mL dialysis tube, molecular weight cut-off: 100 – 500 Da). After 48 h and 6 water changes (4 L), the solvent was removed under reduced pressure to afford the complex.

[Eu(pDO3A)]: Prepared from [Eu(OTf)$_3$.xH$_2$O] following general procedure **4**. White hygroscopic powder (yield 60%); ^{1}H NMR (400 MHz, D$_2$O, 298 K) δ (ppm): 25.37, 18.86, 17.21, 15.36, 13.55, 12.06, 10.28, 0.84, -0.39, -1.34, -1.67, -5.76, -6.81, -8.96, -10.15, -11.16, -11.97, -13.91, -15.32, -19.11, -21.20 (only resolved peaks outside 0 – 10 ppm reported); ^{19}F NMR (376 MHz, D$_2$O, 298 K) δ (ppm): -75.55 (CF_3COOH), -78.80 (CF_3SO$_3$Na); HRMS (ESI in MeOH) *m/z* (% relative intensity): [M + H]$^+$ found, 535.1058 (100%). Calc. for EuC$_{17}$H$_{26}$N$_4$O$_6$, M_r = 535.1060.

[Tb(pDO3A)]: Prepared from [Tb(OTf)$_3$.xH$_2$O] following general procedure **4**. Off-white hygroscopic powder (yield 45%); ^{1}H NMR (500 MHz, D$_2$O, 298 K) δ (ppm): 405.28, 246.19, 222.90, 184.93, 121.09, 104.87, 82.82, 72.07, -78.48, -100.44, -122.03, -133.83, -139.73, -145.27, -167.22, -179.79, -203.59, -217.77, -225.23, -244.86, -286.33, -304.43, -333.16, -365.33, -380.66, -405.41 (only the resolved peaks outside -50 – 50 ppm reported); HRMS (ESI in MeOH) *m/z* (% relative intensity): [M + H]$^+$ found, 541.1092 (100%). Calc. for TbC$_{17}$H$_{26}$N$_4$O$_6$, M_r = 541.1100.

[Yb(pDO3A)]: Prepared from [Yb(OTf)$_3$.xH$_2$O] following general procedure **4**. White hygroscopic powder (yield 57%); ^{1}H NMR (400 MHz, D$_2$O, 298 K) δ (ppm): 133.93, 126.02, 119.29, 38.68, 31.71, 25.89, 23.25, 16.22, 13.73, -17.27, -56.95, -61.09, -72.58, -75.59, -83.27, -95.34 (only the resolved peaks outside -10 to 10 ppm reported); ^{19}F NMR (376 MHz, D$_2$O, 298 K) δ (ppm): -78.28 (CF_3SO$_3$Na); HRMS (ESI in MeOH) *m/z* (% relative intensity): [M + H]$^+$ found 556.1241 (100%). Calc. for YbC$_{17}$H$_{26}$N$_4$O$_6$, M_r = 556.1235.

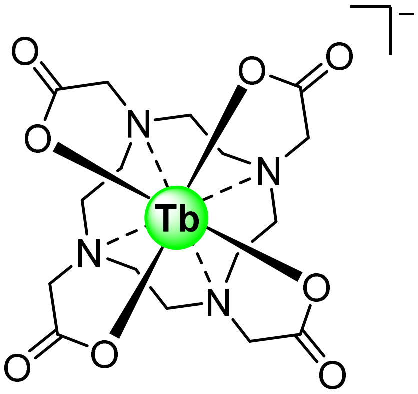

[Tb(**DOTA**)]⁻

Prepared from DOTA (50 mg, 1.00 equiv) and [Tb(OTf)$_3$.*x*H_2O] following general procedure **4.** White hygroscopic powder (yield 30%); ^{1}H NMR (400 MHz, D_2O, 298 K) δ (ppm): 259, 174.2, 136.6, 83.2, 61.7, -69.0, -94.7, -98.3, -228.6, -396.1 (only the resolved peaks outside -50 to 50 ppm reported); LRMS (ESI in MeOH) *m/z* (% relative intensity): 582.190 (100%) [M + Na] ($TbC_{16}H_{22}N_4O_6Na$).

4. Mass and NMR Spectra of Ligands and Complexes

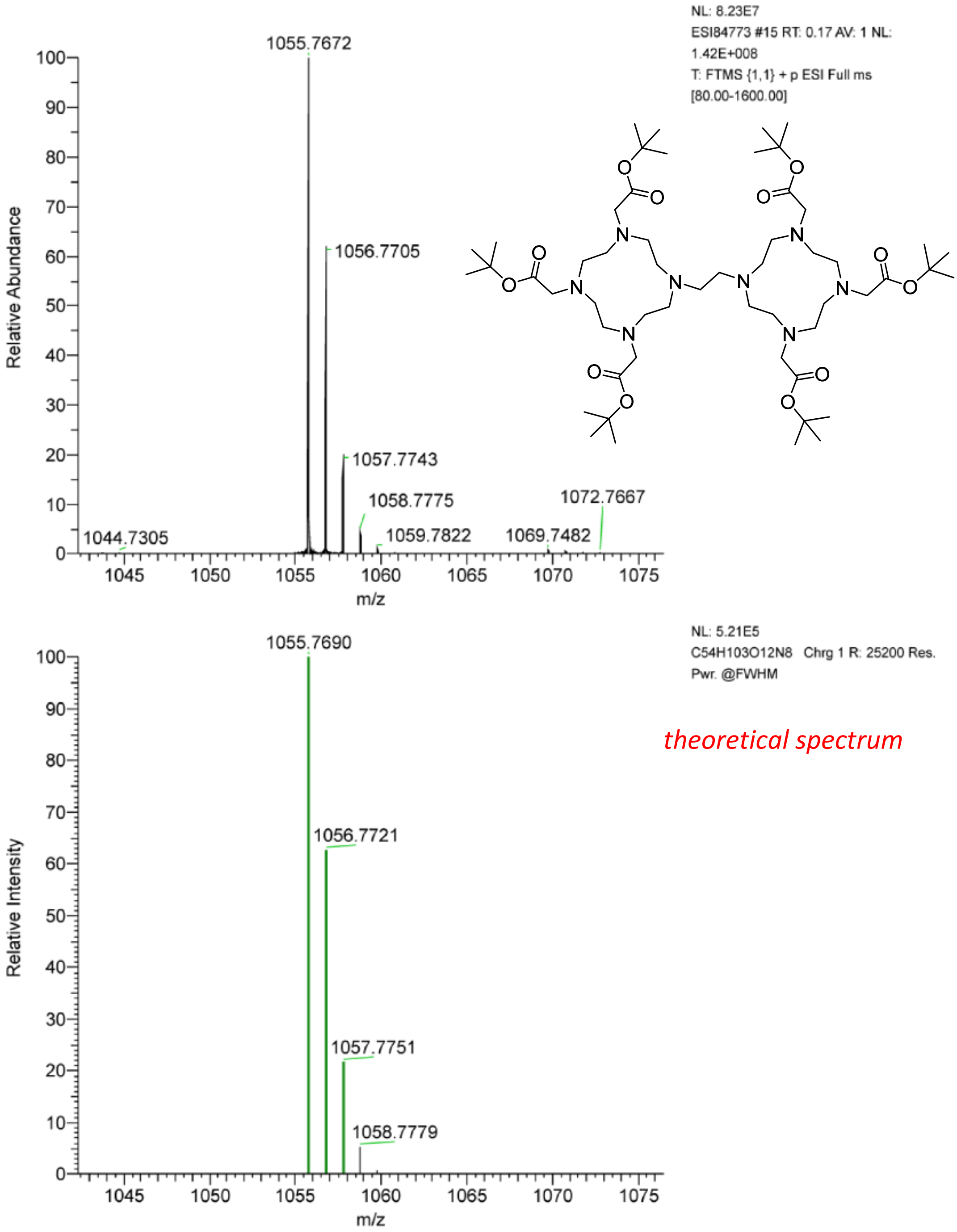

Figure S2: ESI-Mass spectrum of **(DO3A(*t*-BuO)$_3$)$_2$C-2**, experimental (*top*) and theoretical (*bottom*).

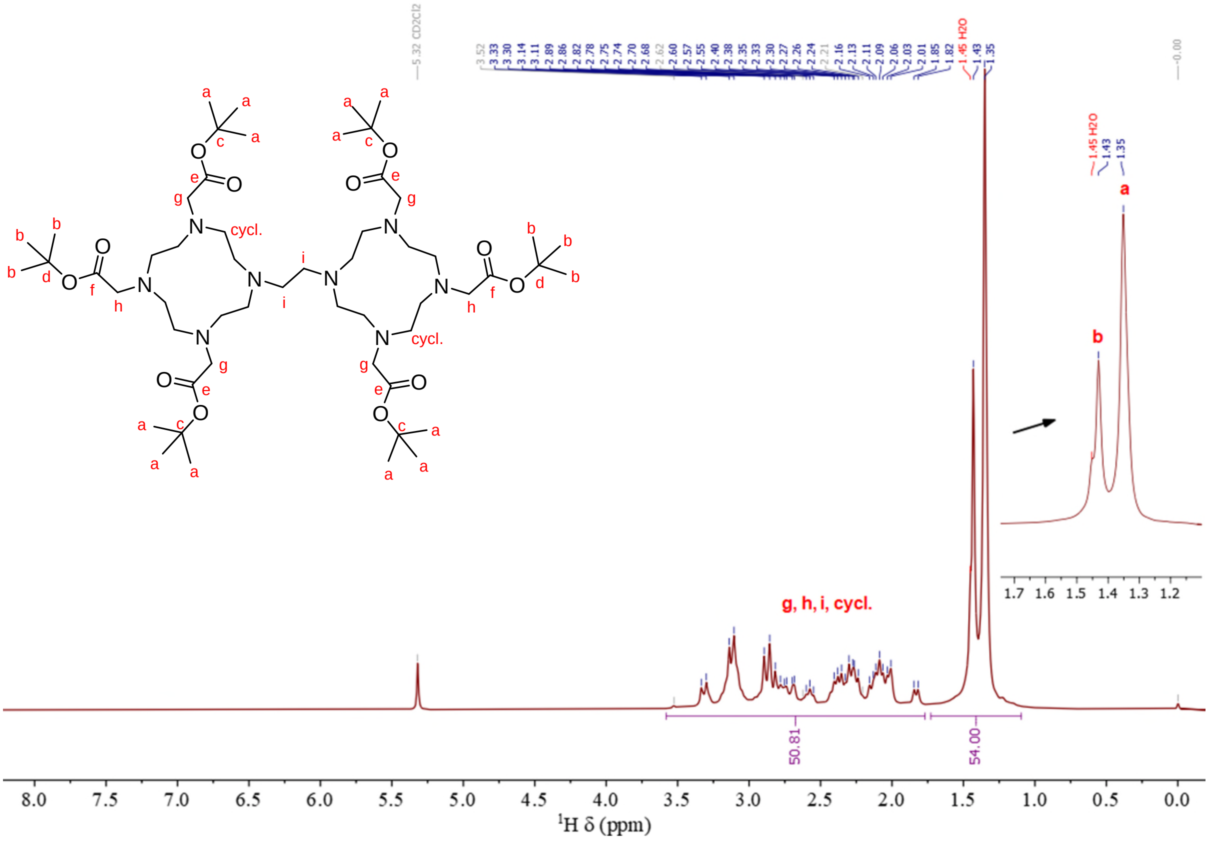

Figure S3: 500 MHz ^{1}H NMR spectrum of **(DO3A(*t*-BuO)$_3$)$_2$C-2** in CD_2Cl_2 at 203 K.

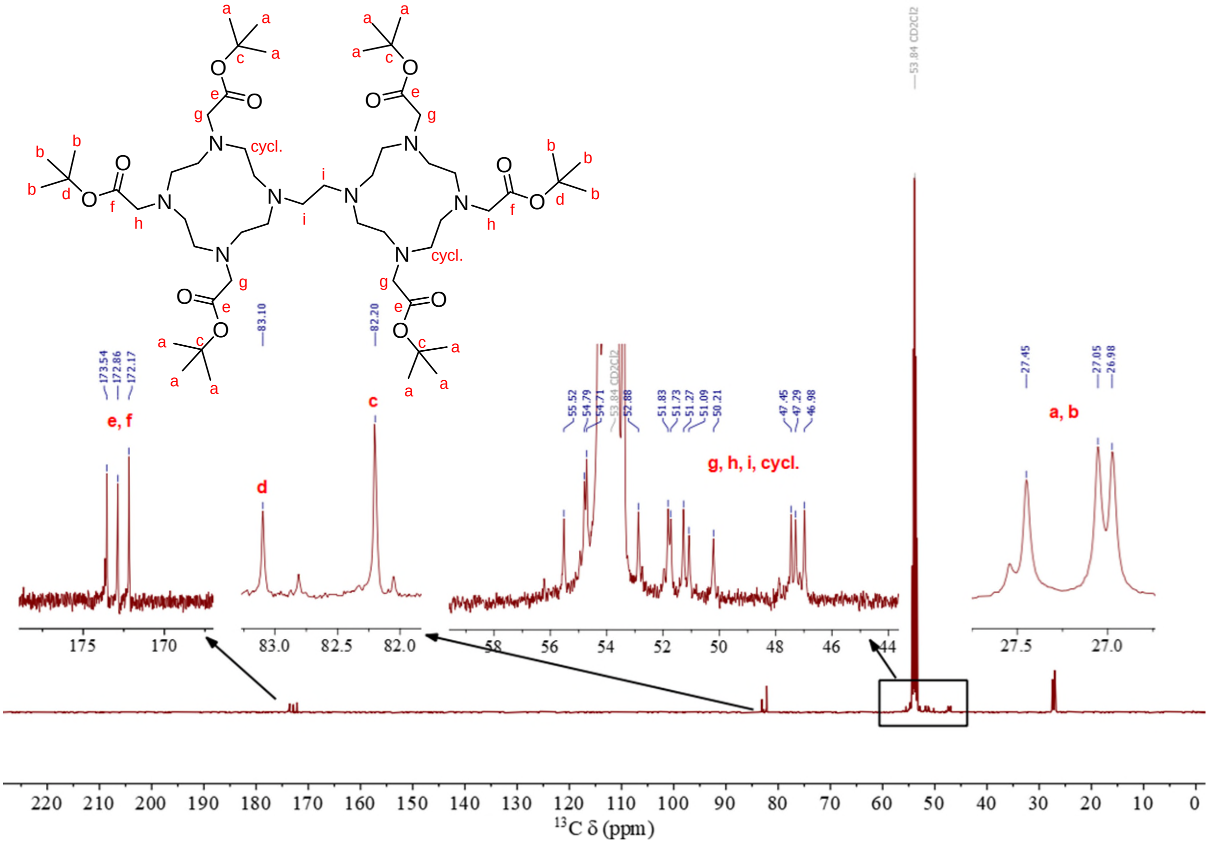

Figure S4: 126 MHz ^{13}C NMR spectrum of **(DO3A(*t*-BuO)$_3$)$_2$C-2** in CD_2Cl_2 at 203 K.

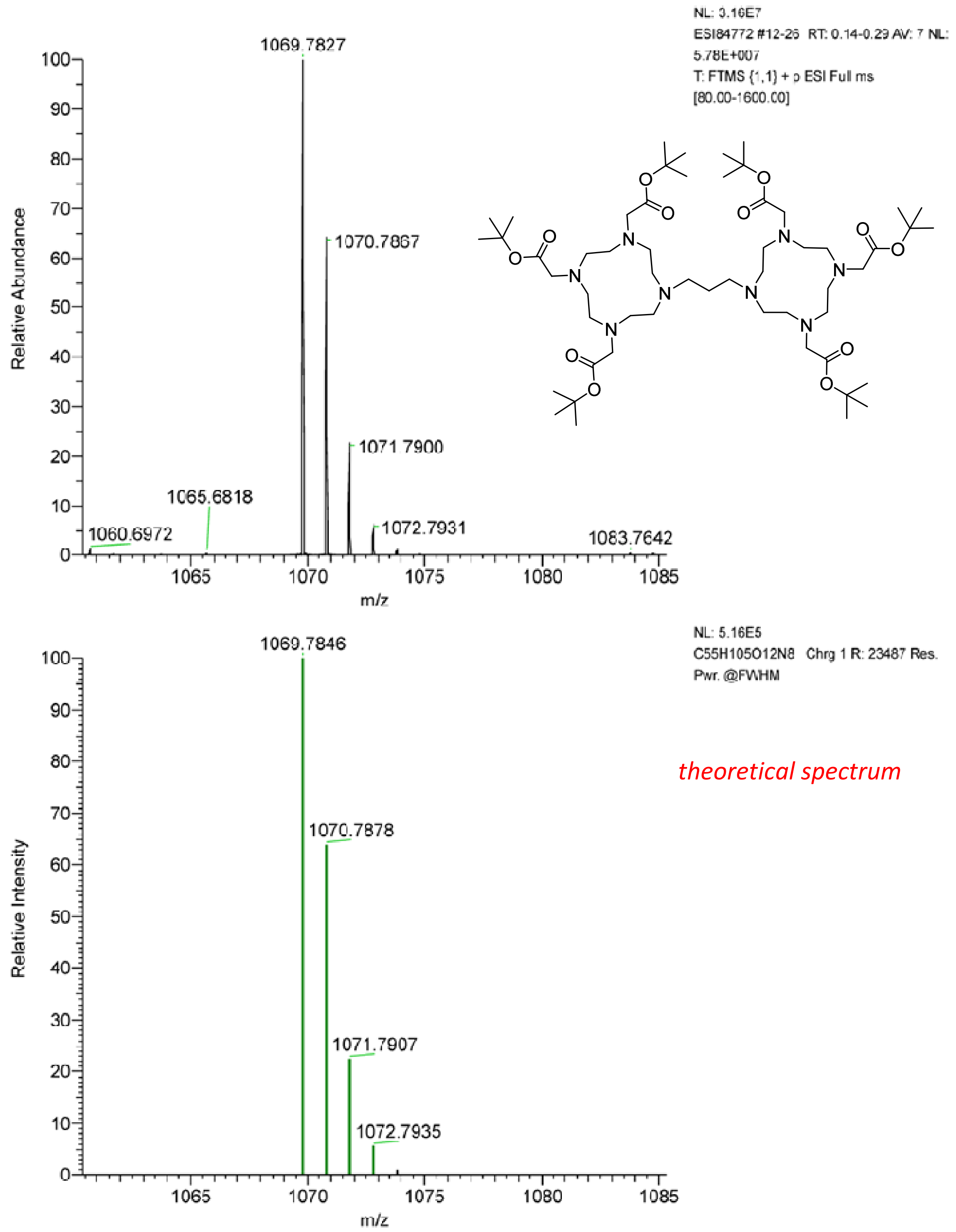

Figure S5: ESI-Mass spectrum of **(DO3A(*t*-BuO)$_3$)$_2$C-3**, experimental (*top*) and theoretical (*bottom*).

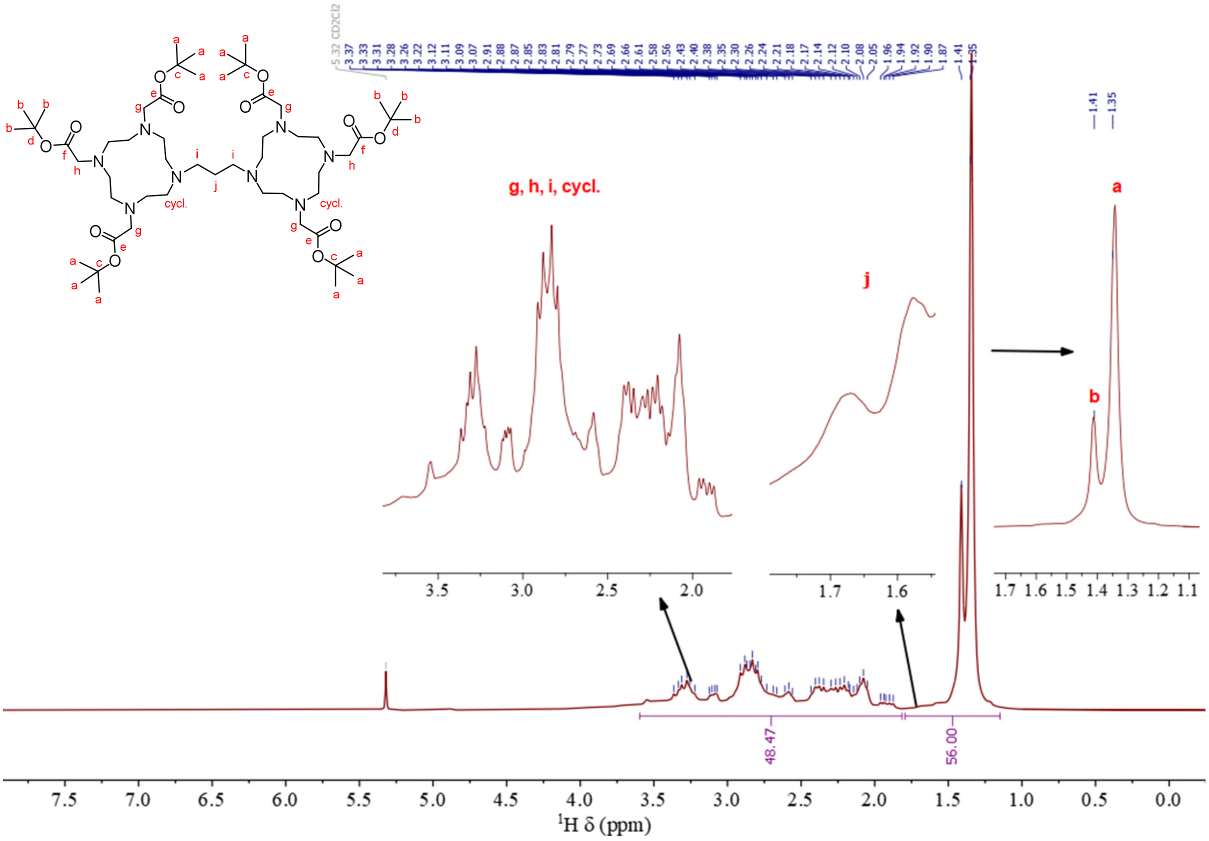

Figure S6: 500 MHz ^{1}H NMR spectrum of **(DO3A(*t*-BuO)$_3$)$_2$C-3** in CD_2Cl_2 at 193 K.

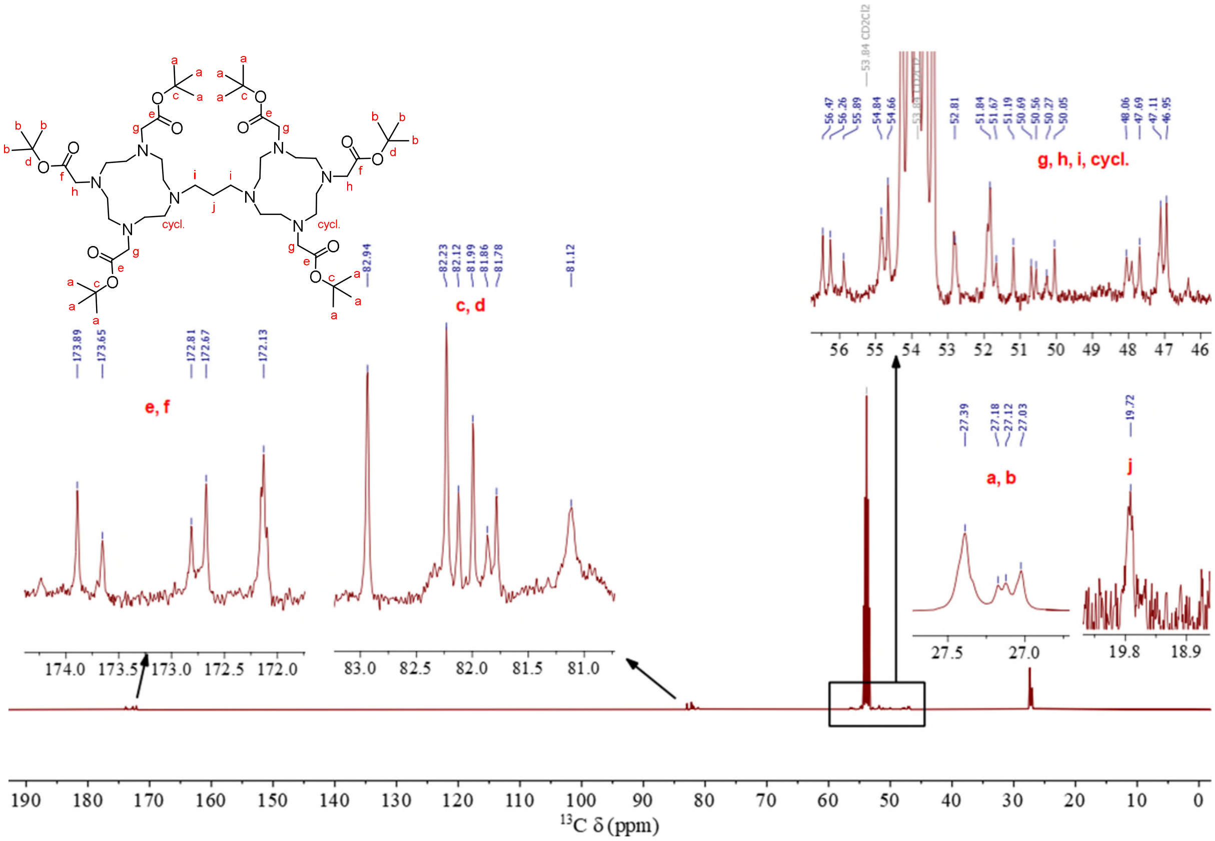

Figure S7: 126 MHz ^{13}C NMR spectrum of **(DO3A(*t*-BuO)$_3$)$_2$C-3** in CD_2Cl_2 at 203 K.

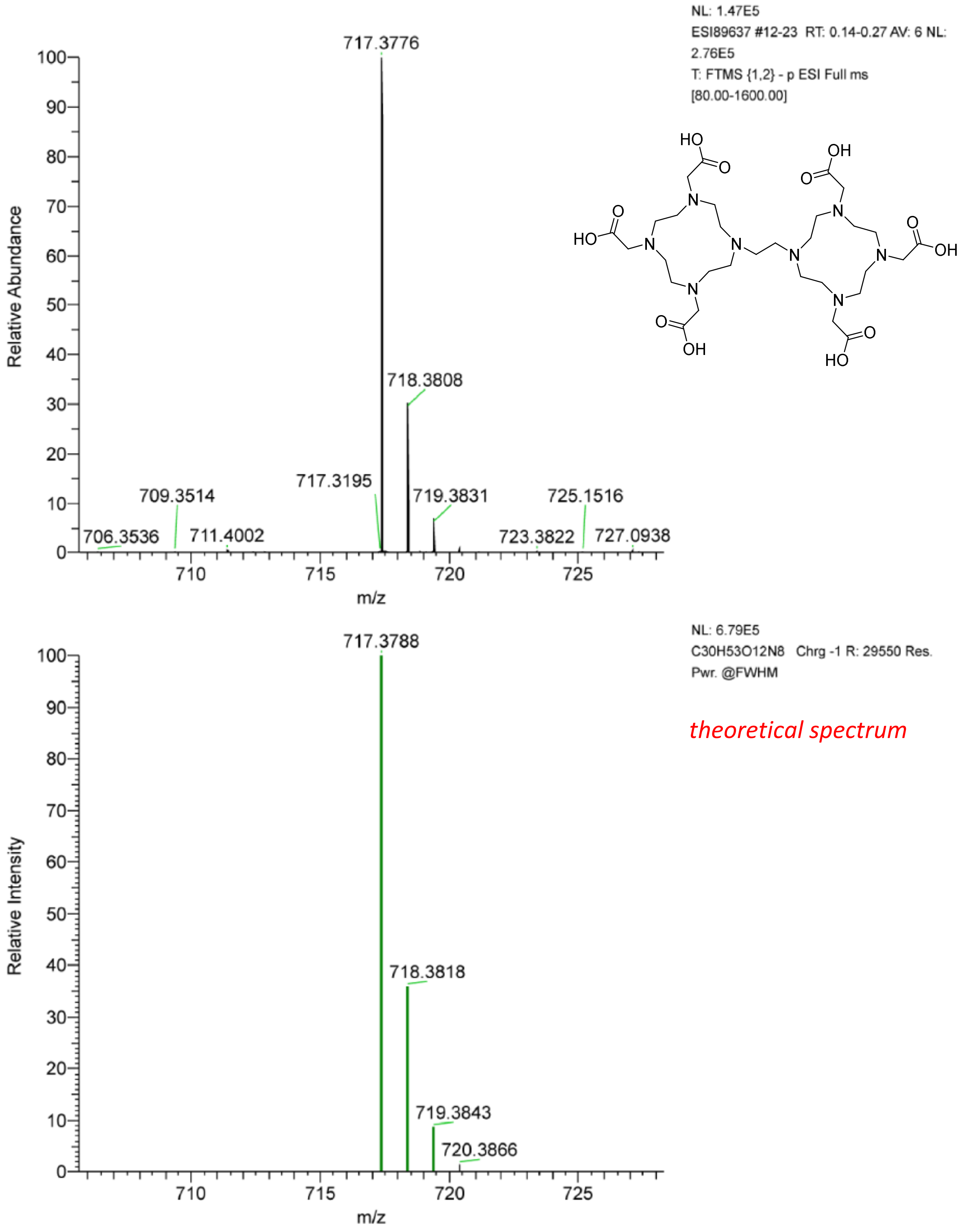

Figure S8: ESI-Mass spectrum of **(DO3A)$_2$C-2**, experimental (*top*) and theoretical (*bottom*).

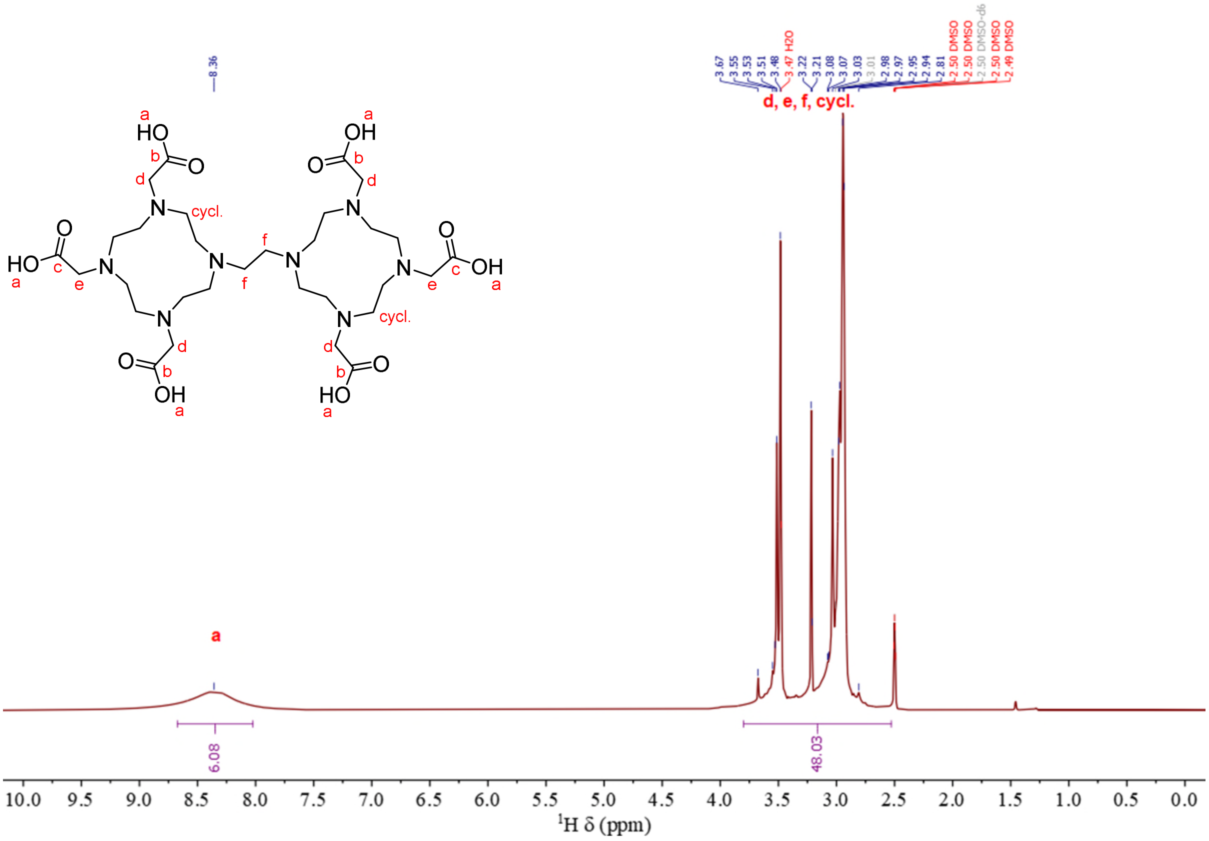

Figure S9: 500MHz ^{1}H NMR spectrum of **(DO3A)$_2$C-2** in DMSO-d_6 at 403 K.

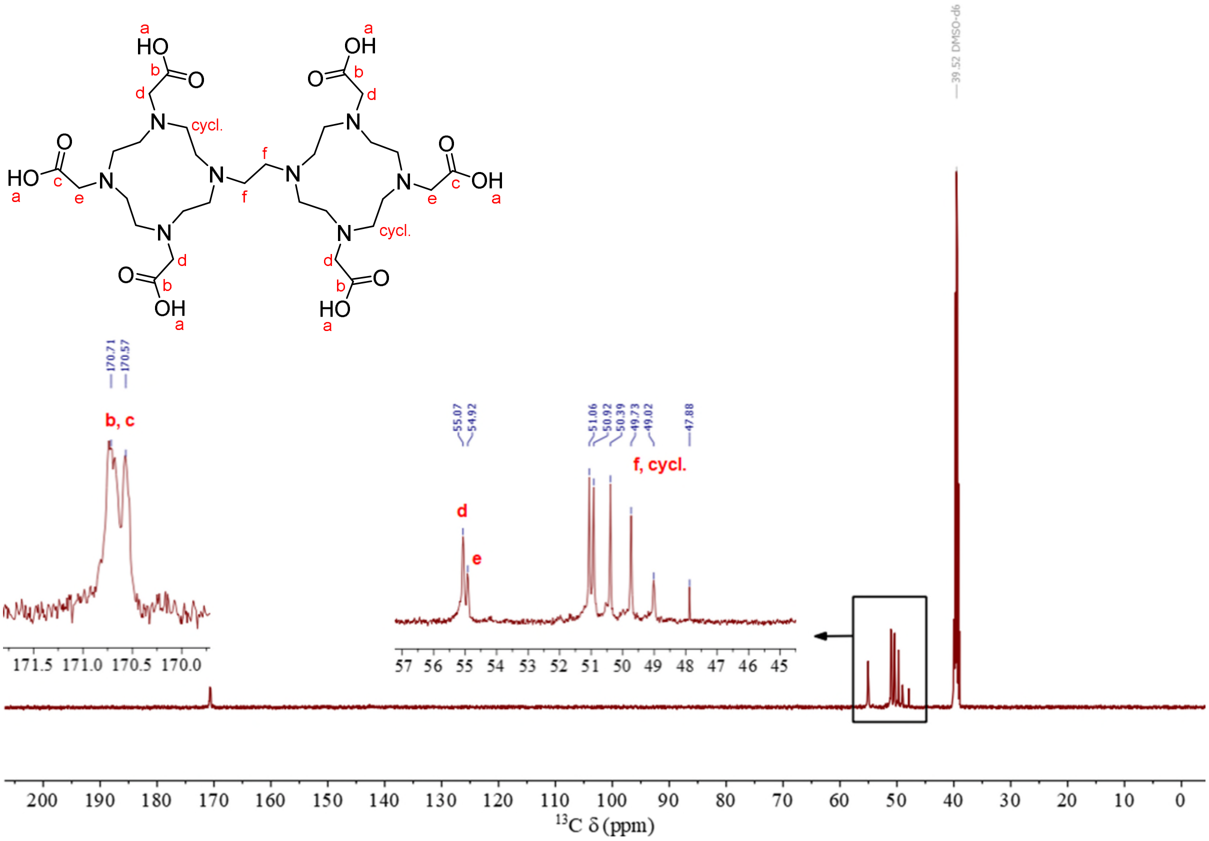

Figure S10: 126 MHz ^{13}C NMR spectrum of **(DO3A)$_2$C-2** in DMSO-d_6 at 403 K.

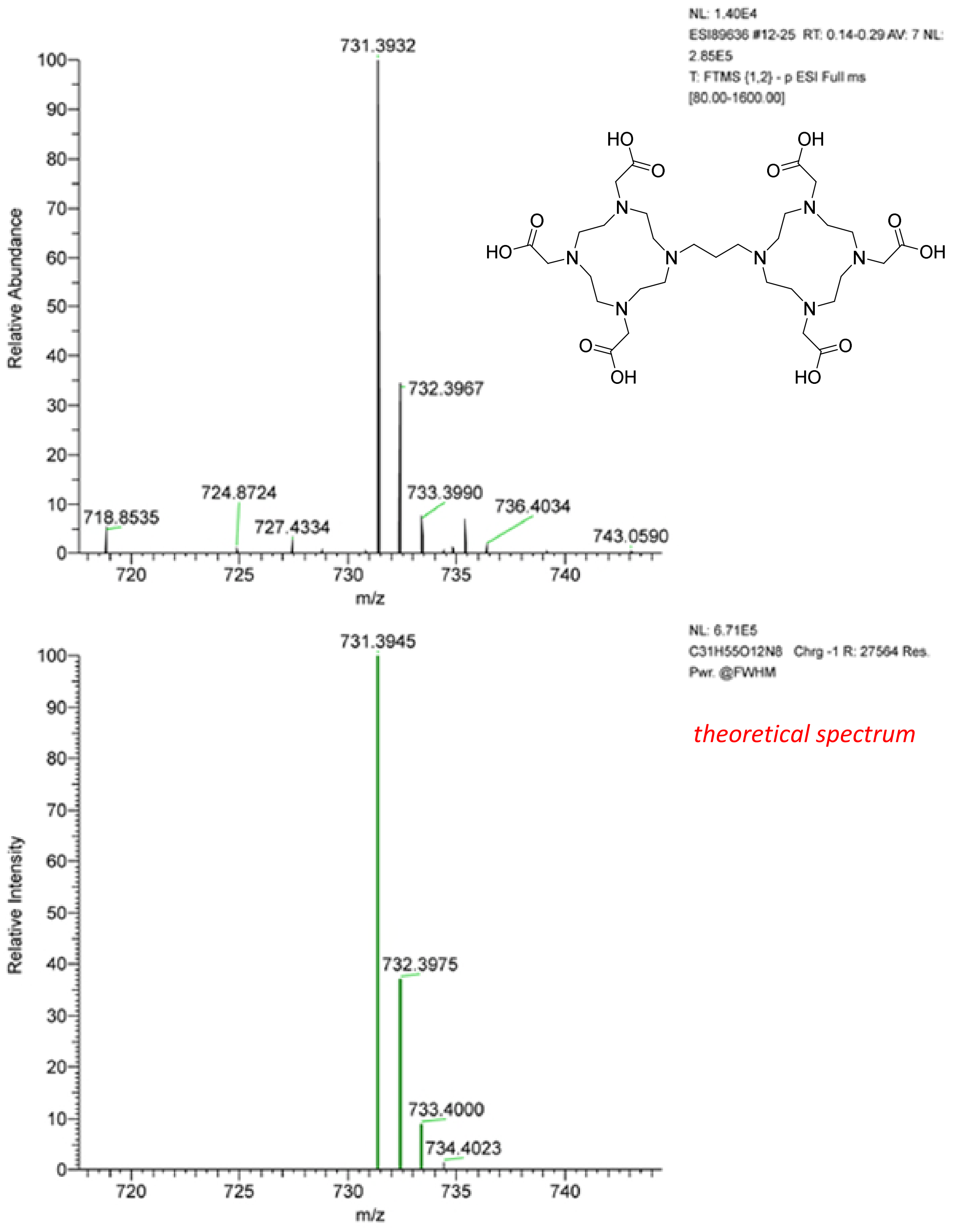

Figure S11: ESI-Mass spectrum of **(DO3A)$_2$C-3**, experimental (*top*) and theoretical (*bottom*).

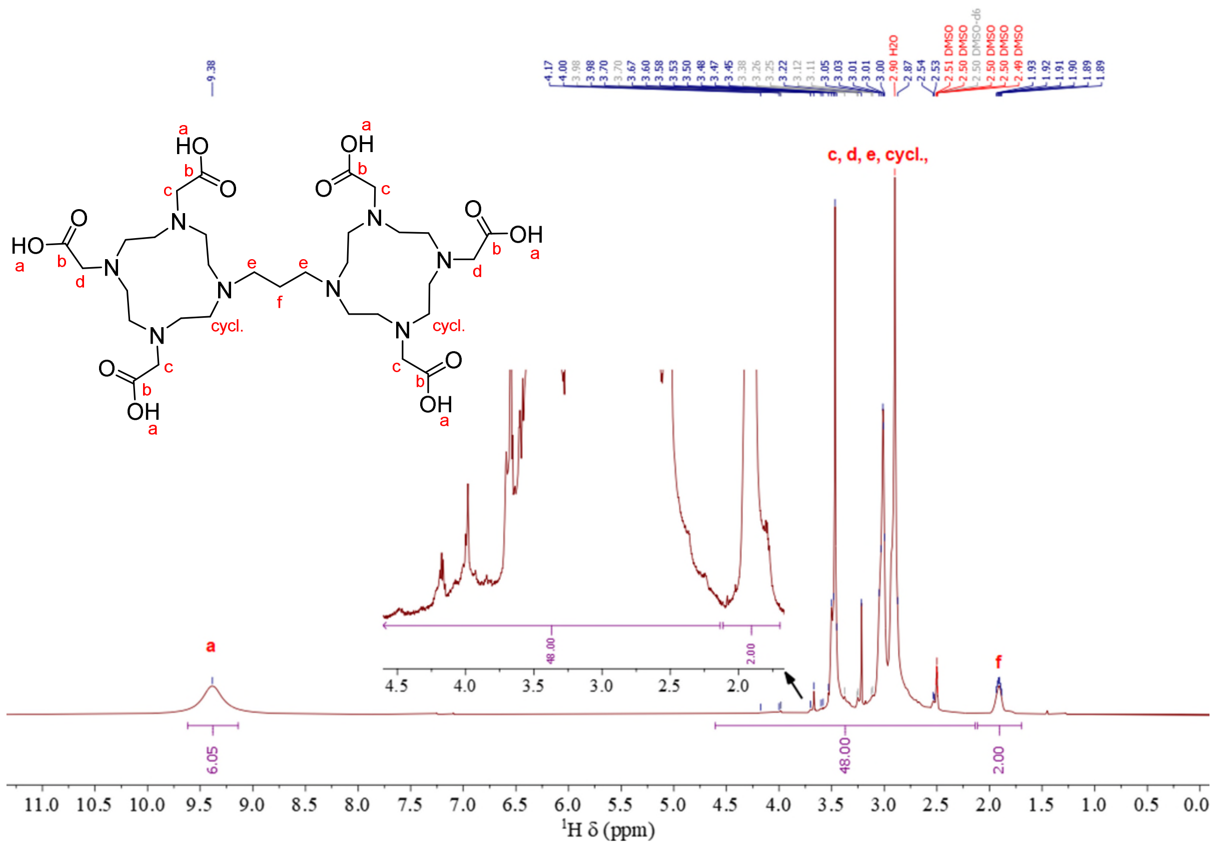

Figure S12: 500 MHz ^{1}H NMR spectrum of **(DO3A)₂C-3** in DMSO-d_6 at 403 K.

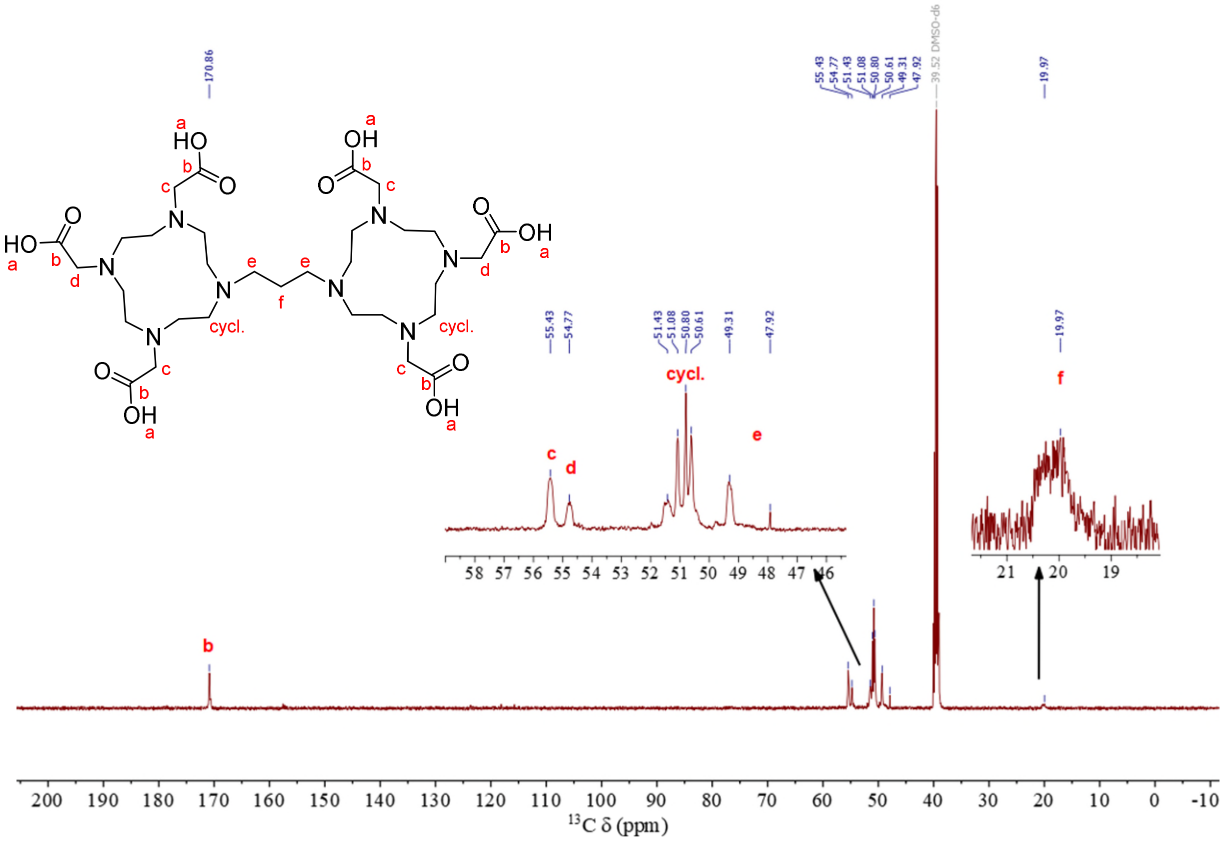

Figure S13: 126 MHz ^{13}C NMR spectrum of **(DO3A)₂C-3** in DMSO-d_6 at 403 K.

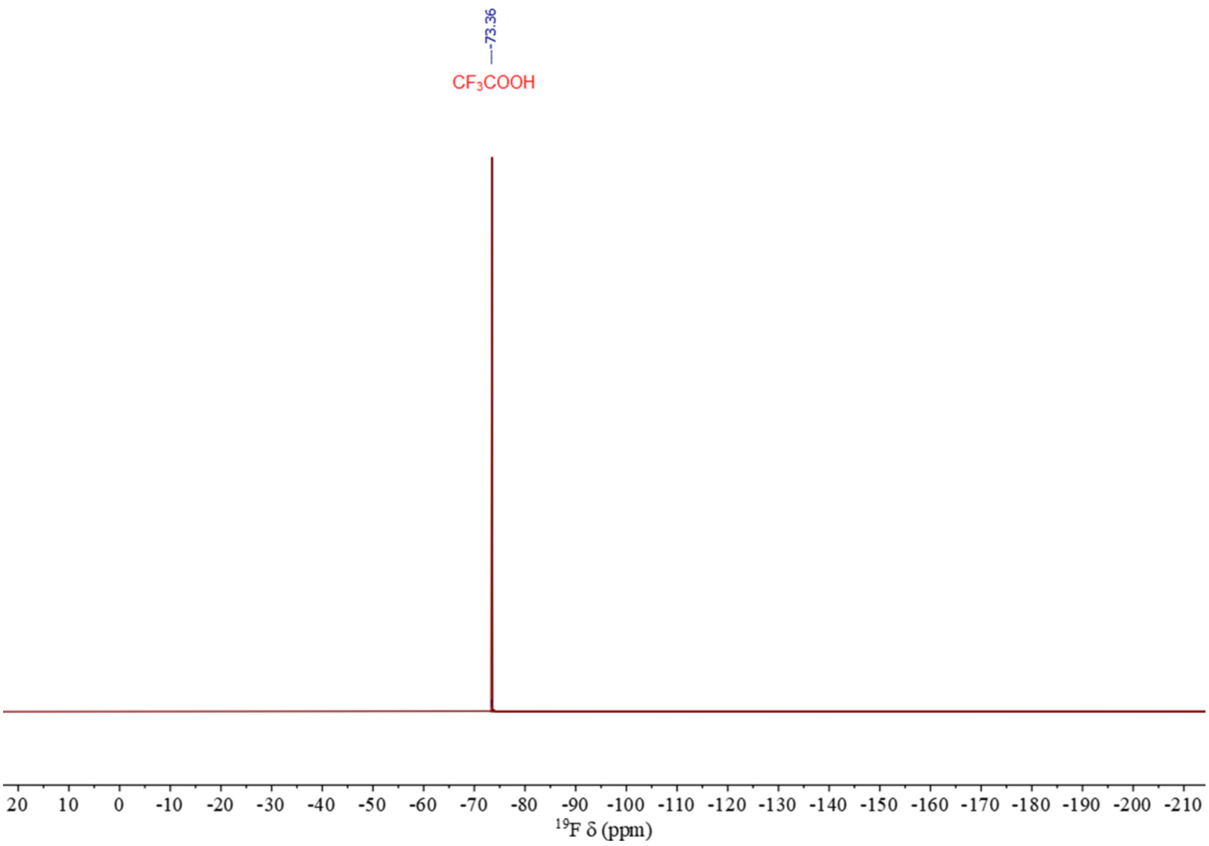

Figure S14: 565 MHz ^{19}F NMR spectrum of **(DO3A)$_2$C-2** in DMSO-d_6 at 298 K.

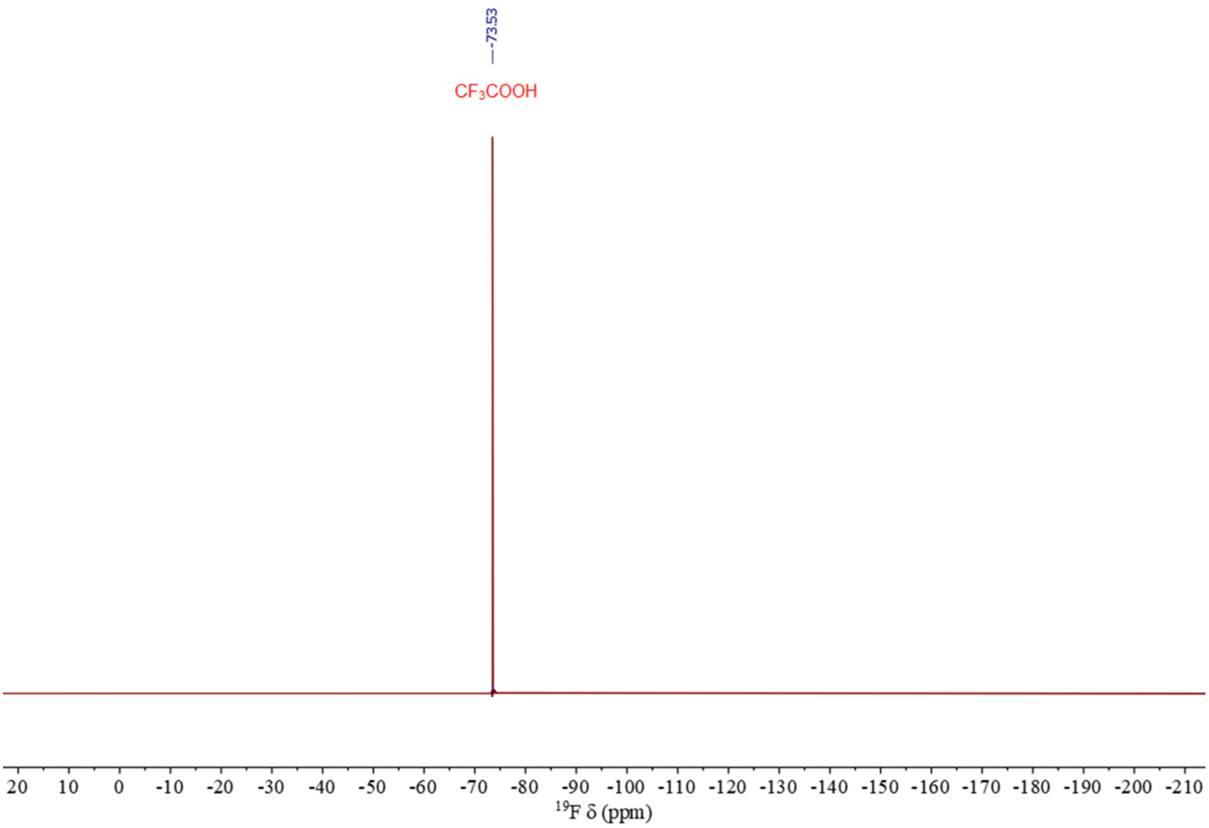

Figure S15: 565 MHz ^{19}F NMR spectrum of **(DO3A)$_2$C-3** in DMSO-d_6 at 298 K.

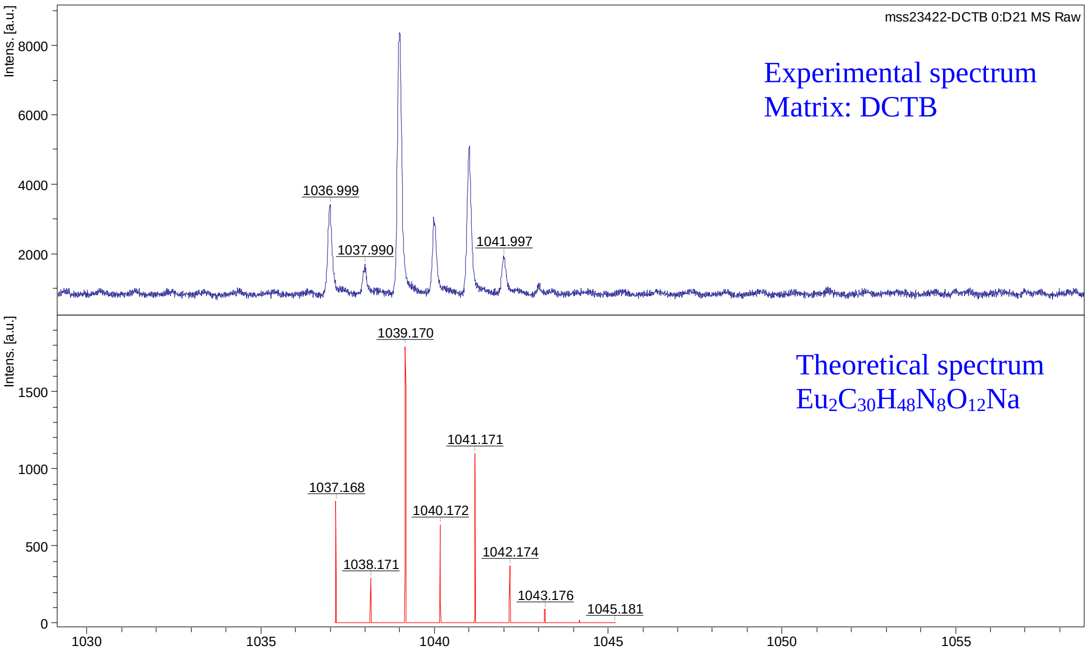

Figure S16: MALDI-TOF Mass spectrum of [Eu_2(**DO3A**)$_2$**C-2**], experimental (*top*) and theoretical (*bottom*).

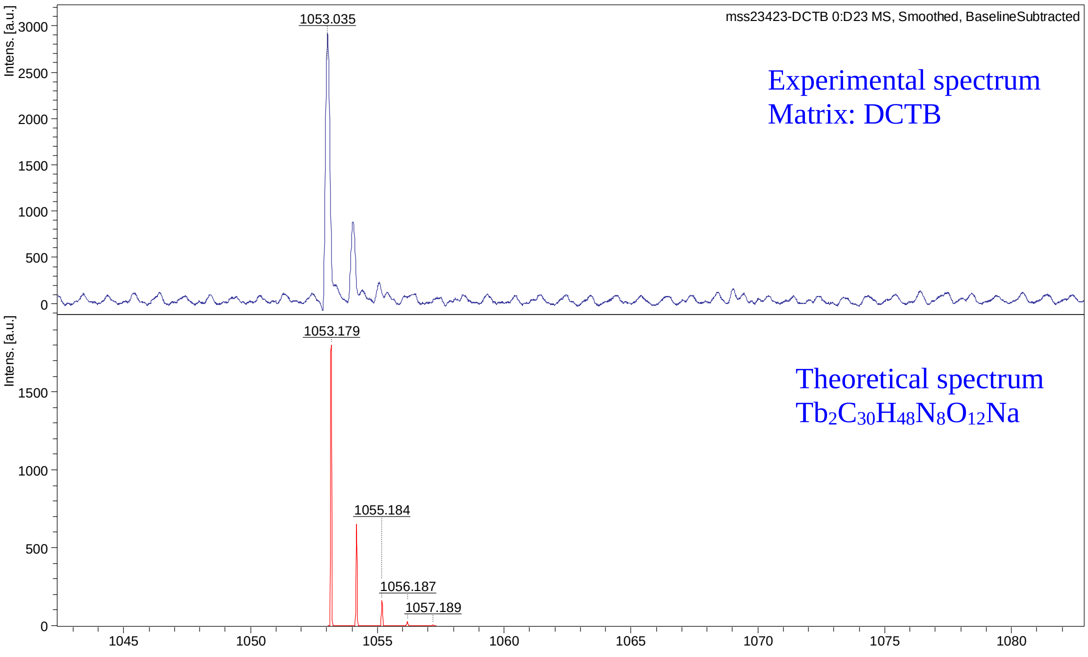

Figure S17: MALDI-TOF Mass spectrum of [Tb_2(**DO3A**)$_2$**C-2]**, experimental (*top*) and theoretical (*bottom*).

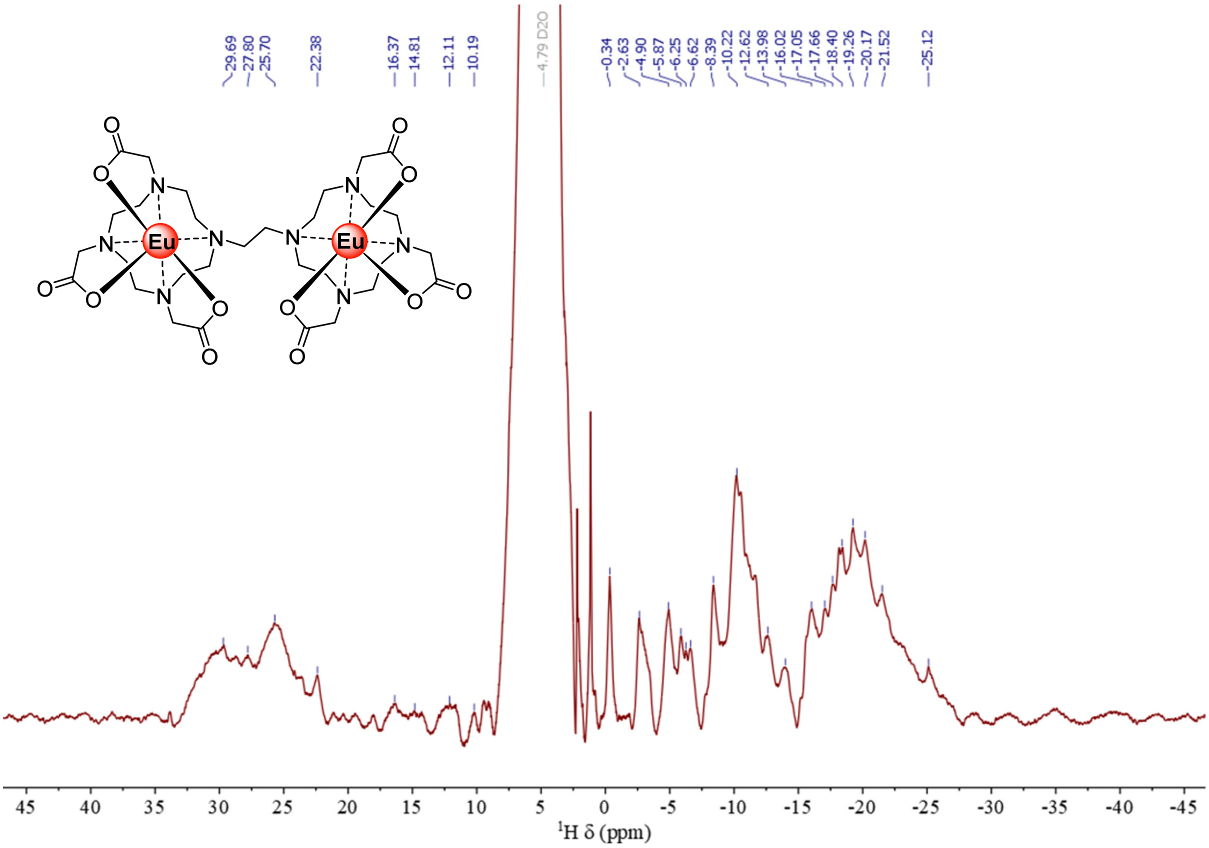

Figure S18: 400 MHz ^{1}H NMR spectrum of [Eu_2(**DO3A**)$_2$**C-2**] in D_2O at 298 K.

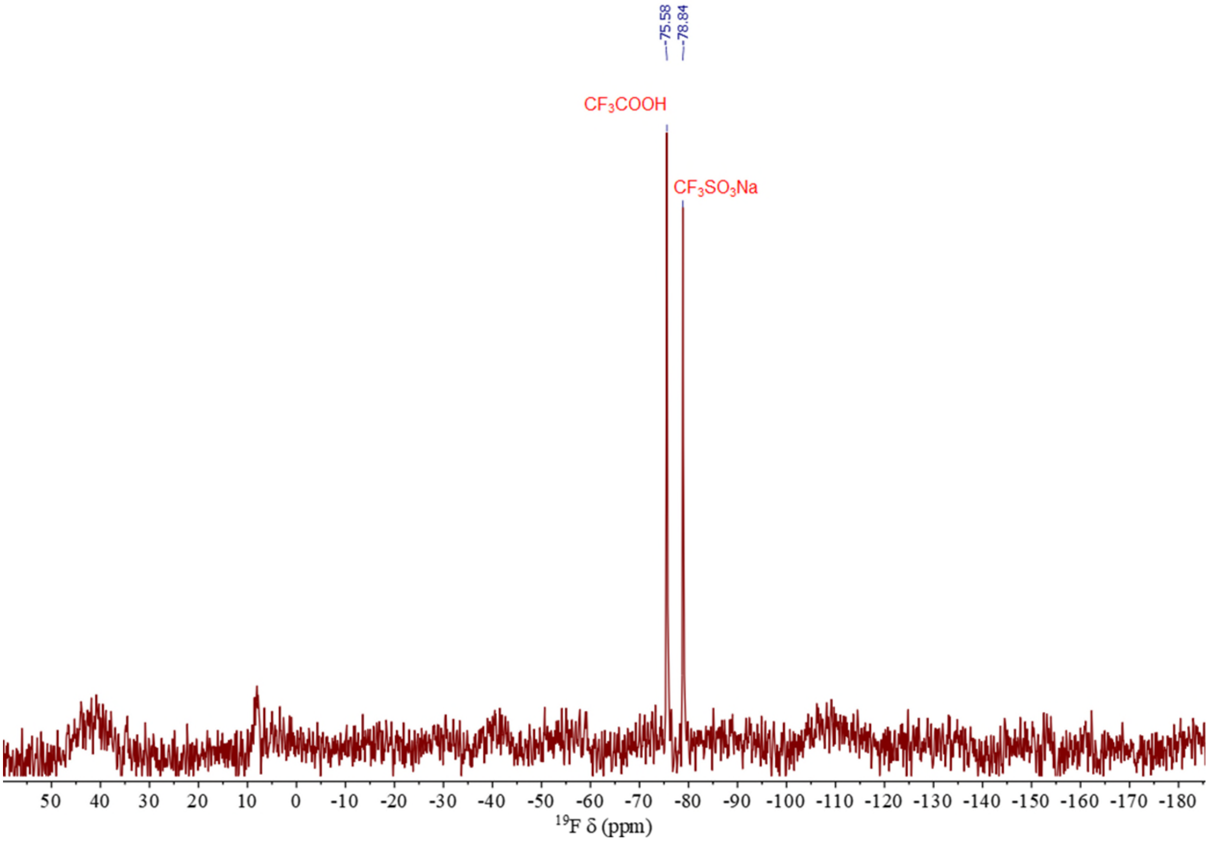

Figure S19: 377 MHz ^{19}F NMR spectrum of [Eu_2(**DO3A**)$_2$**C-2**] in D_2O at 298 K.

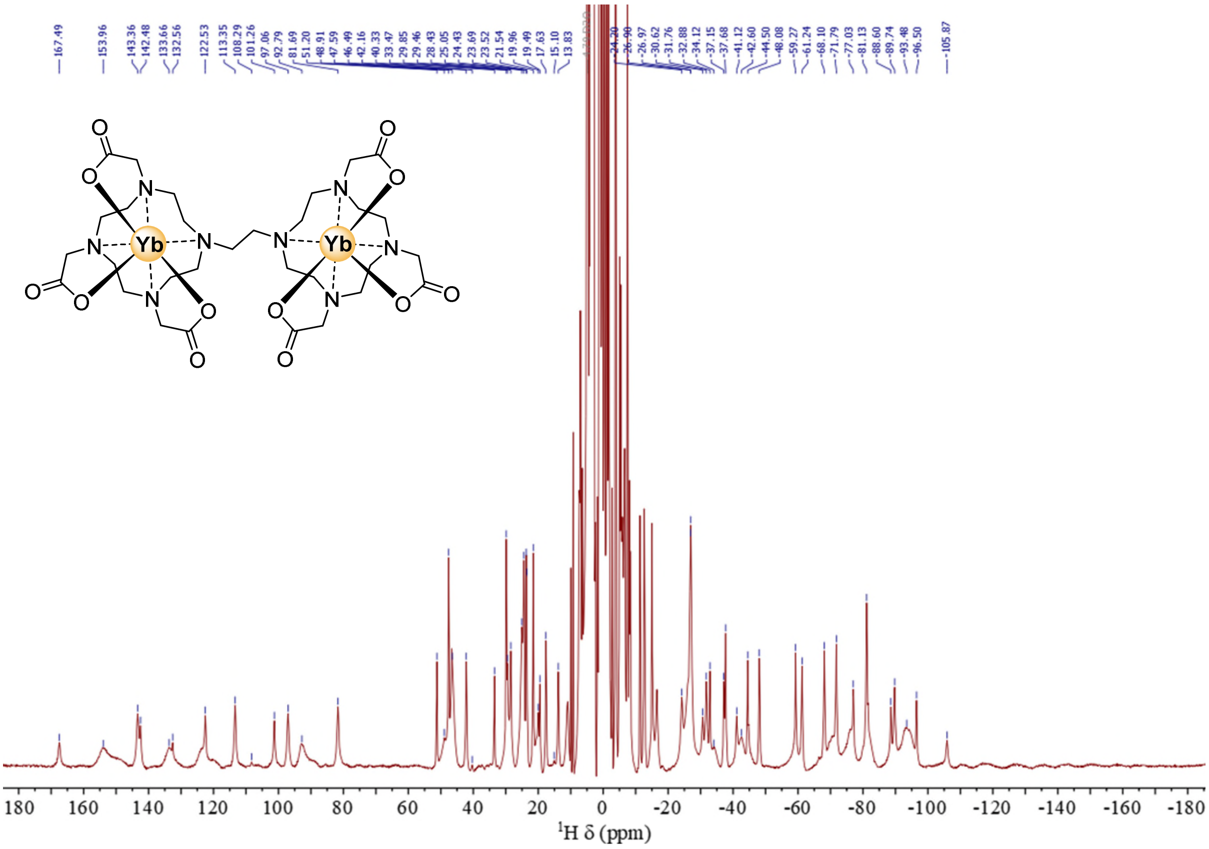

Figure S20: 500 MHz ^{1}H NMR spectrum of [Yb_2(**DO3A**)$_2$**C-2**] in D_2O at 298 K.

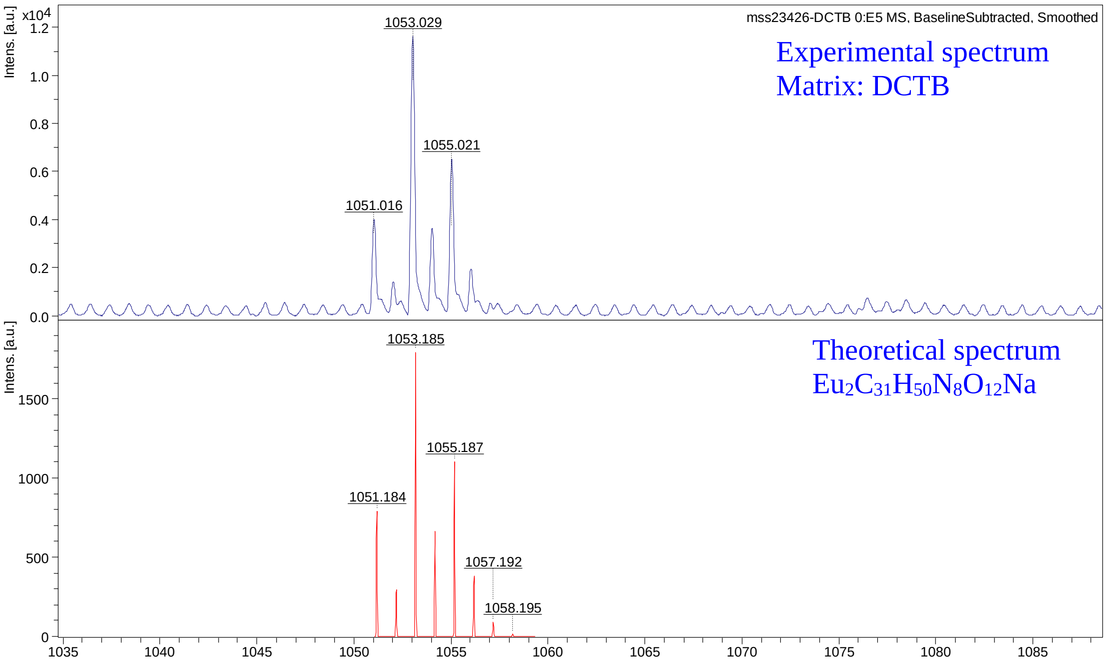

Figure S21: MALDI-TOF Mass spectrum of [Eu_2(**DO3A**)$_2$**C-3**], experimental (*top*) and theoretical (*bottom*).

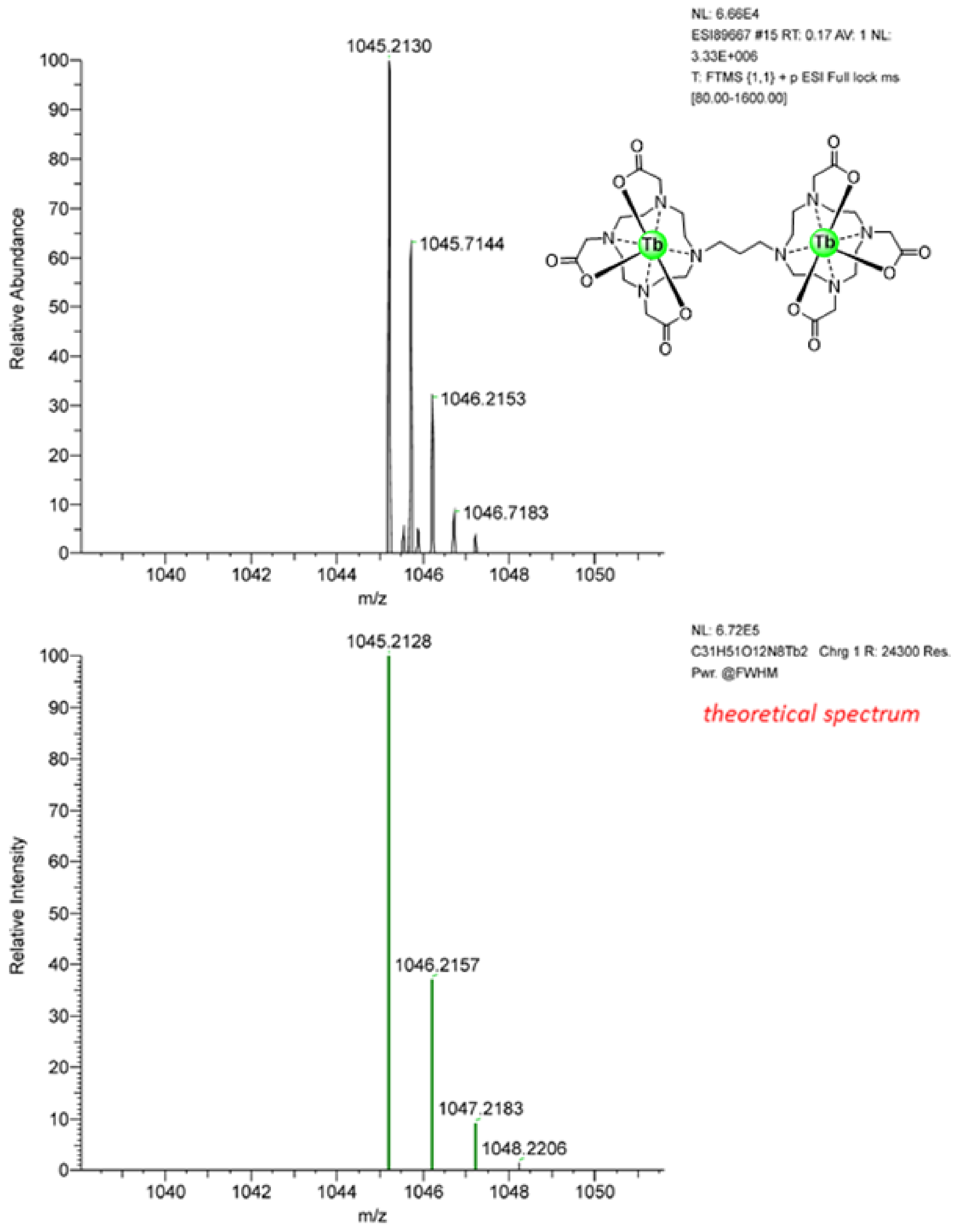

Figure S22: ESI-Mass spectrum of [Tb_2(**DO3A**)$_2$**C-3**], experimental (*top*) and theoretical (*bottom*).

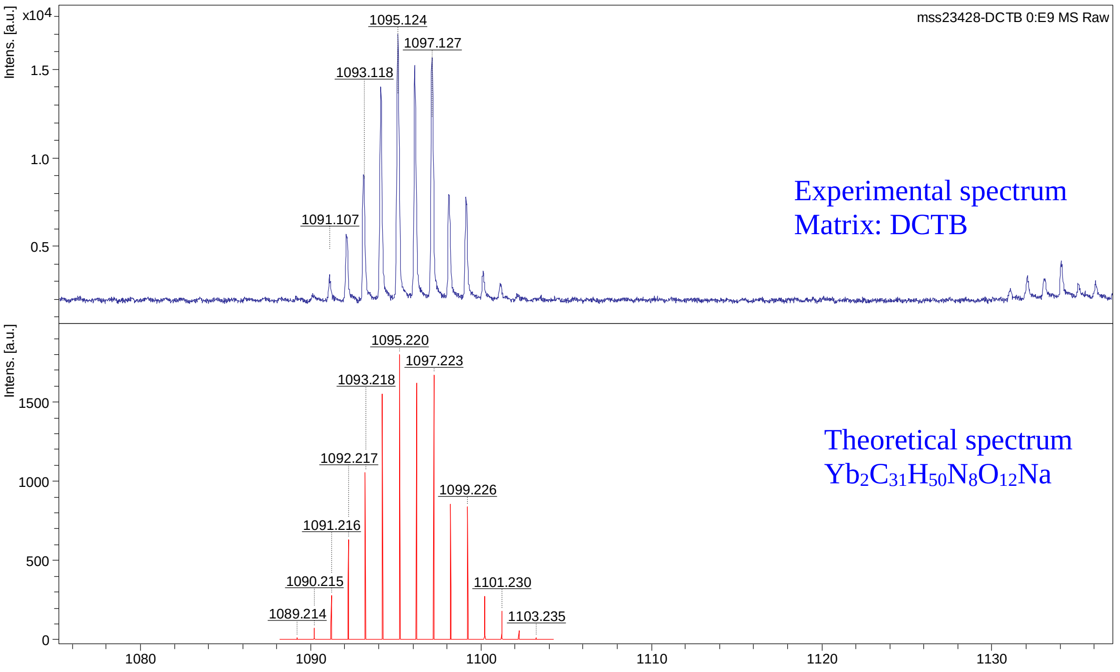

Figure S23: MALDI-TOF Mass spectrum of [Yb_2(**DO3A**)$_2$**C-3**].

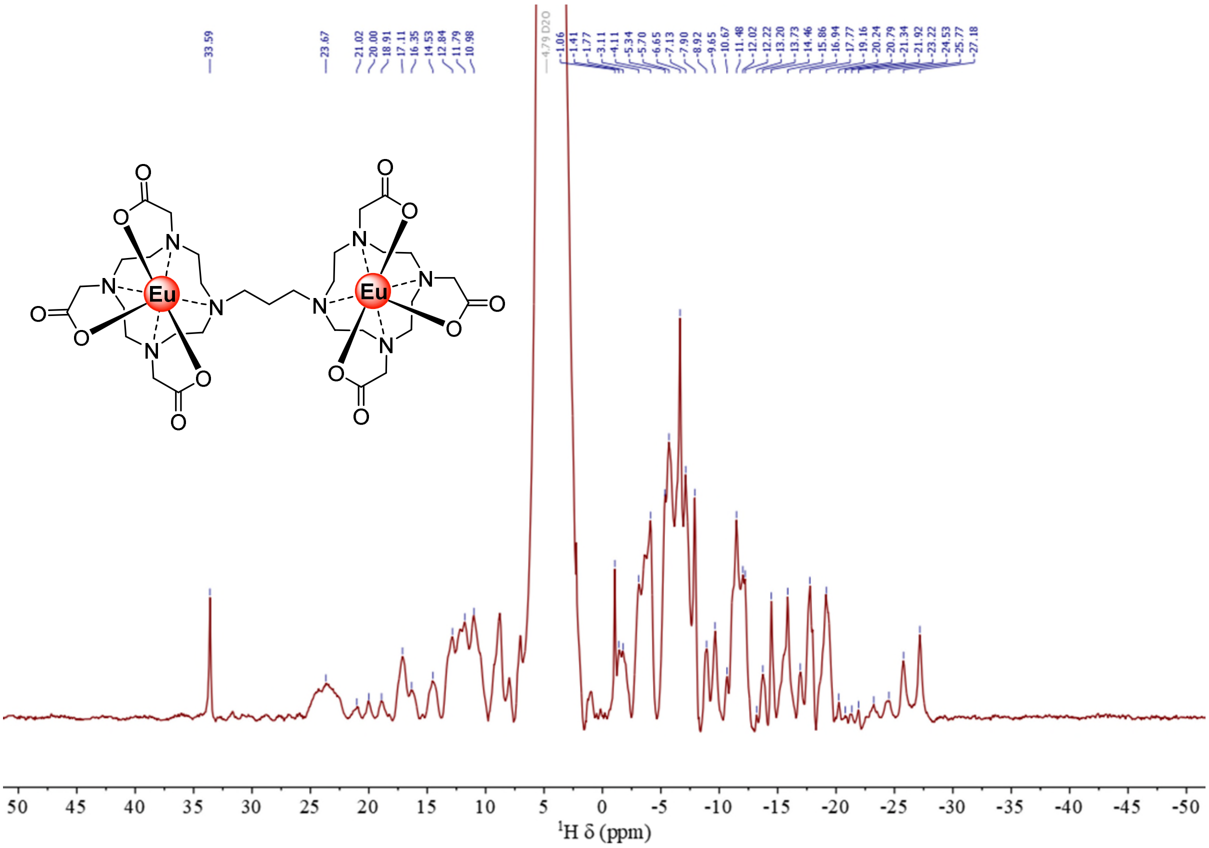

Figure S24: 500 MHz ^{1}H NMR spectrum of [Eu_2(**DO3A**)$_2$**C-3**] in D_2O at 298 K.

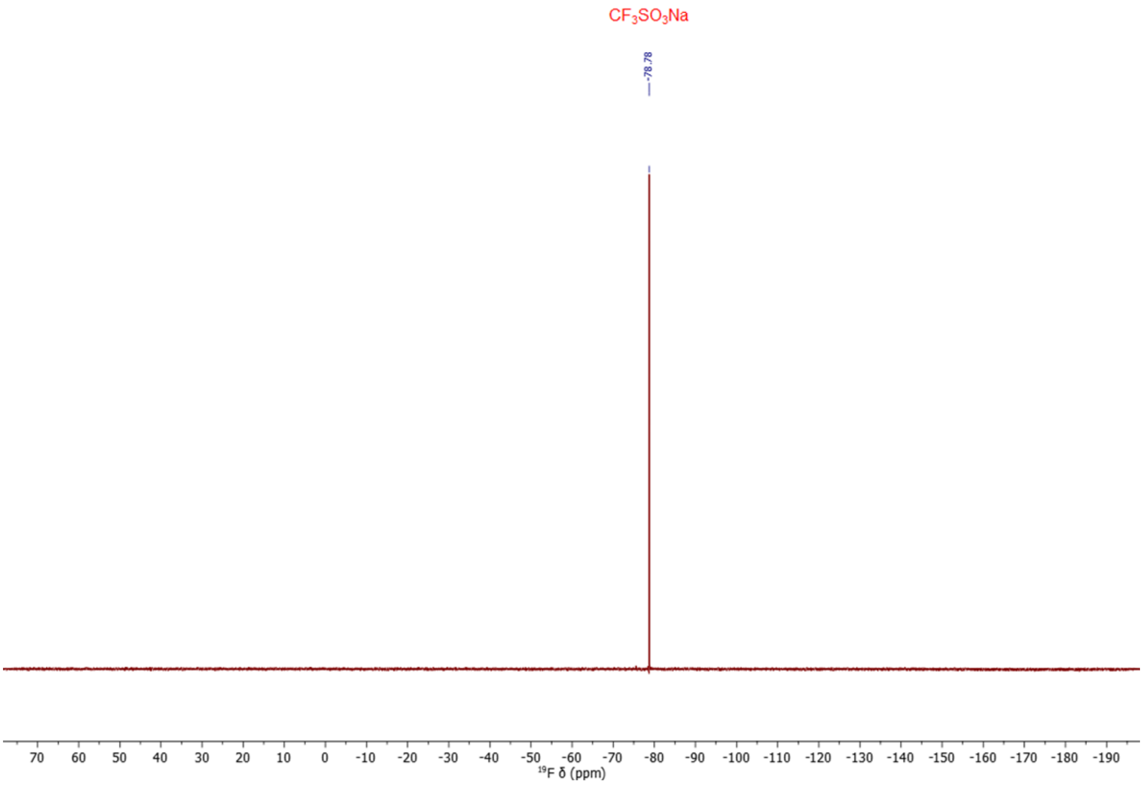

Figure S25: 377 MHz ^{19}F NMR spectrum of [Eu$_2$(**DO3A**)$_2$**C-3**] in D_2O at 298 K.

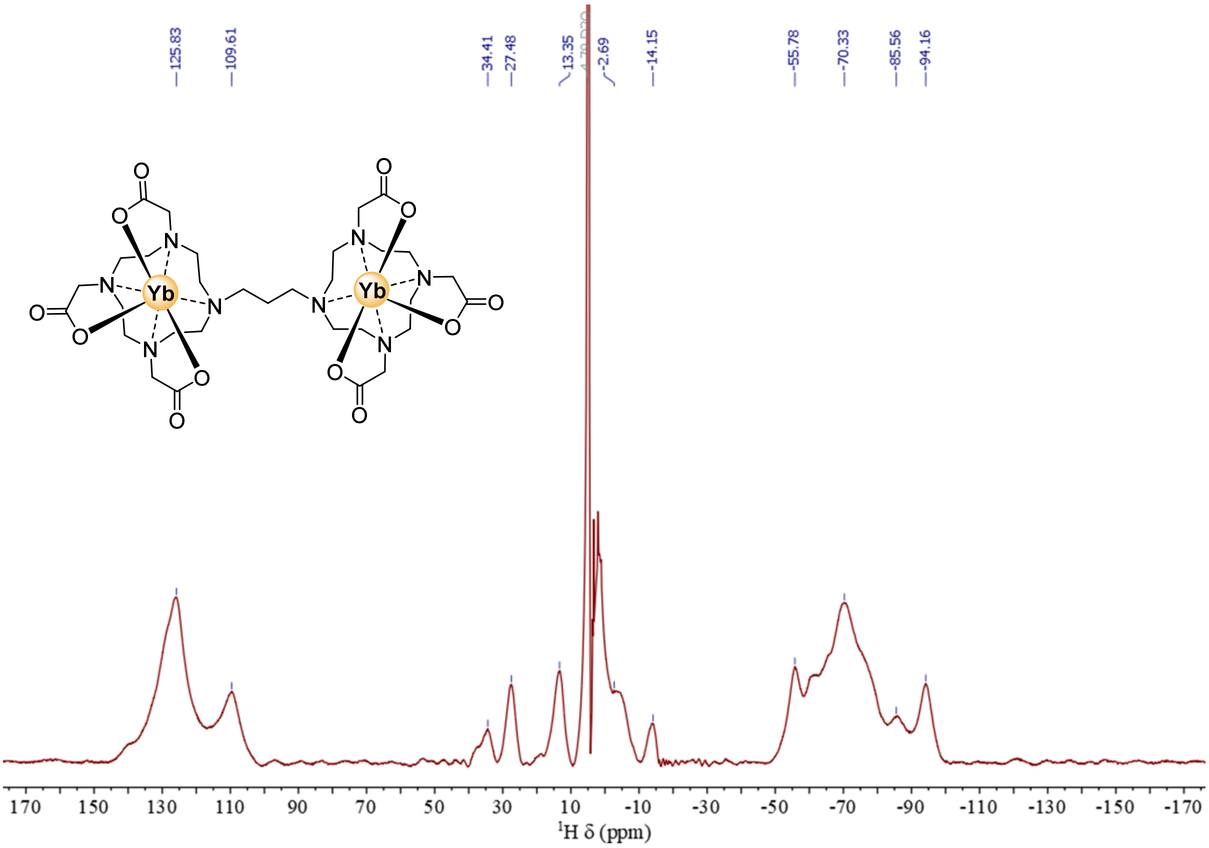

Figure S26: 500 MHz ^{1}H NMR spectrum of [Yb$_2$(**DO3A**)$_2$**C-3**] in D_2O at 298 K.

5. Paramagnetic ^{1}H NMR spectra of mono- and binuclear Ln(III) complexes

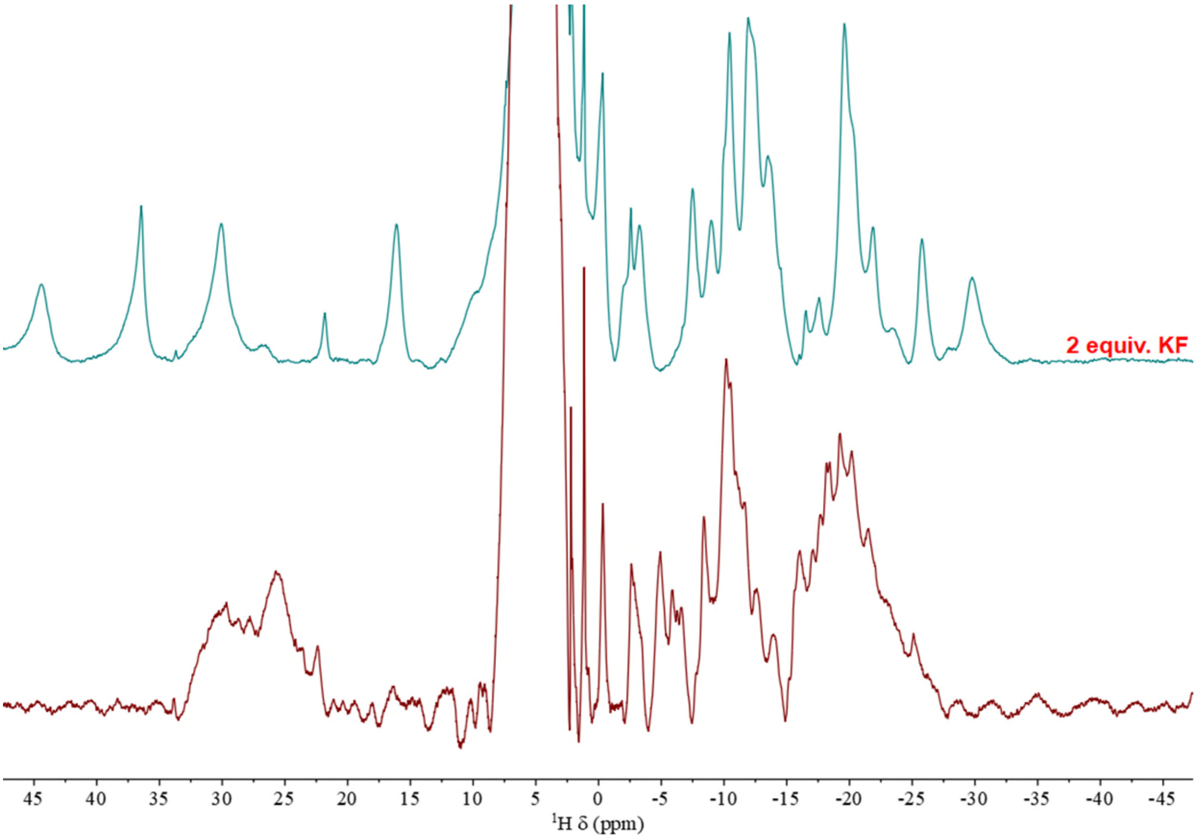

Figure S27: 500 MHz stacked ^{1}H NMR spectra of [Eu_2(**DO3A**)$_2$**C-2**] with and without KF in D_2O at 298 K.

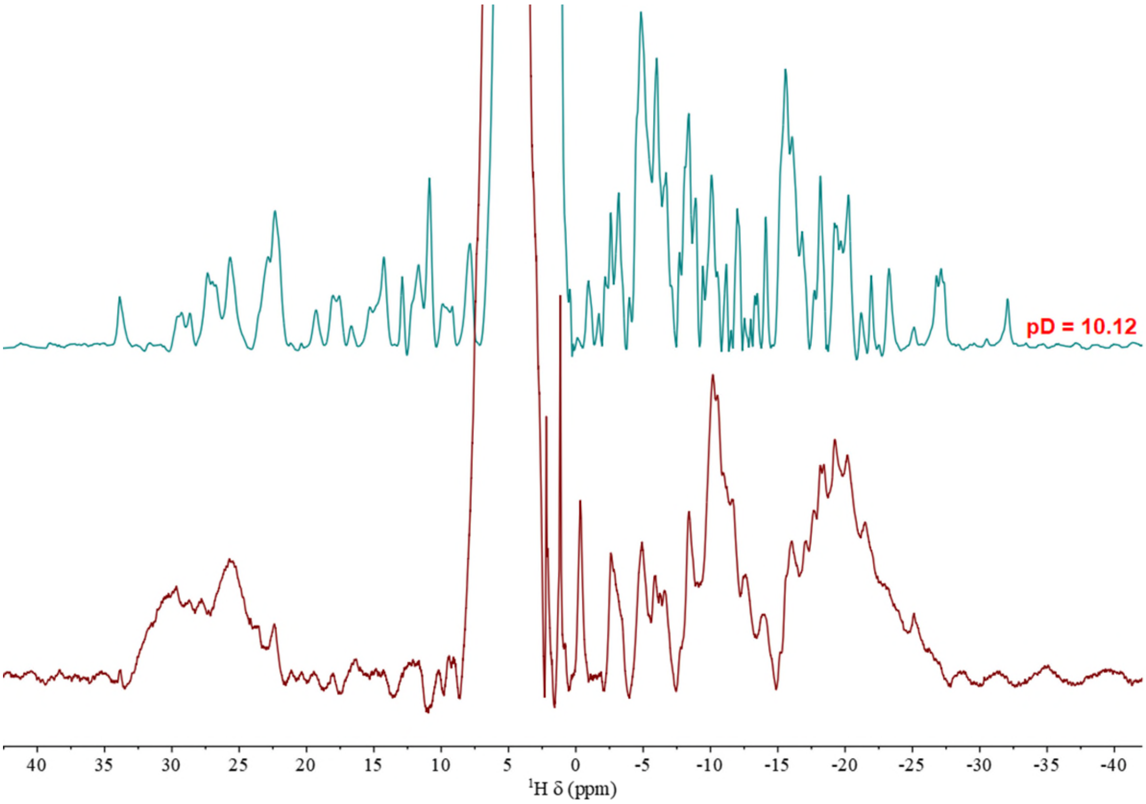

Figure S28: 500 MHz stacked ^{1}H NMR spectra of [Eu_2(**DO3A**)$_2$**C-2**] in D_2O and pD 10.12 at 298 K.

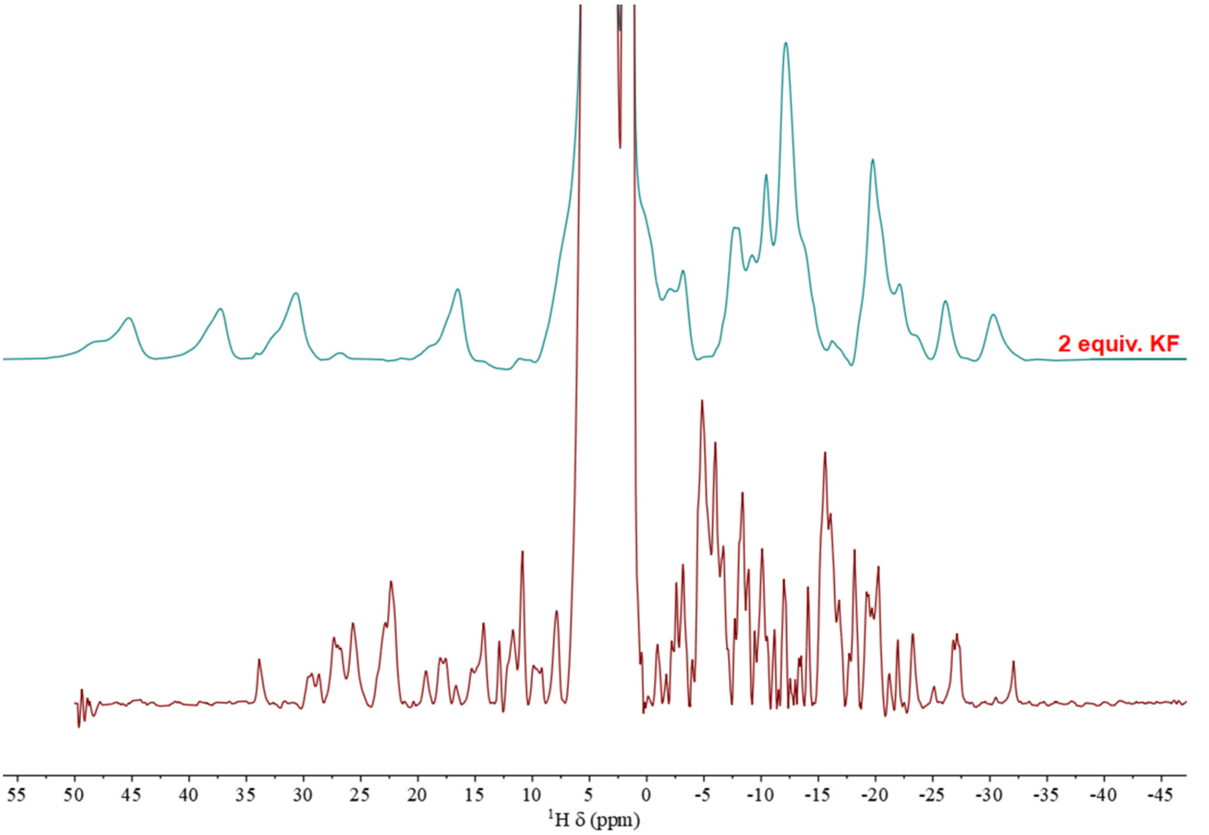

Figure S29: 500 MHz stacked ^{1}H NMR spectra of [Eu_2(**DO3A**)$_2$**C-2**] with and without KF in pD 10.12 at 298 K.

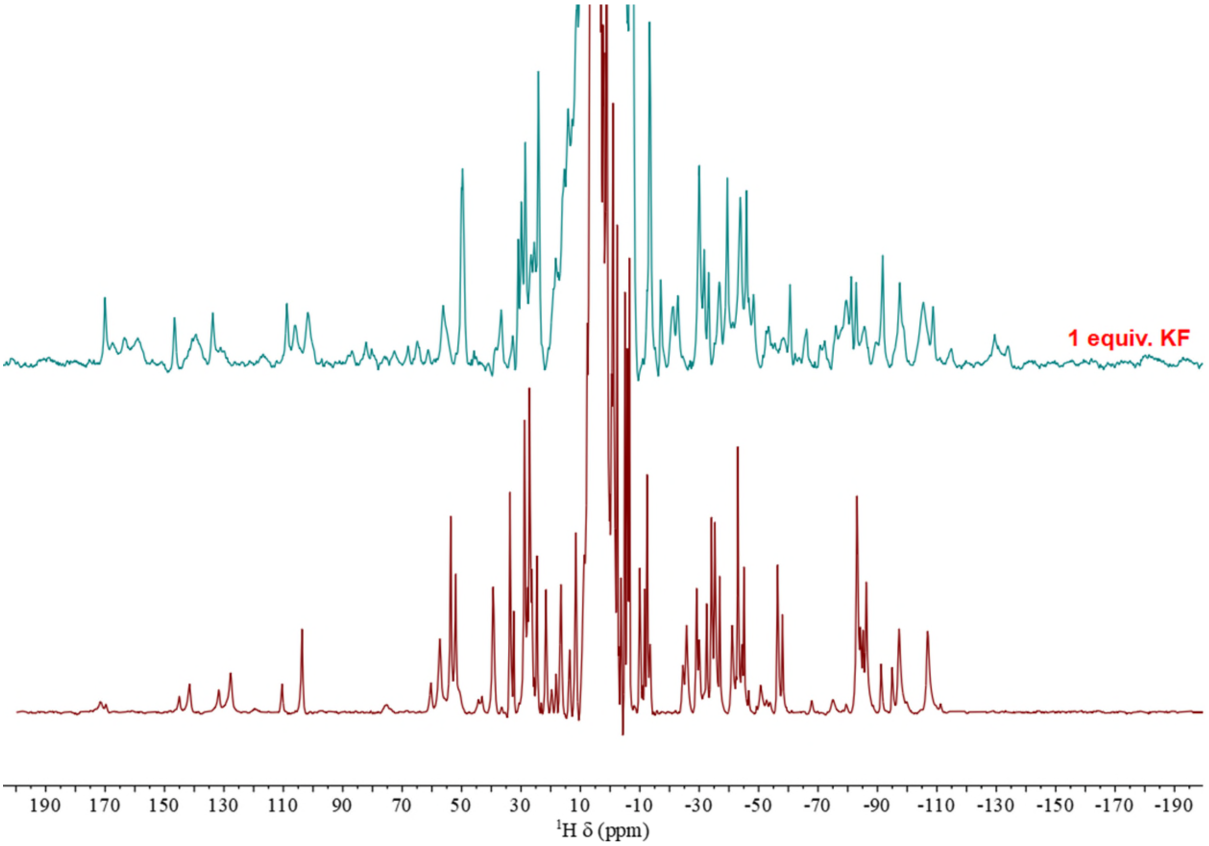

Figure S30: 500 MHz stacked ^{1}H NMR spectra of [Yb_2(**DO3A**)$_2$**C-2**] with and without KF in CD_3OD at 298 K.

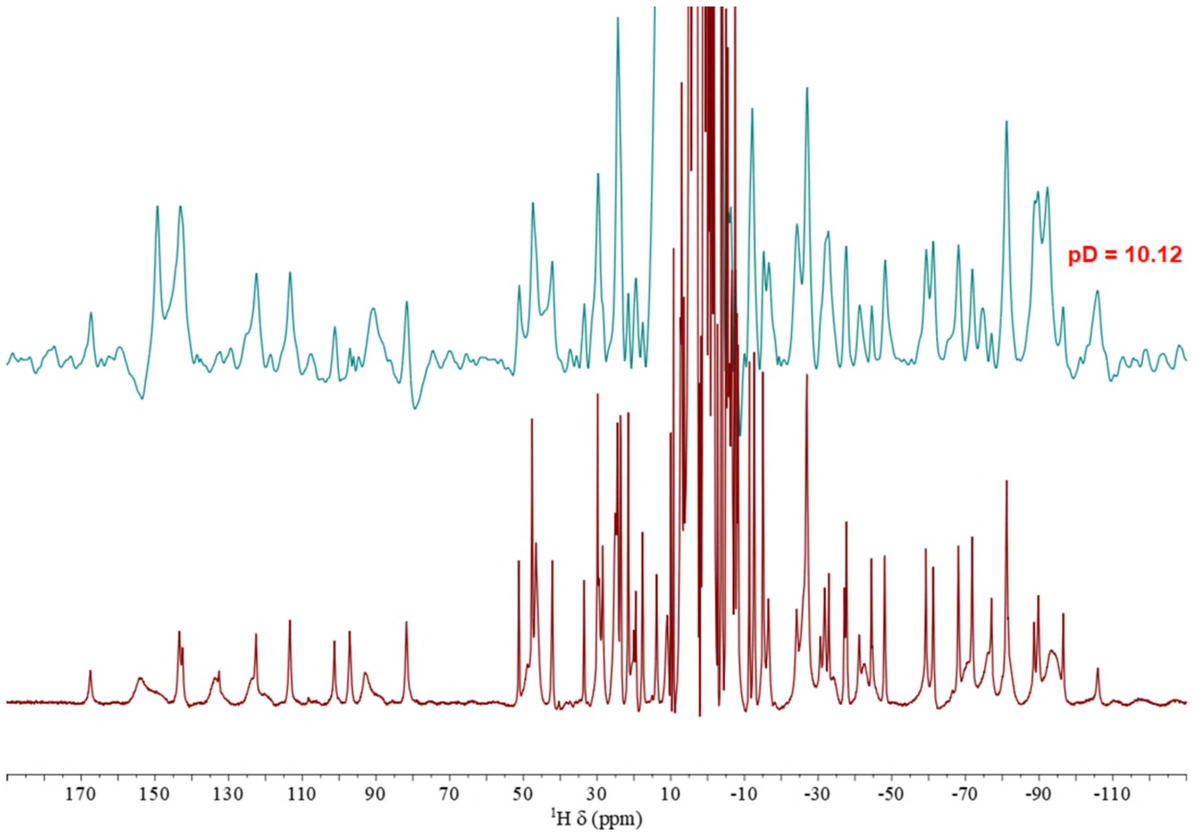

Figure S31: 500 MHz stacked ^{1}H NMR spectra of [Yb_2(**DO3A**)$_2$**C-2**] in D_2O (pD 10.12) at 298 K.

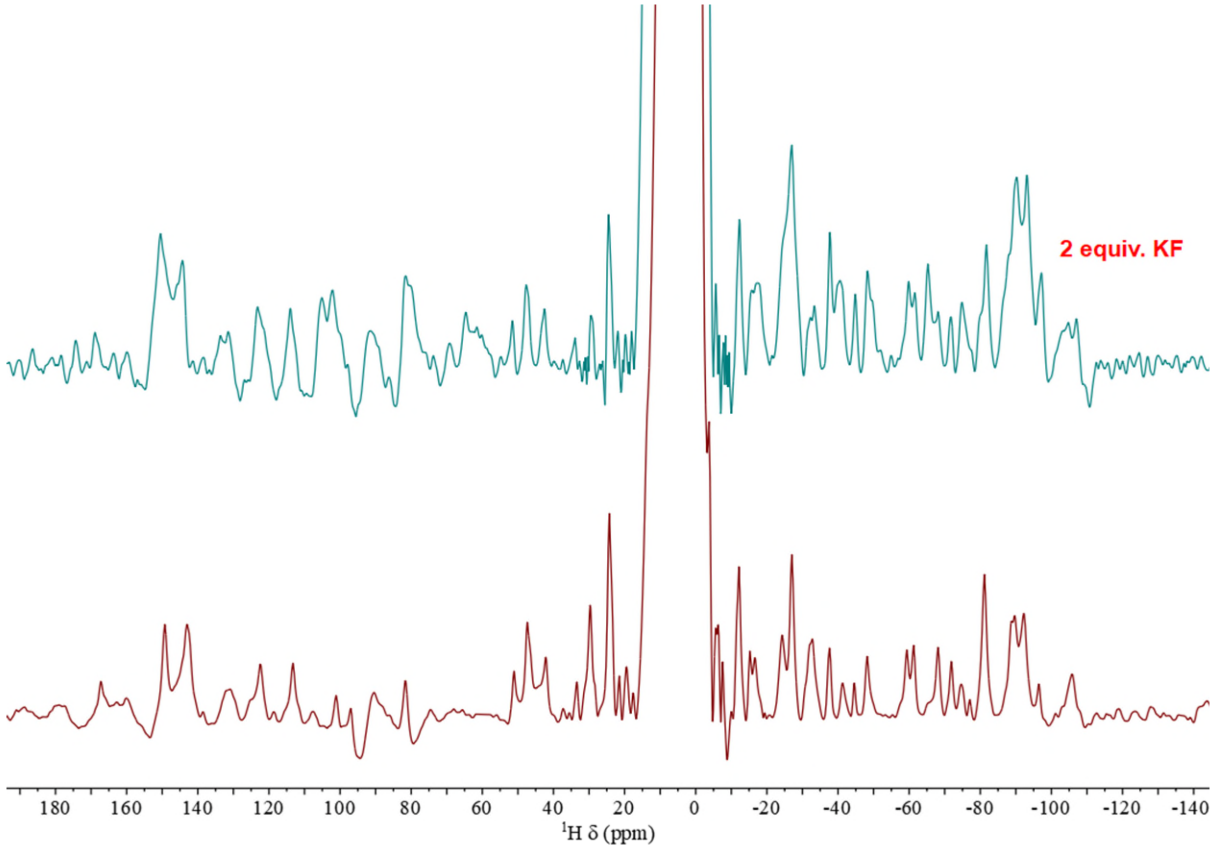

Figure S32: 500 MHz stacked ^{1}H NMR spectra of [Yb_2(**DO3A**)$_2$**C-2**] with and without KF (pD 10.12) at 298 K.

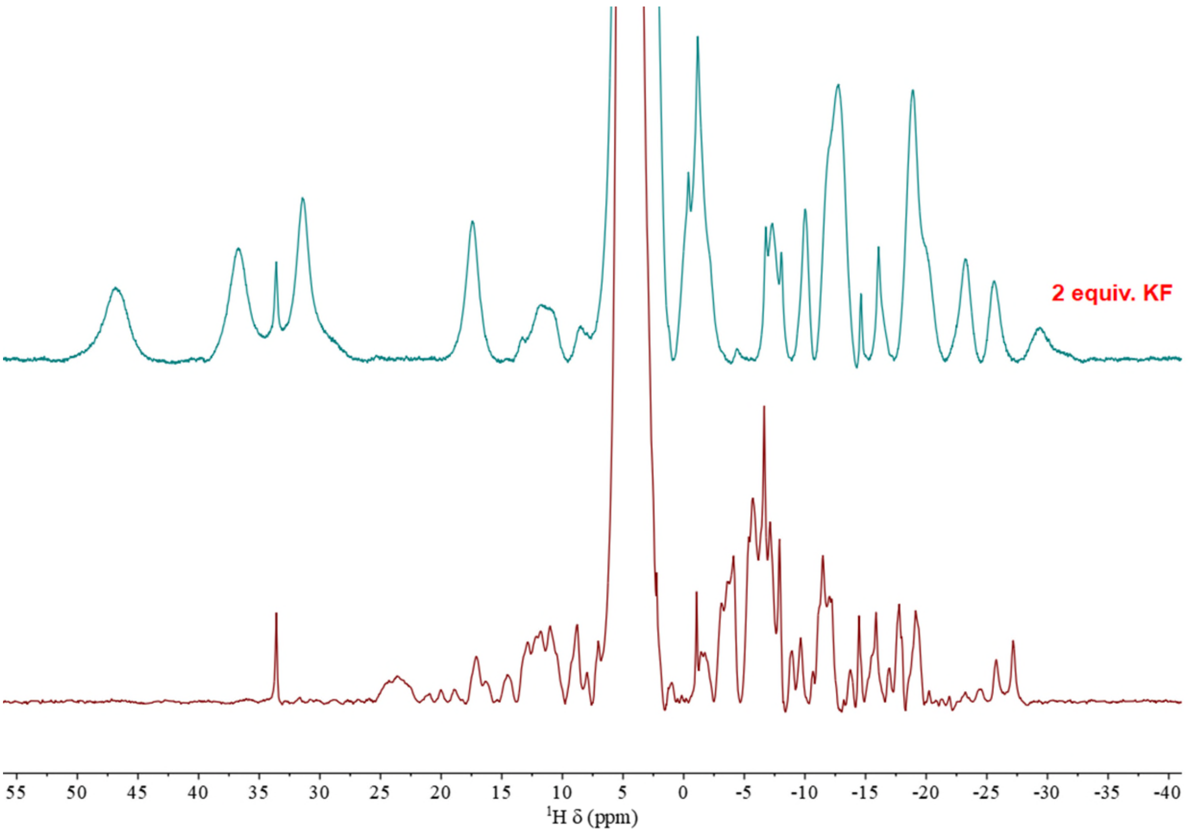

Figure S33: 500 MHz Stacked ^{1}H NMR spectra of [Eu_2(**DO3A**)$_2$**C-3**] with and without KF in D_2O at 298 K.

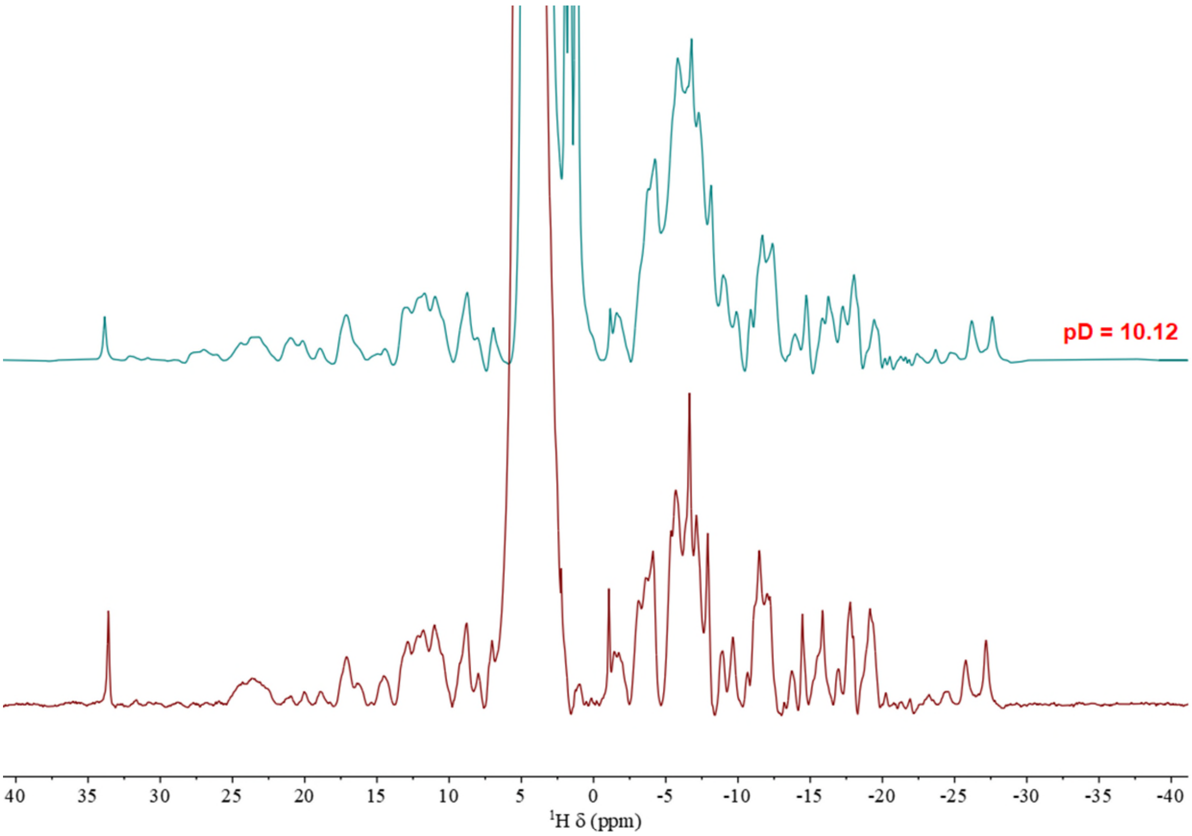

Figure S34: 500 MHz stacked ^{1}H NMR spectra of [Eu_2(**DO3A**)$_2$**C-3**] in D_2O (pD 10.12) at 298 K.

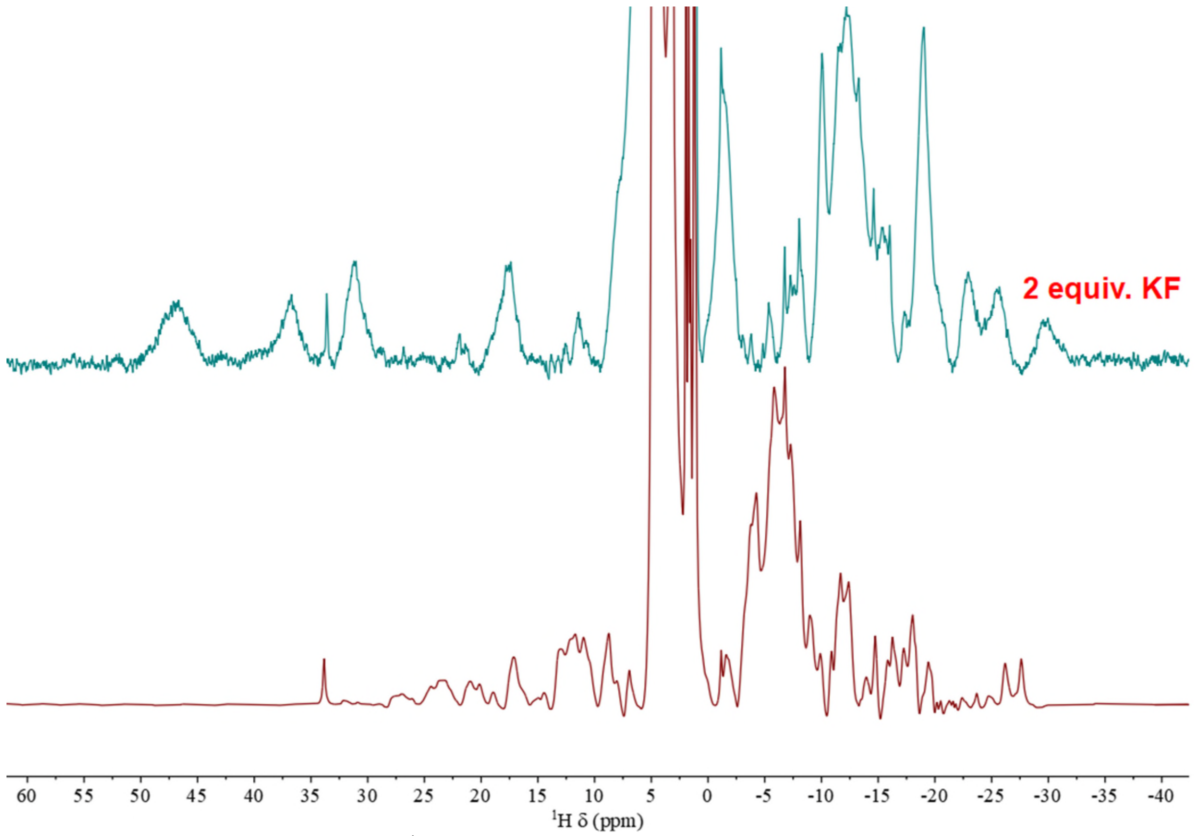

Figure S35: 500 MHz stacked ^{1}H NMR spectra of [Eu_2(**DO3A**)$_2$**C-3**] with and without KF (pD 10.12) at 298 K.

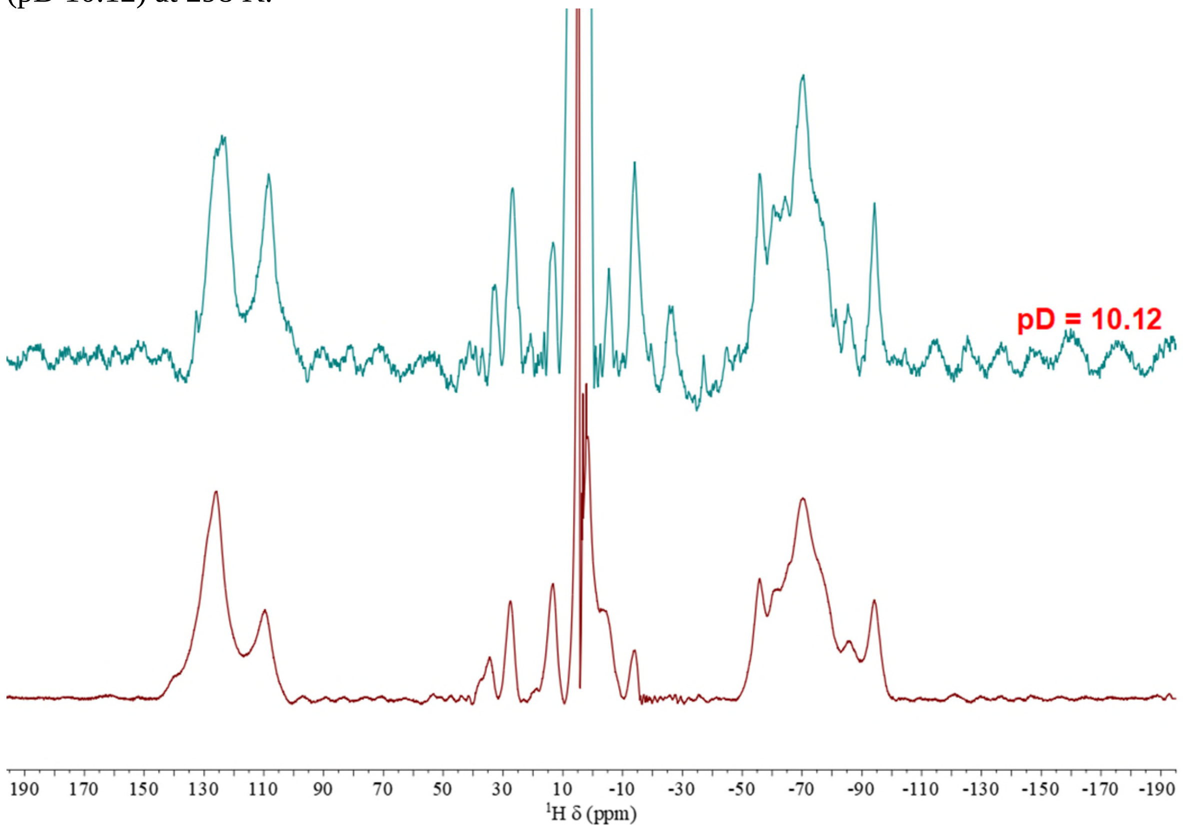

Figure S36: 500 MHz stacked ^{1}H NMR spectra of [Yb_2(**DO3A**)$_2$**C-3**] in D_2O (pD 10.12) at 298 K.

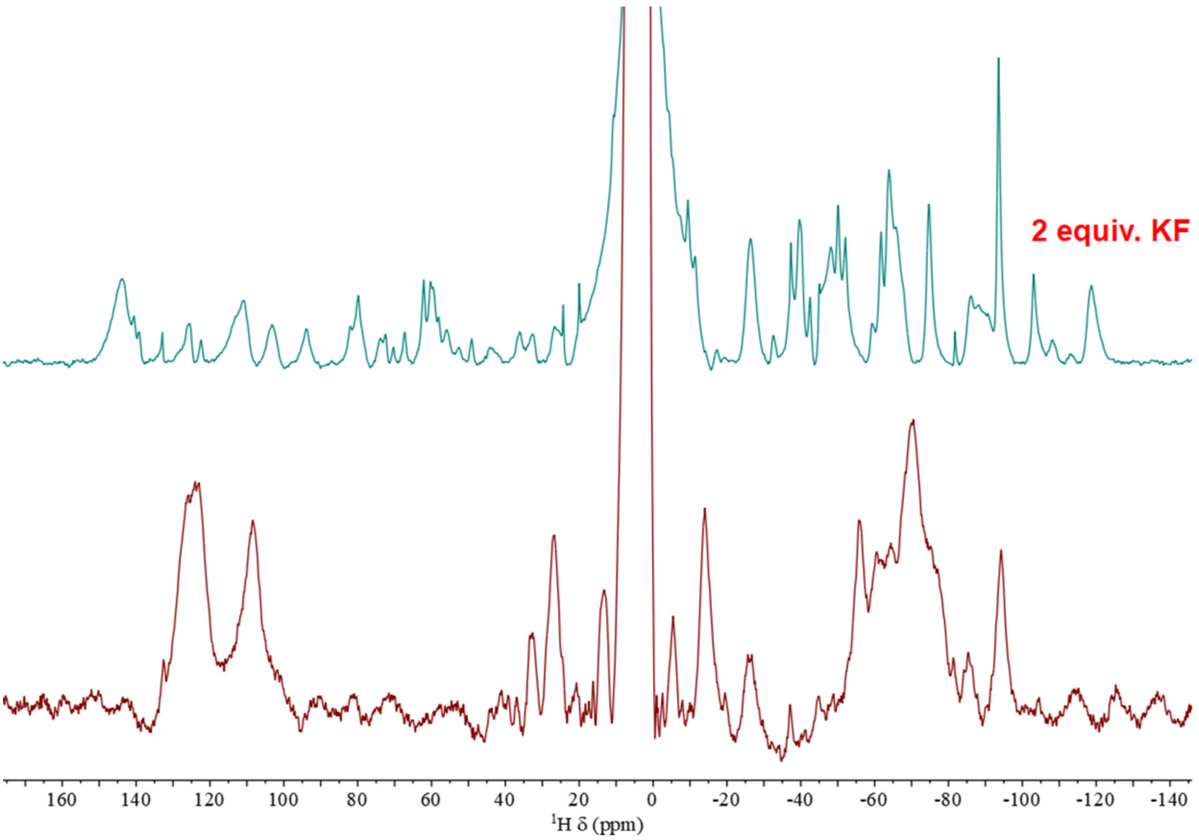

Figure S37: 500 MHz stacked ^{1}H NMR spectra of [Yb_2(**DO3A**)$_2$**C-3**] with and without KF (pD 10.12) at 298 K.

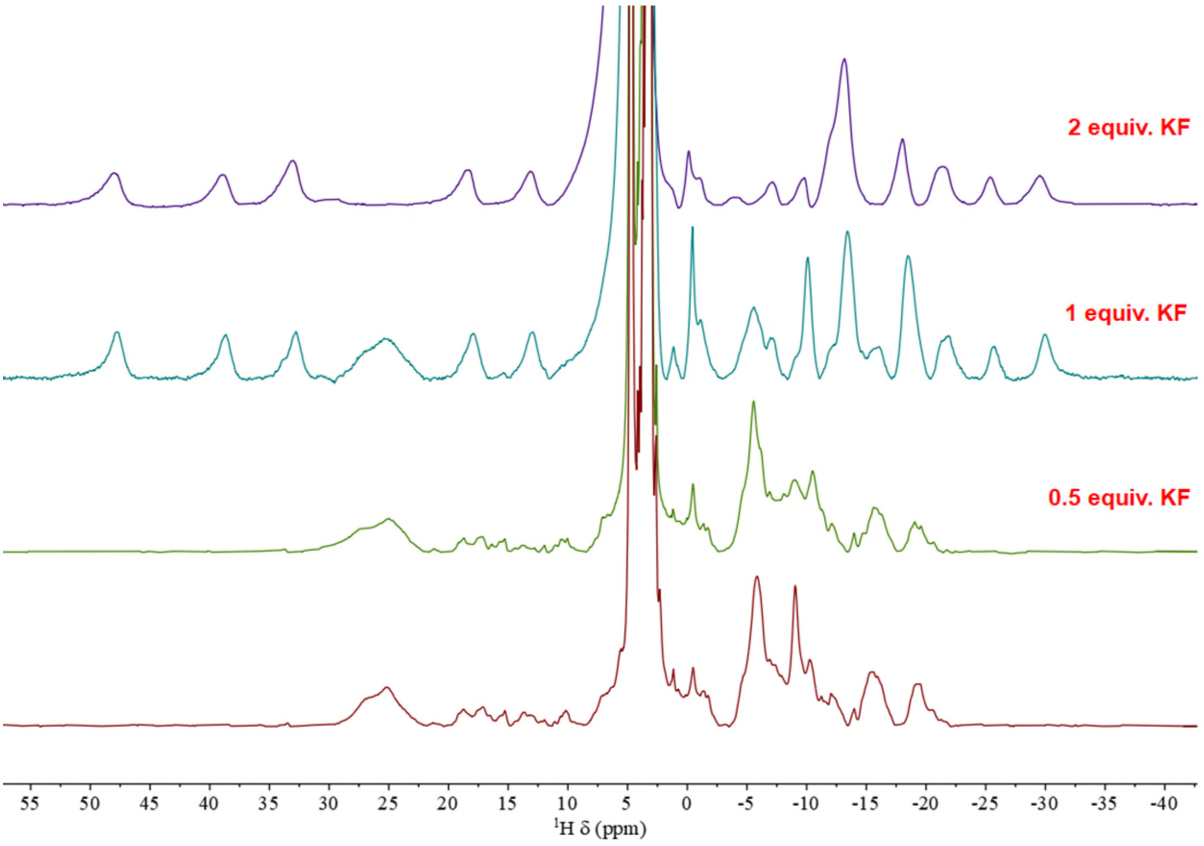

Figure S38: 400 MHz stacked ^{1}H NMR spectra of [Eu(**pDO3A**)] with increasing KF in D_2O at 298 K.

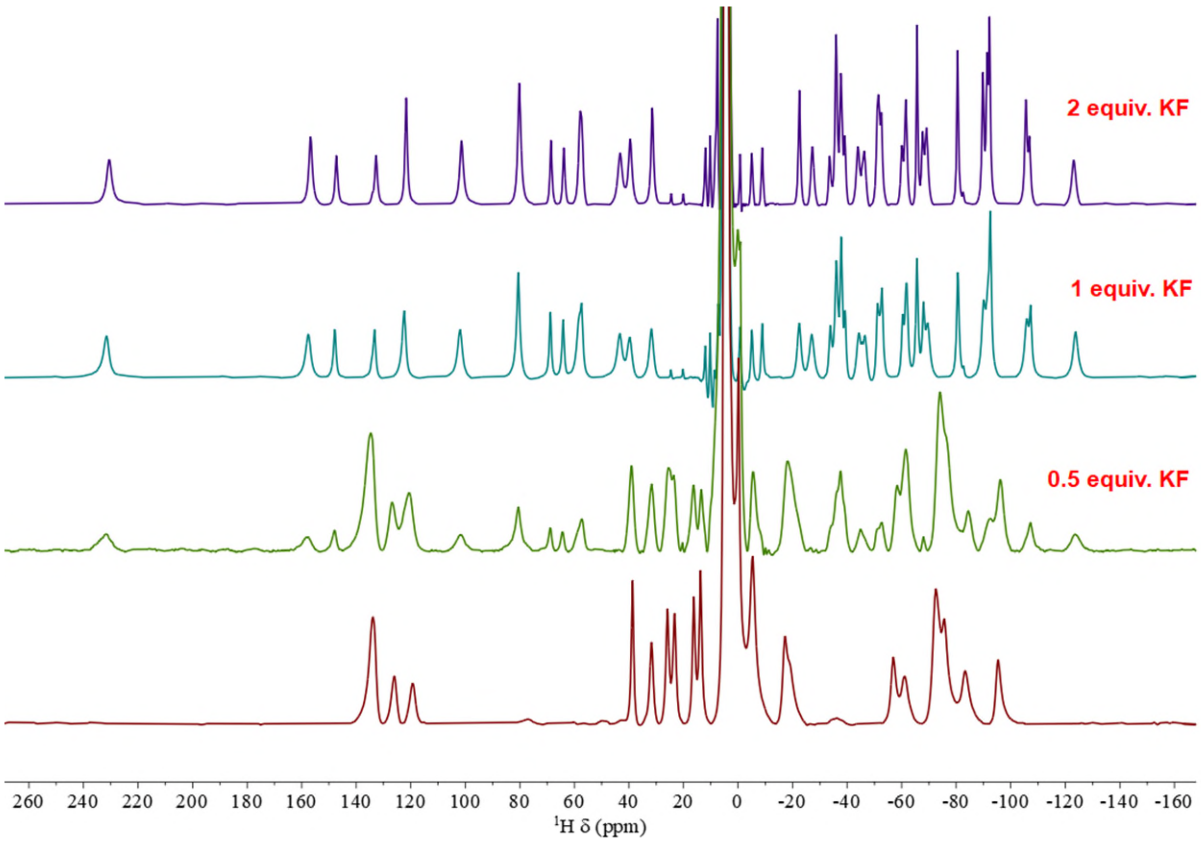

Figure S39: 400 MHz Stacked ^{1}H NMR spectra of [Yb(**pDO3A**)] with increasing KF in D_2O at 298 K.

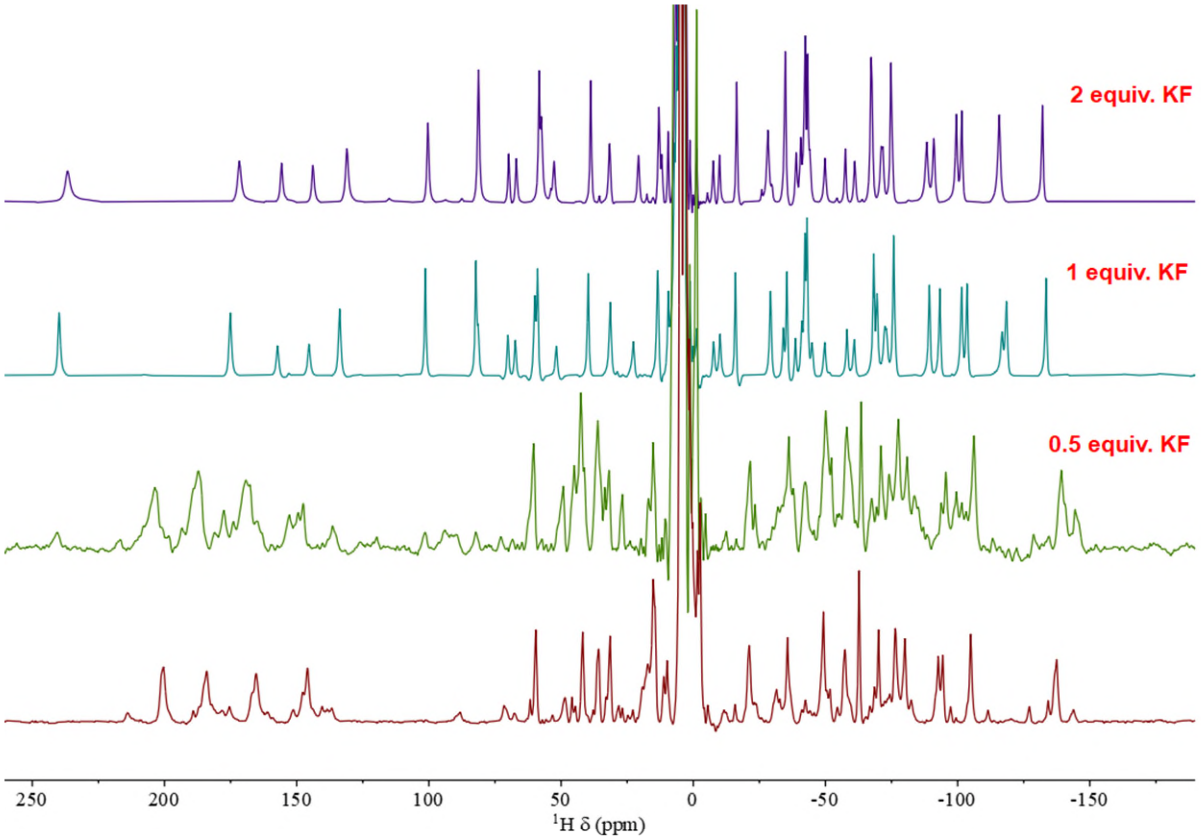

Figure S40: 400 MHz stacked ^{1}H NMR spectra of [Yb(**pDO3A**)] with increasing KF in CD_3OD at 298 K.

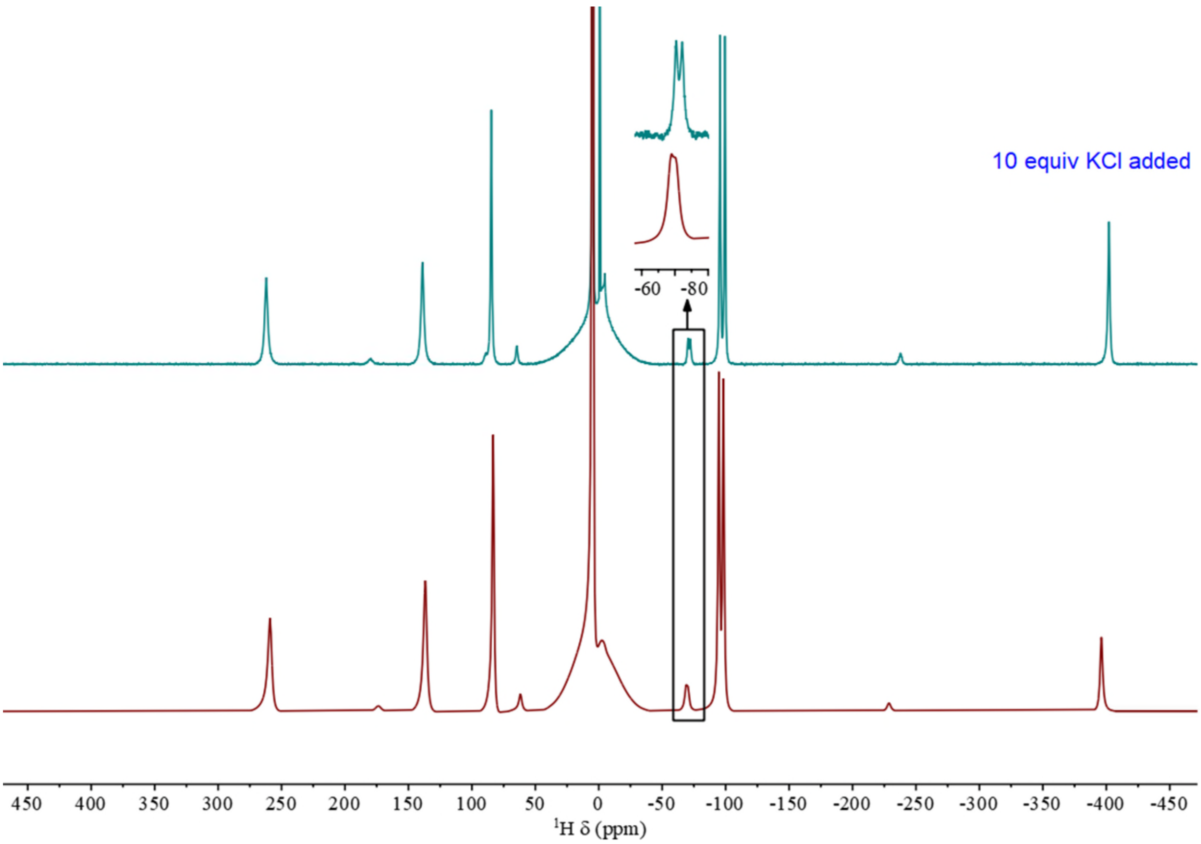

Figure S41: 400 MHz stacked ^{1}H NMR spectra of [Tb(**DOTA**)]$^-$ with and without KCl in D_2O at 298 K. Concentration of the complex used was 58 mM.

6. ^{19}F and ^{35}Cl NMR spectra of complexes in the presence of KF and KCl, binding isotherms, and van't Hoff plot

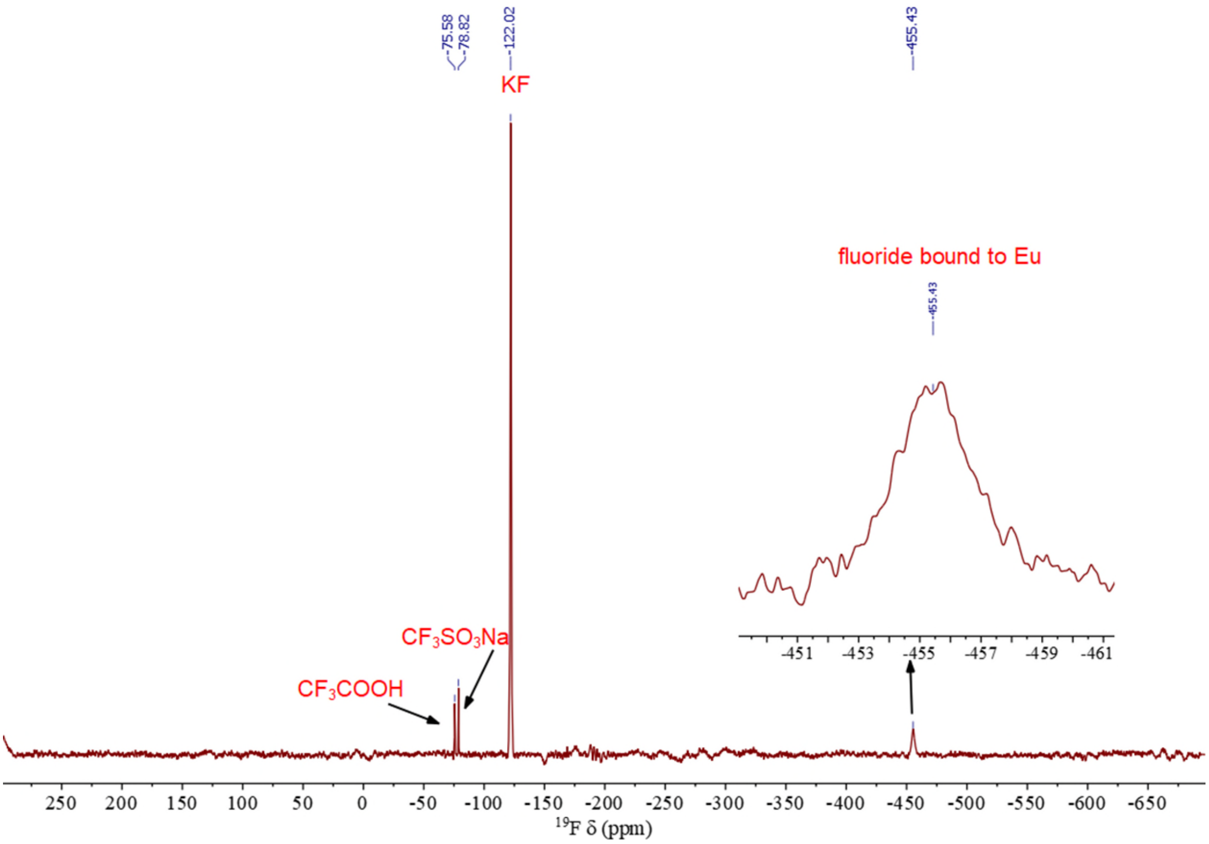

Figure S42: 470 MHz ^{19}F NMR spectrum of [Eu_2(**DO3A**)$_2$**C-2**] with 5 equiv. KF in D_2O at 298 K.

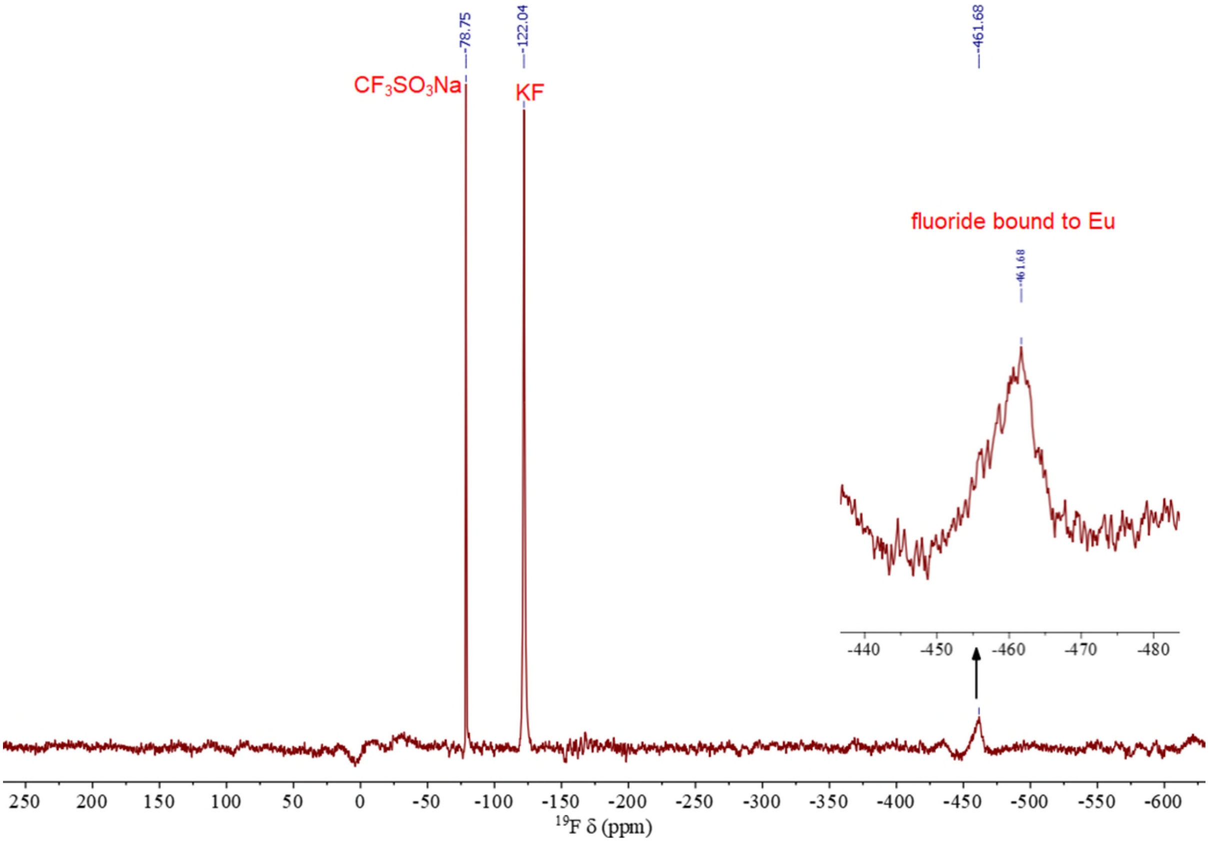

Figure S43: 470 MHz ^{19}F NMR spectrum of [Eu_2(**DO3A**)$_2$**C-3**] with 5 equiv. KF in D_2O at 298 K.

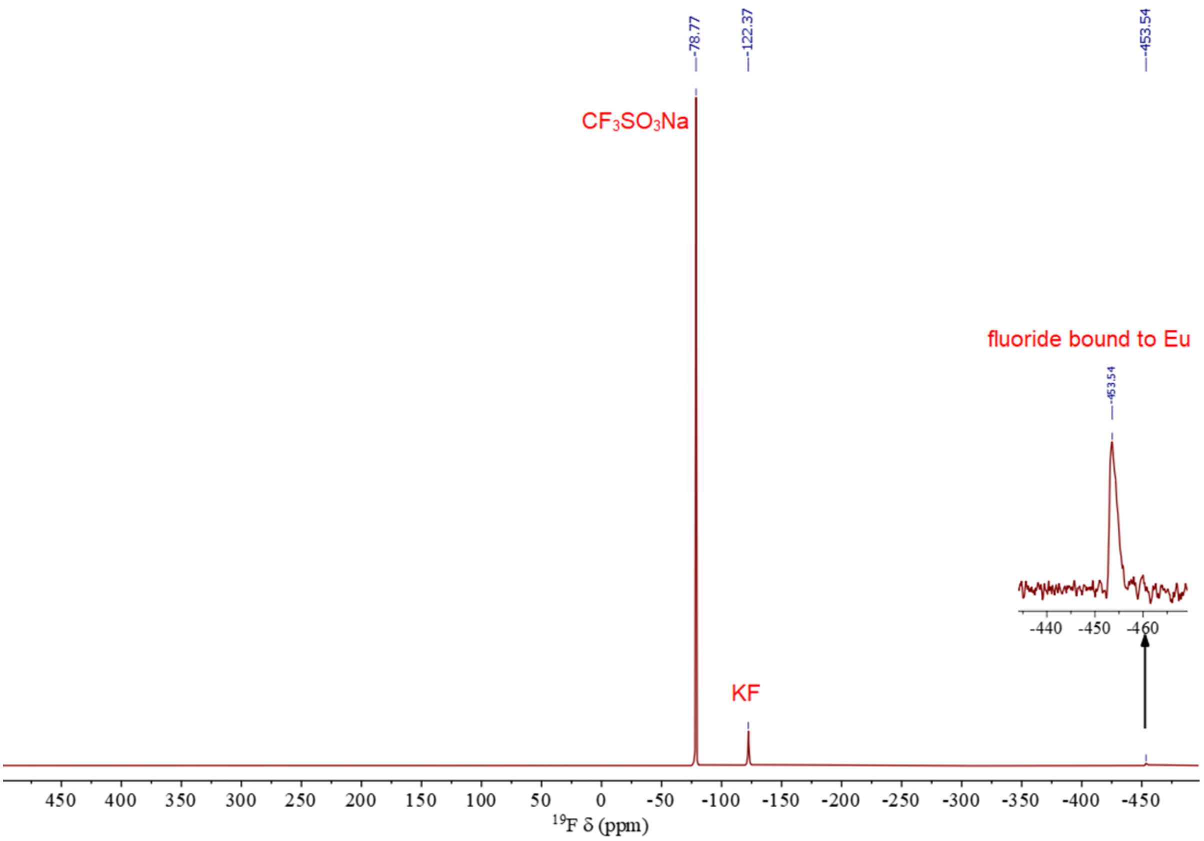

Figure S44: 470 MHz ^{19}F NMR spectrum of [Eu(**pDO3A**)] with 5 equiv. KF in D_2O at 298 K.

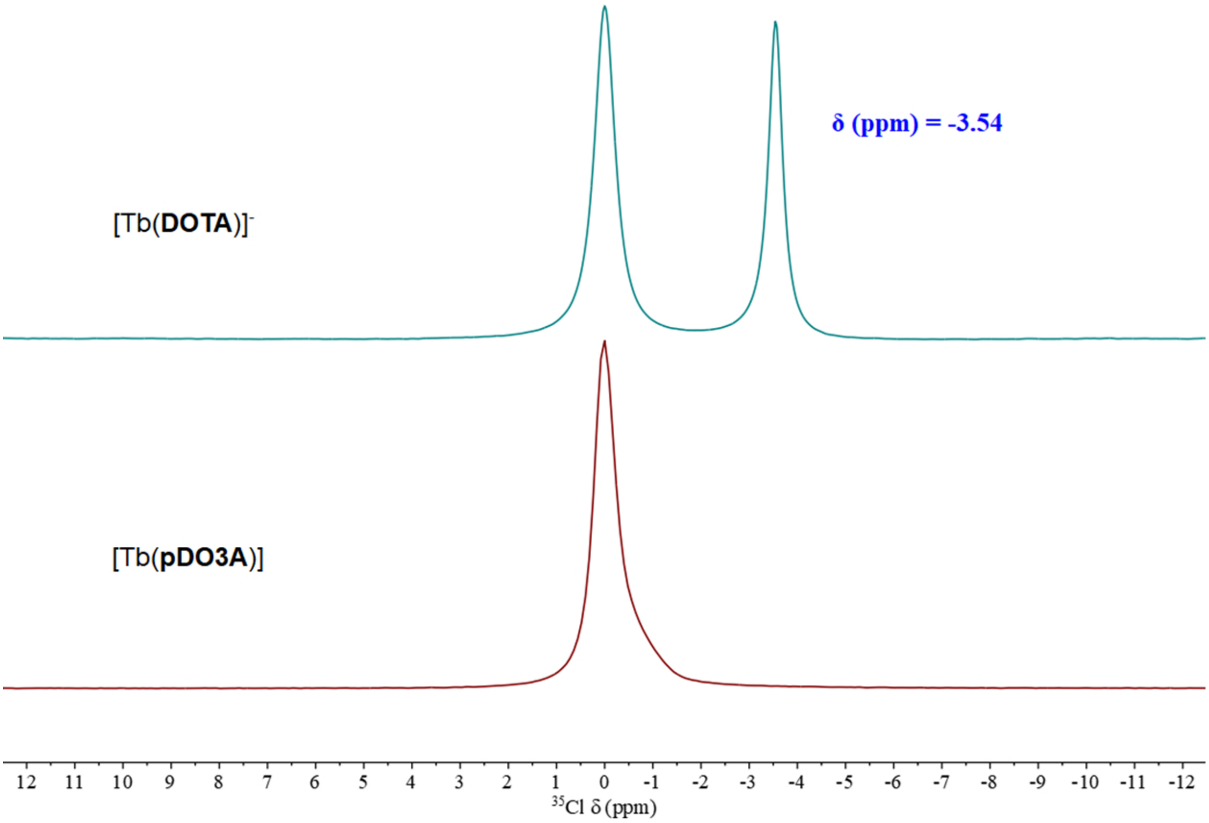

Figure S45: 49MHz stacked ^{35}Cl NMR spectra of [Tb(**pDO3A**)] and [Tb(**DOTA**)]$^{-}$ with 10 equiv KCl in D_2O at 298 K. Spectra were recorded with a capillary tube insert containing saturated KCl in D_2O. (δ (ppm) in blue = [Tb(**DOTA**)**Cl**]$^{-}$) Concentration of the complexes were 58 mM.

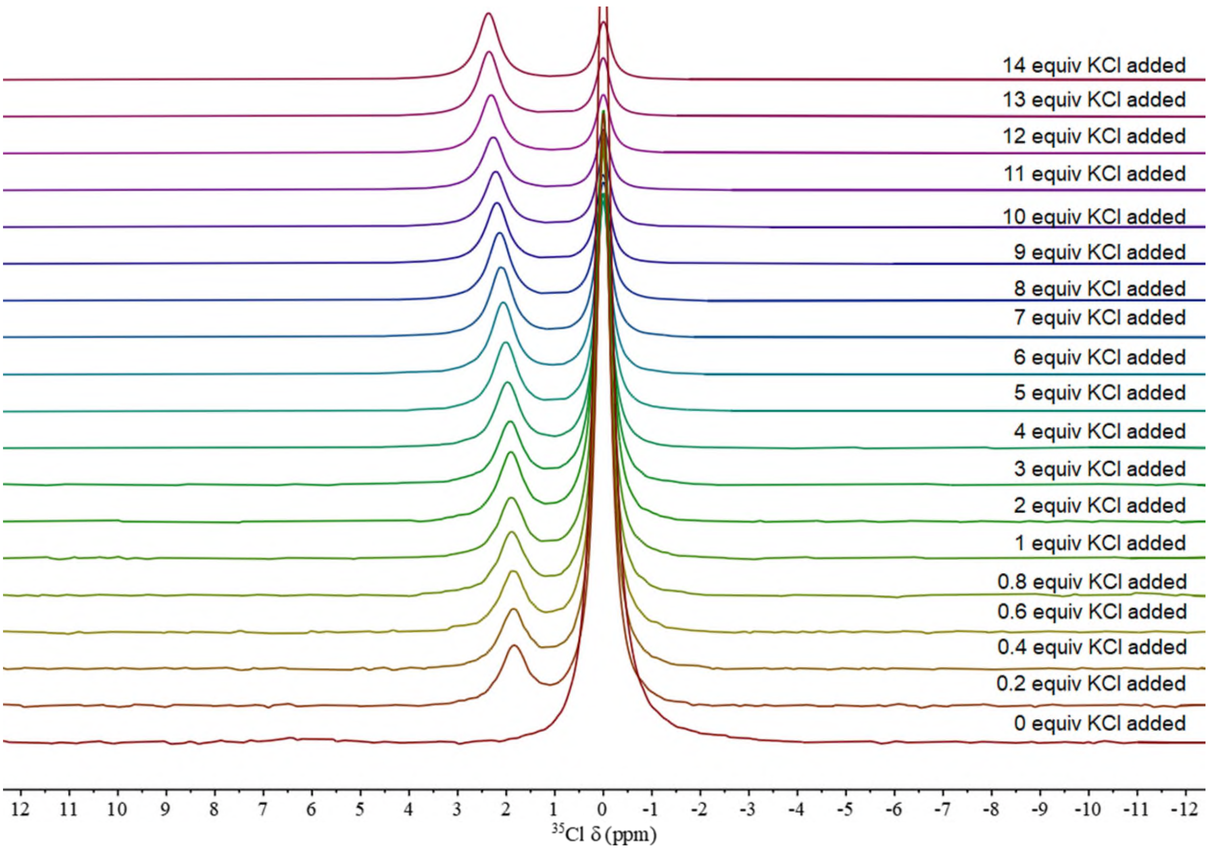

Figure S46: 49 MHz ^{35}Cl NMR titration spectra of 0.035 M [Tb$_2$(**DO3A**)$_2$**C-3**] with increasing concentration of KCl (stock concentration = 1.4 M) in D_2O at 304 K. Spectra were recorded with a capillary tube insert containing saturated KCl in D_2O (non-dilution method used).

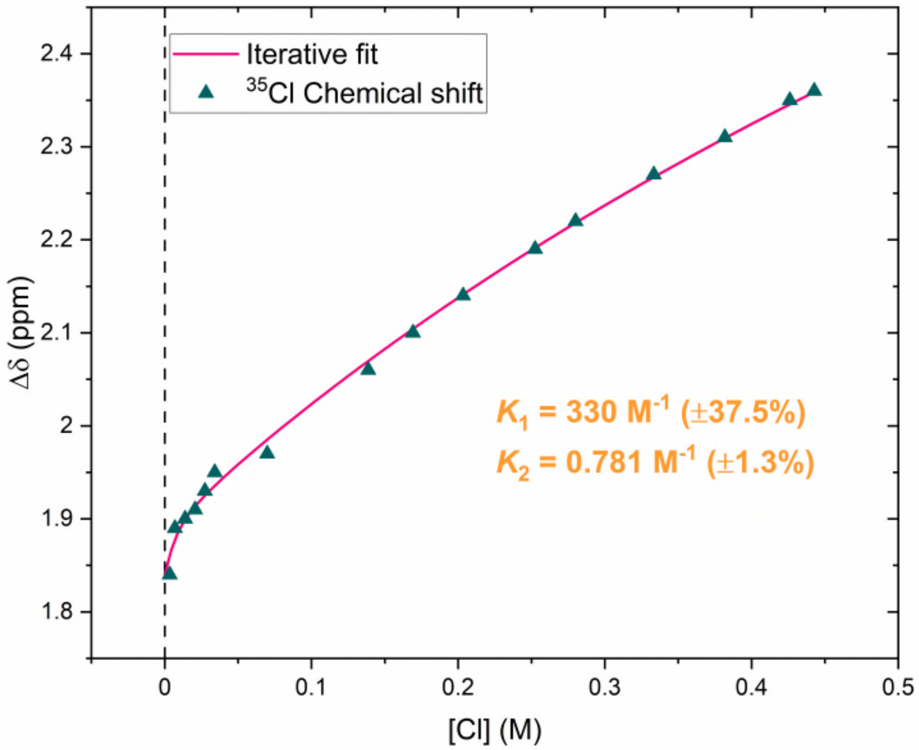

Figure S47: Binding isotherm for the binding of chloride to [Tb$_2$(**DO3A**)$_2$**C-3**], obtained by plotting the chloride chemical shift as a function of the concentration of KCl in D_2O at 304 K. 95% CI for K_1 is 160 – 980 M^{-1}, K_2 is 0.759 – 0.803 M^{-1}.

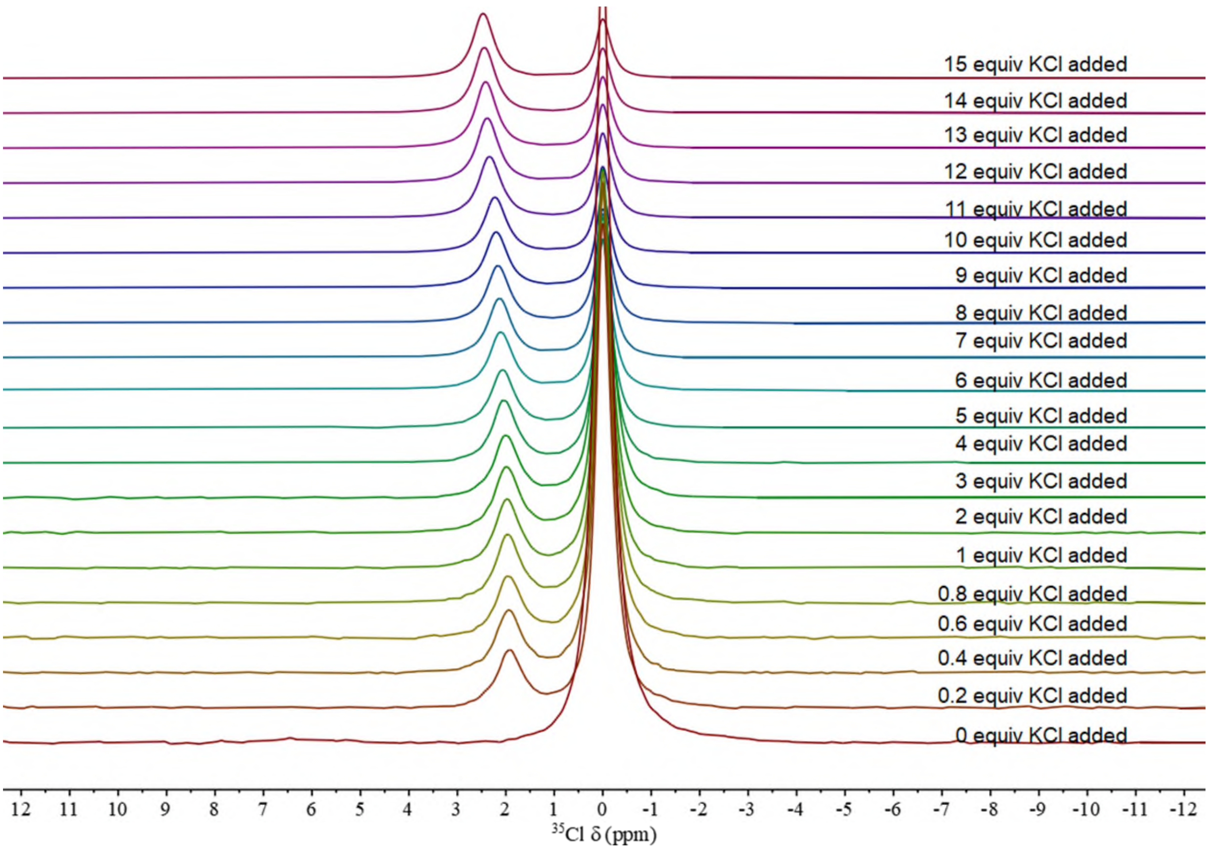

Figure S48: 49 MHz ^{35}Cl NMR titration spectra of 0.035 M [Tb_2(**DO3A**)$_2$**C-3**] with increasing concentration of KCl (stock concentration = 1.4 M) in D_2O at 311 K. Spectra were recorded with a capillary tube insert containing saturated KCl in D_2O (non-dilution method used).

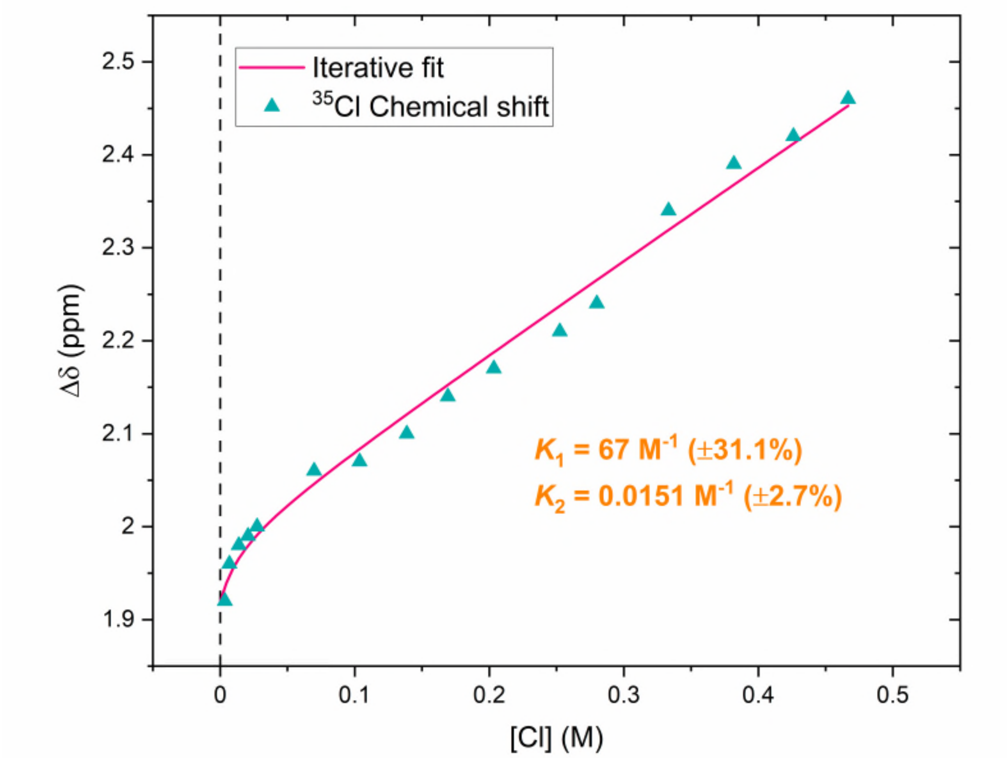

Figure S49: Binding isotherm for the binding of chloride to [Tb_2(**DO3A**)$_2$**C-3**], obtained by plotting the chloride chemical shift as a function of the concentration of KCl in D_2O at 311 K. 95% CI for K_1 is 34 – 150 M^{-1}, K_2 is 0.01426 – 0.01615 M^{-1}.

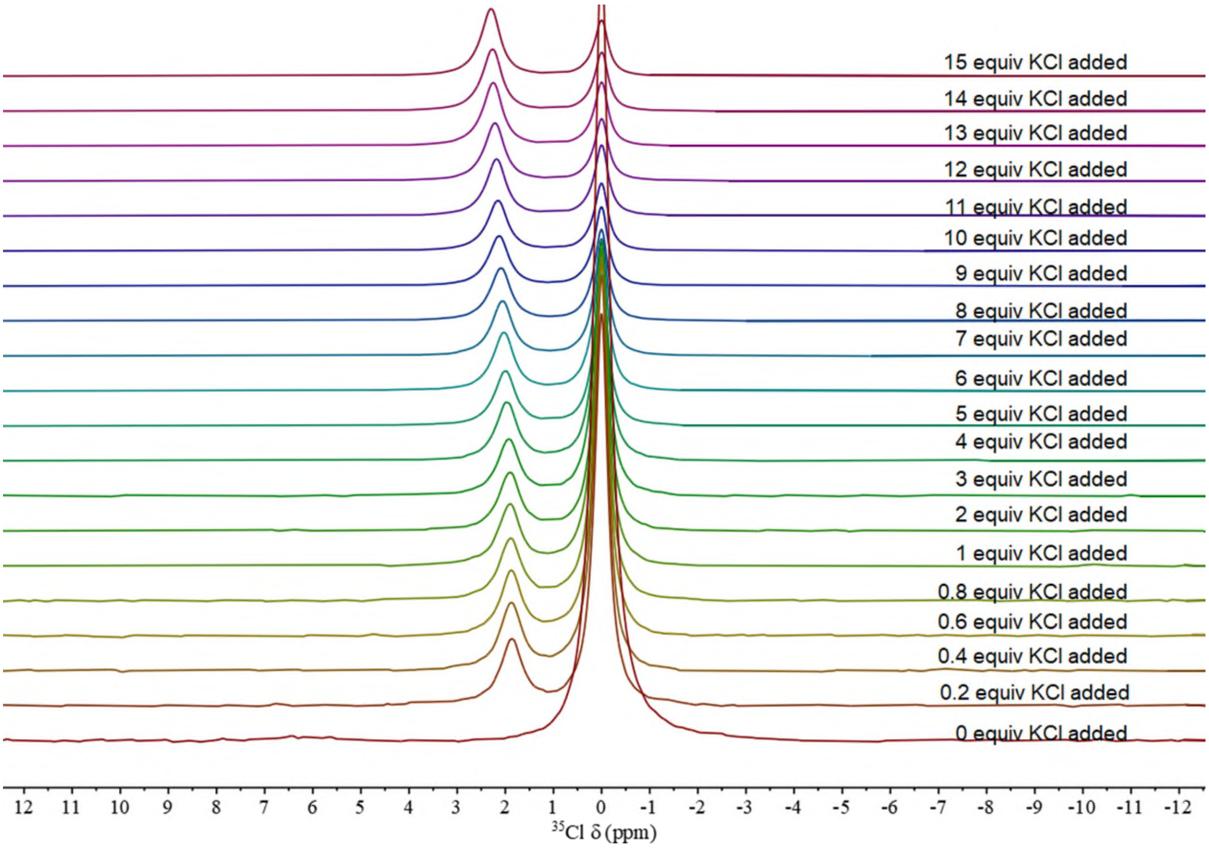

Figure S50: 49 MHz ^{35}Cl NMR titration spectra of 0.035 M [Tb_2(**DO3A**)$_2$**C-3**] with increasing concentration of KCl (stock concentration = 1.4 M) in D_2O at 317 K. Spectra were recorded with a capillary tube insert containing saturated KCl in D_2O (non-dilution method used).

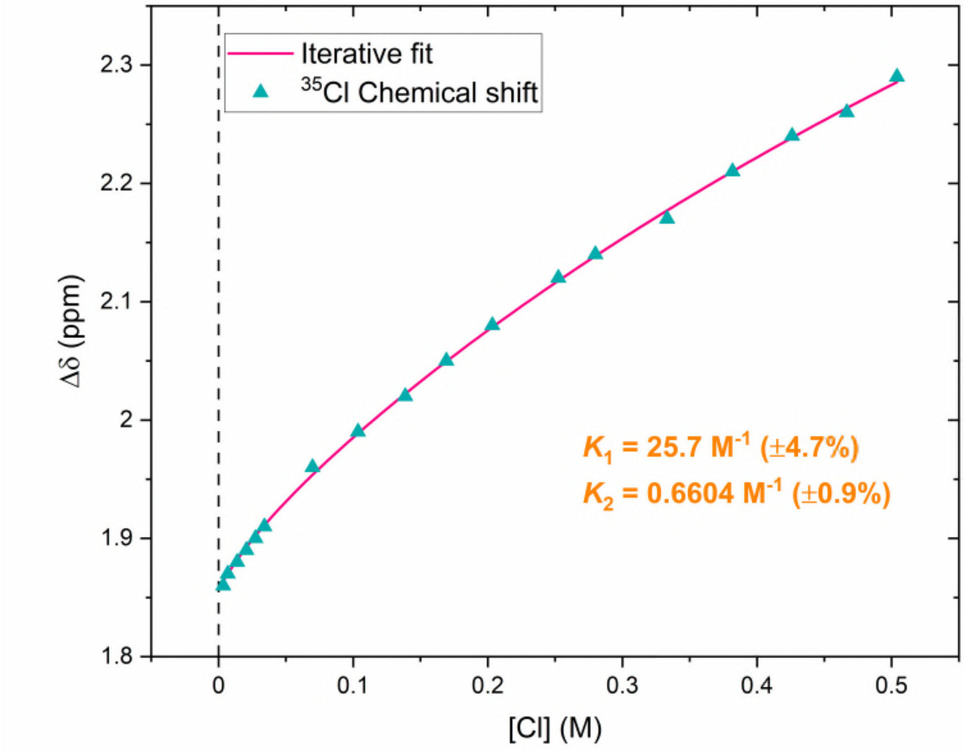

Figure S51: Binding isotherm for the binding of chloride to [Tb_2(**DO3A**)$_2$**C-3**], obtained by plotting the chloride chemical shift as a function of the concentration of KCl in D_2O at 317 K. 95% CI for K_1 is 23.3 – 28.4 M^{-1}, K_2 is 0.6486 – 0.6726 M^{-1}.

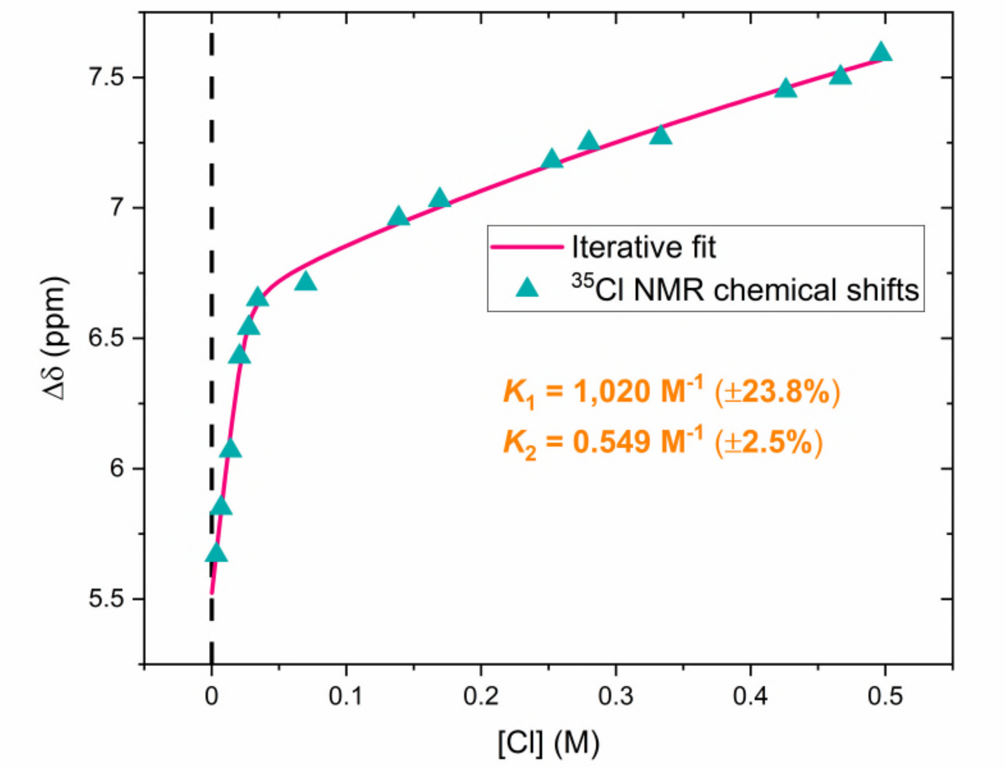

Figure S52: Binding isotherm for the binding of chloride to [Tb_2(**DO3A**)$_2$**C-3**], obtained by plotting the chloride chemical shift as a function of the concentration of KCl in D_2O at 298 K. 95% CI for K_1 is 646 – 1900 M^{-1}, K_2 is 0.52 – 0.579 M^{-1}. NMR titration spectra located in the manuscript (Figure 2b).

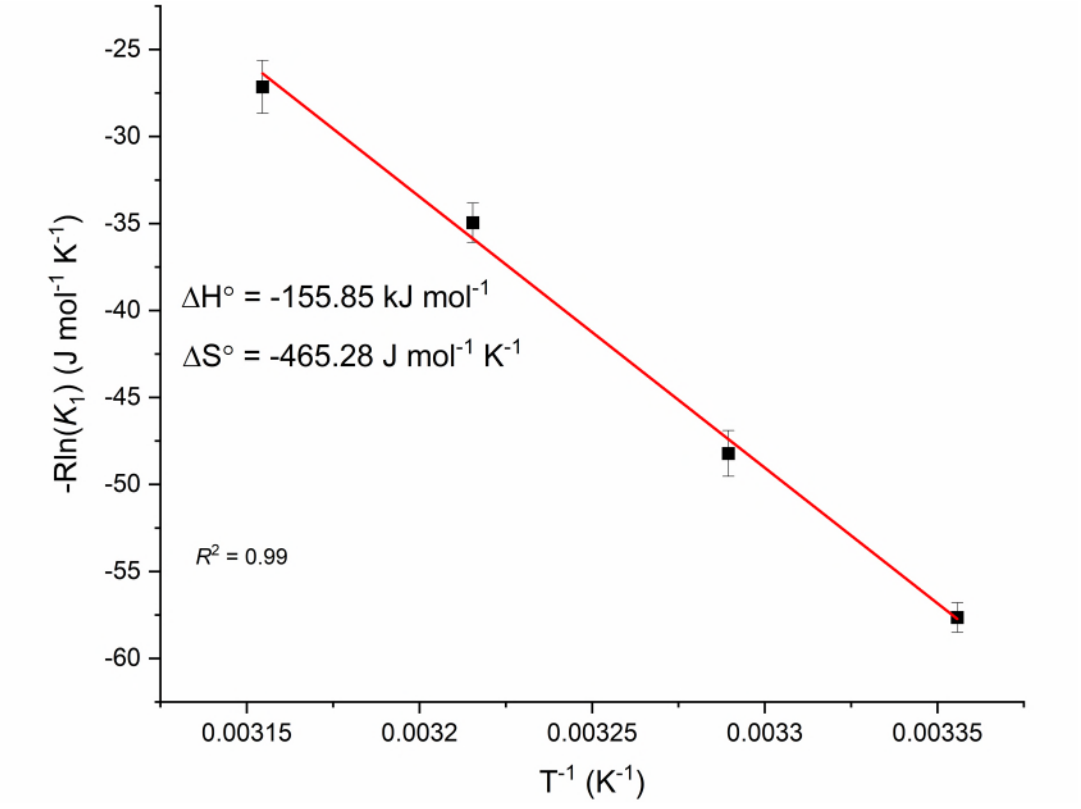

Figure S53: van't Hoff plot of chloride binding to [Tb_2(**DO3A**)$_2$**C-3**] obtained from binding constants originating in ^{35}Cl NMR titrations in D_2O at 298 K, 304 K, 311 K, and 317 K.

7. Mass Spectra of Eu(III) complexes bound to fluoride and chloride in water

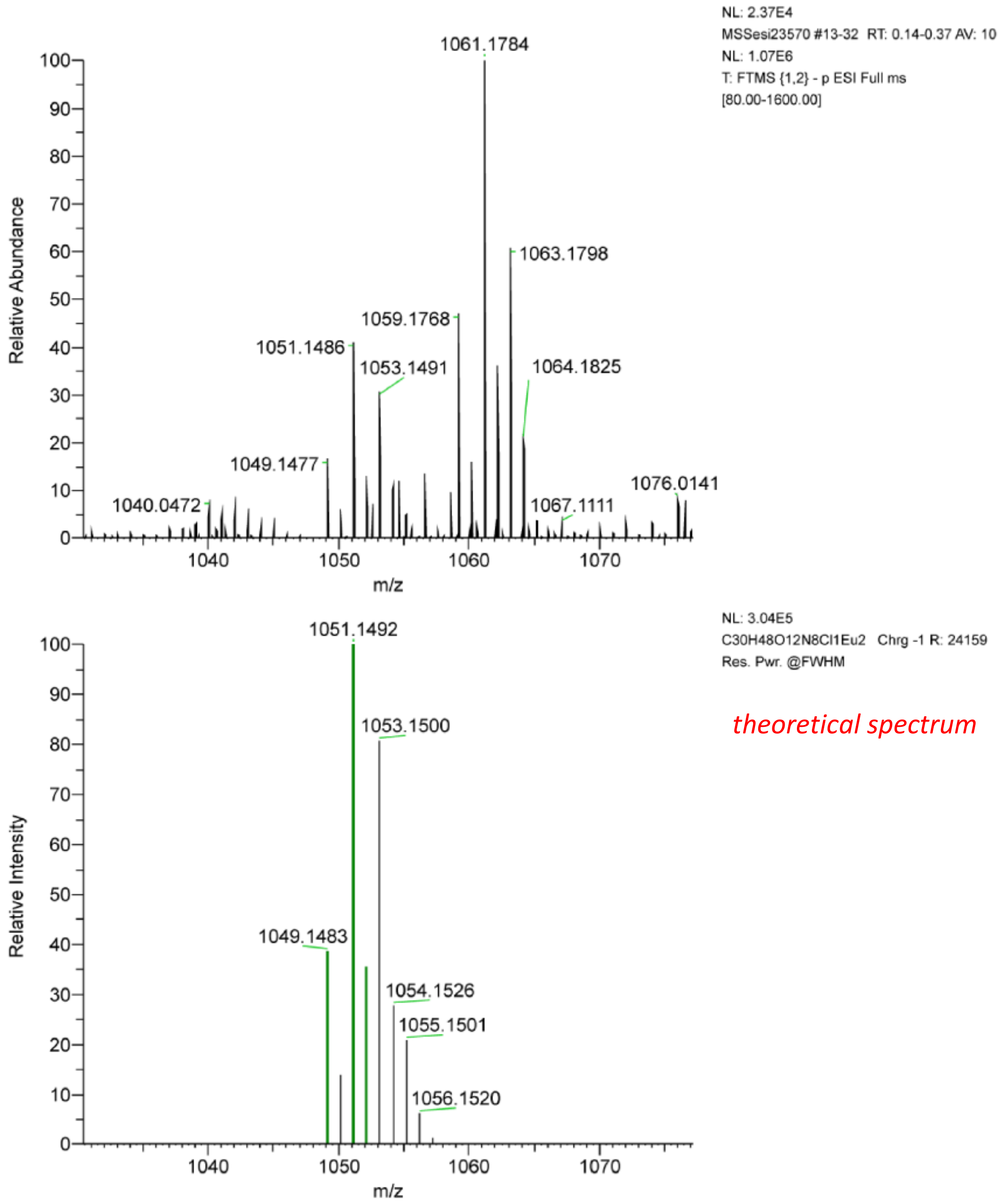

Figure S54: ESI-Mass spectrum of [Eu_2(**DO3A**)$_2$**C-2(**μ-Cl**)**]$^-$ in deionised H_2O, experimental spectrum (*top*) and theoretical spectrum (*bottom*).

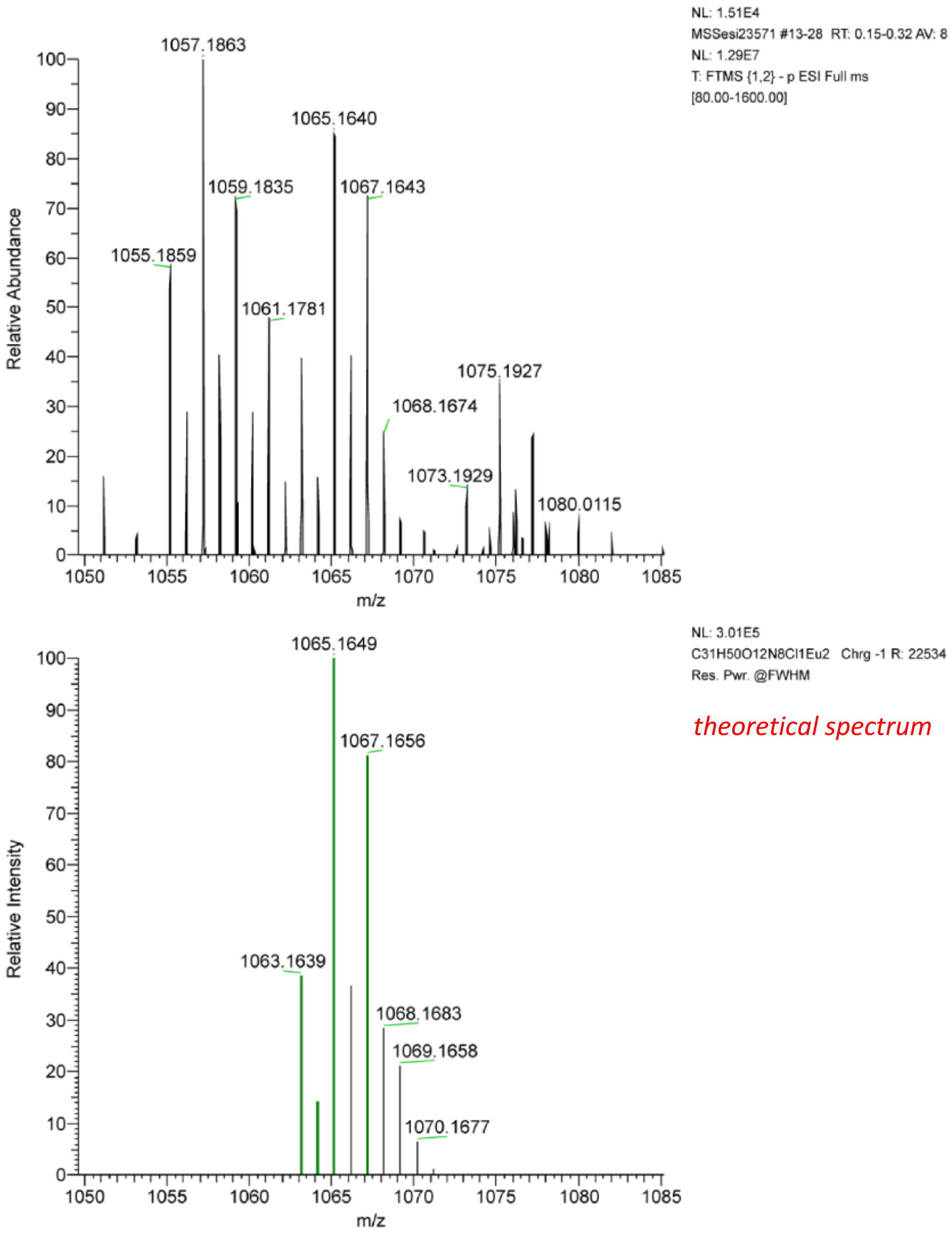

Figure S55: ESI-Mass spectrum of [Eu_2(**DO3A**)$_2$**C-3**(μ-Cl)]$^-$ recorded in deionised H_2O, experimental spectrum (*top*) and theoretical spectrum (*bottom*).

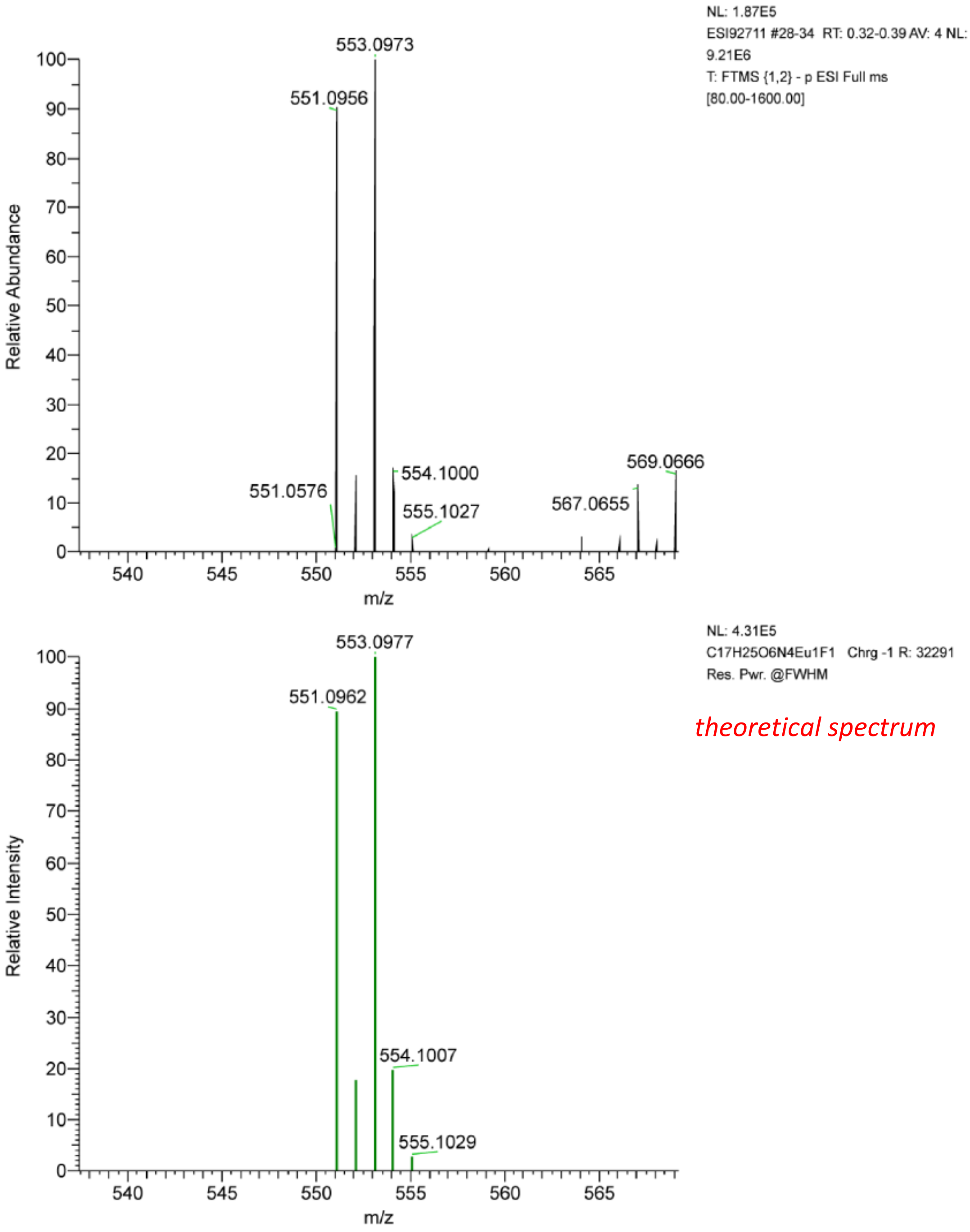

Figure S56: ESI-Mass spectrum of [Eu(**pDO3A**)F]$^-$ recorded in deionised H_2O, experimental spectrum (*top*) and theoretical spectrum (*bottom*).

8. Photophysical studies of mono and binuclear Eu(III) Complexes

8.1 Electronic absorption spectra in water

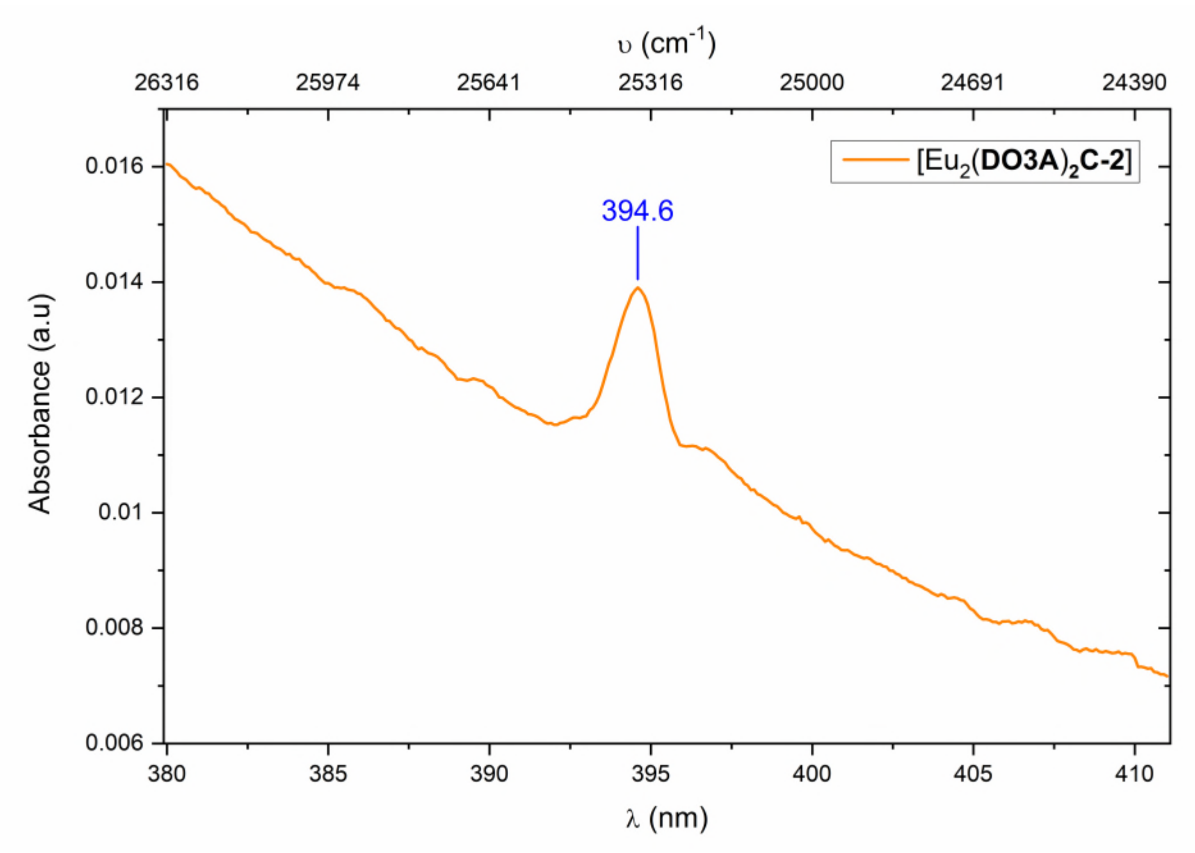

Figure S57: Electronic absorption spectrum of 5 mM [Eu_2(**DO3A**)$_2$**C-2**] in water at 22 °C.

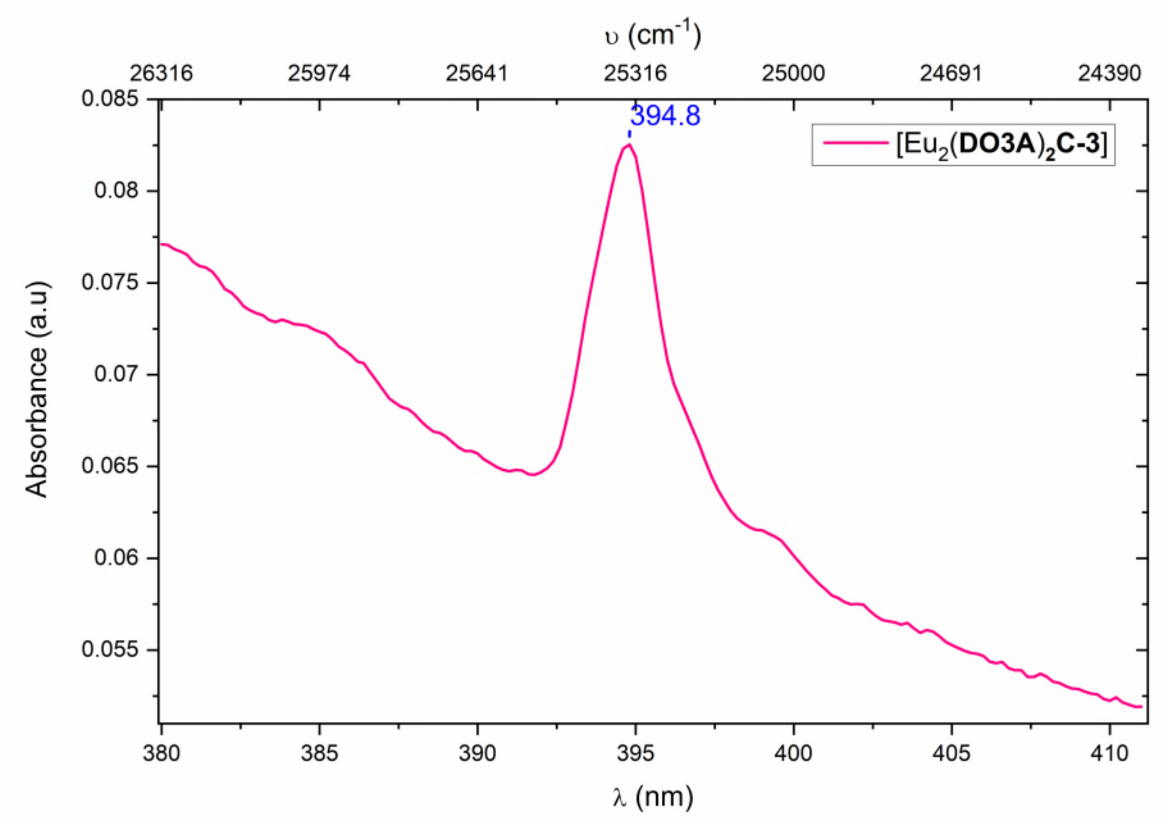

Figure S58: Electronic absorption spectrum of 5 mM [Eu_2(**DO3A**)$_2$**C-3**] in water at 22 °C.

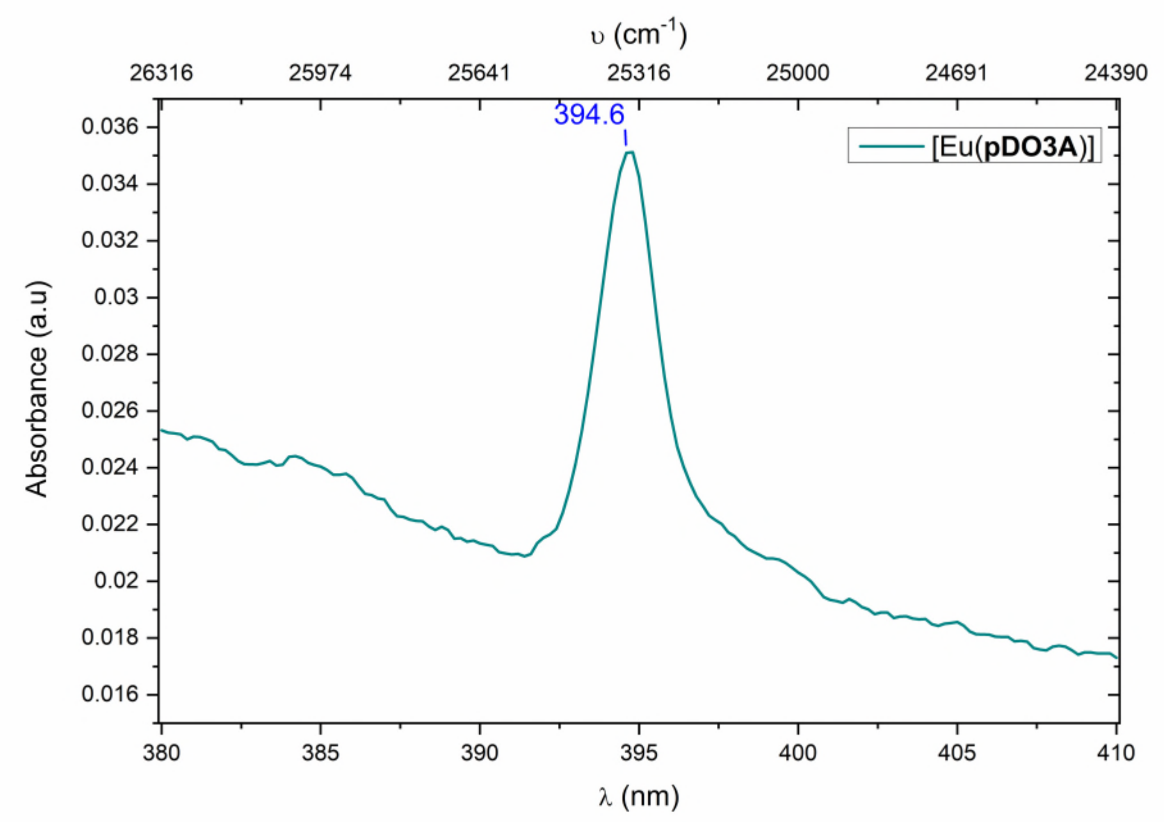

Figure S59: Electronic absorption spectrum of 5 mM [Eu(**pDO3A**)] in water at 22 °C.

8.2 Excitation and emission spectra

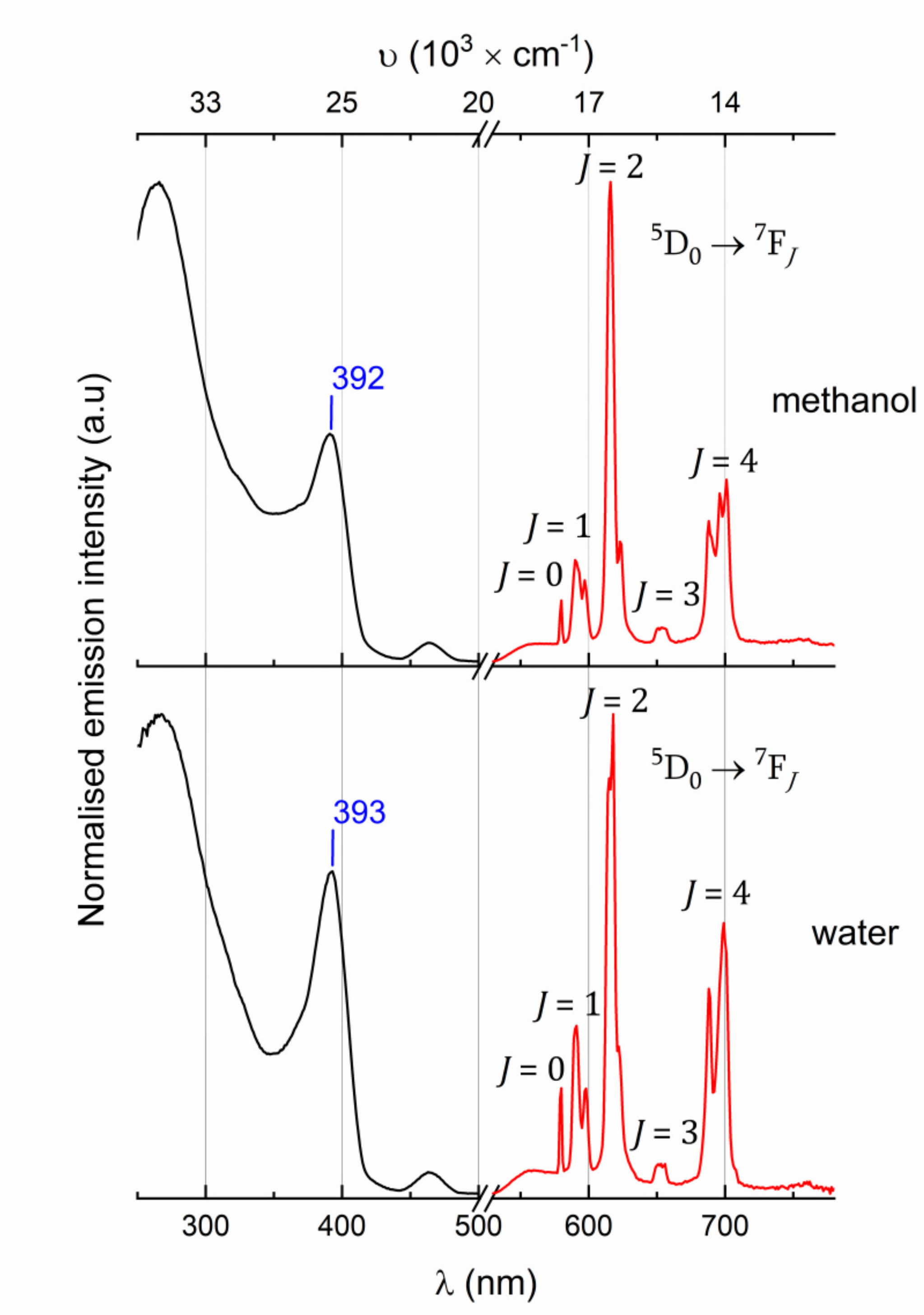

Figure S60: Excitation spectra (*left*) and steady-state emission spectra (*right*) of 1 mM [Eu_2(**DO3A**)$_2$**C-2**] in water and methanol (λ_{max} of the excitation spectra highlighted in blue) at 22 °C.

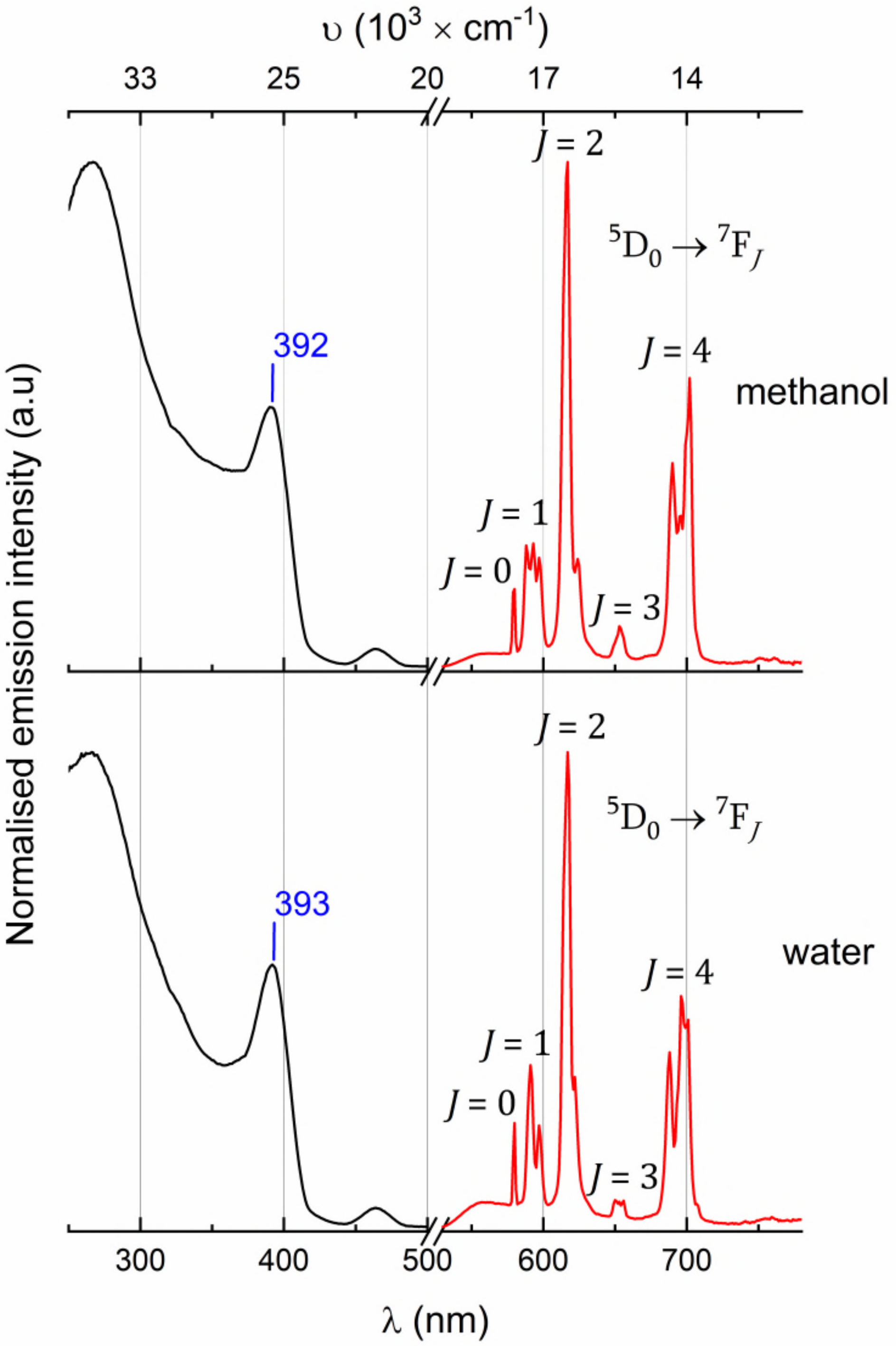

Figure S61: Excitation spectra (*left*) and steady-state emission spectra (*right*) of 1 mM [Eu_2(**DO3A**)$_2$**C-3**] in water and methanol (λ_{max} of the excitation spectra highlighted in blue) at 22 °C.

8.3 Steady-state emission spectra at different pH and pD

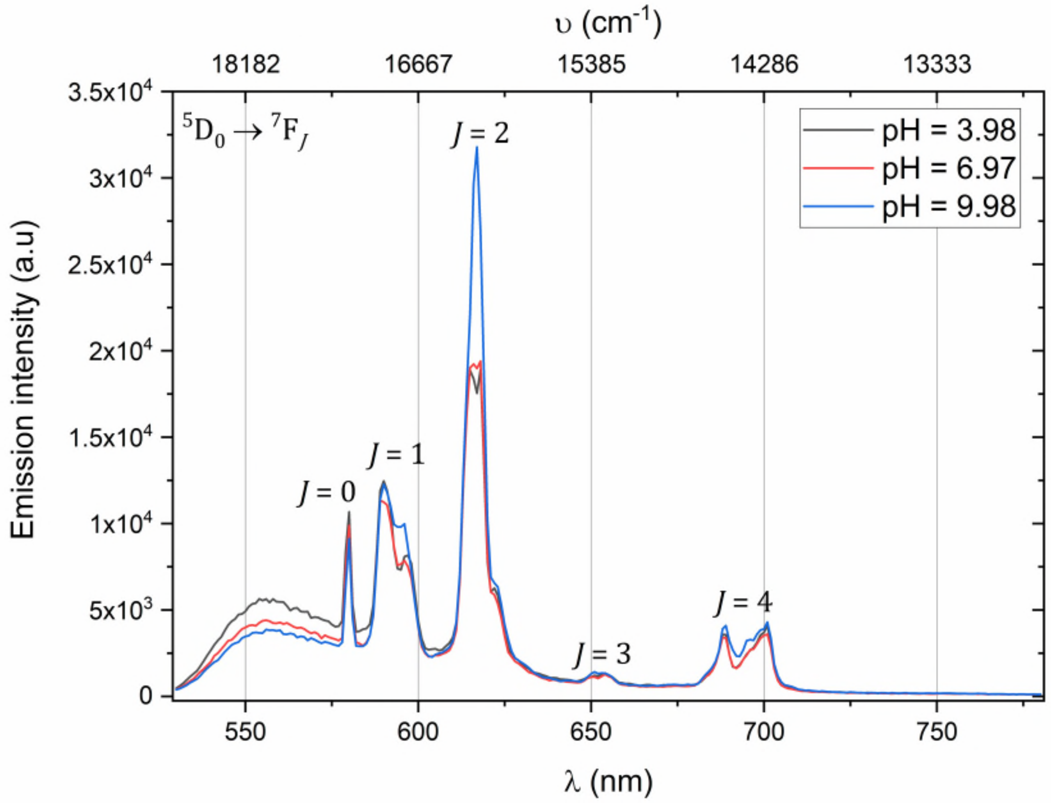

Figure S62: Steady-state emission spectra of 1 mM [Eu_2(**DO3A**)$_2$**C-2**] (λ_{ex} = 393 nm) at different pH (22 °C).

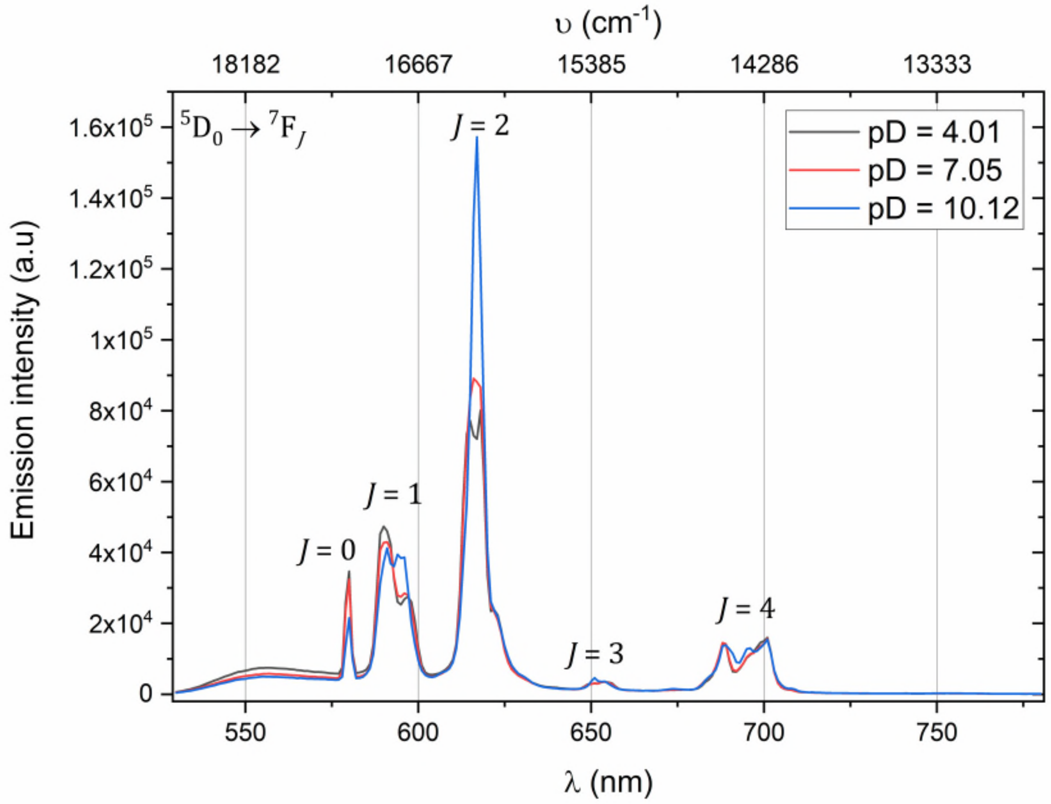

Figure S63: Steady-state emission spectra of 1 mM [Eu_2(**DO3A**)$_2$**C-2**] (λ_{ex} = 393 nm) at different pD (22 °C).

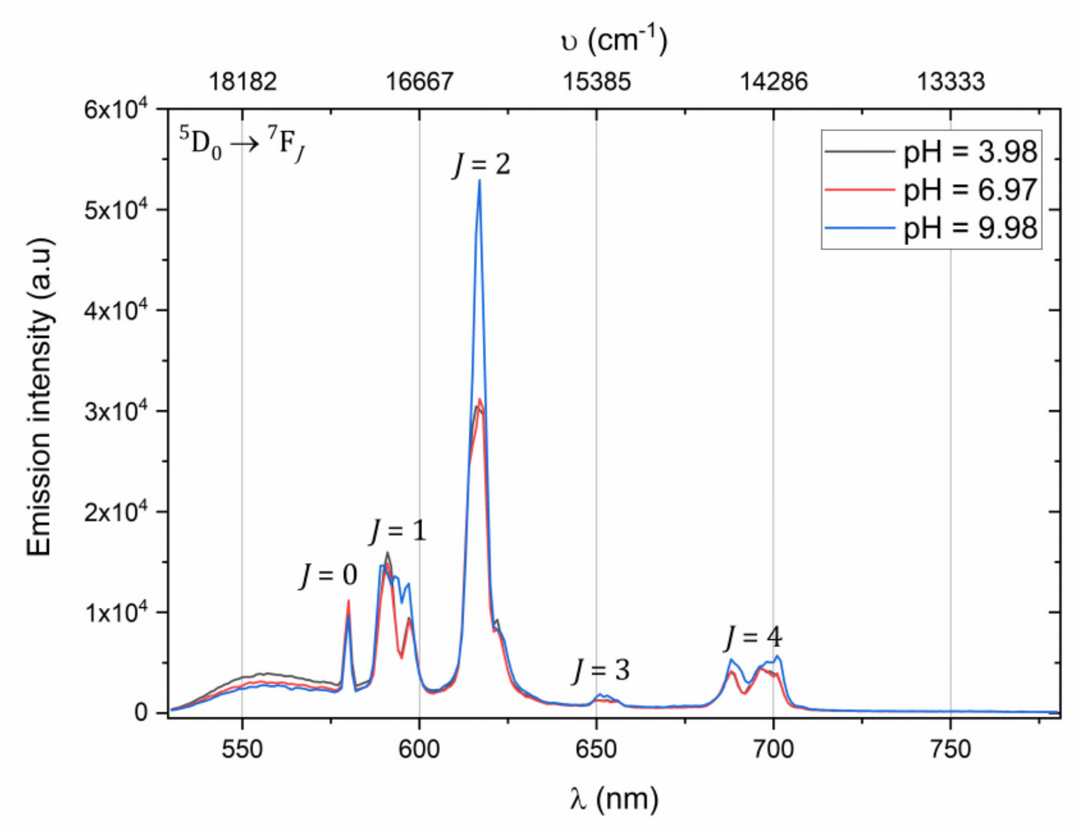

Figure S64: Steady-state emission spectra of 1 mM [Eu_2(**DO3A**)$_2$**C-3**] (λ_{ex} = 393 nm) at different pH (22 °C).

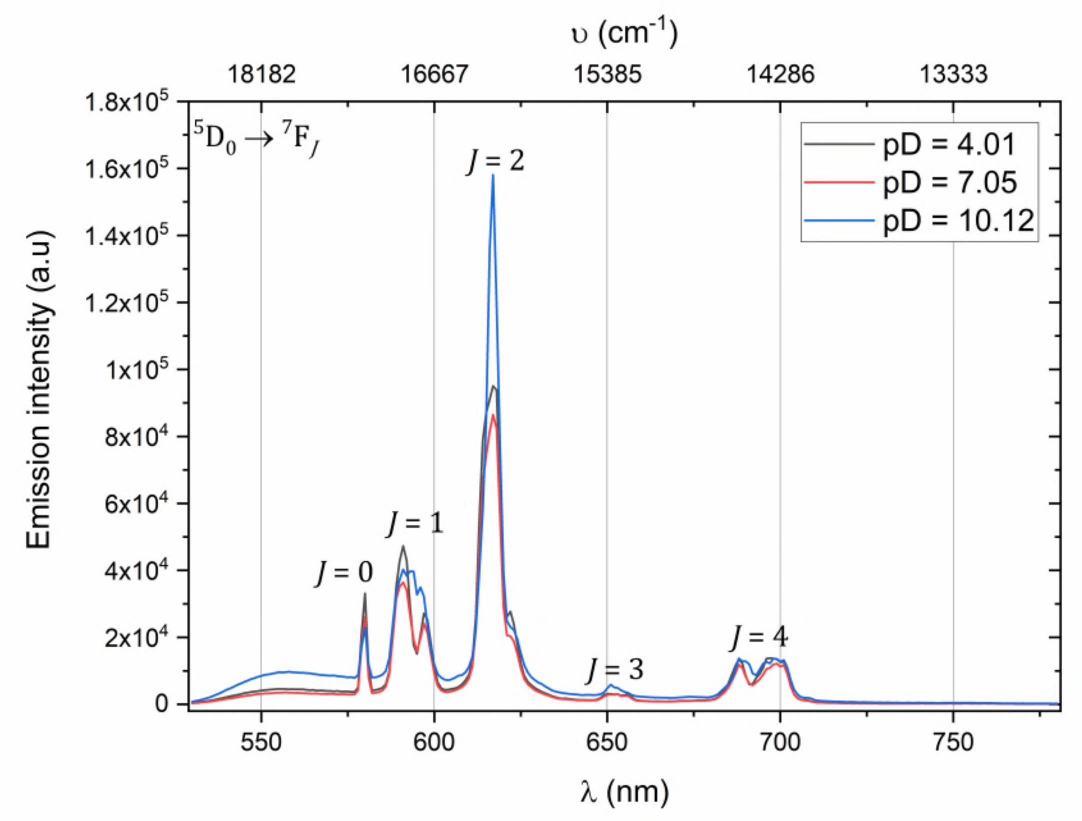

Figure S65: Steady-state emission spectra of 1 mM [Eu_2(**DO3A**)$_2$**C-3**] (λ_{ex} = 393 nm) at different pD (22 °C).

8.4. Luminescence Titration with Halides: Spectra, Binding Isotherms, and Speciation Plots

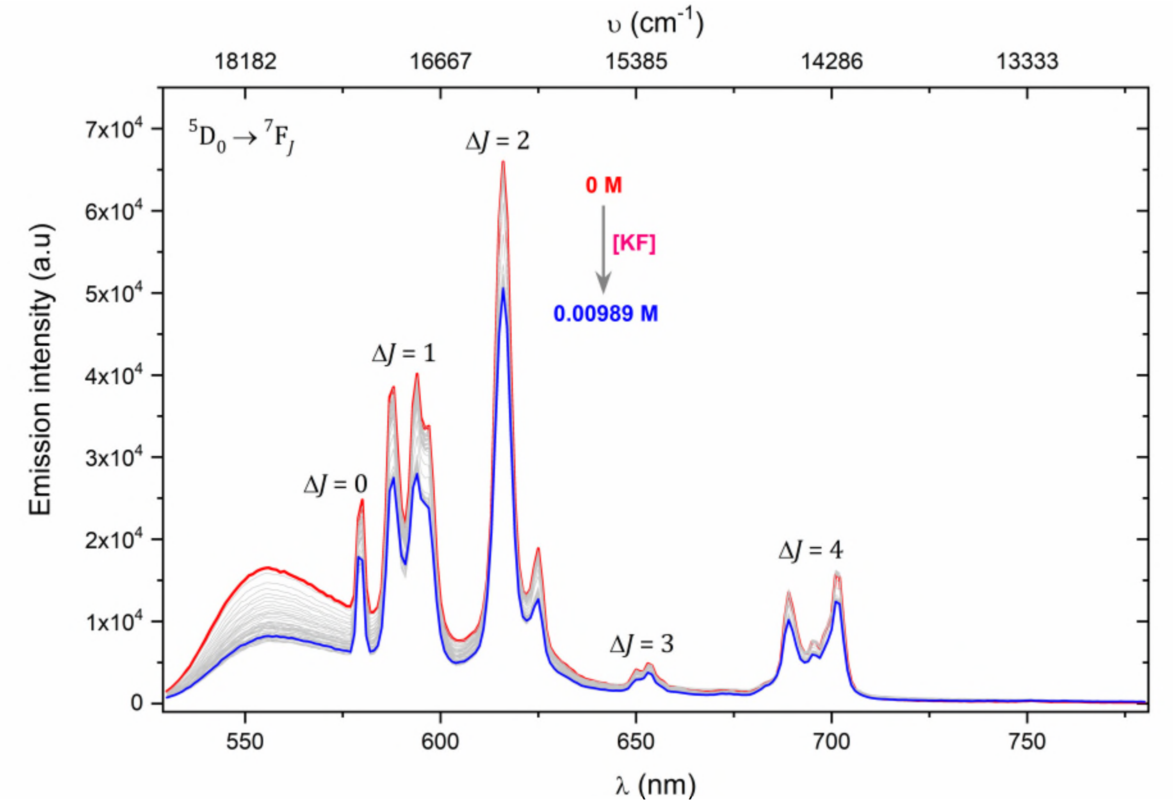

Figure S66: Steady-state luminescence titration spectra of 1 mM [Eu_2(**DO3A**)$_2$**C-2**] (λ_{ex} = 393 nm) against KF (stock concentration = 0.02 M) in 0.01 M PBS (pH 7.4) at 22 °C; spectrum of neat complex solution (*red*), spectra upon the additions of KF (*grey*), spectrum upon the final addition of KF (*blue*) (non-dilution method used).

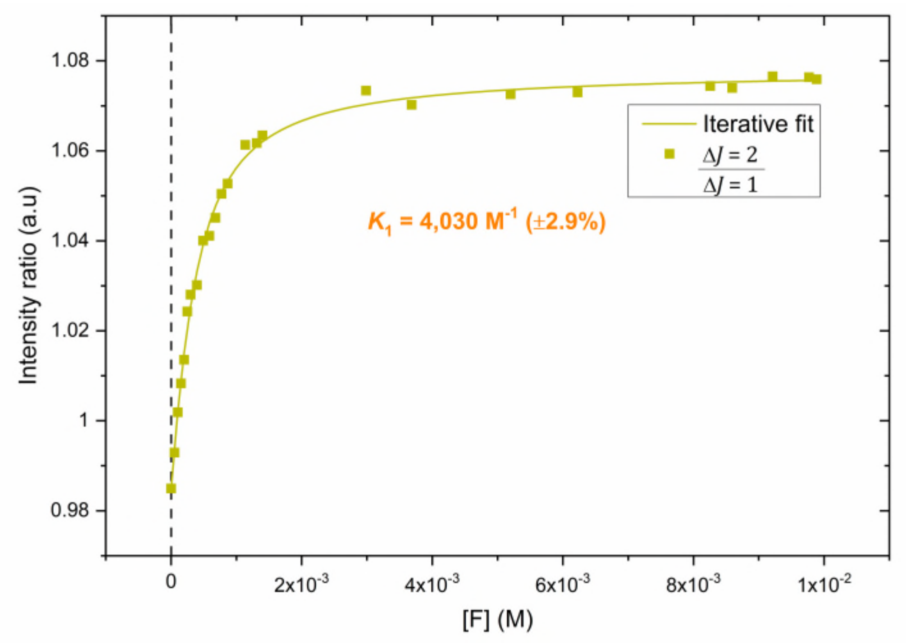

Figure S67: Binding isotherm for the binding of fluoride to [Eu_2(**DO3A**)$_2$**C-2**], obtained by plotting the ratio of emission bands $\Delta J = 2/\Delta J = 1$ as a function of the concentration of KF in 0.01 M PBS (pH 7.4) at 22 °C. 95% CI for K_1 is 3,800 – 4,290 M^{-1}.

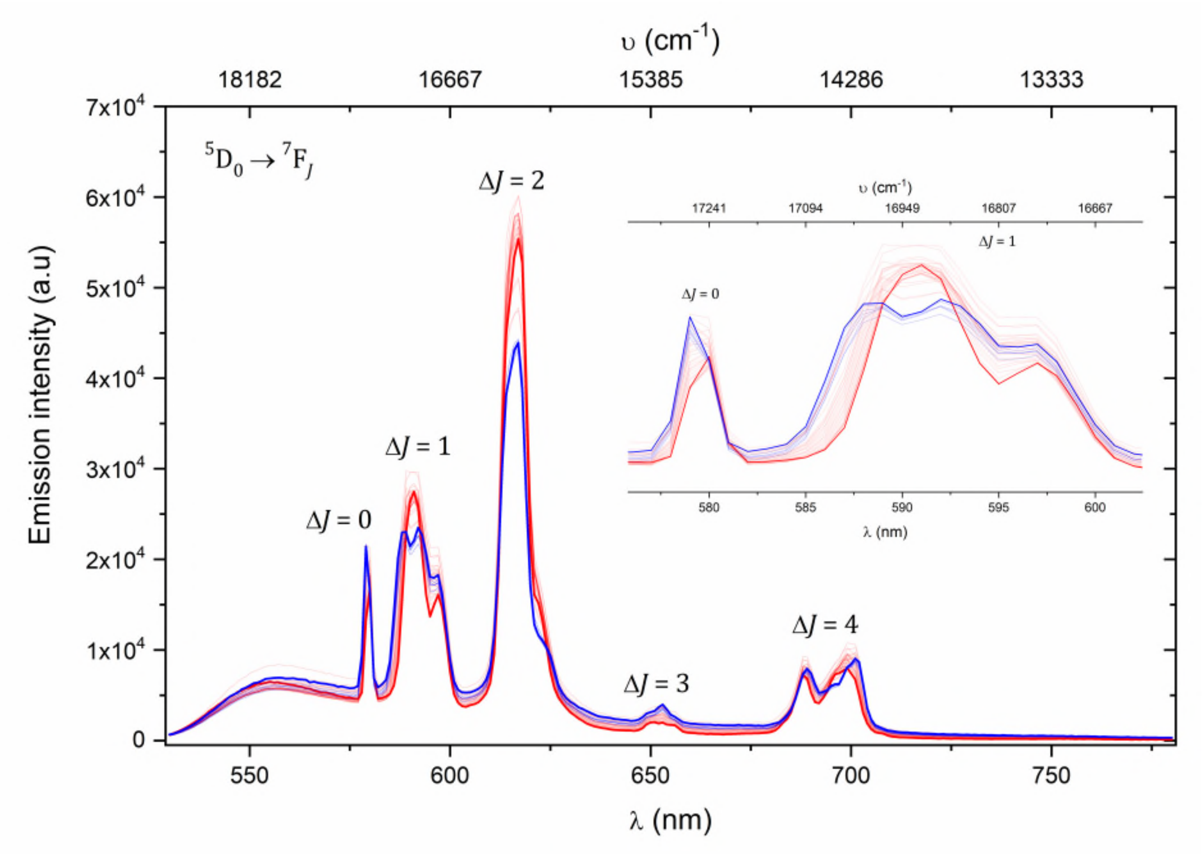

Figure S68: Steady-state luminescence titration spectra of 1 mM [Eu_2(**DO3A**)$_2$**C-2**] (λ_{ex} = 393 nm) against KF (stock concentration = 0.02 M) in 0.01 M Tris-HCl buffer (pH 7.4) at 22°C; spectrum of neat complex solution (***red in bold***), spectra upon the additions of KF (*red to blue*), spectrum upon the final addition of KF (***blue in bold***) (non-dilution method used). The inset expands the Δ*J* = *0* and Δ*J* = 1 emission bands.

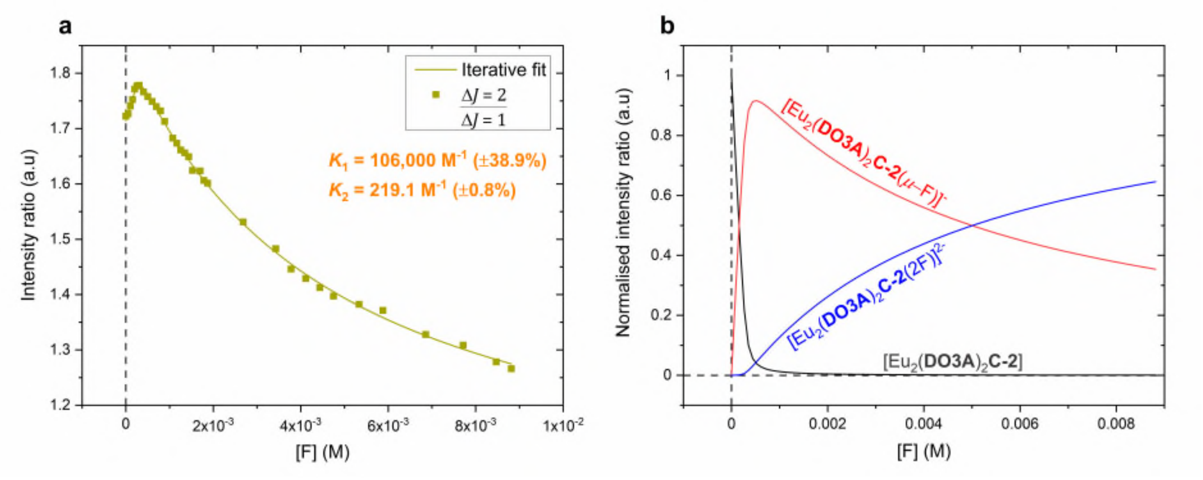

Figure S69: (a) Binding isotherm for the binding of fluoride to [Eu_2(**DO3A**)$_2$**C-2**], obtained by plotting the ratio of emission bands Δ*J* = 2/Δ*J* = 1 as a function of the concentration of KF in 0.01 M Tris-HCl buffer (pH 7.4) at 22°C. 95% CI for K_1 is 53,700 – 299,000 M^{-1}; K_2 is 215.7 – 222.7 M^{-1}. **(b)** Normalised model of speciation for the binding of fluoride to [Eu_2(**DO3A**)$_2$**C-2**], obtained by plotting the ratio of emission bands Δ*J* = 2/Δ*J* = 1 as a function of the concentration of KF in 0.01 M Tris-HCl buffer (pH 7.4) at 22°C.

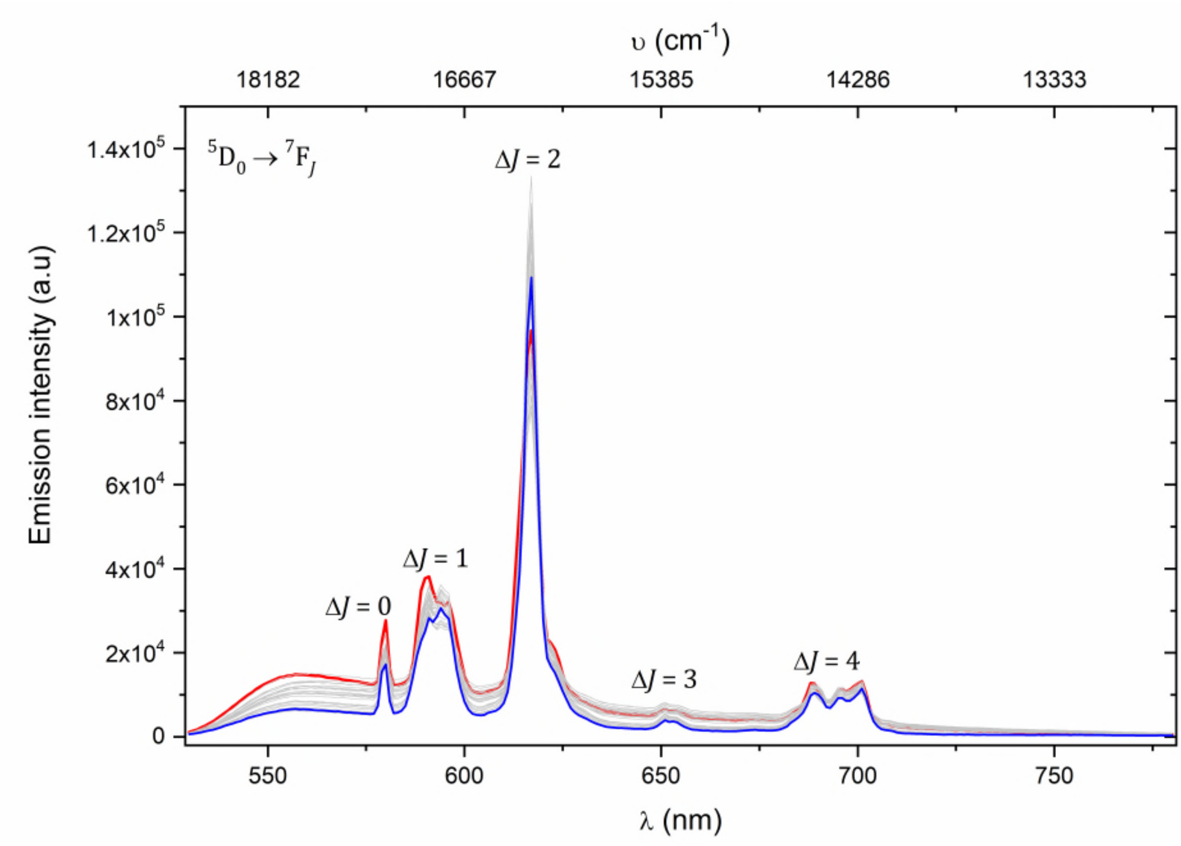

Figure S70: Steady-state luminescence titration spectra of 1 mM [Eu_2(**DO3A**)$_2$**C-2**] (λ_{ex} = 393 nm) against KF (stock concentration = 0.02 M) in 0.01 M CHES buffer (pH 9.98) at 22 °C; spectrum of neat complex solution (*red*), spectra upon the additions of KF (*grey*), spectrum upon the final addition of KF (*blue*) (non-dilution method used).

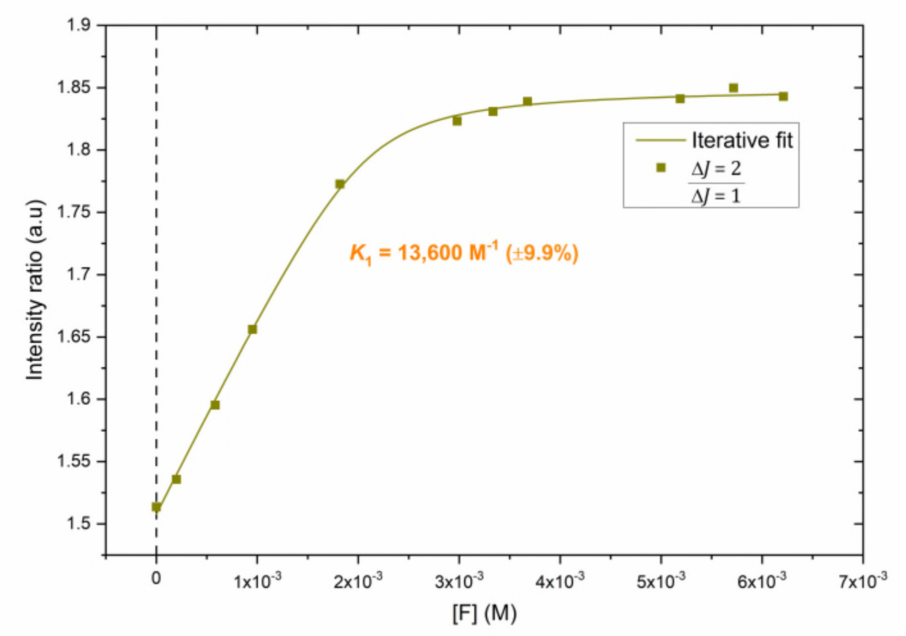

Figure S71: Binding isotherm for the binding of fluoride to [Eu_2(**DO3A**)$_2$**C-2**], obtained by plotting the ratio of emission bands $\Delta J = 2/\Delta J = 1$ as a function of the concentration of KF in 0.01 M CHES buffer (pH 9.98) at 22 °C. 95% CI for K_1 is 11,000 – 17,200 M^{-1}.

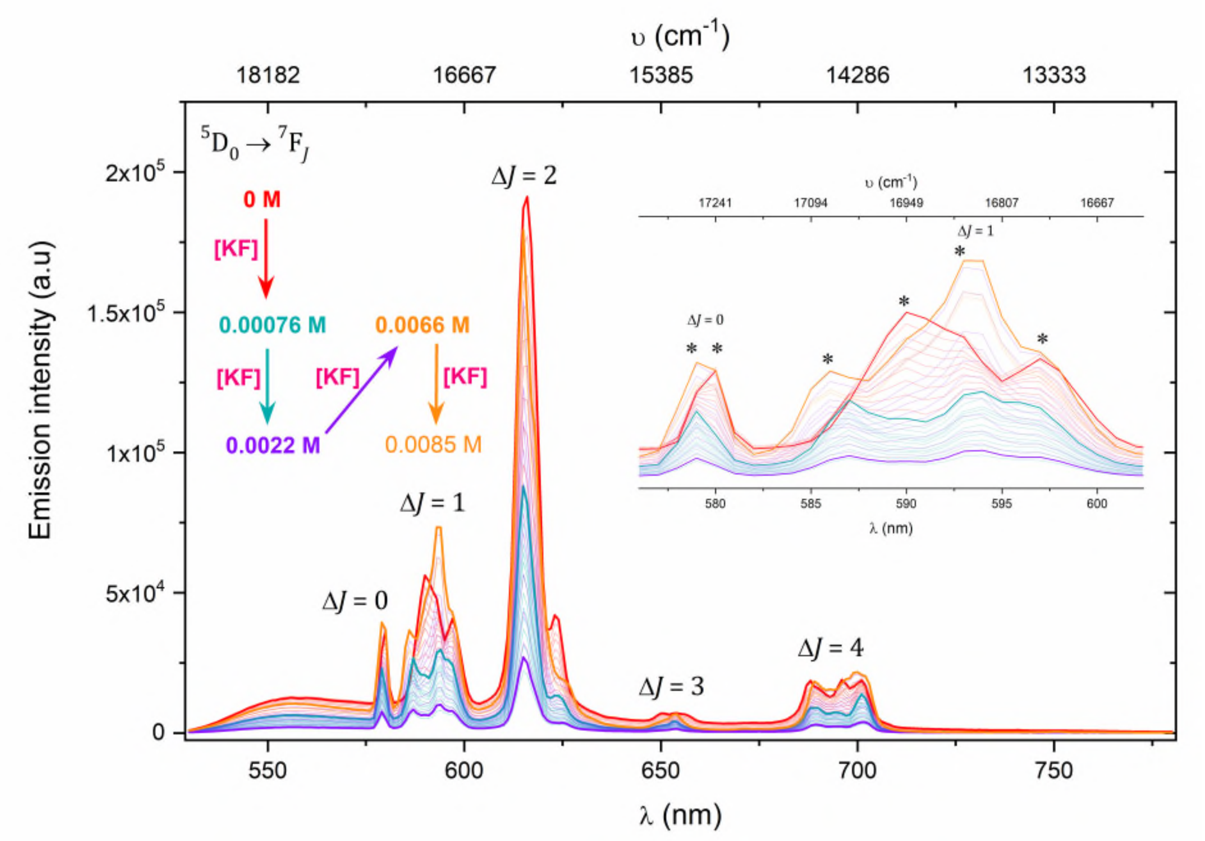

Figure S72: Steady-state luminescence titration spectra of 1 mM [Eu_2(**DO3A**)$_2$**C-2**] (λ_{ex} = 393 nm) against KF (stock concentration = 0.02 M) in methanol at 22 °C; spectrum of neat complex solution (***red in bold***), spectra upon the additions of KF (*red*, ***cyan in bold***, *cyan*, ***violet in bold***, *violet*, ***orange in bold*** *and orange*), spectrum upon the final addition of KF (*orange*) (non-dilution method used). The inset expands the ΔJ = *0* and ΔJ = 1 emission bands and the asterisks highlight the emission maxima used in quantifying binding of fluoride to the complex.

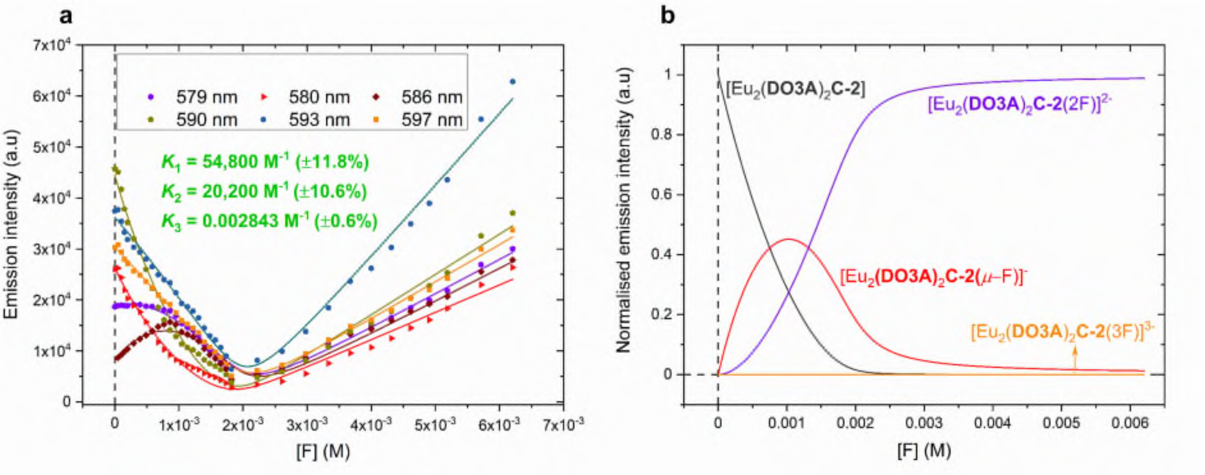

Figure S73: (a) Binding isotherm for the binding of fluoride to [Eu_2(**DO3A**)$_2$**C-2**], obtained by plotting the intensity of the emission maxima of the ΔJ = *0* and ΔJ = *1* emission bands as a function of the concentration of KF in methanol. 95% CI for K_1 is 43,500 – 70,800 M^{-1}; K_2 is 16,500 – 25,400 M^{-1}; K_3 is 0.002807 – 0.002879 M^{-1}. All solid lines represent the iterative fit. **(b)** Normalised model of speciation for the binding of fluoride to [Eu_2(**DO3A**)$_2$**C-2**], obtained by plotting the intensity of the 586 nm emission as a function of the concentration of KF in methanol at 22 °C.

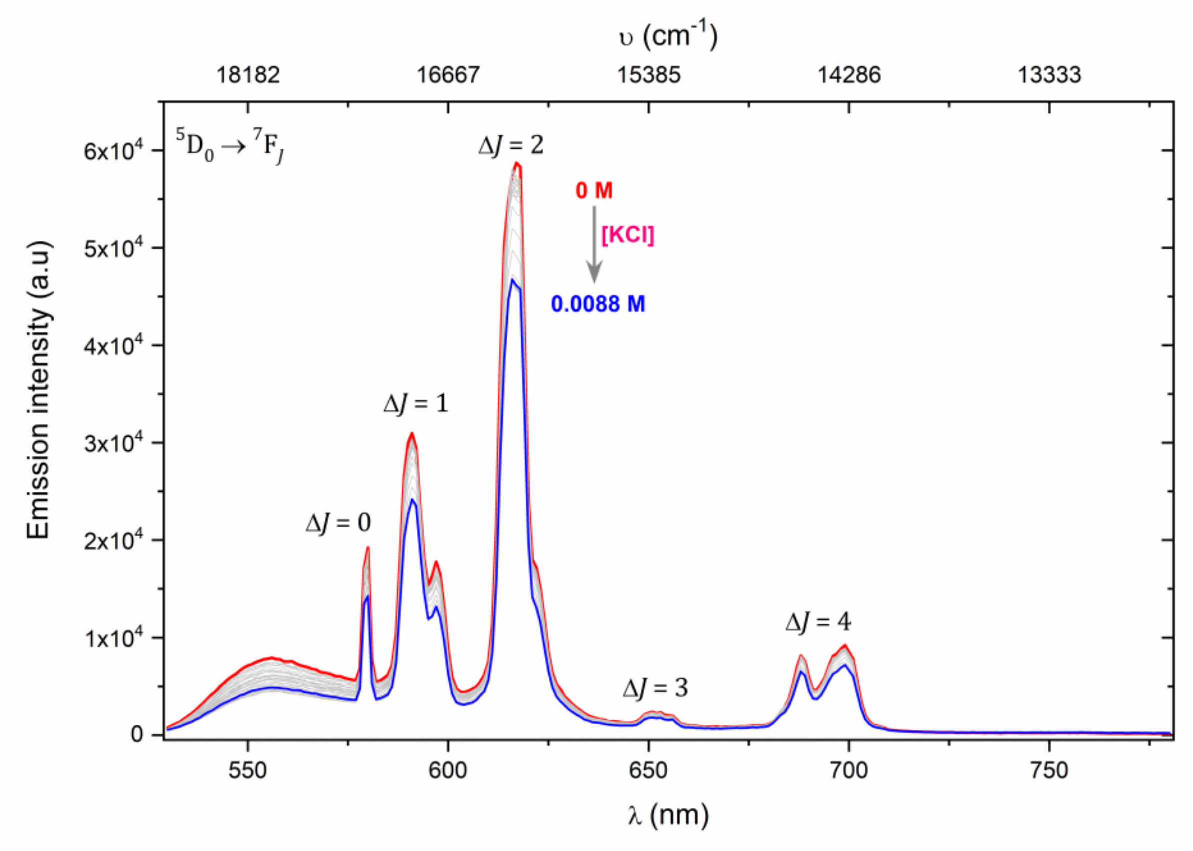

Figure S74: Steady-state luminescence titration spectra of 1 mM [Eu_2(**DO3A**)$_2$**C-2**] (λ_{ex} = 393 nm) against KCl (stock concentration = 0.02 M) in deionised water at 22 °C; spectrum of neat complex solution (*red*), spectra upon the additions of KCl (*grey*), spectrum upon the final addition of KCl (*blue*) (non-dilution method used).

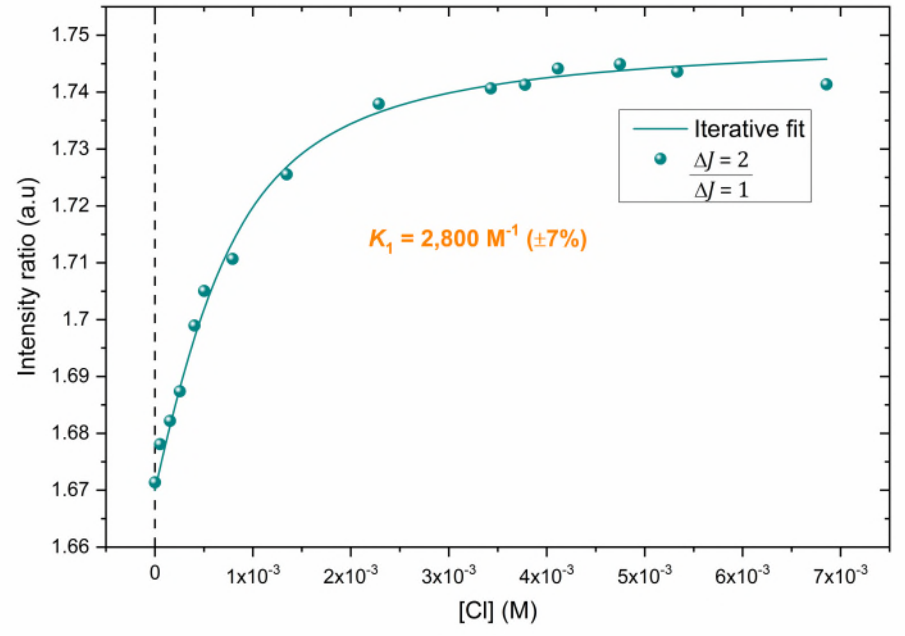

Figure S75: Binding isotherm for the binding of chloride to [Eu_2(**DO3A**)$_2$**C-2**], obtained by plotting the ratio of emission bands $\Delta J = 2/\Delta J = 1$ as a function of the concentration of KCl in deionised water at 22 °C. 95% CI for K_1 is 2,420 – 3,270 M^{-1}.

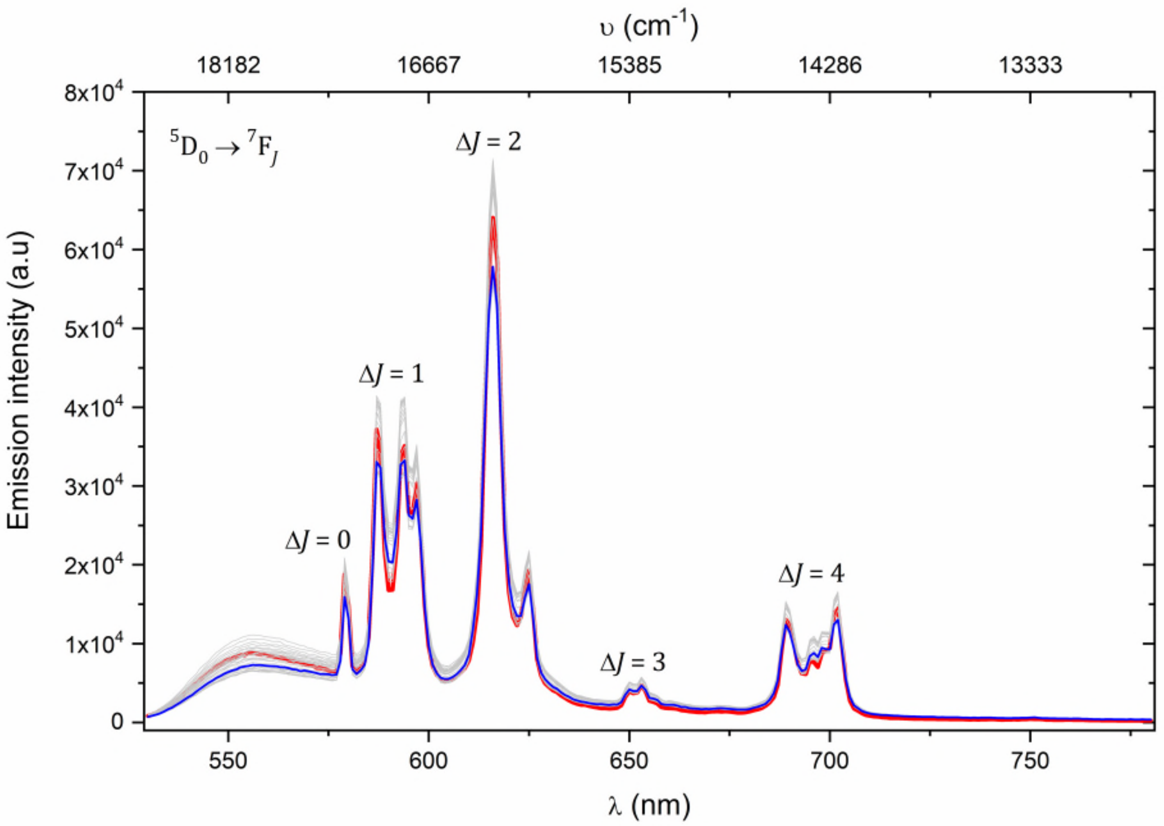

Figure S76: Steady-state luminescence titration spectra of 1 mM [Eu_2(**DO3A**)$_2$**C-2**] (λ_{ex} = 393 nm) against KCl (stock concentration = 0.02 M) in 0.01 M phosphate buffer (pH 7.4) at 22 °C; spectrum of neat complex solution (*red*), spectra upon the additions of KCl (*grey*), spectrum upon the final addition of KCl (*blue*) (non-dilution method used).

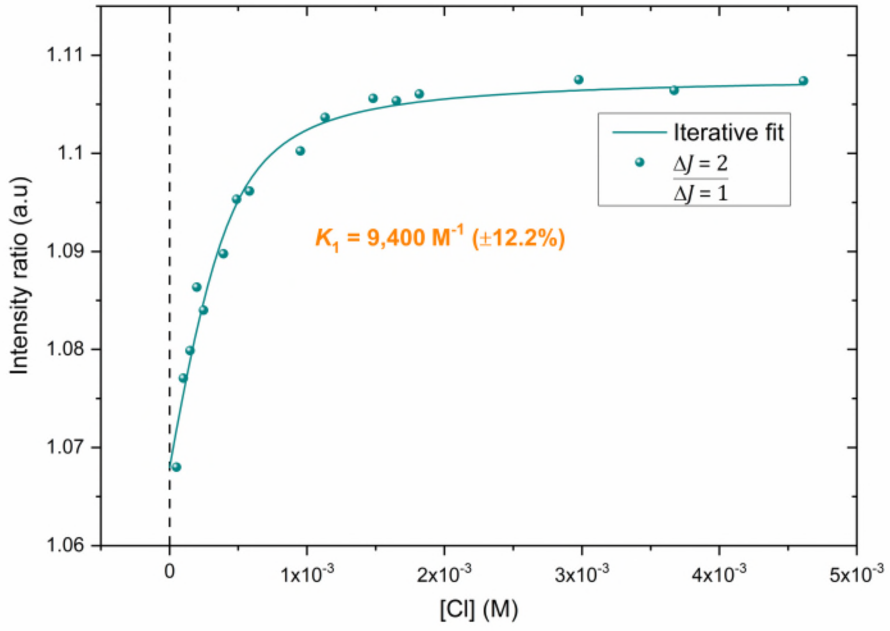

Figure S77: Binding isotherm for the binding of chloride to [Eu_2(**DO3A**)$_2$**C-2**], obtained by plotting the ratio of emission bands $\Delta J = 2/\Delta J = 1$ as a function of the concentration of KCl in 0.01 M phosphate buffer (pH 7.4) at 22 °C. 95% CI for K_1 is 7,300 – 12,000 M^{-1}.

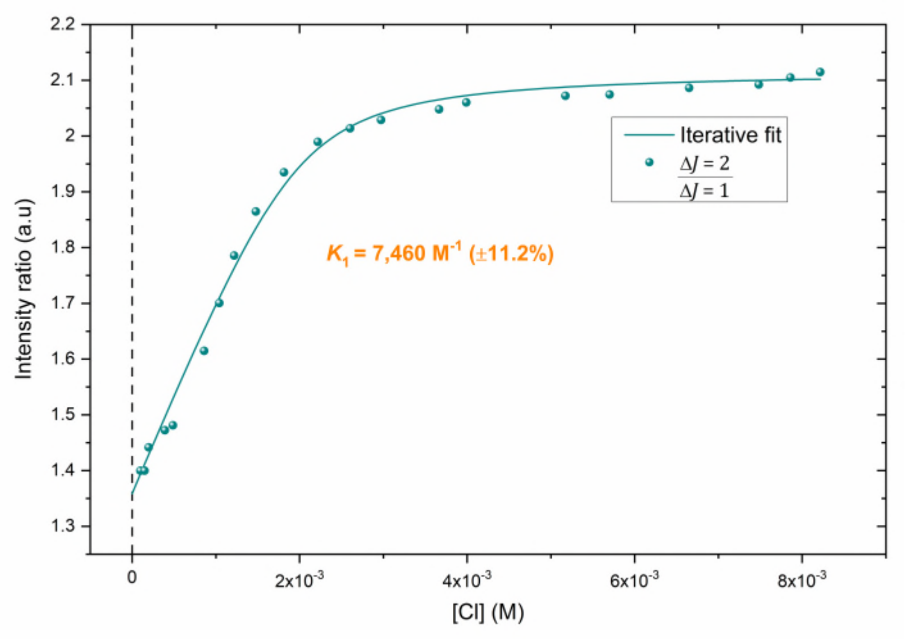

Figure S78: Binding isotherm for the binding of chloride to [Eu2(**DO3A**)2**C-2**], obtained by plotting the ratio of emission bands $\Delta J = 2/\Delta J = 1$ as a function of the concentration of KCl in 0.01 M CHES buffer (pH 9.98) at 22 °C. 95% CI for K_1 is 5,960 – 9,640 M^{-1}. Steady-state titration spectra located in the manuscript (Figure 2c).

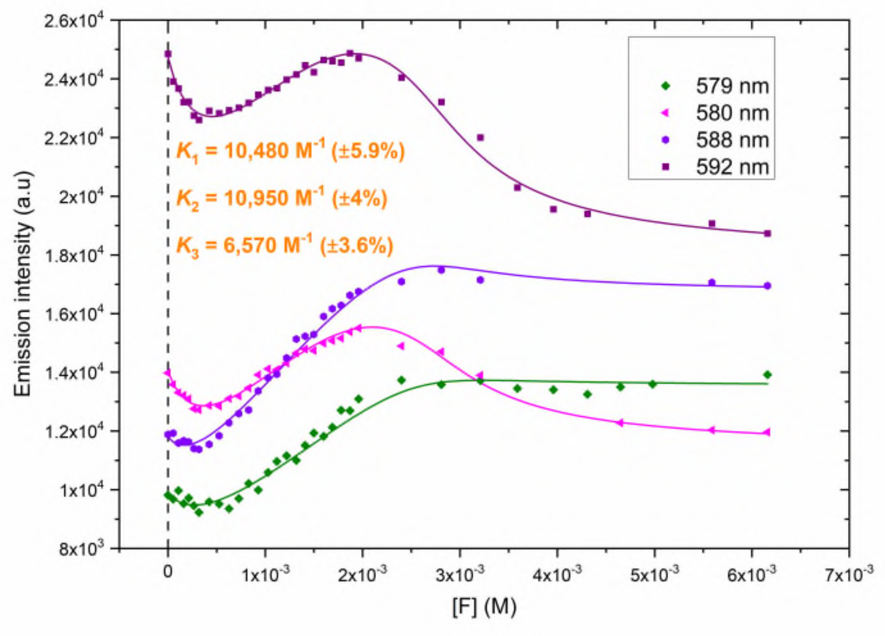

Figure S79: Binding isotherm for the binding of fluoride to [Eu2(**DO3A**)2**C-2**], obtained by plotting the intensity of the emission maxima in the $\Delta J = 0$ and $\Delta J = 1$ emission bands as a function of the concentration of KF in deionised water at 22 °C. All solid lines represent the iterative fit. 95% CI for K_1 is 9,323 – 11,780 M^{-1}; K_2 is 10,130 – 11,850 M^{-1}; K_3 is 6,120 – 7,060 M^{-1}. Steady-state titration spectra located in the manuscript (Figure 3a).

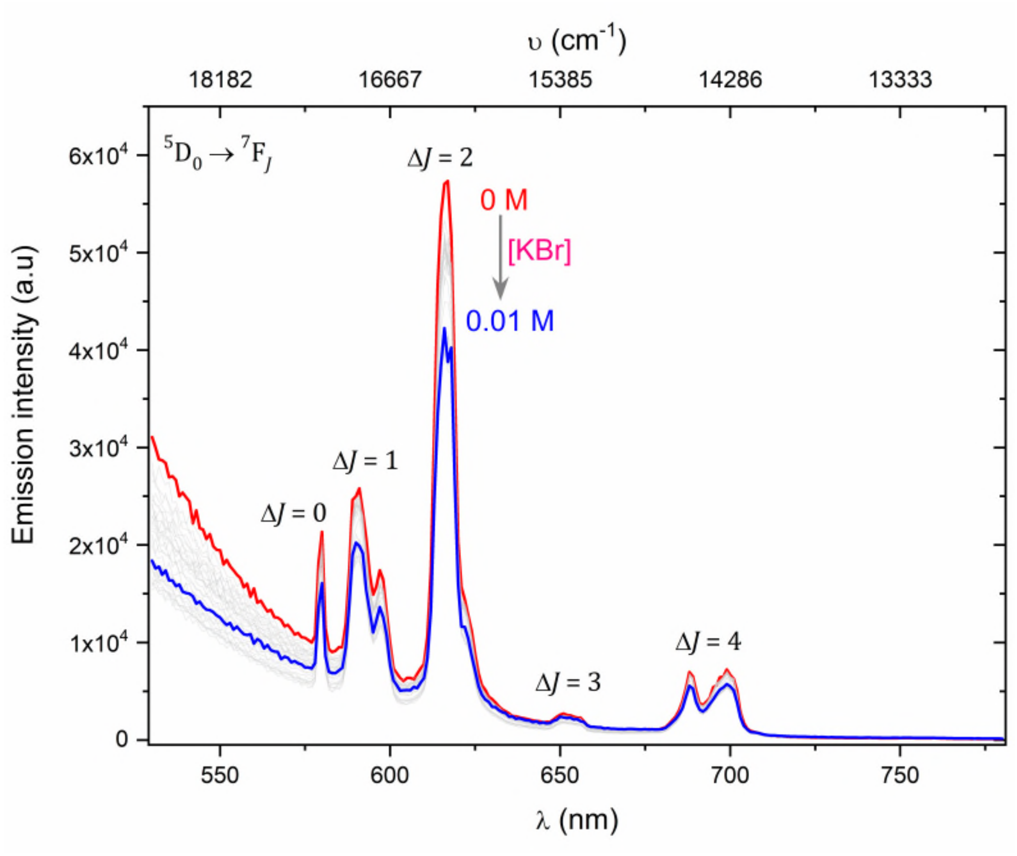

Figure S80: Steady-state luminescence titration spectra of 1 mM [Eu_2(**DO3A**)$_2$**C-2**] (λ_{ex} = 393 nm) against KBr (stock concentration = 0.02 M) in deionised water at 22 °C; spectrum of neat complex solution (*red*), spectra upon the additions of KBr (*grey*), spectrum upon the final addition of KBr (*blue*) (non-dilution method used).

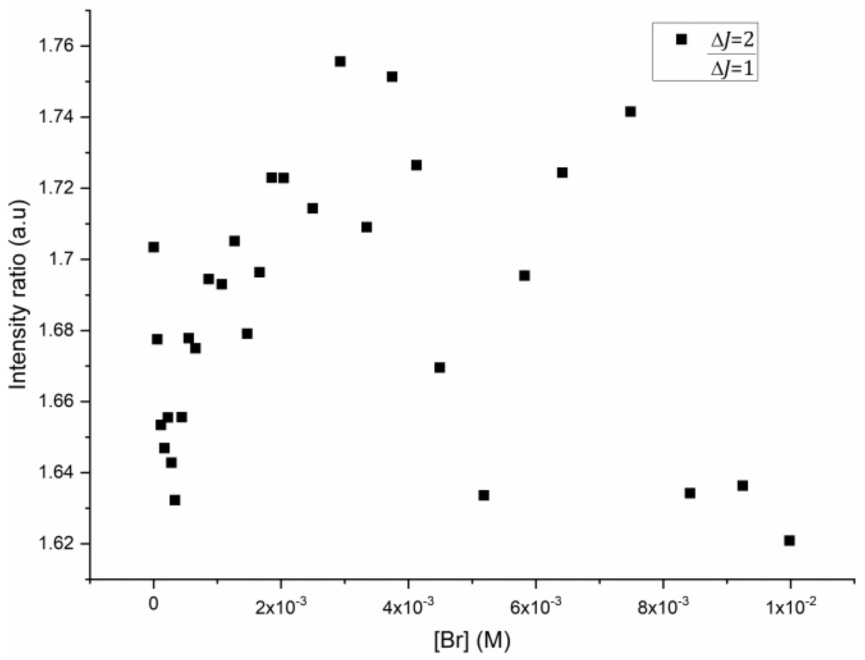

Figure S81: Emission trend for the interaction of bromide to [Eu_2(**DO3A**)$_2$**C-2**] obtained by plotting the ratio of emission bands Δ*J* = 2/Δ*J* = 1 as a function of the concentration of KBr in deionised water at 22 °C.

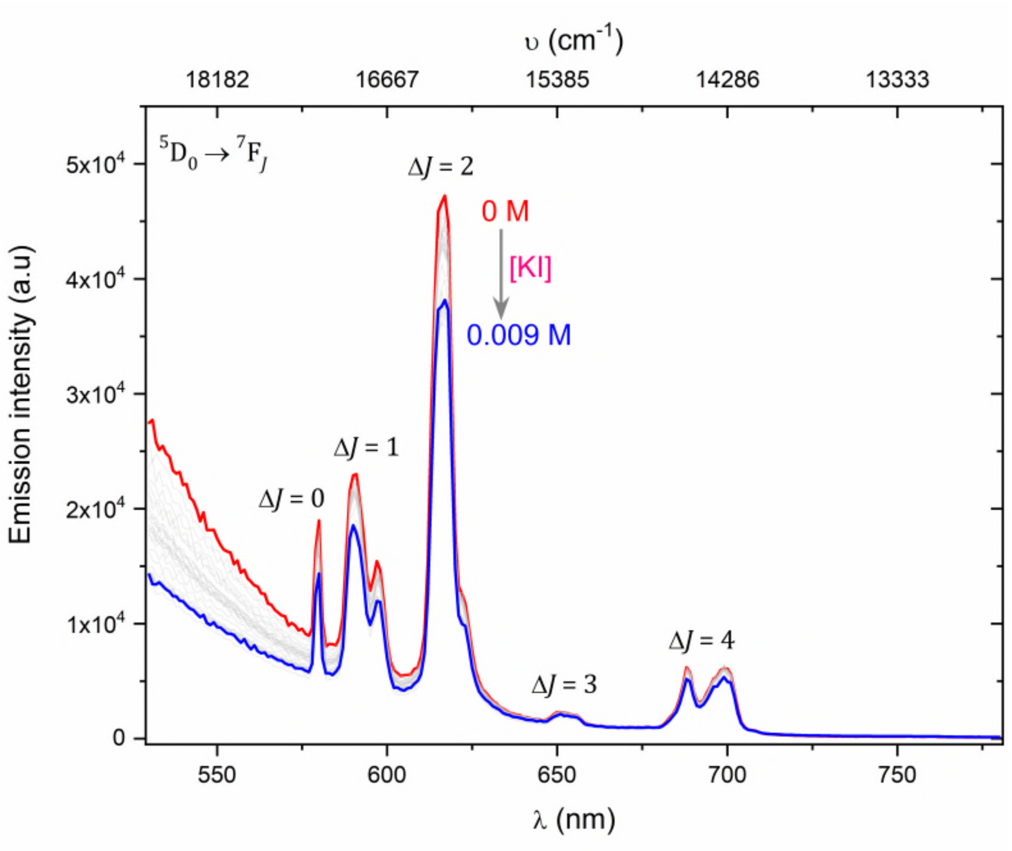

Figure S82: Steady-state luminescence titration spectra of 1 mM [Eu2(**DO3A**)2**C-2**] (λ_{ex} = 393 nm) against KI (stock concentration = 0.02 M) in deionised water at 22 °C; spectrum of neat complex solution (*red*), spectra upon the additions of KI (*grey*), spectrum upon the final addition of KI (*blue*) (non-dilution method used).

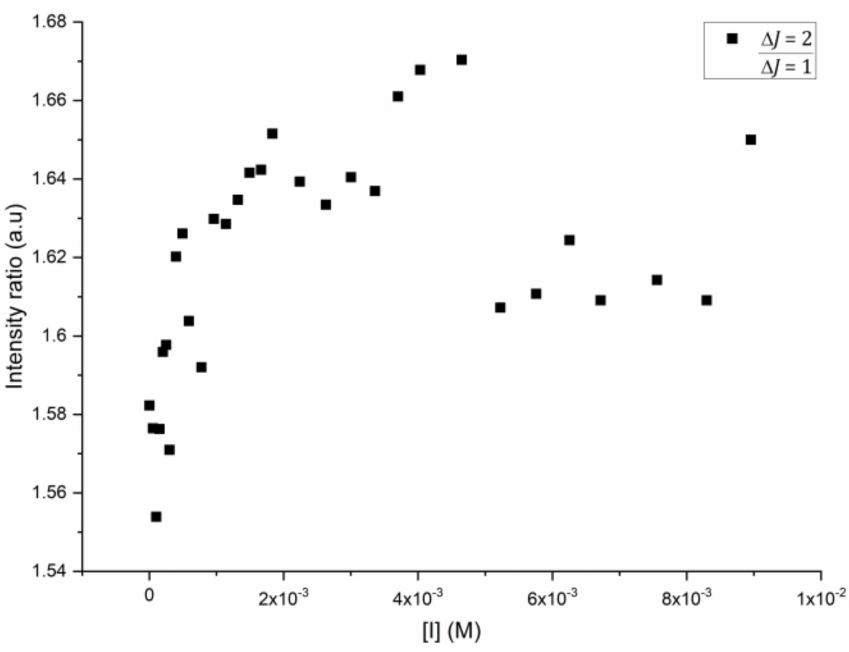

Figure S83: Emission trend for the interaction of iodide to [Eu2(**DO3A**)2**C-2**] obtained by plotting the ratio of emission bands Δ*J* = 2/Δ*J* = 1 as a function of the concentration of KI in deionised water at 22 °C.

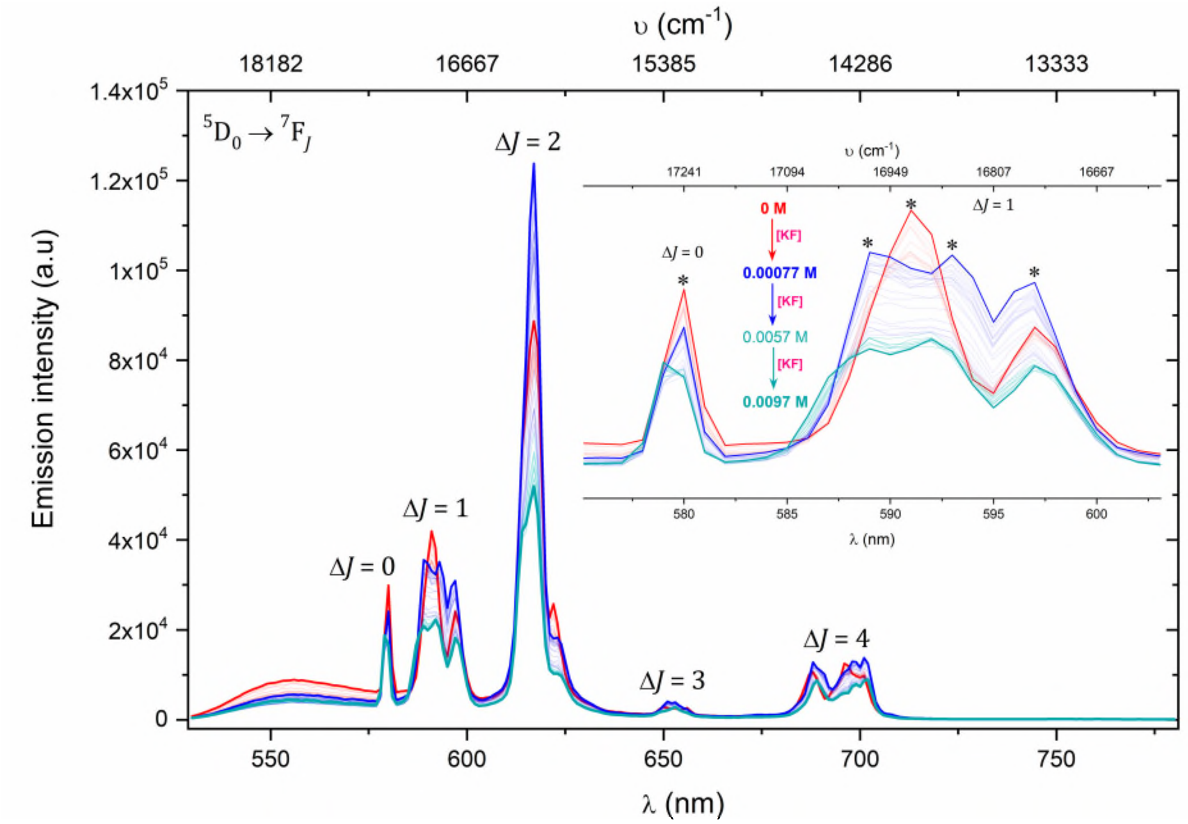

Figure S84: Steady-state luminescence titration spectra of 1 mM [Eu_2(**DO3A**)$_2$**C-3**] (λ_{ex} = 393 nm) against KF in deionised water at 22 °C; spectrum of neat complex solution (***red in bold***), spectra upon the additions of KF (*red*, ***blue in bold***, *blue to cyan*), spectrum upon the final addition of KF (***cyan in bold***) (non-dilution method used). The inset expands the $\Delta J = 0$ and $\Delta J = 1$ emission bands and the asterisks highlight the emission maxima used in quantifying binding of fluoride to the complex.

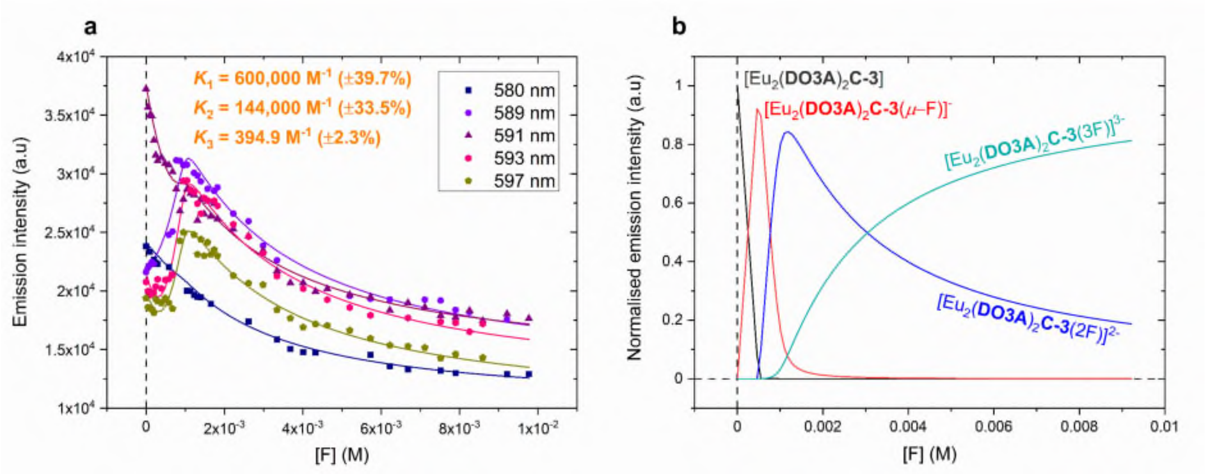

Figure S85: (a) Binding isotherm for the binding of fluoride to [Eu_2(**DO3A**)$_2$**C-3**], obtained by plotting the intensity of emission maxima in the $\Delta J = 0$ and $\Delta J = 1$ emission bands as a function of the concentration of KF in deionised water at 22 °C. 95% CI for K_1 is 290,000 – 1,600,000 M^{-1}; K_2 is 79,500 –363,000 M^{-1}; K_3 is 377.2 – 413.4 M^{-1}. All solid lines represent the iterative fit. **(b)** Normalised model of speciation for the binding of fluoride to [Eu_2(**DO3A**)$_2$**C-3**], obtained by plotting the 580 nm emission as a function of the concentration of KF in deionised water at 22 °C.

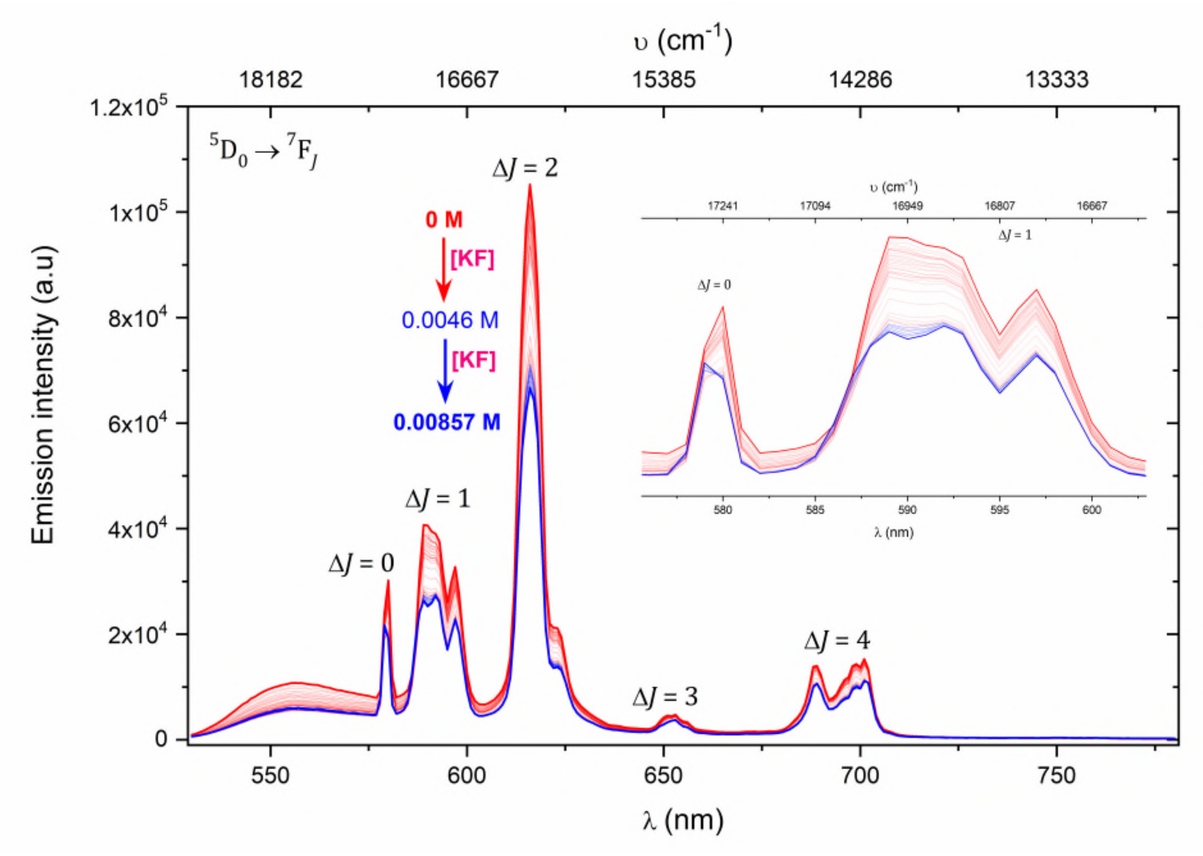

Figure S86: Steady-state luminescence titration spectra of 1 mM [Eu_2(**DO3A**)$_2$**C-3**] (λ_{ex} = 393 nm) against KF (stock concentration = 0.02 M) in 0.01 M PBS (pH 7.4) at 22 °C; spectrum of neat complex solution (***red in bold***), spectra upon the additions of KF (*red to blue*), spectrum upon the final addition of KF (***blue in bold***) (non-dilution method used). The inset expands the $\Delta J = 0$ and $\Delta J = 1$ emission bands.

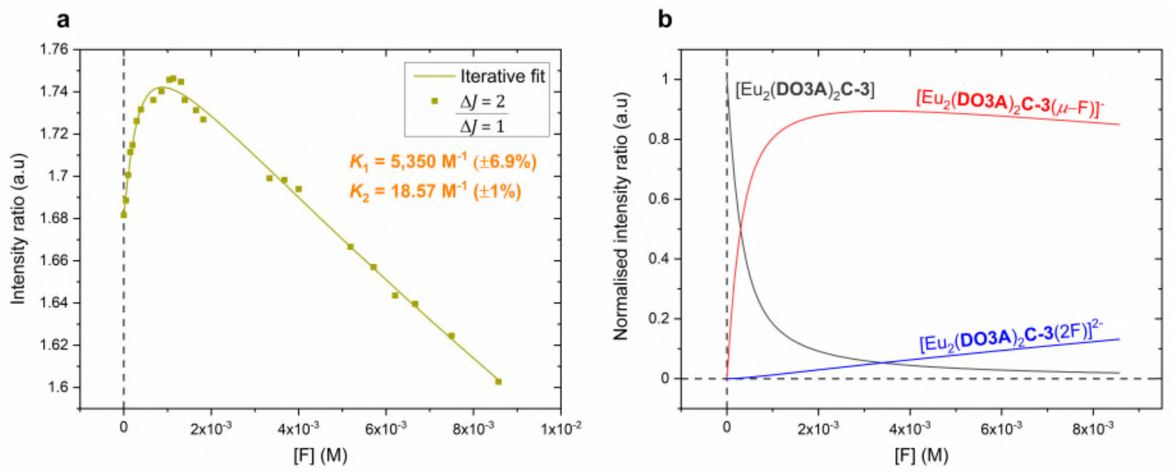

Figure S87: (a) Binding isotherm for the binding of fluoride to [Eu_2(**DO3A**)$_2$**C-3**], obtained by plotting the ratio of the emission bands $\Delta J = 2/\Delta J = 1$ as a function of the concentration of KF in 0.01 M PBS (pH 7.4) at 22 °C. 95% CI for K_1 is 4,640 – 6,190 M^{-1}; K_2 is 18.2 – 18.94 M^{-1}. **(b)** Normalised model of speciation for the binding of fluoride to [Eu_2(**DO3A**)$_2$**C-3**], obtained by plotting the ratio of the emission bands $\Delta J = 2/\Delta J = 1$ as a function of the concentration of KF in 0.01 M PBS (pH 7.4) at 22 °C.

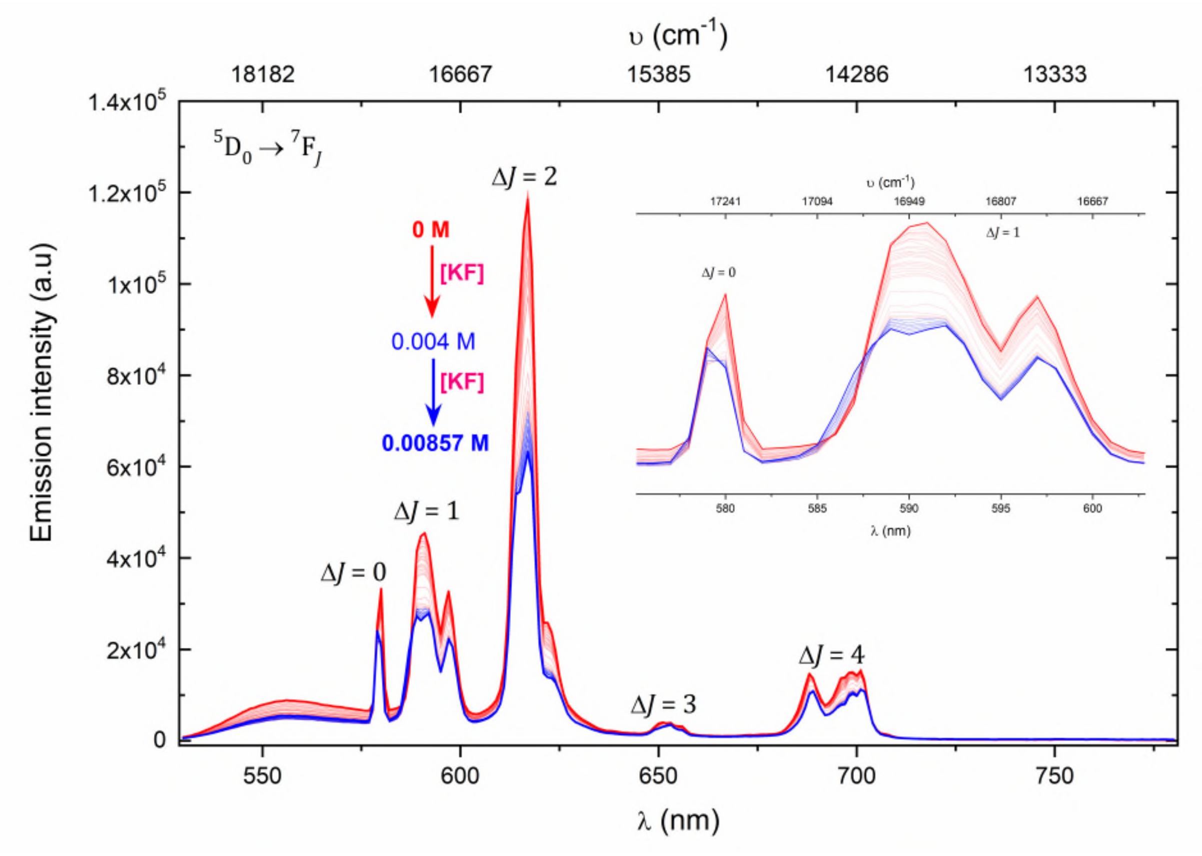

Figure S88: Steady-state luminescence titration spectra of 1 mM [Eu_2(**DO3A**)$_2$**C-3**] (λ_{ex} = 393 nm) against KF (stock concentration = 0.02 M) in 0.01 M Tris-HCl buffer (pH 7.4) at 22 °C; spectrum of neat complex solution (***red in bold***), spectra upon the additions of KF (*red to blue*), spectrum upon the final addition of KF (***blue in bold***) (non-dilution method used). The inset expands the Δ*J* = *0* and Δ*J* = *1* emission bands.

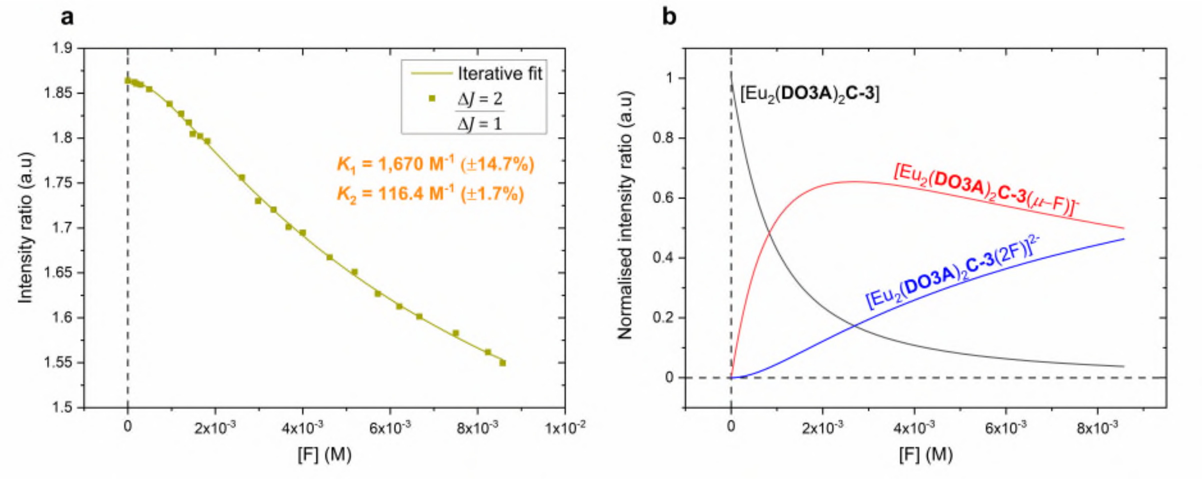

Figure S89: (a) Binding isotherm for the binding of fluoride to [Eu_2(**DO3A**)$_2$**C-3**], obtained by plotting the ratio of the intensity of emission bands Δ*J* = 2/Δ*J* = 1 as a function of the concentration of KF in 0.01 M Tris-HCl buffer (pH 7.4) at 22 °C. 95% CI for K_1 is 1,260 – 2,340 M^{-1}; K_2 is 112.5 – 120.5 M^{-1}. **(b)** Normalised model of speciation for the binding of fluoride to [Eu_2(**DO3A**)$_2$**C-3**], obtained by plotting the ratio of the emission bands Δ*J* = 2/Δ*J* = 1 as a function of the concentration of KF in 0.01 M Tris-HCl buffer (pH 7.4) at 22 °C.

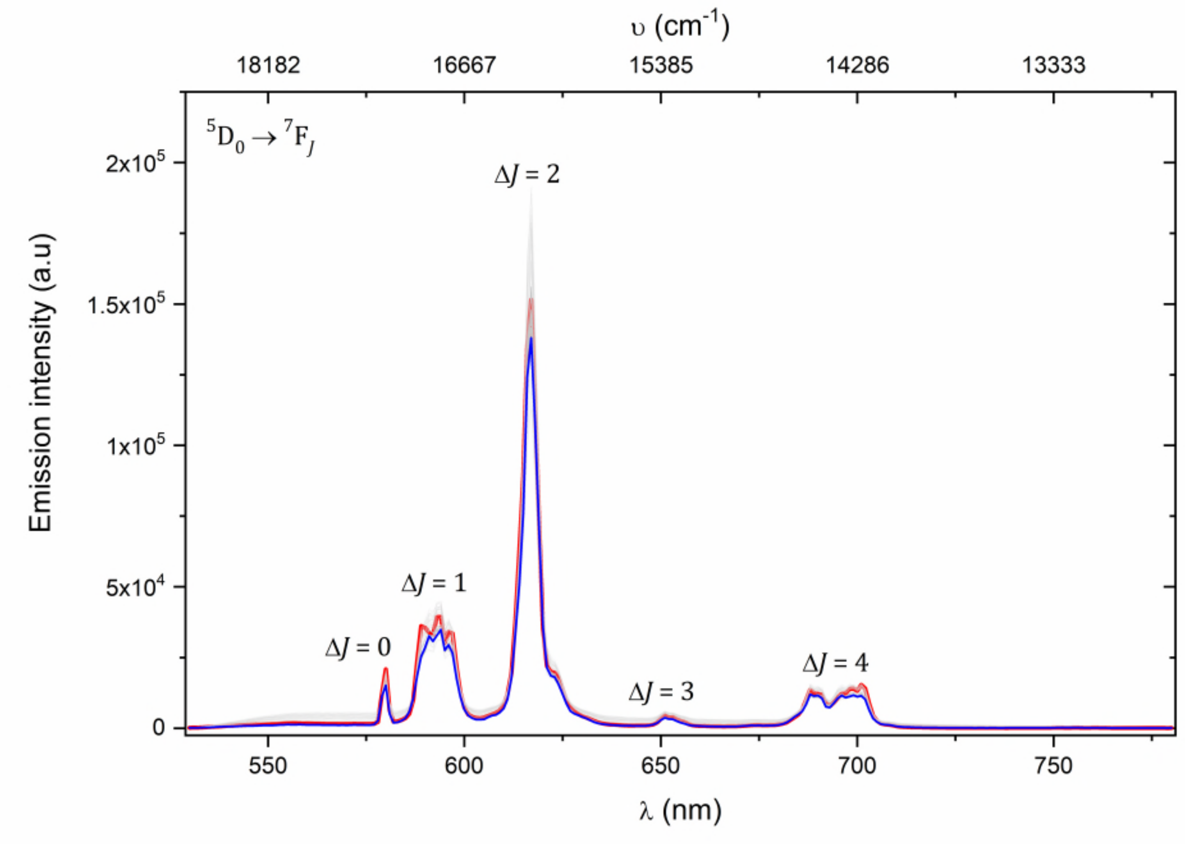

Figure S90: Steady-state luminescence titration spectra of 1 mM [Eu_2(**DO3A**)$_2$**C-3**] (λ_{ex} = 393 nm) against KF (stock concentration = 0.02 M) in 0.01 M CHES buffer (pH 9.98) at 22 °C; spectrum of neat complex solution (*red*), spectra upon the additions of KF (*grey*), spectrum upon the final addition of KF (*blue*) (non-dilution method used).

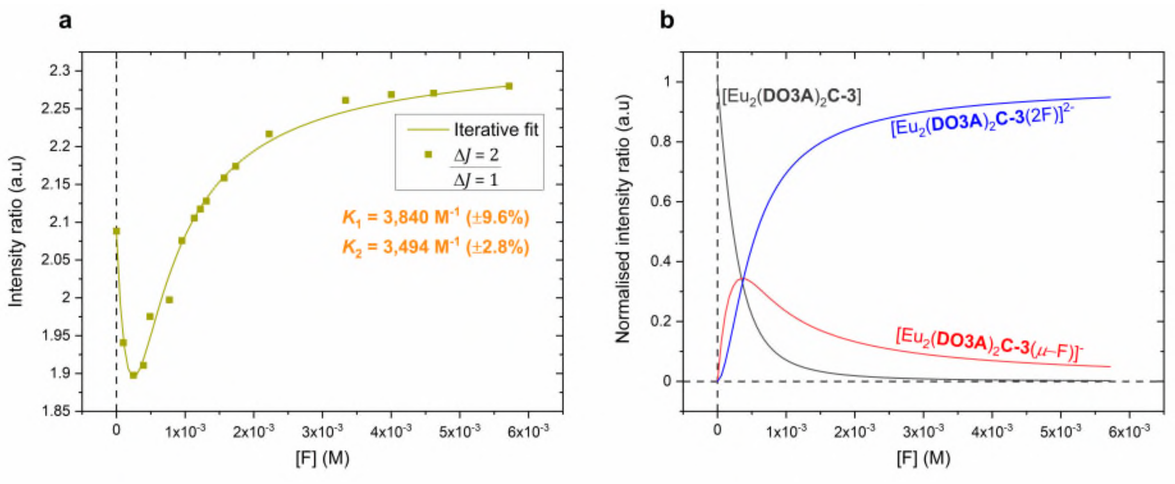

Figure S91: **(a)** Binding isotherm for the binding of fluoride to [Eu_2(**DO3A**)$_2$**C-3**], obtained by plotting the ratio of the emission bands $\Delta J = 2/\Delta J = 1$ as a function of the concentration of KF in 0.01 M CHES buffer (pH 9.98) at 22 °C. 95% CI for K_1 is 3,120 – 4,710 M^{-1}; K_2 is 3,293 – 3,709 M^{-1}. **(b)** Normalised model of speciation for the binding of fluoride to [Eu_2(**DO3A**)$_2$**C-3**], obtained by plotting the ratio of the emission bands $\Delta J = 2/\Delta J = 1$ as a function of the concentration of KF in 0.01 M CHES buffer (pH 9.98) at 22 °C.

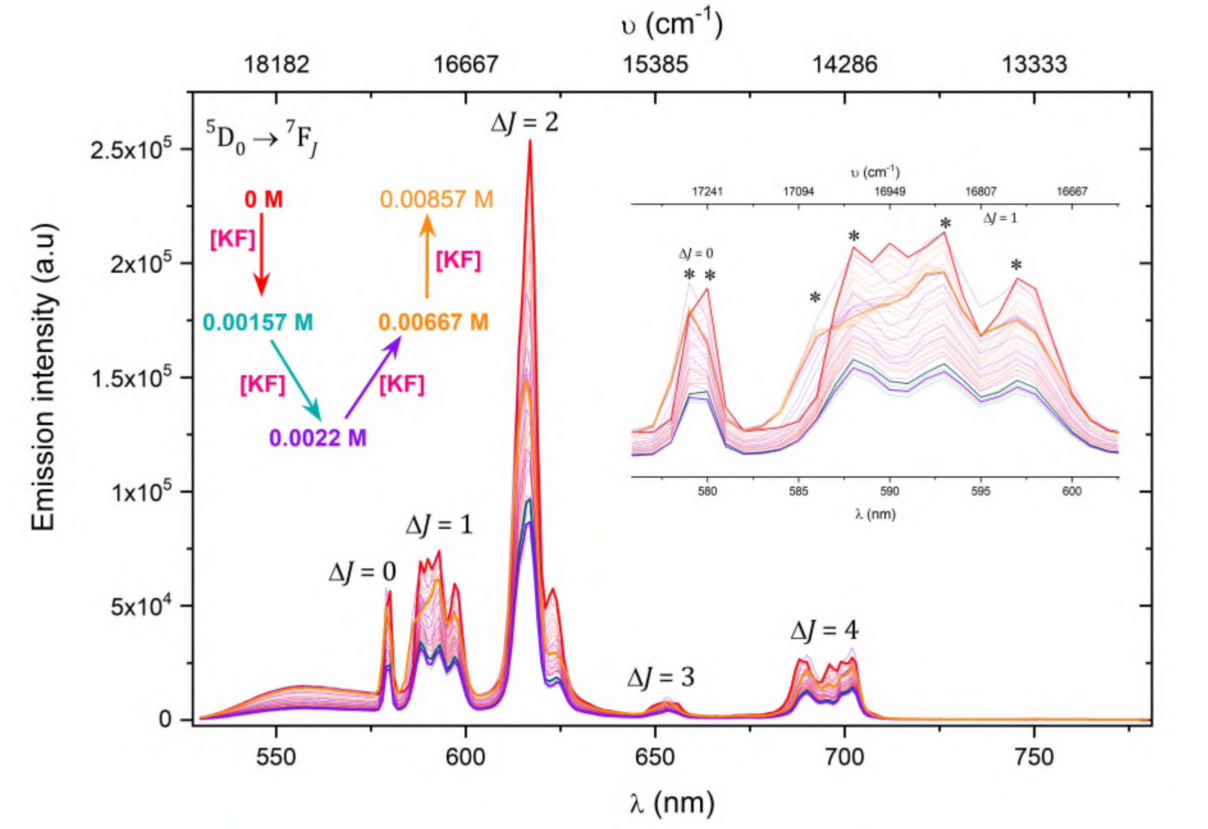

Figure S92: Steady-state luminescence titration spectra of 1 mM [Eu_2(**DO3A**)$_2$**C-3**] (λ_{ex} = 393 nm) against KF (stock concentration = 0.02 M) in methanol at 22 °C; spectrum of neat complex solution (***red in bold***), spectra upon the additions of KF (*red*, ***cyan in bold***, *cyan*, ***violet in bold***, *violet*, ***orange in bold*** *and* *orange*), spectrum upon the final addition of KF (*orange*) (non-dilution method used). The inset expands the Δ*J* = *0* and Δ*J* = 1 emission bands and the asterisks highlight the emission maxima used in quantifying binding of fluoride to the complex.

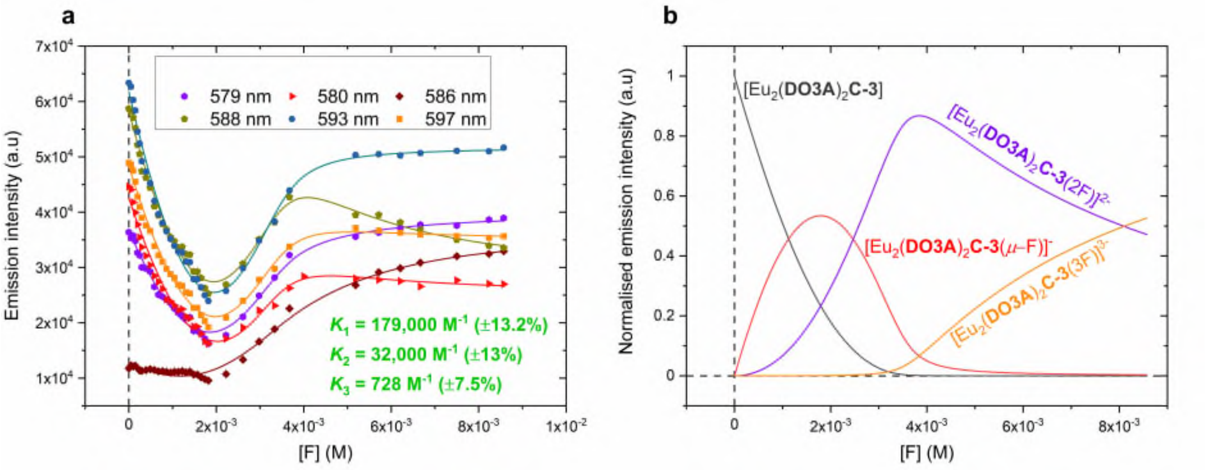

Figure S93: **(a)** Binding isotherm for the binding of fluoride to [Eu_2(**DO3A**)$_2$**C-3**], obtained by plotting the intensity of the emission maxima of the Δ*J* = *0* and Δ*J* = *1* emission bands as a function of the concentration of KF in methanol at 22 °C. 95% CI for K_1 is 139,000 – 234,000 M^{-1}; K_2 is 25,100 – 41,800 M^{-1}; K_3 is 629 – 847 M^{-1}. All solid lines represent the iterative fit. **(b)** Normalised model of speciation for the binding of fluoride to [Eu_2(**DO3A**)$_2$**C-3**], obtained by plotting the 588 nm emission as a function of the concentration of KF in methanol at 22 °C.

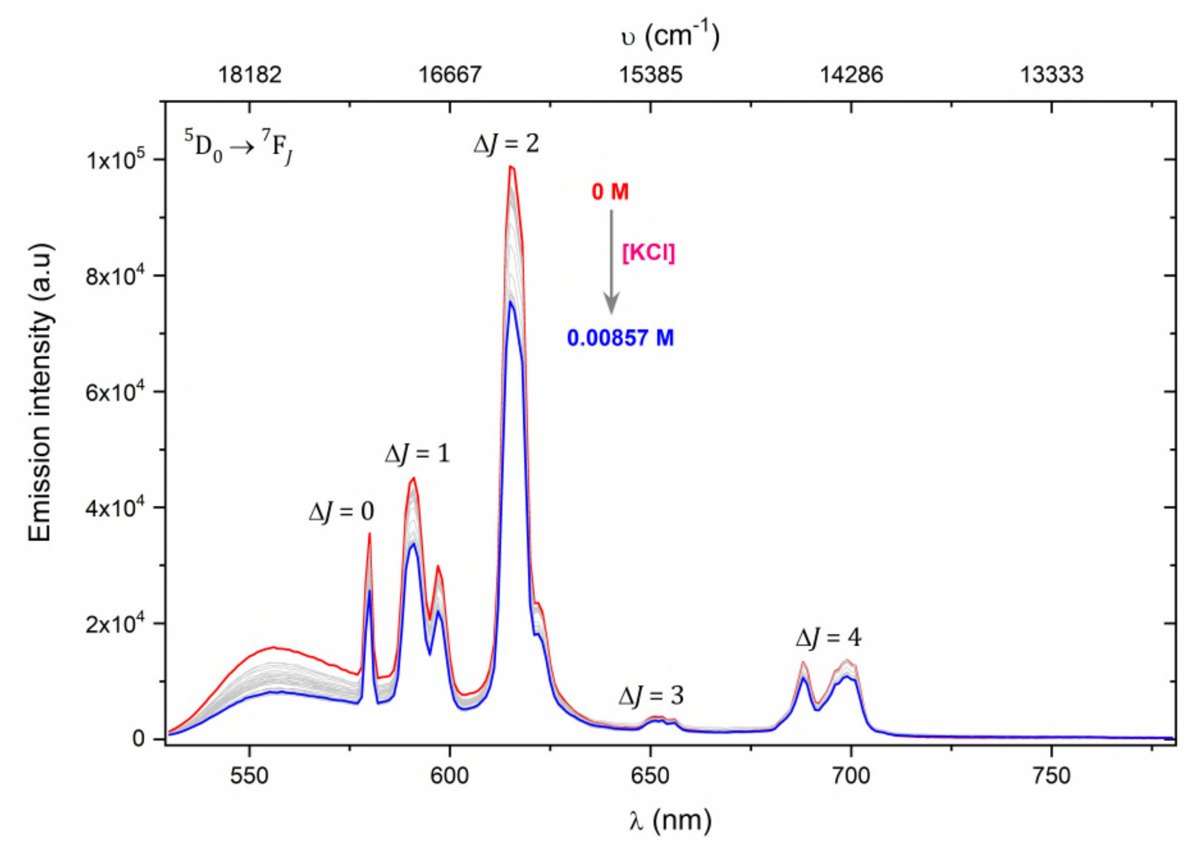

Figure S94: Steady-state luminescence titration spectra of 1 mM [Eu_2(**DO3A**)$_2$**C-3**] (λ_{ex} = 393 nm) against KCl (stock concentration = 0.02 M) in deionised water at 22 °C; spectrum of neat complex solution (*red*), spectra upon the additions of KCl (*grey*), spectrum upon the final addition of KCl (*blue*) (non-dilution method used).

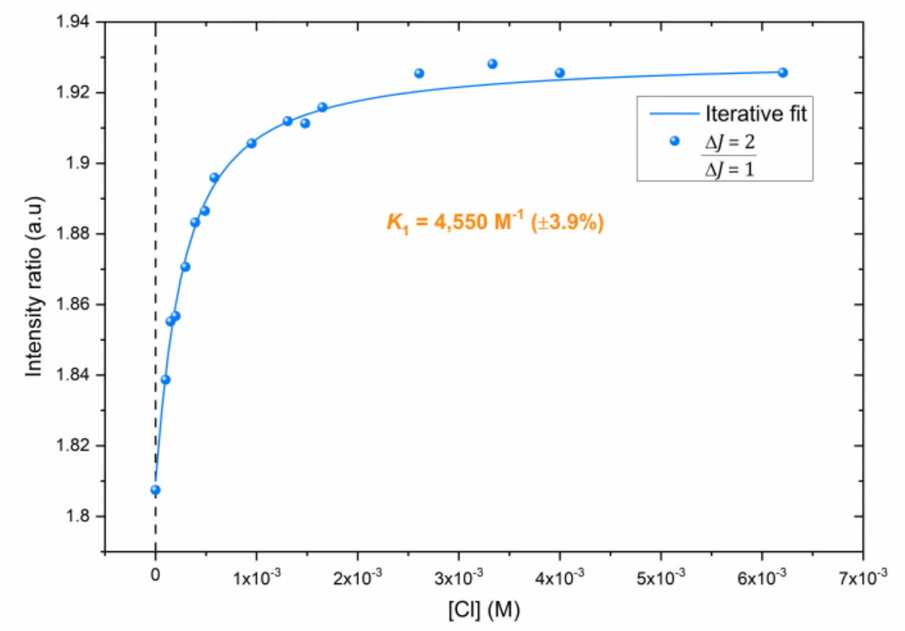

Figure S95: Binding isotherm for the binding of chloride to [Eu_2(**DO3A**)$_2$**C-3**], obtained by plotting the ratio of the emission bands $\Delta J = 2/\Delta J = 1$ as a function of the concentration of KCl in deionised water. 95% CI for K_1 is 4,200 – 4,940 M^{-1}.

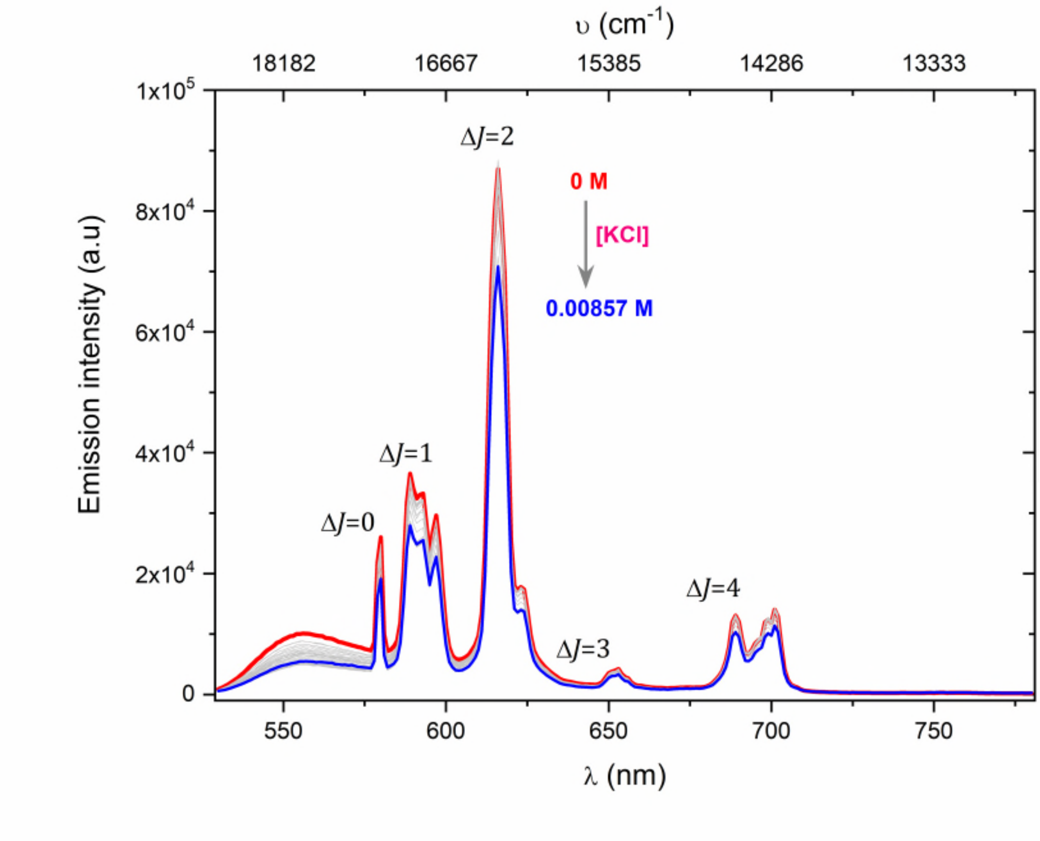

Figure S96: Steady-state luminescence titration spectra of 1 mM [Eu$_2$(**DO3A**)$_2$**C-3**] (λ_{ex} = 393 nm) against KCl (stock concentration = 0.02 M) in 0.01 M phosphate buffer (pH 7.4) at 22 °C; spectrum of neat complex solution (*red*), spectra upon the additions of KCl (*grey*), spectrum upon the final addition of KCl (*blue*) (non-dilution method used).

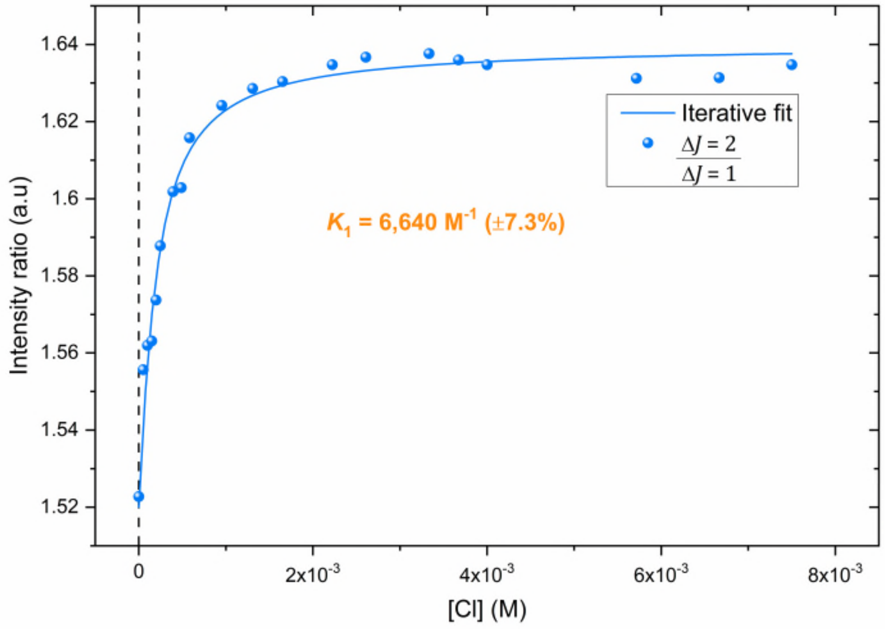

Figure S97: Binding isotherm for the binding of chloride to [Eu$_2$(**DO3A**)$_2$**C-3**], obtained by plotting the ratio of the emission bands $\Delta J = 2/\Delta J = 1$ as a function of the concentration of KCl in 0.01 M phosphate buffer (pH 7.4) at 22 °C. 95% CI for K_1 is 5,710 – 7,760 M^{-1}.

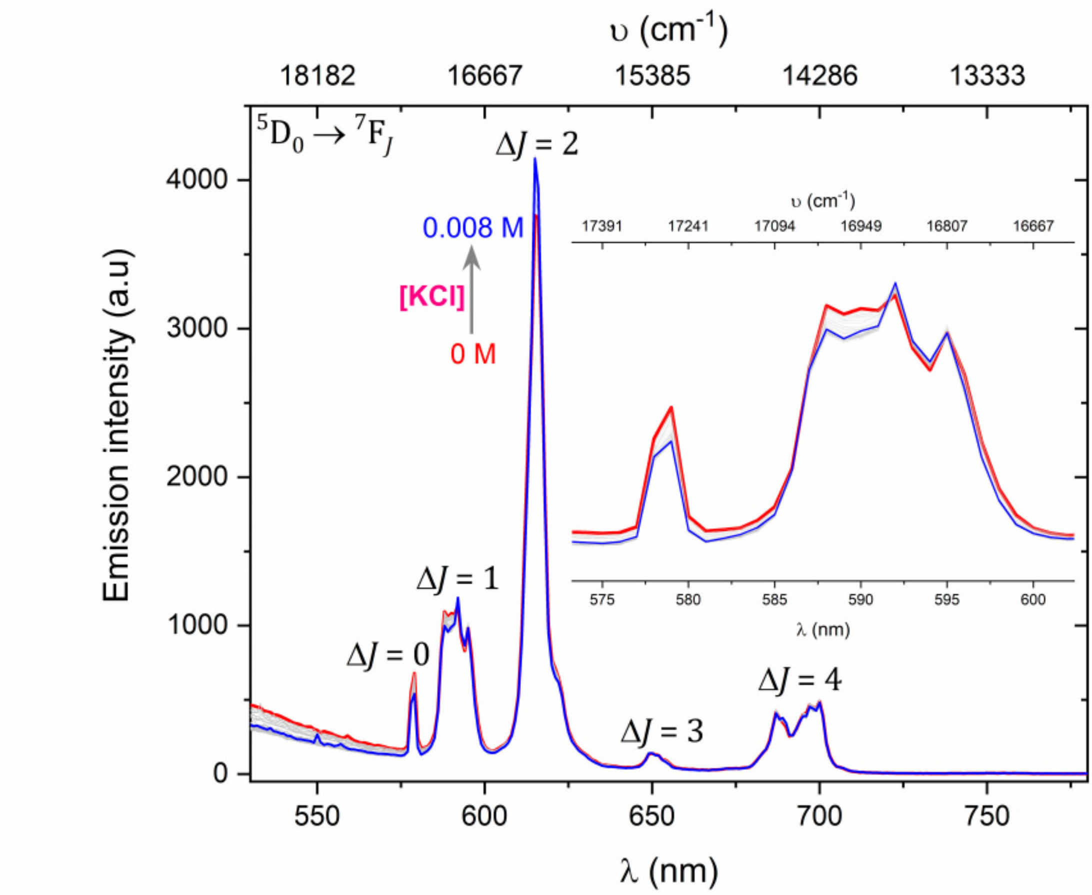

Figure S98: Steady-state luminescence titration spectra of 1 mM [Eu_2(**DO3A**)$_2$**C-3**] (λ_{ex} = 393 nm) against KCl (stock concentration = 0.02 M) in 0.01 M CHES buffer (pH 9.98) at 22 °C; spectrum of neat complex solution (*red*), spectra upon the additions of KCl (*grey*), spectrum upon the final addition of KCl (*blue*) (non-dilution method used). The inset expands the Δ*J* = *0* and Δ*J* = *1* emission bands.

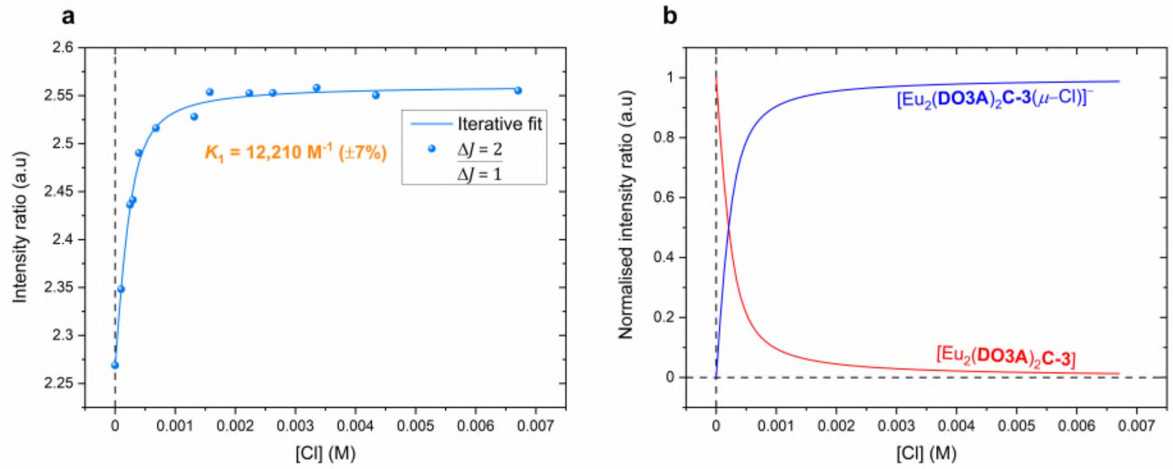

Figure S99: (a) Binding isotherm for the binding of chloride to [Eu_2(**DO3A**)$_2$**C-3**], obtained by plotting the ratio of the emission bands Δ*J* = 2/Δ*J* = 1 as a function of the concentration of KCl in 0.01 M CHES buffer (pH 9.88) at 22 °C. 95% CI for K_1 is 10,520 – 14,290 M^{-1}. **(b)** Normalised model of speciation for the binding of chloride to [Eu_2(**DO3A**)$_2$**C-3**], obtained by plotting the ratio of the emission bands Δ*J* = 2/Δ*J* = 1 as a function of the concentration of KCl in in 0.01 M CHES buffer (pH 9.88) at 22 °C.

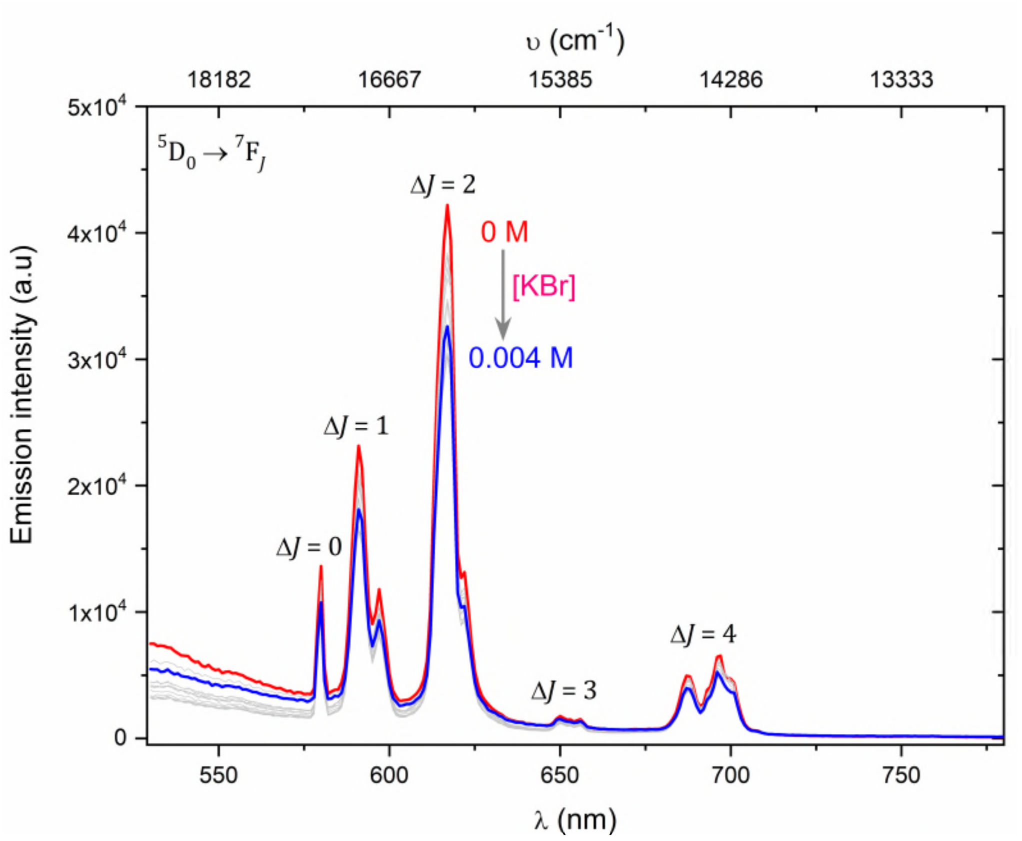

Figure S100: Steady-state luminescence titration spectra of 1 mM [Eu_2(**DO3A**)$_2$**C-3**] (λ_{ex} = 393 nm) against KBr (stock concentration = 0.02 M) in deionised water at 22 °C; spectrum of neat complex solution (*red*), spectra upon the additions of KBr (*grey*), spectrum upon the final addition of KBr (*blue*) (non-dilution method used).

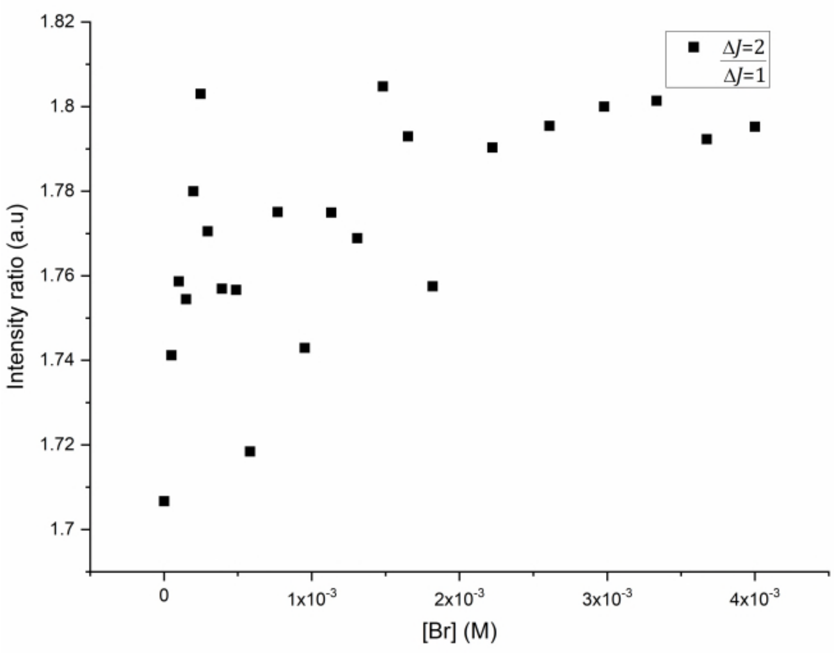

Figure S101: Emission trend for the interaction of bromide to [Eu_2(**DO3A**)$_2$**C-3**] obtained by plotting the ratio of emission bands Δ*J* = 2/Δ*J* = 1 as a function of the concentration of KBr in deionised water at 22 °C.

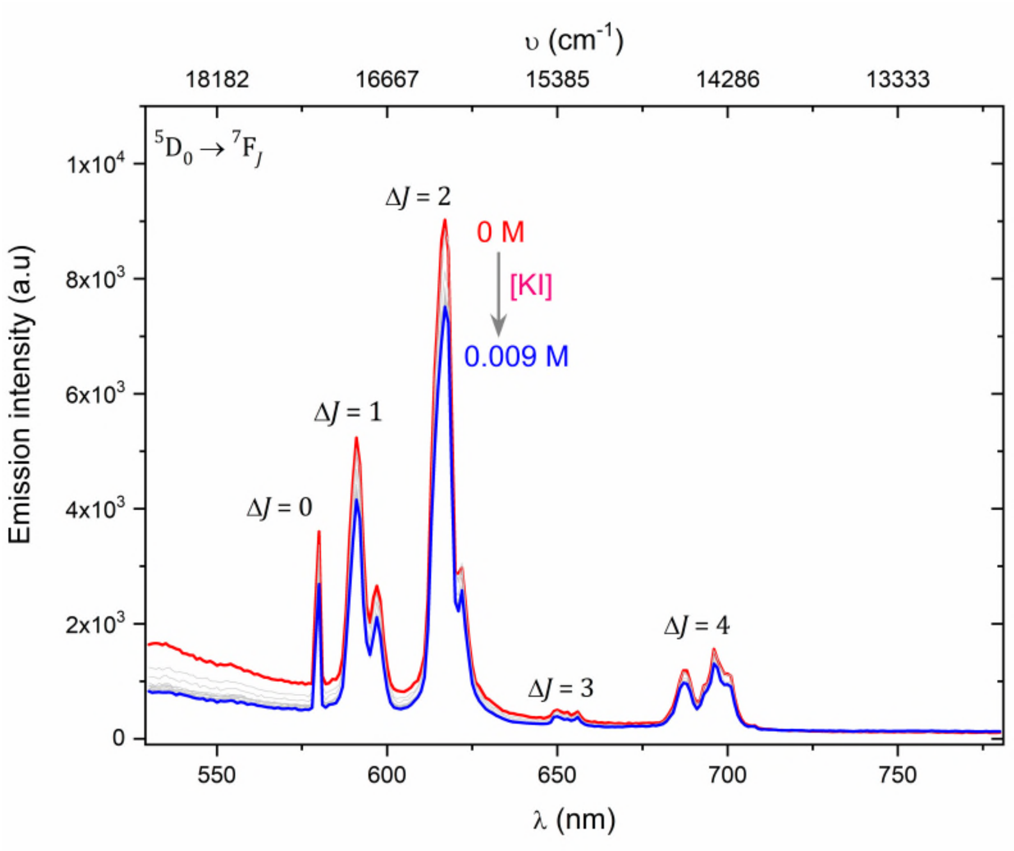

Figure S102: Steady-state luminescence titration spectra of 1 mM [Eu_2(**DO3A**)$_2$**C-3**] (λ_{ex} = 393 nm) against KI (stock concentration = 0.02 M) in deionised water at 22 °C; spectrum of neat complex solution (*red*), spectra upon the additions of KI (*grey*), spectrum upon the final addition of KI (*blue*) (non-dilution method used).

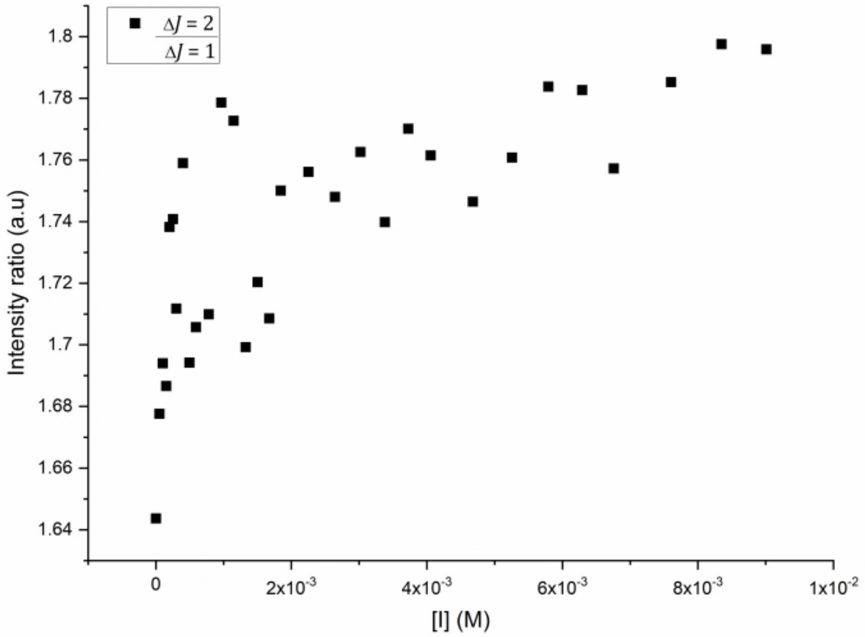

Figure S103: Emission trend for the interaction of iodide to [Eu_2(**DO3A**)$_2$**C-3**] obtained by plotting the ratio of emission bands ΔJ = 2/ΔJ = 1 as a function of the concentration of KI in deionised water at 22 °C.

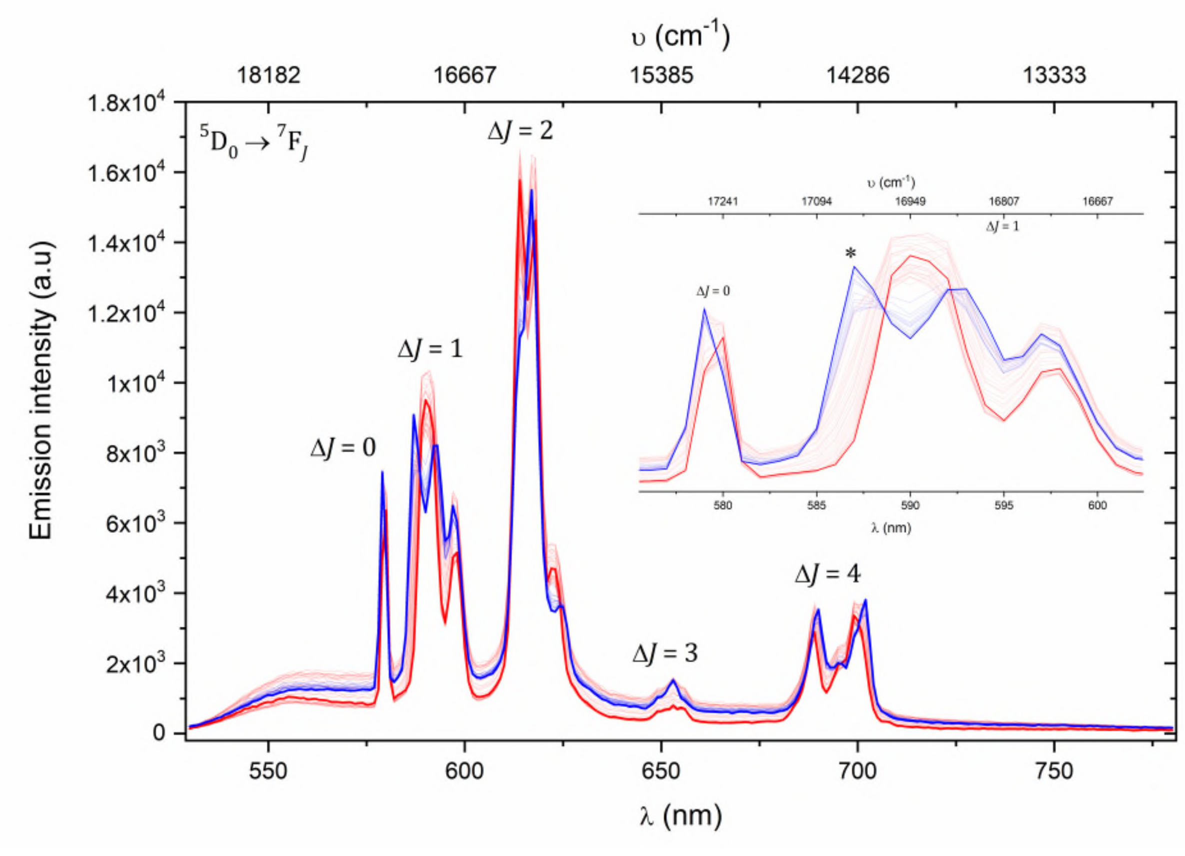

Figure S104: Steady-state luminescence titration spectra of 1 mM [Eu(**pDO3A**)] (λ_{ex} = 393 nm) against KF (stock concentration = 0.02 M) in deionised water at 22 °C; spectrum of neat complex solution (***red in bold***), spectra upon the additions of KF (*red* to *blue*), spectrum upon the final addition of KF (***blue in bold***) (non-dilution method used). The inset expands the Δ*J* = *0* and Δ*J* = 1 emission bands and the asterisk highlight the emission maximum used in quantifying binding of fluoride to the complex.

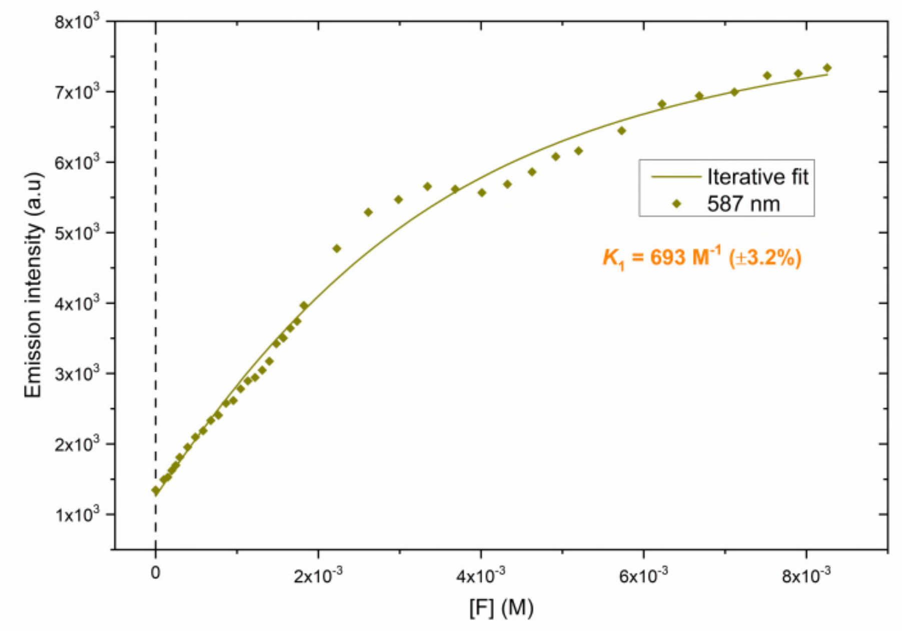

Figure S105: Binding isotherm for the binding of fluoride to [Eu(**pDO3A**)], obtained by plotting the 587 nm emission as a function of the concentration of KF in deionised water at 22 °C. 95% CI for K_1 is 650 - 740 M^{-1}.

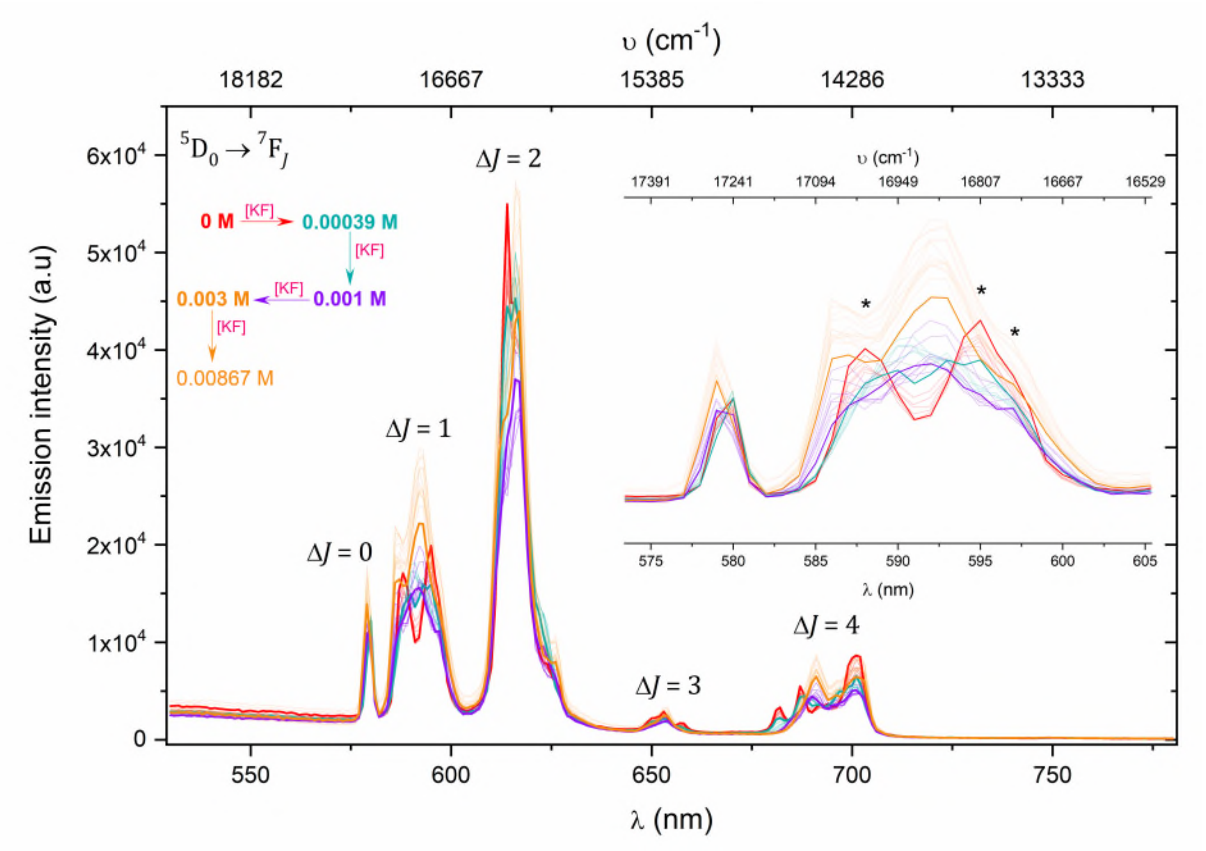

Figure S106: Steady-state luminescence titration spectra of 1 mM [Eu(**pDO3A**)] (λ_{ex} = 393 nm) against KF (stock concentration = 0.02 M) in methanol at 22 °C; spectrum of neat complex solution (***red in bold***), spectra upon the additions of KF (*red*, ***cyan in bold***, *cyan*, ***violet in bold***, *violet*, ***orange in bold*** *and* *orange*), spectrum upon the final addition of KF (*orange*) (non-dilution method used). The inset expands the Δ*J* = *0* and Δ*J* = 1 emission bands the asterisks highlight the emission intensities used in quantifying binding of fluoride to the complex.

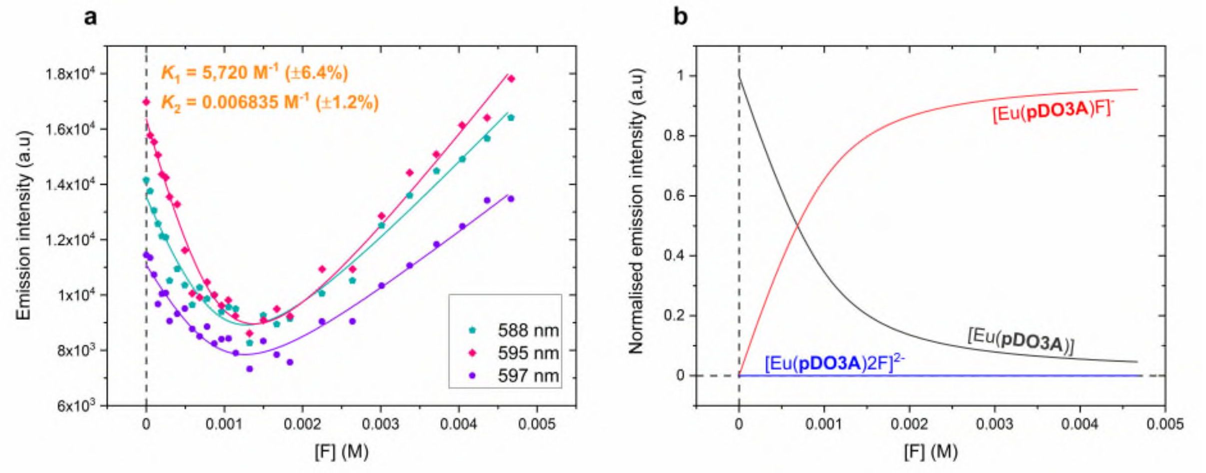

Figure S107: (a) Binding isotherm for the binding of fluoride to [Eu(**pDO3A**)], obtained by plotting few emission intensities in the Δ*J* = 1 transition as a function of the concentration of KF in methanol at 22 °C. All solid lines represent the iterative fit. 95% CI for K_1 is 5,050 – 6,510 M^{-1}; K_2 is 0.006672 – 0.006997 M^{-1}. **(b)** Normalised model of speciation for the binding of fluoride to [Eu(**pDO3A**)], obtained by plotting the 595 nm emission as a function of the concentration of KF in methanol.

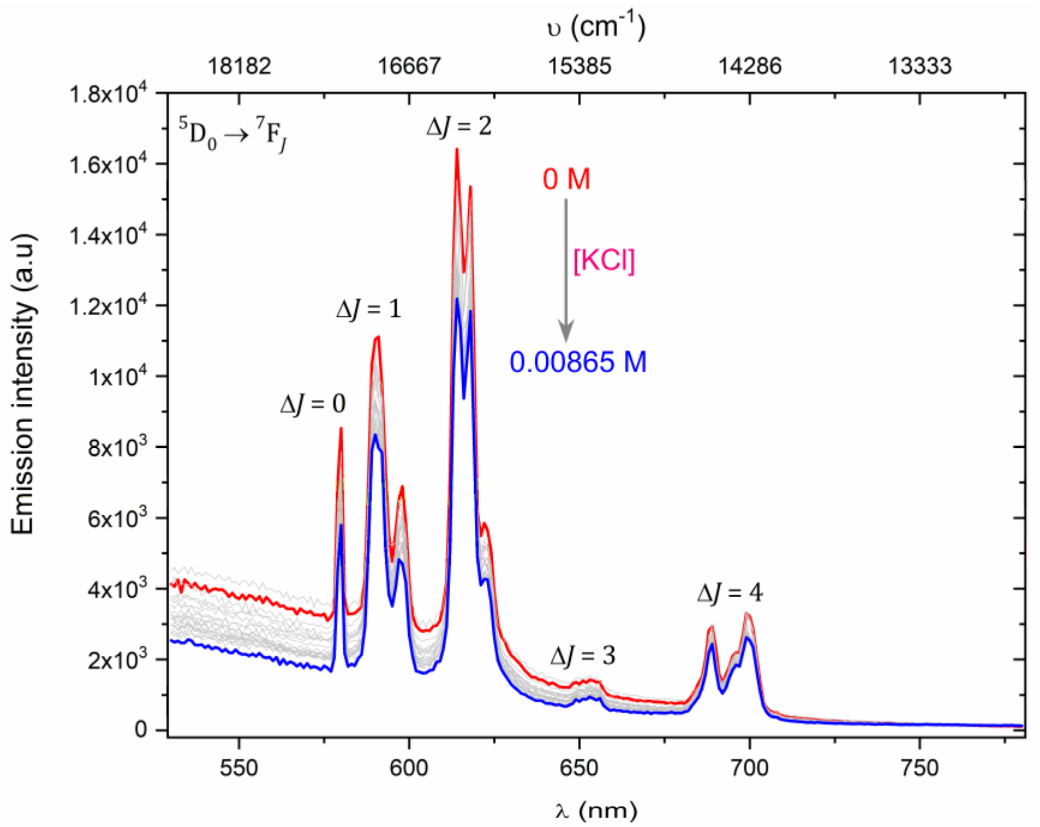

Figure S108: Steady-state luminescence titration spectra of 1 mM [Eu(**pDO3A**)] (λ_{ex} = 393 nm) against KCl (stock concentration = 0.02 M) in deionised water at 22 °C; spectrum of neat complex solution (*red*), spectra upon the additions of KCl (*grey*), spectrum upon the final addition of KCl (*blue*) (non-dilution method used).

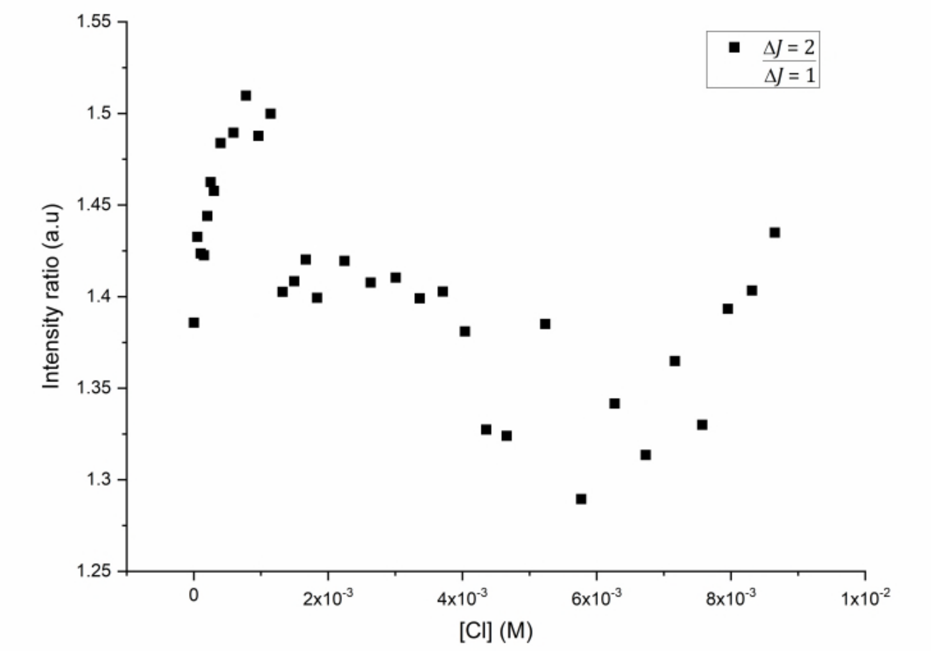

Figure S109: Emission trend for the interaction of chloride to [Eu(**pDO3A**)] obtained by plotting the ratio of emission bands $\Delta J = 2/\Delta J = 1$ as a function of the concentration of KCl in deionised water at 22 °C.

8.5. Time-resolved Luminescence Lifetime Data for Eu(III) complexes

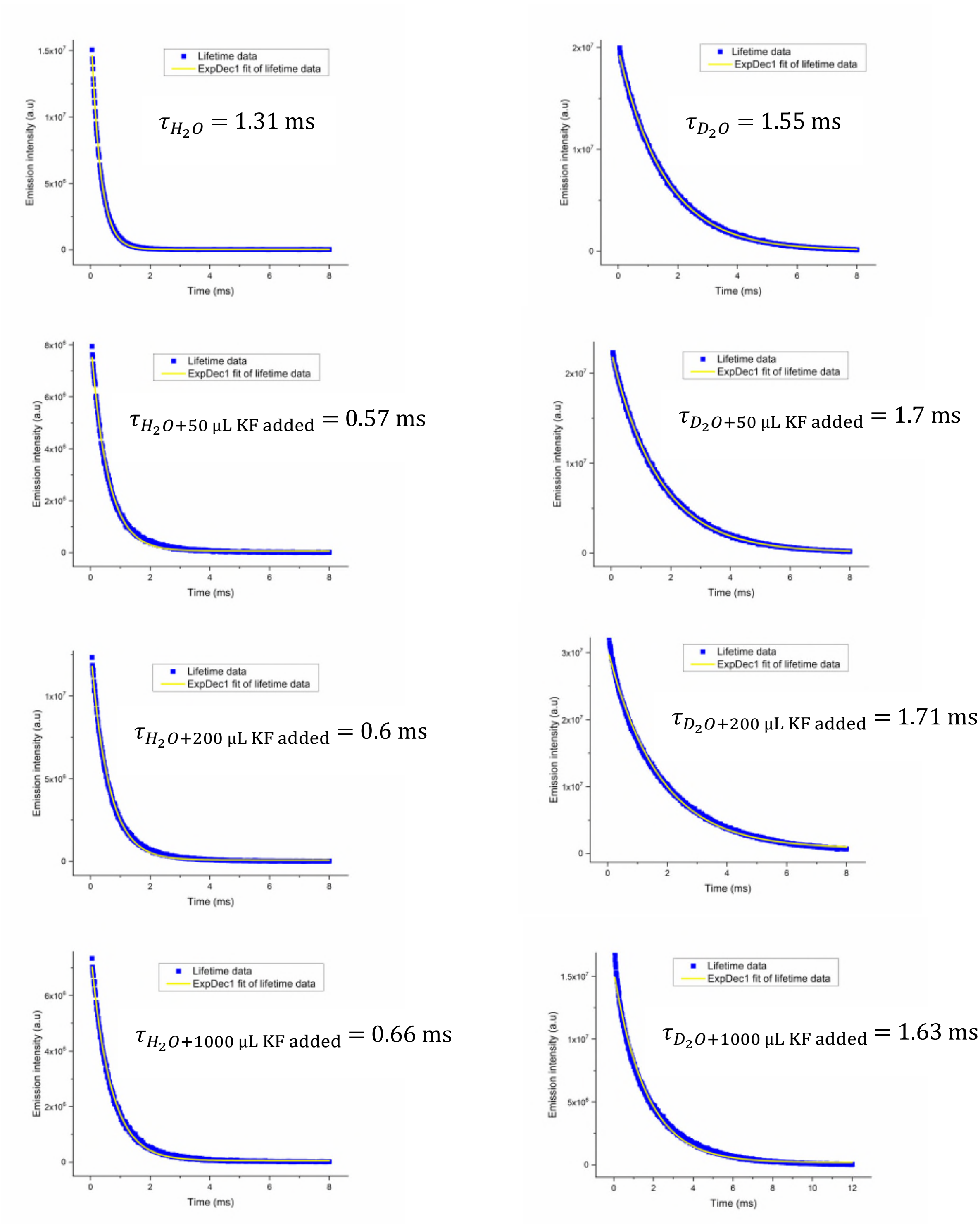

Figure S110: Time-resolved luminescence lifetime of [Eu_2(**DO3A**)$_2$**C-2**] with increasing concentration of KF in water (*left*) and deuterium oxide (*right*); concentration of [Eu_2(**DO3A**)$_2$**C-2**] is 1 mM, concentration of stock solution of KF in [Eu_2(**DO3A**)$_2$**C-2**] is 0.02 M (non-dilution method used).

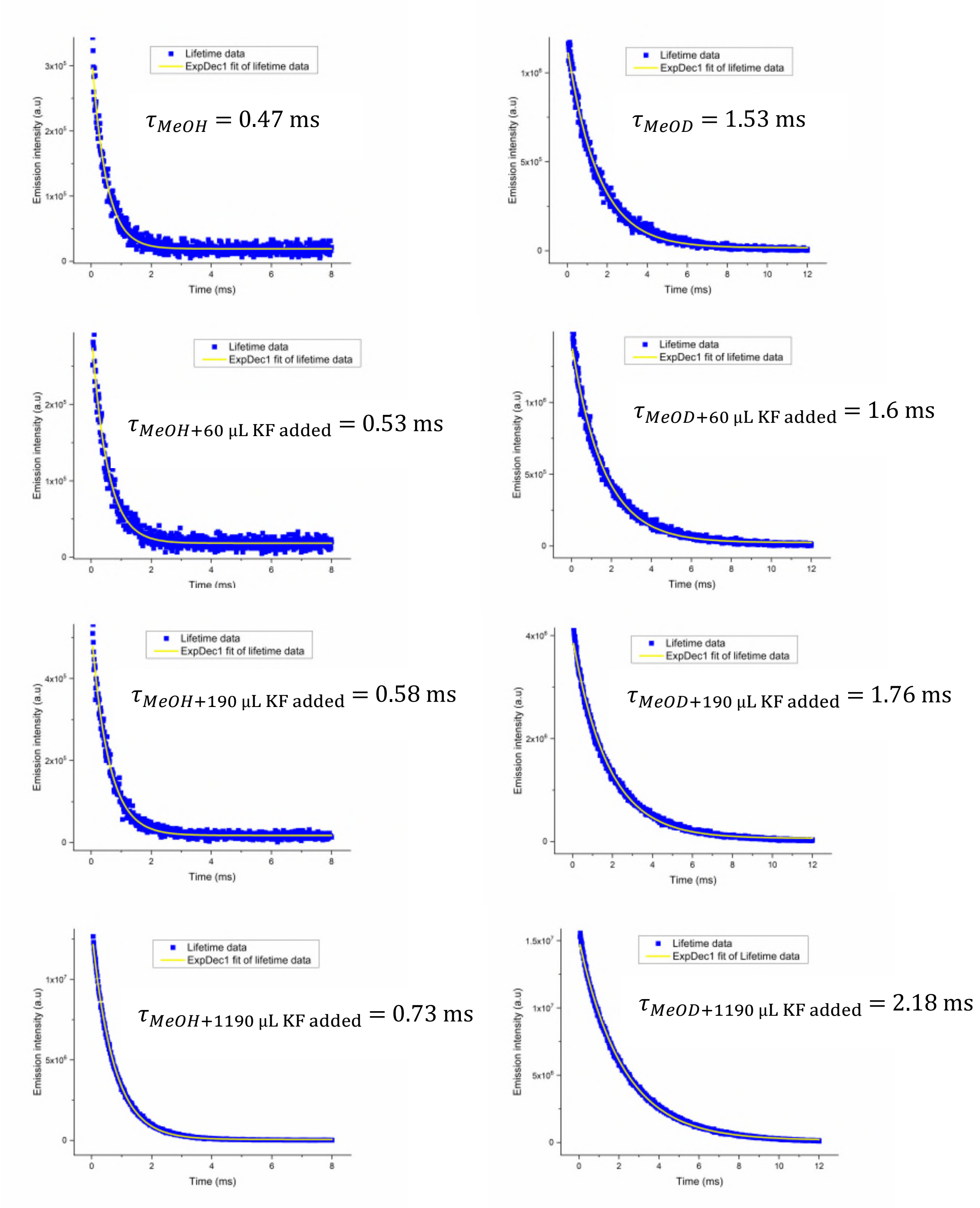

Figure S111: Time-resolved luminescence lifetime of [Eu_2(**DO3A**)$_2$**C-2**] with increasing concentration of KF in methanol (*left*) and deuterated methanol (*right*); concentration of [Eu_2(**DO3A**)$_2$**C-2**] is 1 mM, concentration of stock solution of KF in [Eu_2(**DO3A**)$_2$**C-2**] is 0.02 M (non-dilution method used).

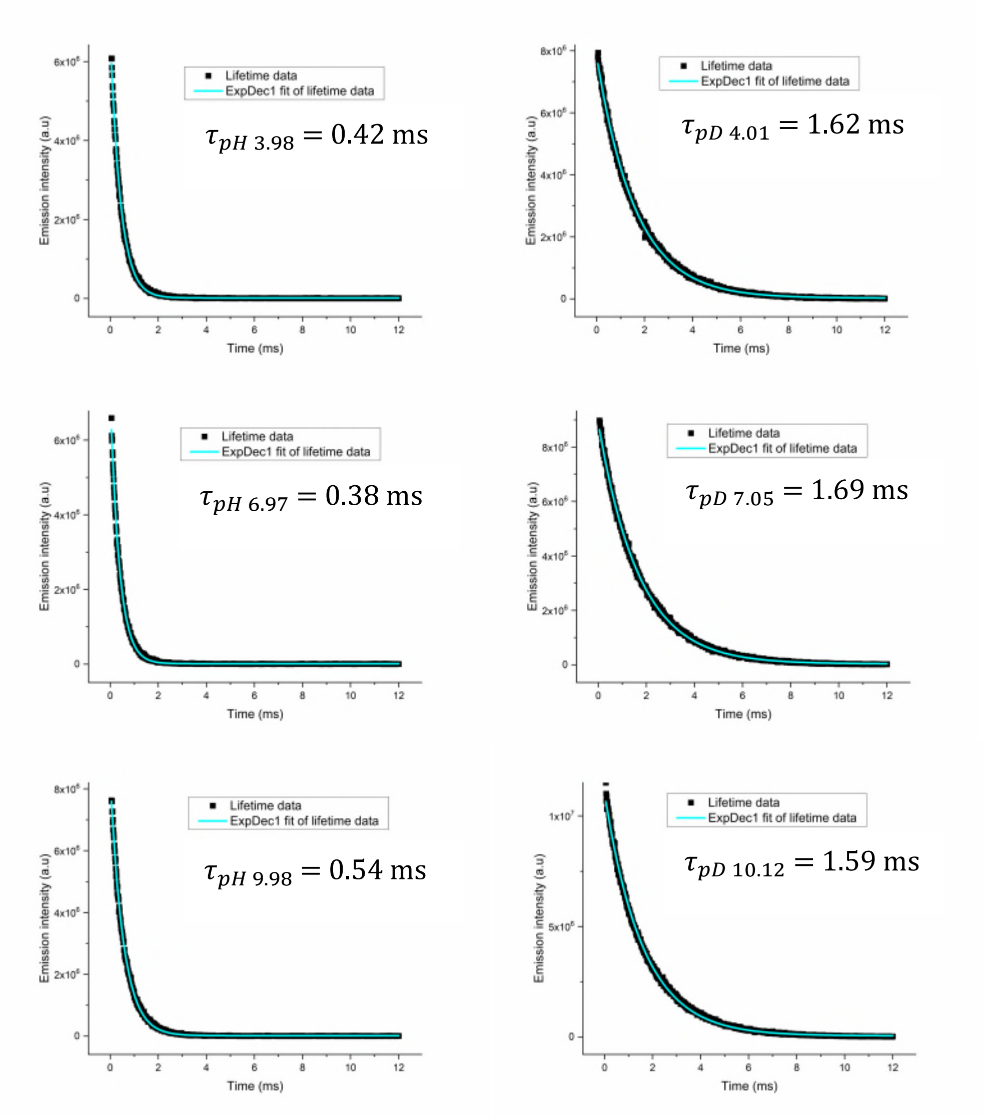

Figure S112: Time-resolved luminescence lifetime of [Eu_2(**DO3A**)$_2$**C-2**] at different pH (*left*) and pD (*right*); concentration of [Eu_2(**DO3A**)$_2$**C-2**] is 1 mM.

Table S1: Luminescence lifetimes for [Eu_2(**DO3A**)$_2$**C-2**] at different pH and pD*

Complex in different pH/pD	Lifetime in H_2O (ms)	Lifetime in D_2O (ms)	Hydration number (*q*)
Complex in pH 3.98/pD 4.01	0.42	1.62	1.8
Complex in pH 6.97/pD 7.05	0.38	1.69	2.1
Complex in pH 9.98/pD 10.12	0.54	1.59	1.2

* All lifetime values are subjected to an error of ±10%.

Table S2: Luminescence lifetimes for [Eu_2(**DO3A**)$_2$**C-2**] with increasing concentration of KF in H_2O and D_2O*

Complex and amount of KF added	Lifetime in H_2O (ms)	Lifetime in D_2O (ms)	Hydration number (*q*)
Complex	0.31	1.55	2.8
Complex + 50 μL KF	0.57	1.7	1.1
Complex + 200 μL KF	0.6	1.71	1
Complex + 1000 μL KF	0.66	1.63	0.8

* All lifetime values are subjected to an error of ±10%.

Table S3: Luminescence lifetimes for [Eu_2(**DO3A**)$_2$**C-2**] with increasing concentration of KF in CH_3OH and CD_3OD*

Complex and amount of KF added	Lifetime in MeOH (ms)	Lifetime in MeOD (ms)	Hydration number (*q*)
Complex	0.47	1.53	3.2
Complex + 60 μL KF	0.53	1.6	2.8
Complex + 190 μL KF	0.58	1.76	2.5
Complex + 1190 μL KF	0.73	2.18	1.9

* All lifetime values are subjected to an error of ±10%.

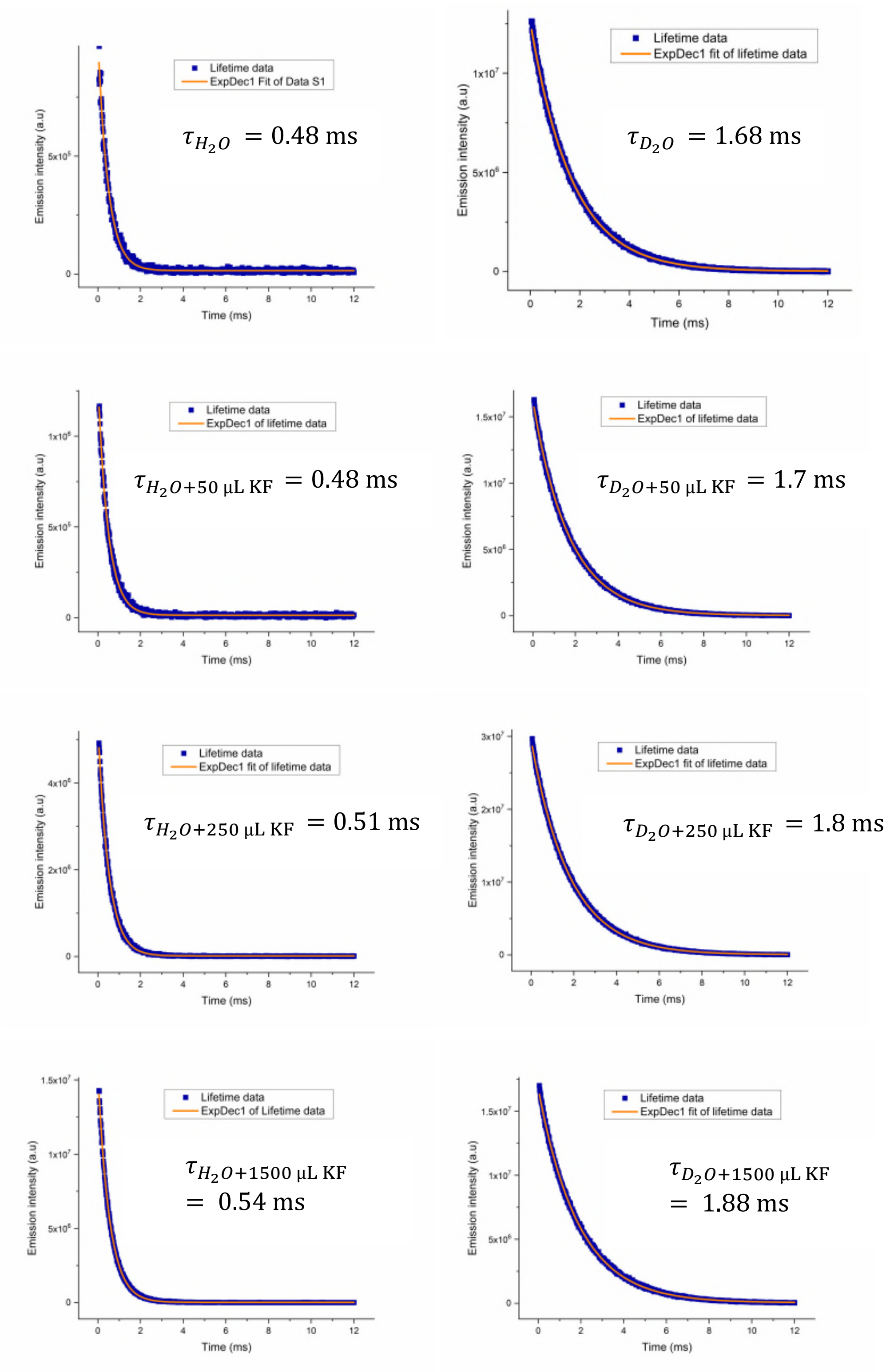

Figure S113: Time-resolved luminescence lifetime of [Eu2(**DO3A**)2**C-3**] with increasing concentration of KF in water (*left*) and deuterium oxide (*right*); concentration of [Eu2(**DO3A**)2**C-3**] is 1 mM, concentration of stock solution of KF in [Eu2(**DO3A**)2**C-3**] is 0.02 M (non-dilution method used).

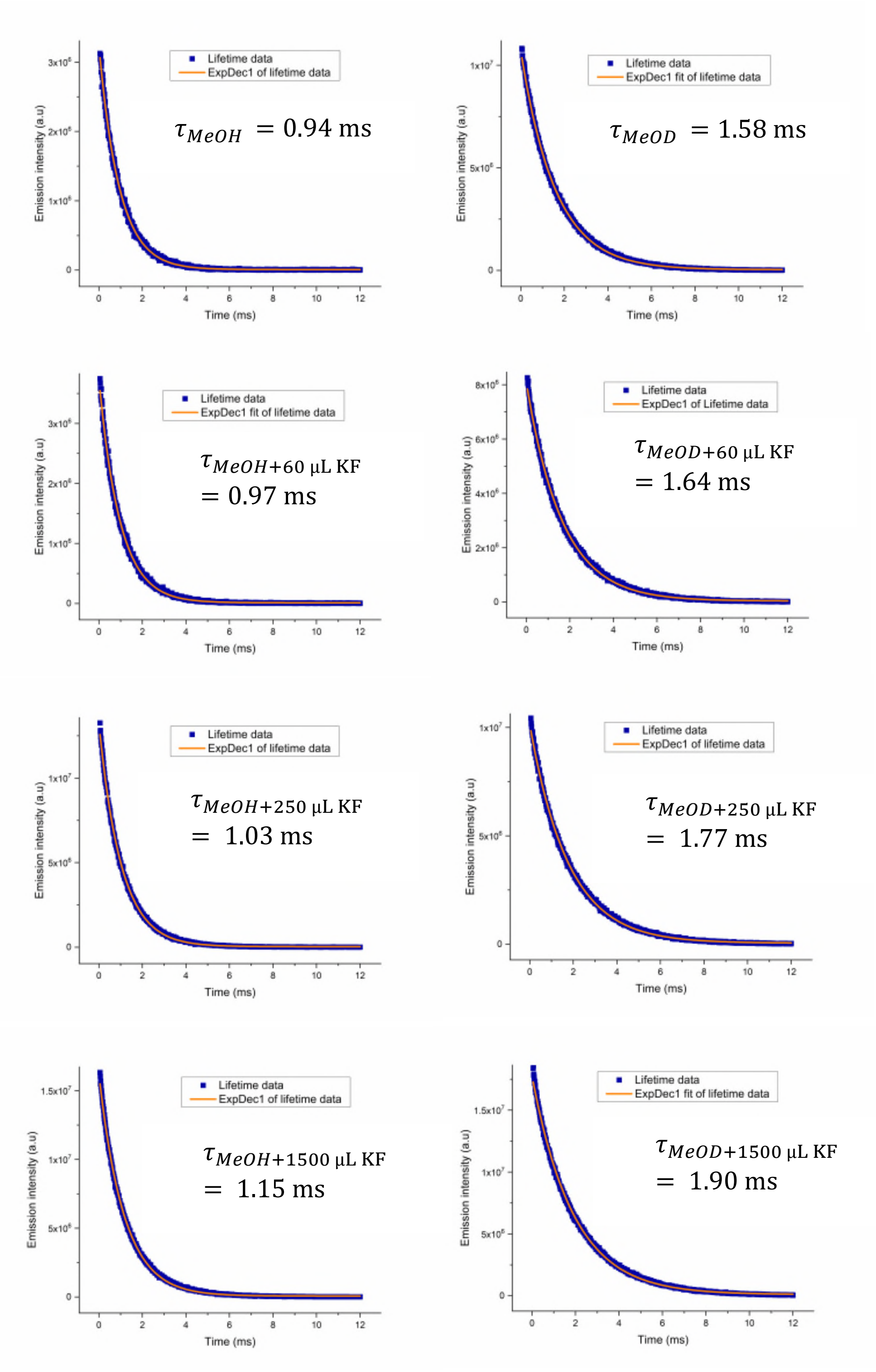

Figure S114: Time-resolved luminescence lifetime of [Eu2(**DO3A**)2**C-3**] with increasing concentration of KF in methanol (*left*) and deuterated methanol (*right*); concentration of [Eu2(**DO3A**)2**C-3**] is 1 mM, concentration of stock solution of KF in [Eu2(**DO3A**)2**C-3**] is 0.02 M (non-dilution method used).

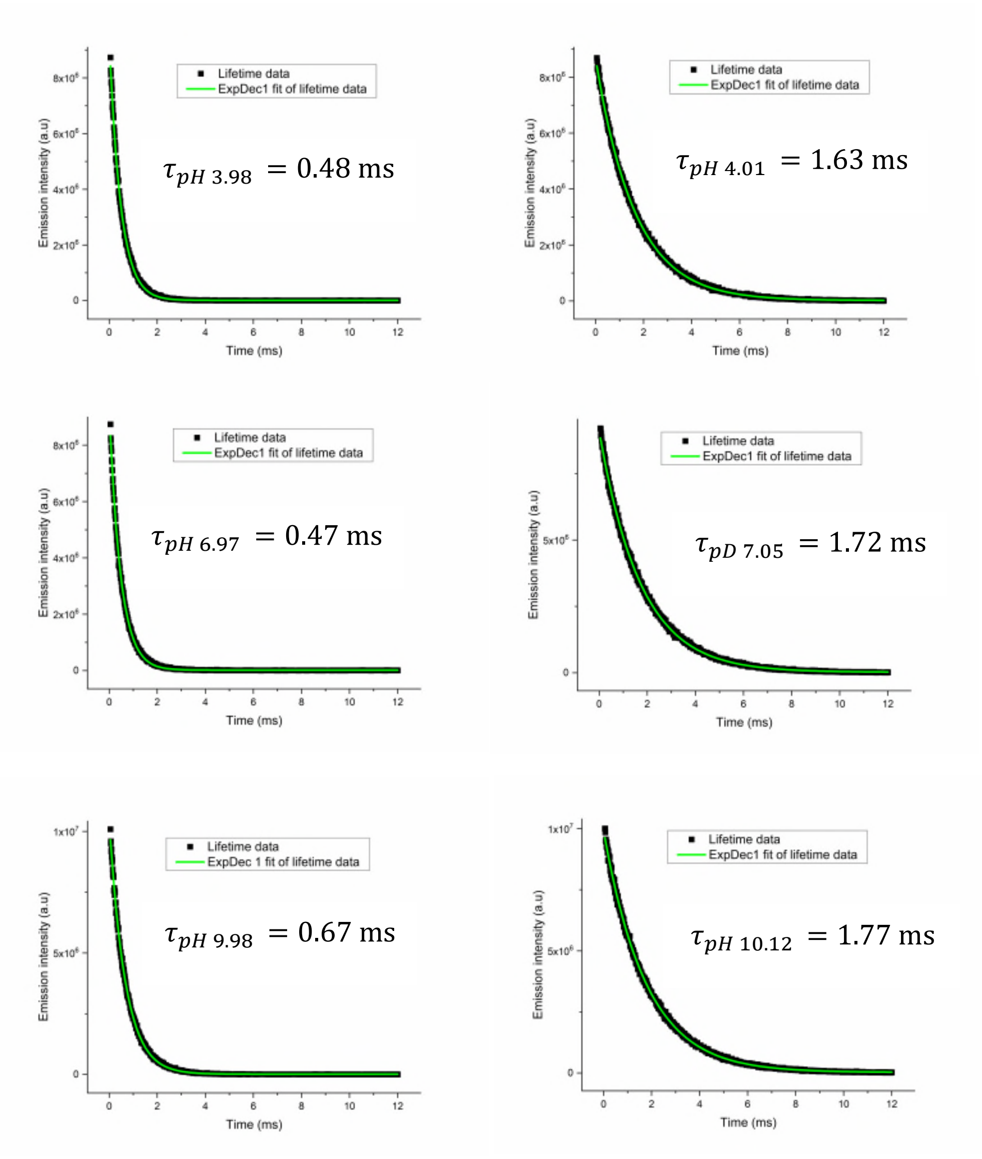

Figure S115: Time-resolved luminescence lifetime of [Eu_2(**DO3A**)$_2$**C-3**] at different pH (*left*) and pD (*right*); concentration of [Eu_2(**DO3A**)$_2$**C-3**] is 1 mM.

Table S4: Luminescence lifetimes for [Eu_2(**DO3A**)$_2$**C-3**] at different pH and pD*

Complex in different pH/pD	Lifetime in H_2O (ms)	Lifetime in D_2O (ms)	Hydration number (*q*)
Complex in pH 3.98/pD 4.01	0.48	1.63	1.5
Complex in pH 6.97/pD 7.05	0.47	1.72	1.5
Complex in pH 9.98/pD 10.12	0.67	1.77	0.8

* All lifetime values are subjected to an error of ±10%.

Table S5: Luminescence lifetimes for [Eu_2(**DO3A**)$_2$**C-3**] with increasing concentration of KF in H_2O and D_2O*

Complex and amount of KF added	Lifetime in H_2O (ms)	Lifetime in D_2O (ms)	Hydration number (*q*)
Complex	0.48	1.68	1.5
Complex + 50 μL KF	0.48	1.7	1.5
Complex + 250 μL KF	0.51	1.8	1.4
Complex + 1500 μL KF	0.54	1.88	1.3

* All lifetime values are subjected to an error of ±10%.

Table S6: Luminescence lifetimes for [Eu_2(**DO3A**)$_2$**C-3**] with increasing concentration of KF in CH_3OH and CD_3OD*

Complex and amount of KF added	Lifetime in MeOH (ms)	Lifetime in MeOD (ms)	Hydration number (*q*)
Complex	0.94	1.58	0.73
Complex + 60 μL KF	0.97	1.64	0.7
Complex + 200 μL KF	1.03	1.77	0.6
Complex + 1500 μL KF	1.15	1.90	0.5

* All lifetime values are subjected to an error of ±10%.

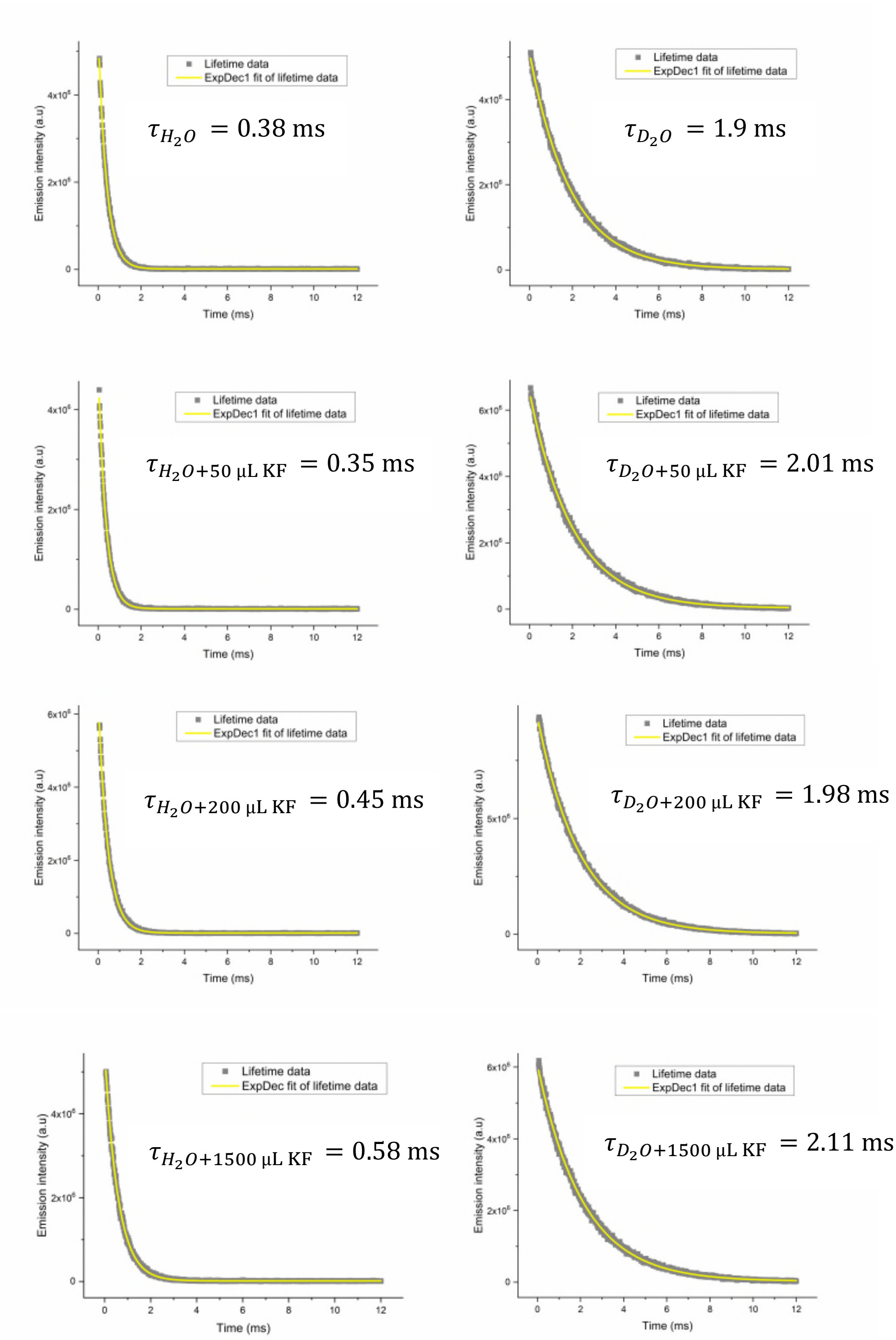

Figure S116: Time-resolved luminescence lifetime of [Eu(**pDO3A**)] with increasing concentration of KF in water (*left*) and deuterium oxide (*right*); concentration of [Eu(**pDO3A**)] is 1 mM, concentration of stock solution of KF in [Eu(**pDO3A**)] is 0.02 M (non-dilution method used).

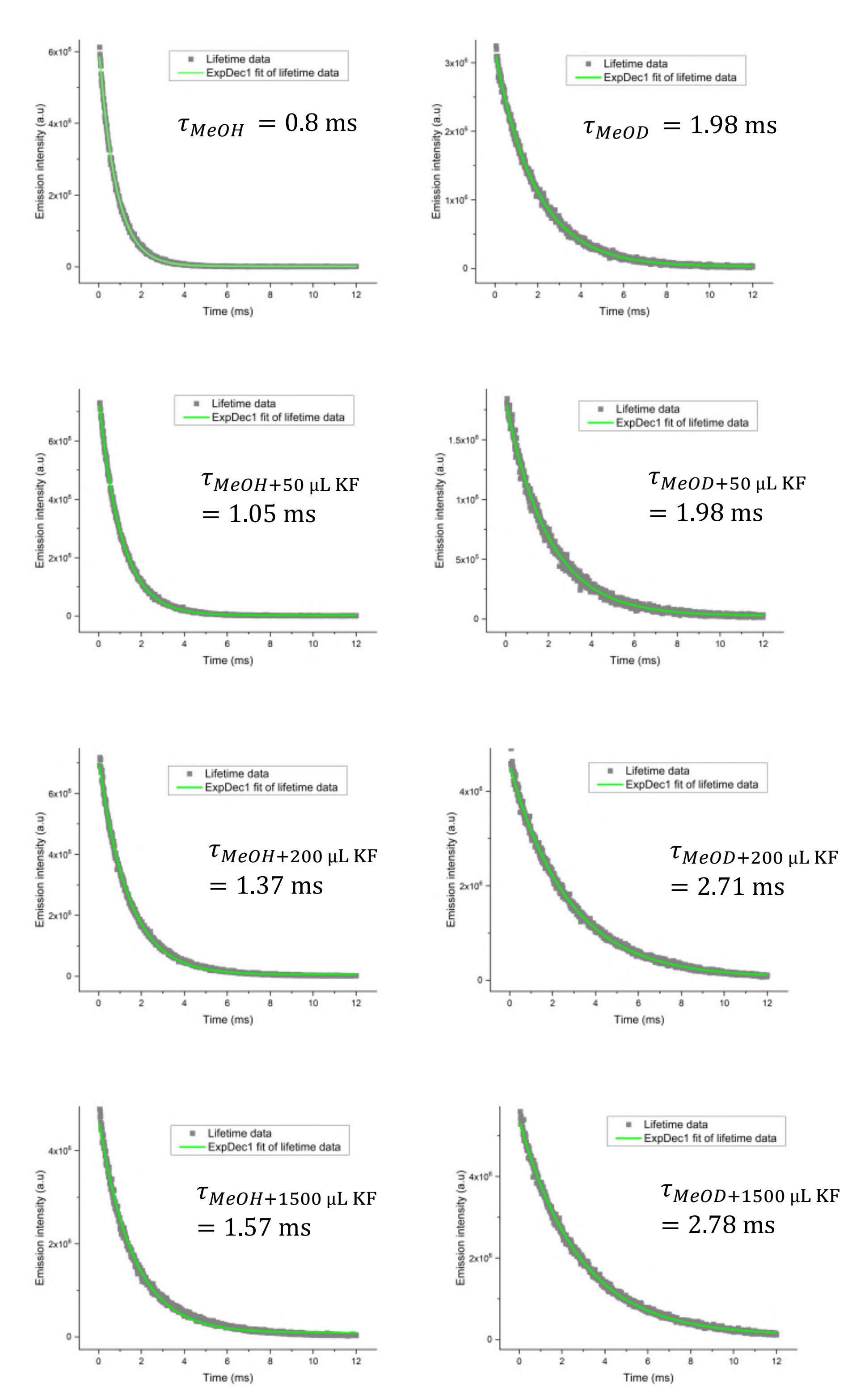

Figure S117: Time-resolved luminescence lifetime of [Eu(**pDO3A**)] with increasing concentration of KF in methanol (*left*) and deuterated methanol (*right*); concentration of [Eu(**pDO3A**)] is 1 mM, concentration of stock solution of KF in [Eu(**pDO3A**)] is 0.02 M (non-dilution method used).

Table S7: Luminescence lifetimes for [Eu(**pDO3A**)] with increasing concentration of KF in H_2O and D_2O*

Complex and amount of KF added	Lifetime in H_2O (ms)	Lifetime in D_2O (ms)	Hydration number (*q*)
[Eu(**pDO3A**)]	0.38	1.98	2.2
[Eu(**pDO3A**)] + 50 μL KF	0.35	2.01	2.5
[Eu(**pDO3A**)] + 200 μL KF	0.45	1.98	1.7
[Eu(**pDO3A**)] + 1500 μL KF	0.59	2.11	1.2

* All lifetime values are subjected to an error of ±10%.

Table S8: Luminescence lifetimes for [Eu(**pDO3A**)] with increasing concentration of KF in CH_3OH and CD_3OD*

Complex and amount of KF added	Lifetime in MeOH (ms)	Lifetime in MeOD (ms)	Hydration number (*q*)
[Eu(**pDO3A**)]	0.8	1.9	1.4
[Eu(**pDO3A**)] + 50 μL KF	1.05	2	0.8
[Eu(**pDO3A**)] + 200 μL KF	1.37	2.71	0.6
[Eu(**pDO3A**)] + 1500 μL KF	1.57	2.8	0.4

* All lifetime values are subjected to an error of ±10%.

9. Limits of Detection and Quantification with KCl in Water

The limit of detection (LOD) and limit of quantification (LOQ) is calculated using the formula

$$Limits\ of\ detection\ and\ quantification = \frac{p \times \sigma}{b}$$

where, $p = 3$ for LoD and 10 for LoQ,[15] σ = standard deviation of the luminescence emission bands corresponding to the transitions (ΔJ = 2 / ΔJ =1) from neat complex solution in water (four measurements used) and b = slope of luminescence emission (ΔJ = 2 / ΔJ =1) upon the addition of guest in water (M^{-1}).

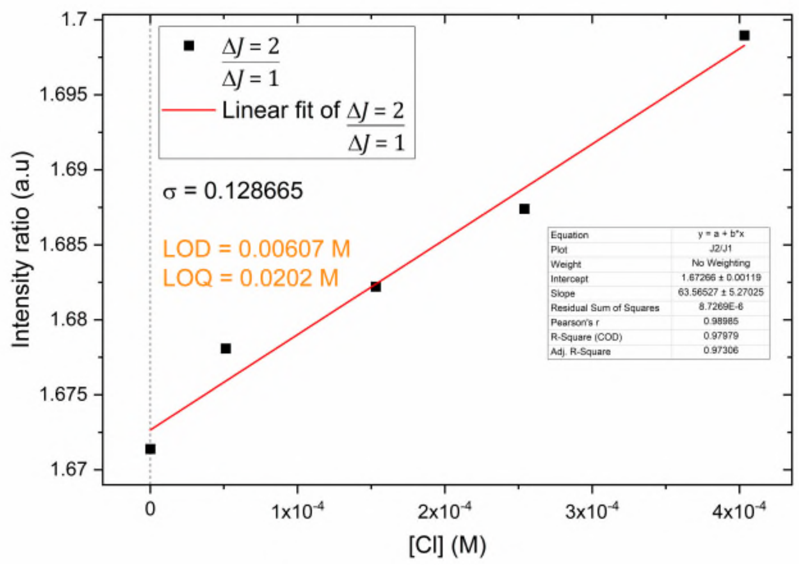

Figure S118: LOD and LOQ plots for [Eu2(**DO3A**)2**C-2**] with KCl in deionised water at 22 °C.

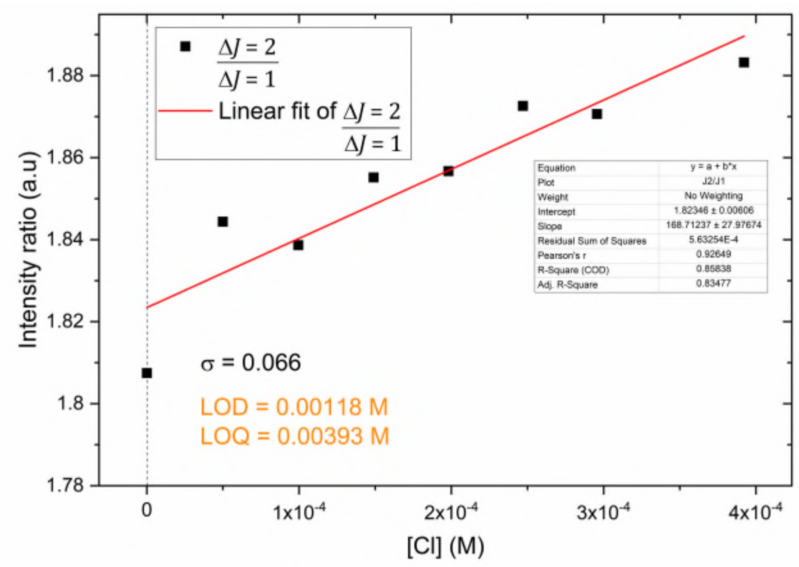

Figure S119: LOD and LOQ plots for [Eu2(**DO3A**)2**C-3**] with KCl in deionised water at 22 °C.

10. DYNAFIT Script

10.1 A typical DYNAFIT script for a single binding event

A typical DYNAFIT script for a single binding event is:

```
[task]
   task = fit  ;
   data = equilibria  ;
[mechanism]
   Eu + Cl <==> EuCl : K1 assoc
[constants]
   K1 = 10E4??
[concentrations]
   Eu = 1.00E-03?
[data]
   variable Cl
file .\CA_E71_01_Eu_PB_titration_Cl\CA_E71_01_PB_titration_Cl.txt |response
Eu = 1.19E+04?, EuCl = 1.48E+04?
[output]
directory .\Desktop\DYNAFIT4\Results
[end]
```

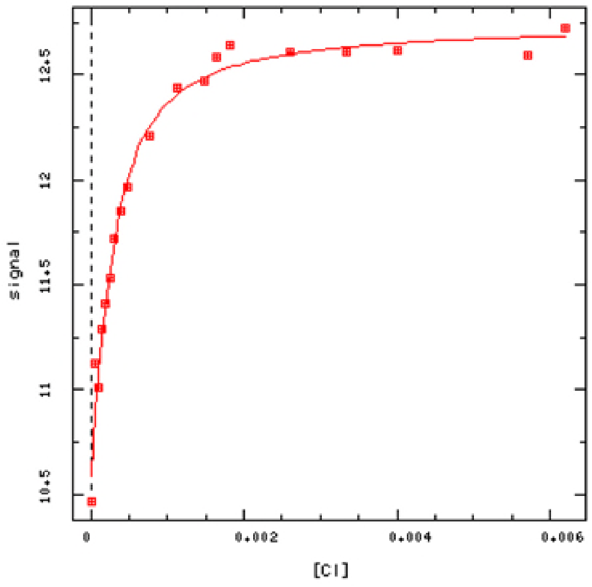

Figure S120: A typical binding isotherm generated from DYNAFIT® software for a single binding event.

10.2 A typical DYNAFIT script for a two binding event

```
Two event binding - peak sum
[task]
   data = equil
   task = fit
[mechanism]
   Eu + F <==> EuF : K1 assoc
   EuF + F <==> EuFF : K2 assoc
[constants]
   K1 = 100000?
   K2 = 10000?
[concentrations]
   Eu = 9.99E-04?
[responses]
[equil]
   variable F
file    .\CA_E71_01_Eu_water_titration_F\CA_E71_01_Eu_water_titration_F.txt
|response Eu = 1.21E+04?, EuF = 1.14E+04?, EuFF = 1.08E+04?
[output]
directory .\DYNAFIT4\Results
[end]
```

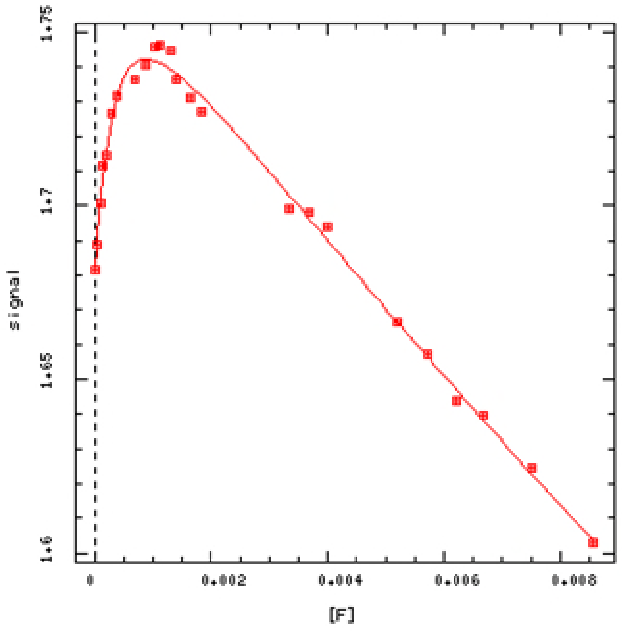

Figure S121: A typical binding isotherm generated from DYNAFIT® software for a two-binding event.

10.3 Speciation modelling

Speciation models were generated using DYNAFIT® by employing the data used to produce the binding isotherm. A modification of the EQUIL algorithm[16a] has been implemented in DYNAFIT® as reported in the literature to generate speciation models.[16b,16c]

11. X-ray Crystal Structures of Ligands and Complexes

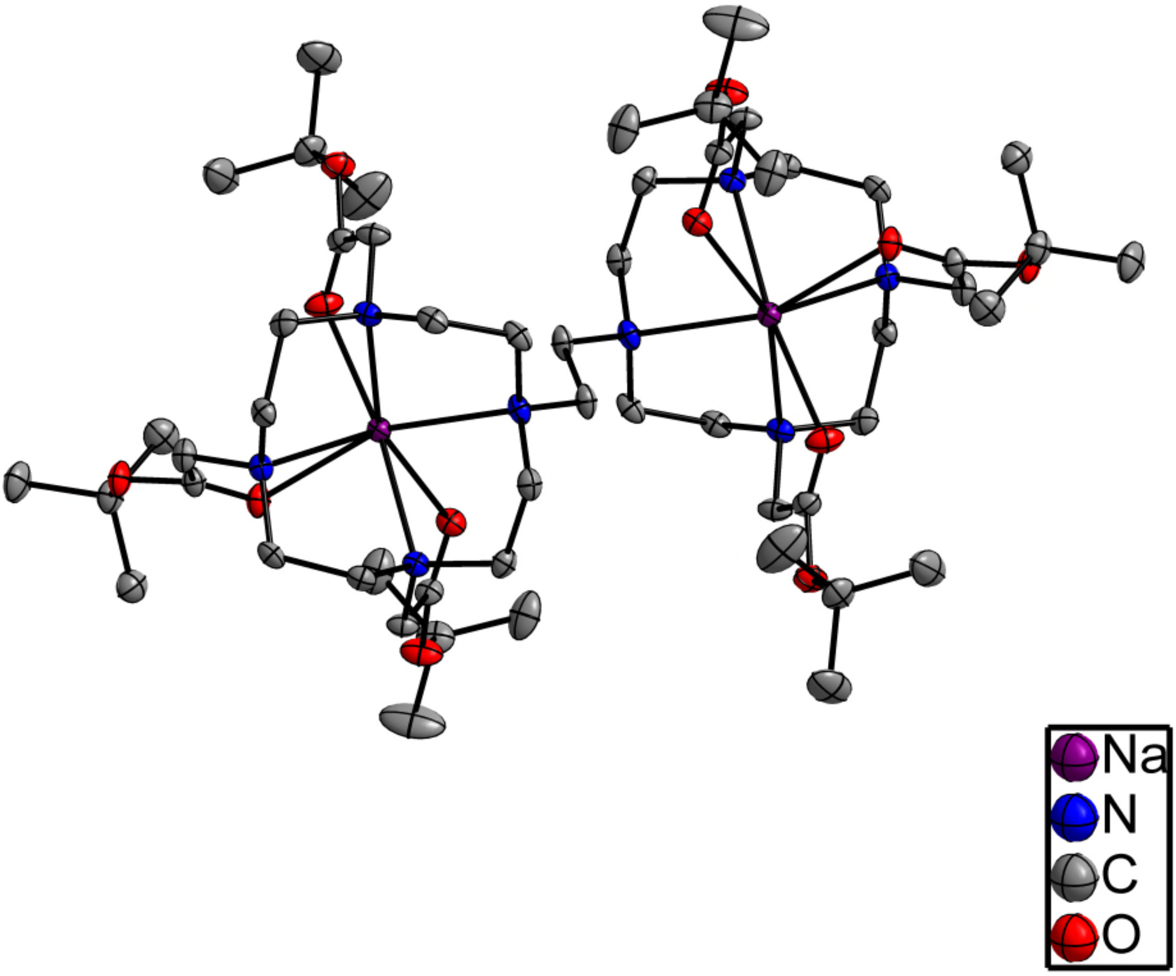

Figure S122: Single crystal X-ray structure of (DO3A(*t*-BuO$_3$))$_2$C-2. H atoms, Br atoms, and solvent molecules are omitted for clarity. Thermal ellipsoid drawn at 30% probability level.

Table S9: Crystal data and structure refinement for (DO3A(*t*-BuO)$_3$)$_2$C-2

**Parameters**	**(DO3A(*t*-BuO)$_3$)$_2$C-2**	
CCDC No.	2201928	
Empirical formula	$C_{60}H_{108}Br_2Cl_{18}N_8Na_2O_{12}$	
Formula weight	1977.49	
Temperature	150 K	
Wavelength	1.54180 Å	
Crystal system	Triclinic	
Space group	*P*-1	
Unit cell dimensions	*a* = 12.4765(3) Å	*α* = 71.979(2)°
	b = 13.7256(3) Å	*β* = 88.7149(18)°
	c = 15.0782(3) Å	*γ* = 78.9608(19)°
Volume	2408.12(10) Å^3	
Z	1	
Density (calculated)	1.364 Mg/m^3	
Absorption coefficient	6.175 mm^{-1}	
F(000)	1017.993	
Crystal size	0.20 × 0.14 × 0.10 mm^3	
Theta range for data collection	3.612° to 75.987°	
Index ranges	-15<=h<=15, -17<=k<=17, -18<=l<=18	
Reflections collected	50946	
Independent reflections	9968 [*R*(int) = 0.044]	
Completeness to theta = 73.257°	99.9%	
Absorption correction	Semi-empirical from equivalents	
Max. and min. transmission	0.54 and 0.35	
Refinement method	Full-matrix least-squares on F^2	
Data / restraints / parameters	9966 / 12 / 473	
Goodness-of-fit on F^2	0.9928	
Final *R* indices [$I > 2\sigma(I)$]	*R*1 = 0.0474, w*R*2 = 0.1308	
R indices (all data)	*R*1 = 0.0529, w*R*2 = 0.1363	
Largest diff. peak and hole	1.79 and -0.81 e.Å^{-3}	

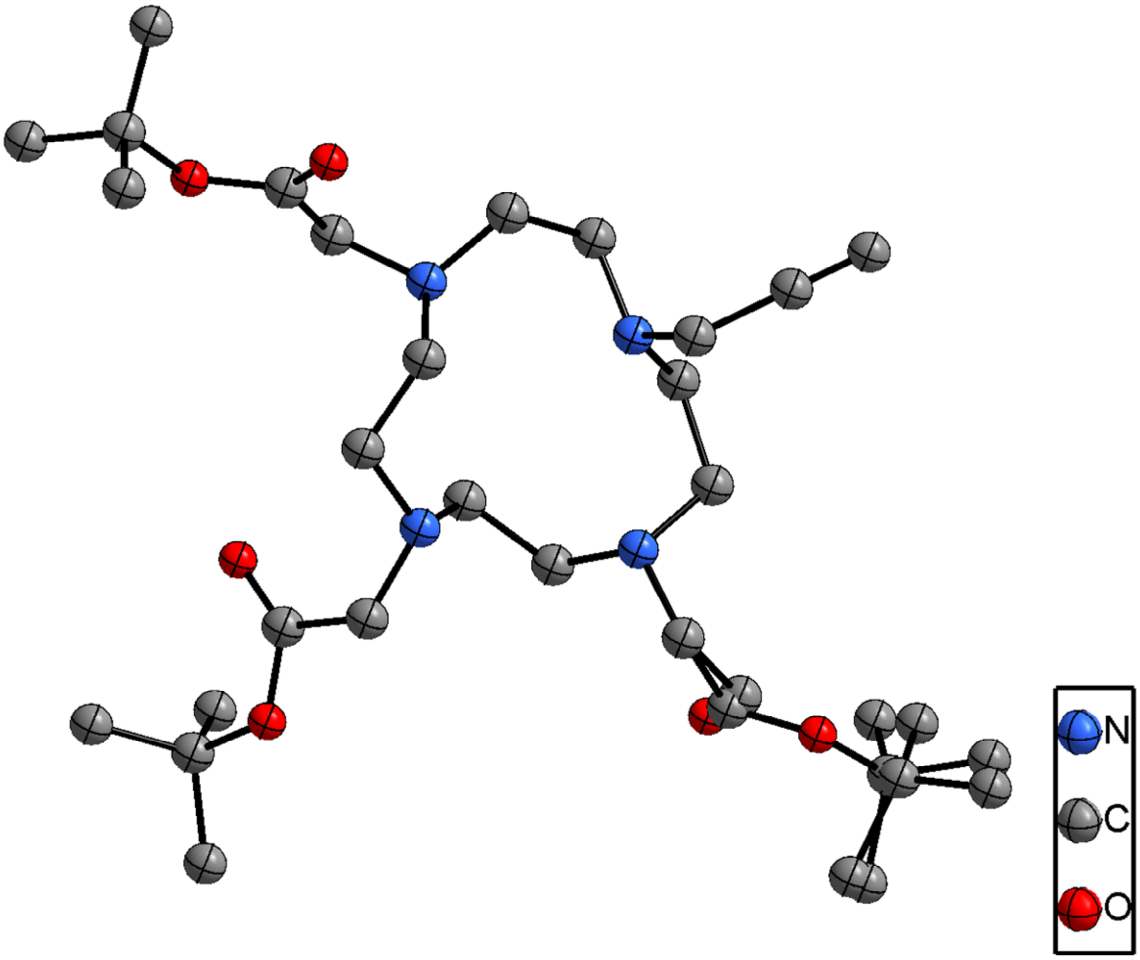

Figure S123: Single crystal X-ray structure of pDO3A(*t*-BuO$_3$). H atoms are omitted for clarity. Thermal ellipsoid drawn at the 30% probability level.

Table S10: Crystal data and structure refinement for pDO3A(*t*-BuO)$_3$

**Parameters**	**pDO3A(*t*-BuO)$_3$**	
CCDC No.	2201929	
Empirical formula	$C_{29}H_{52}N_4O_6$	
Formula weight	552.75	
Temperature (K)	150	
Wavelength	1.54184 Å	
Crystal system	Monoclinic	
Space group	*I*2/a	
Unit cell dimensions	*a* = 25.8831(10) Å	α = 90°
	b = 5.6524(4) Å	β = 94.085(4)°
	c = 43.780(2) Å	γ = 90°
Volume	6388.8(6) Å^3	
Z	8	
Density (calculated	1.149 Mg/m^3	
Absorption coefficient	0.646 mm^{-1}	
F(000)	2416	
Crystal size	0.15 × 0.05 × 0.02 mm^3	
Theta range for data collection	3.424 to 77.581°.	
Index ranges	-32<=h<=29, -6<=k<=6, -54<=l<=55	
Reflections collected	36069	
Independent reflections	6615 [*R*(int) = 0.079]	
Completeness to theta = 73.702°	99.6%	
Absorption correction	Semi-empirical from equivalents	
Max. and min. transmission	0.99 and 0.96	
Refinement method	Full-matrix least-squares on F^2	
Data / restraints / parameters	6615 / 396 / 374	
Goodness-of-fit on F^2	0.9888	
Final *R* indices [$I>2\sigma(I)$]	*R*1 = 0.0553, w*R*2 = 0.1266	
R indices (all data)	*R*1 = 0.0867, w*R*2 = 0.1556	
Largest diff. peak and hole	0.48 and -0.58 e.Å^{-3}	

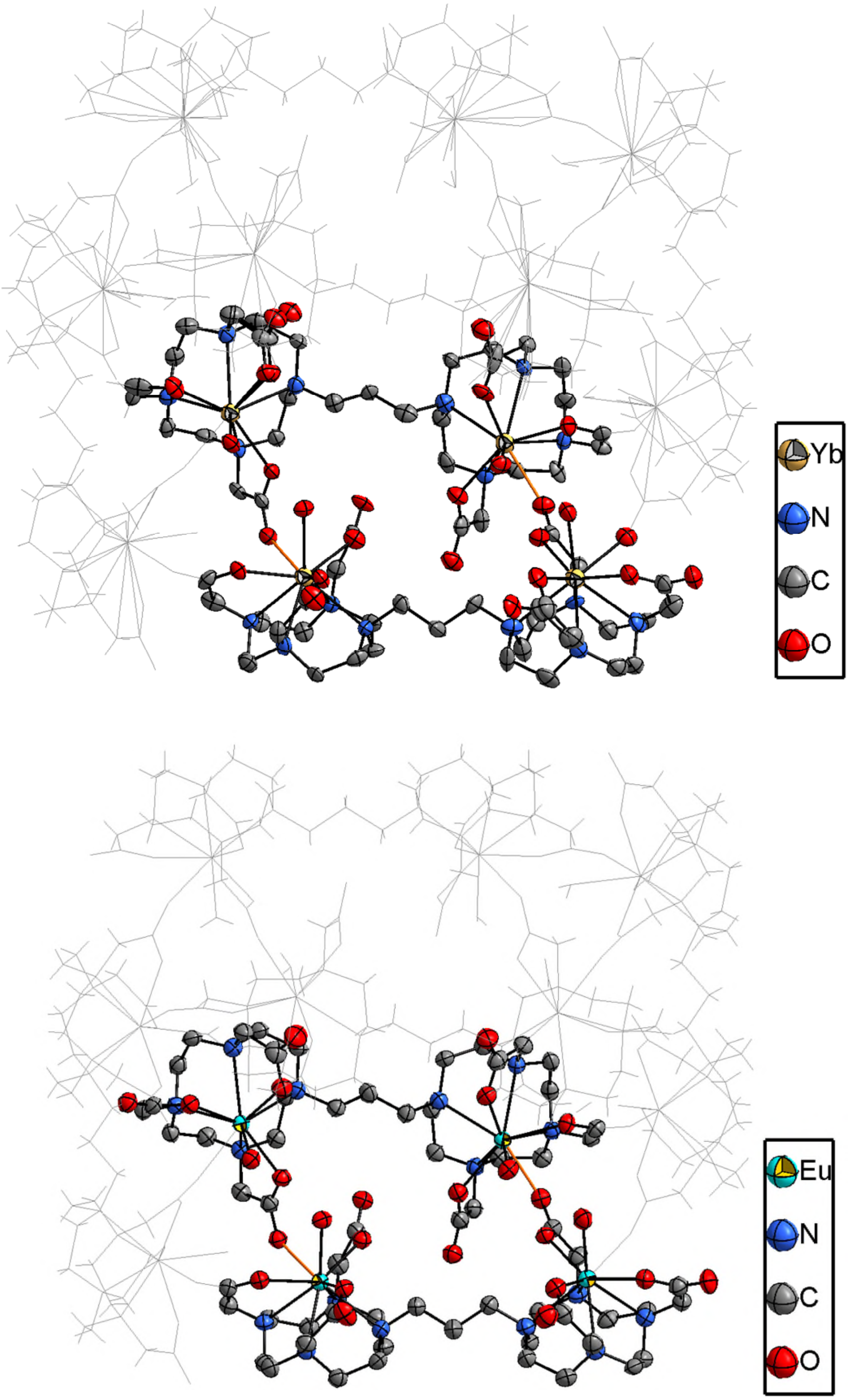

Figure S124: Single crystal X-ray structure of [Yb_2(**DO3A**)$_2$**C-3**] (*top*) and [Eu_2(**DO3A**)$_2$**C-3**] (*bottom*). Except selected structure, the rest are shown in wireframe format, H atoms and water are omitted for clarity. Thermal ellipsoid drawn at 30% probability level. μ-oxo bridging bond is shown in orange.

Table S11: Crystal data and structure refinement for [Ln_2(**DO3A**)$_2$**C-3**] [Ln = Eu(III), Yb(III)]

**Parameters**	[Eu_2(**DO3A**)$_2$**C-3**]	[Yb_2(**DO3A**)$_2$**C-3**]
CCDC No.	2201930	2201931
Empirical formula	C_{186} H_{324} Eu_{12} N_{48} O_{84}	C_{186} H_{316} N_{48} O_{84} Yb_{12}
Formula weight	6400.45	6645.35
Temperature (K)	150	150
Wavelength	1.54180 Å	1.54180 Å
Crystal system	Monoclinic	Monoclinic
Space group	$P2_1$/n	$P2_1$/n
Unit cell dimensions	*a* = 28.5577(5) Å	*a* = 28.4973(7) Å
	b = 15.9841(2) Å	*b* = 15.9022(3) Å
	c = 39.9615(6) Å	*c* = 39.7702(8) Å
	α = 90°	α = 90°
	β = 103.3460(16)°	β = 103.219(2)°
	γ = 90°	γ = 90°
Volume	17748.6(5) $Å^3$	17545.1(7) $Å^3$
Z	2	2
Density (calculated)	1.198 Mg/m^3	1.258 Mg/m^3
Absorption coefficient	15.446 mm^{-1}	6.187 mm^{-1}
F(000)	6408	6560
Crystal size	0.18 × 0.11 × 0.03 mm^3	0.18 × 0.15 × 0.04 mm^3
Theta range for data collection	3.181 to 76.454°	3.597 to 43.217°.
Index ranges	-35<=h<=34, -20<=k<=9, -49<=l<=50	-25<=h<=25, -14<=k<=14, -34<=l<=35
Reflections collected	105177	158465
Independent reflections	36621 [*R*(int) = 0.068]	12717 [*R*(int) = 0.110]
Completeness to theta	99.7% (73.257°)	99.6% (41.921°)
Absorption correction	Semi-empirical from equivalents	Semi-empirical from equivalents
Max. and min. transmission	0.63 and 0.02	0.78 and 0.26
Refinement method	Full-matrix least-squares on F^2	Full-matrix least-squares on F^2
Data / restraints / parameters	36617 / 0 / 1486	12655 / 244 / 1512
Goodness-of-fit on F^2	1.0191	1.0303
Final *R* indices [I>2σ(I)]	*R*1 = 0.1126, w*R*2 = 0.2713	*R*1 = 0.0701, w*R*2 = 0.1987
R indices (all data)	*R*1 = 0.1305, w*R*2 = 0.3036	*R*1 = 0.0851, w*R*2 = 0.2144
Largest diff. peak and hole	5.22 and -1.07 e.$Å^{-3}$	1.86 and -1.73 e.$Å^{-3}$

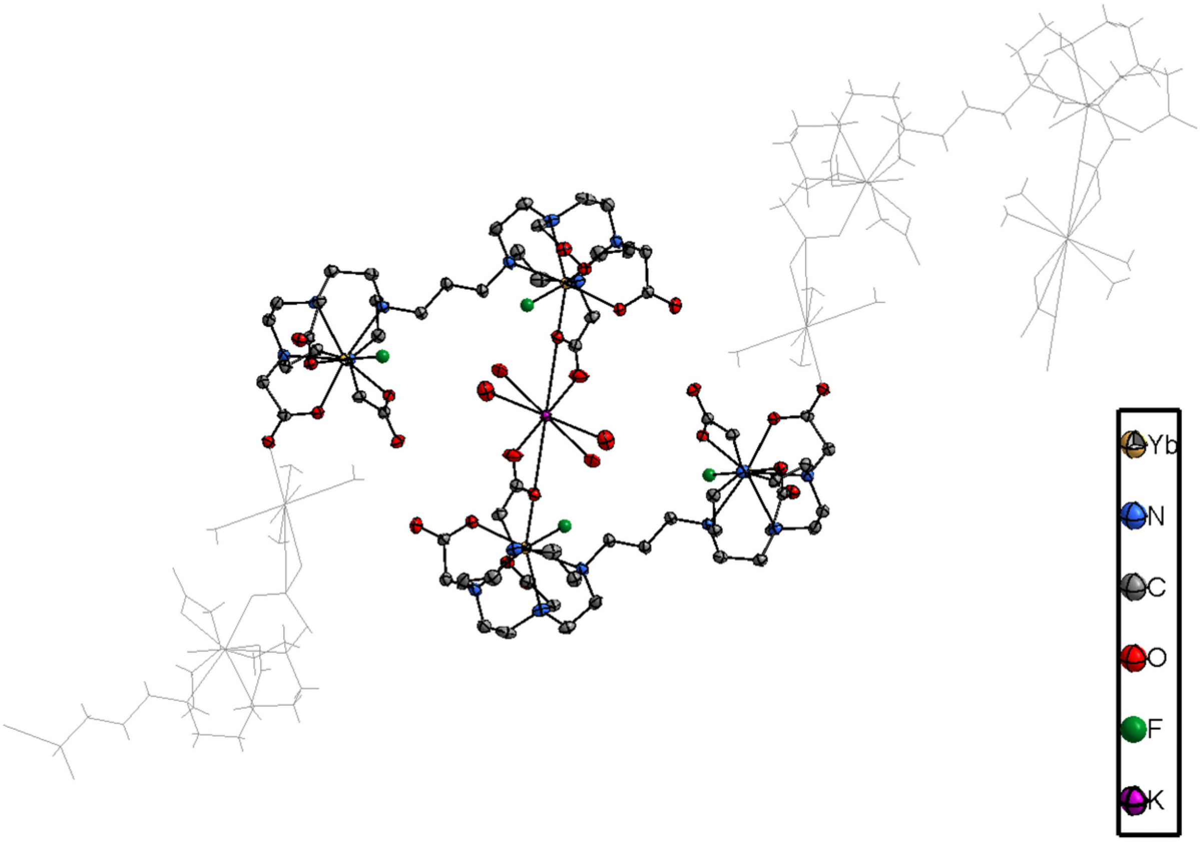

Figure S125: Single crystal X-ray structure of $[Yb_2($**DO3A**$)_2$**C-3**(2F)$]^{2-}$. Except selected structure, the rest are shown in wireframe format, H atoms and water are omitted for clarity. Thermal ellipsoid drawn at 30% probability level.

Table S12: Crystal data and structure refinement for [Yb_2(**DO3A**)$_2$**C-3**(2F)]$^{2-}$

**Parameters**	[Yb_2(**DO3A**)$_2$**C-3**(2F)]$^{2-}$	
CCDC No.	2201932	
Empirical formula	$C_{31} H_{58} F_2 K N_8 O_{16} Yb_2$	
Formula weight	1222.02	
Temperature	150 K	
Wavelength	1.54184 Å	
Crystal system	Triclinic	
Space group	*P*-1	
Unit cell dimensions	*a* = 12.6782(2) Å	*α* = 94.8655(15)°
	b = 13.2543(2) Å	*β* = 102.8002(15)°
	c = 17.7146(3) Å	*γ* = 108.0577(16)°
Volume	2721.73(8) Å^3	
Z	2	
Density (calculated)	1.491 Mg/m^3	
Absorption coefficient	7.456 mm^{-1}	
F(000)	1210	
Crystal size	0.18 × 0.10 × 0.08 mm^3	
Theta range for data collection	3.557 to 76.293°	
Index ranges	-15<=h<=13, -16<=k<=16, -21<=l<=22	
Reflections collected	49071	
Independent reflections	11279 [R(int) = 0.030]	
Completeness to theta = 74.767°	99.6%	
Absorption correction	Semi-empirical from equivalents	
Max. and min. transmission	0.55 and 0.11	
Refinement method	Full-matrix least-squares on F^2	
Data / restraints / parameters	11277 / 0 / 544	
Goodness-of-fit on F^2	1.0117	
Final *R* indices [I>2σ(I)]	*R*1 = 0.0548, w*R*2 = 0.1571	
R indices (all data)	*R*1 = 0.0565, w*R*2 = 0.1598	
Largest diff. peak and hole	2.88 and -2.05 e.Å^{-3}	

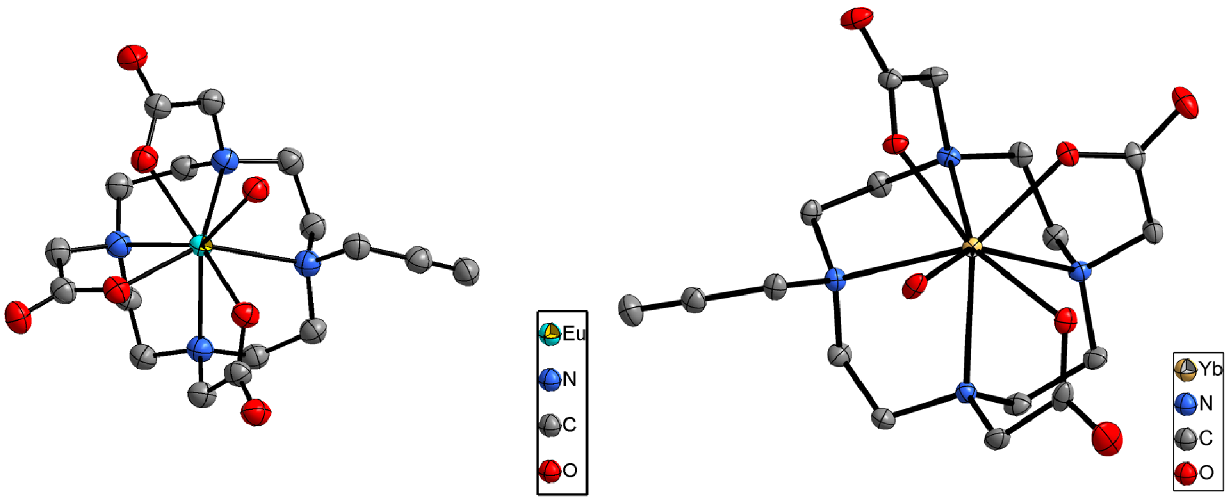

Figure S126: Single crystal X-ray structure of [Eu(**pDO3A**)] (*left*) and [Yb(**pDO3A**)] (*right*). H atoms and water omitted for clarity. Thermal ellipsoid drawn at 30% probability level.

Table S13: Crystal data and structure refinement for [Ln(**pDO3A**)] [Ln = Eu(III), Yb(III)]

**Parameters**	[Eu(**pDO3A**)]	[Yb(**pDO3A**)]
CCDC No.	2201933	2201934
Empirical formula	C_{17} H_{31} Eu N_4 O_9	C_{17} H_{31} Yb N_4 O_9
Formula weight	587.41	608.49
Temperature (K)	150	150
Wavelength	1.54184 Å	1.54184 Å
Crystal system	Monoclinic	Monoclinic
Space group	$P2_1/n$	$P2_1/n$
Unit cell dimensions	a = 10.79910(10) Å	a = 10.8216(2) Å
	b = 18.3916(2) Å	b = 18.3994(3) Å
	c = 11.20760(10) Å	c = 11.1809(2) Å
	α = 90°	α = 90°
	β = 106.5527(13)°	β = 106.757(2)°
	γ = 90°	γ = 90°
Volume	2133.72(4) $Å^3$	2131.71(7) $Å^3$
Z	4	4
Density (calculated)	1.828 Mg/m^3	1.896 Mg/m^3
Absorption coefficient	21.554 mm^{-1}	8.627 mm^{-1}
F(000)	1184	1212
Crystal size	0.29 × 0.15 × 0.02 mm^3	0.50 × 0.20 × 0.05 mm^3
Theta range for data collection	4.767 to 76.441°	4.779 to 76.317°
Index ranges	-13<=h<=13, -23<=k<=23, -13<=l<=14	-12<=h<=13, -23<=k<=22, -13<=l<=13
Reflections collected	52630	12304
Independent reflections	4458 [R(int) = 0.043]	4418 [R(int) = 0.024]
Completeness to theta	99.8% (74.912°)	99.5% (41.921°)
Absorption correction	Semi-empirical from equivalents	Semi-empirical from equivalents
Max. and min. transmission	0.65 and 0.02	0.65 and 0.06
Refinement method	Full-matrix least-squares on F^2	Full-matrix least-squares on F^2
Data / restraints / parameters	4450 / 0 / 288	4417 / 0 / 288
Goodness-of-fit on F^2	1.0350	1.0237
Final R indices [$I>2\sigma(I)$]	$R1$ = 0.0734, w$R2$ = 0.1999	$R1$ = 0.0251, w$R2$ = 0.0648
R indices (all data)	$R1$ = 0.0738, w$R2$ = 0.2017	$R1$ = 0.0258, w$R2$ = 0.0653
Largest diff. peak and hole	4.10 and -0.85 e.$Å^{-3}$	1.13 and -0.96 e.$Å^{-3}$

12. References

1. J. Leonard, B. Lygo and G. Procter, Carrying out the reaction, In *Advanced Practical Organic Chemistry*, 3rd edition, CRC Press Taylor and Francis Group, USA, 2013, pp 137–189.
2. G. R. Fulmer, A. J. M. Miller, N. H. Sherden, H. E. Gottlieb, A. Nudelman, B. M. Stoltz, J. E. Bercaw and K. I. Goldberg, NMR Chemical shifts of trace impurities: Common laboratory solvents, organics, and gases in deuterated solvents relevant to the organometallic chemist, *Organometallics*, 2010, **29**, 2176–2179.
3. (a) L. Palatinus and G. Chapius, SUPERFLIP – a computer program for the solution of crystal structures by charge flipping in arbitrary dimensions, *J. Appl. Cryst.*, 2007, **40**, 786–790; (b) P. Parois, R. I. Cooper and A. L. Thompson, Crystal structures of increasingly large molecules: Meeting the challenges with CRYSTALS software, *Chem. Cent. J.*, 2015, **9**, 30; (c) R. I. Cooper, A. L. Thompson and D. J. Watkin, CRYSTALS enhancements: Dealing with hydrogen atoms in refinement, *J. Appl. Cryst.*, 2010, **43**, 1100–1107.
4. A. Beeby, I. M. Clarkson, R. S. Dickins, S. Faulkner, D. Parker, L. Royle, A. S. de Souza, J. A. G. Williams and M. Woods, Non-radiative deactivation of the excited states of europium, terbium and ytterbium complexes by proximate energy-matched OH, NH and CH oscillators: an improved luminescence method for establishing solution hydration states, *J. Chem. Soc., Perkin Trans. 2*, 1999, 493–504.
5. (a) P. K. Glasoe and F. A. Long, Use of glass electrodes to measure acidities in deuterium oxide, *J. Phys. Chem.*, 1960, **64**, 188–190; (b) A. Krężel and W. Bal, A formula for correlating pK_a values determined in D_2O and H_2O, *J. Inorg. Biochem.*, 2004, **98**, 161–166.
6. O. A. Blackburn, A. M. Kenwright, P. D. Beer and S. Faulkner, Axial fluoride binding by lanthanide DTMA complexes alters the local crystal field, resulting in dramatic spectroscopic changes, *Dalton Trans.*, 2015, **44**, 19509–19517.
7. (a) J. I. Bruce, R. S. Dickins, L. J. Govenlock, T. Gunnlaugsson, S. Lopinski, M. P. Lowe, D. Parker, R. D. Peacock, J. J. B. Perry, S. Aime and M. Botta, The selectivity of reversible oxy-anion binding in aqueous solution at a chiral europium and terbium center: signaling of carbonate chelation by changes in the form and circular polarization of luminescence emission, *J. Am. Chem. Soc.*, 2000, **122**, 9674–9684; (b) S. J. Butler and D. Parker, Anion binding in water at lanthanide centres: From structure and selectivity to signalling and sensing, *Chem. Soc. Rev.*, 2013, **42**, 1652–1666.
8. (a) P. Kuzmič, Program DYNAFIT for the analysis of enzyme kinetic data: application to HIV proteinase, *Anal. Biochem.*, 1996, **237**, 260–273; (b) P. Kuzmič, Chapter 10, DYNAFIT-A software package for enzymology, *Methods Enzymol.*, 2009, **467**, 247–280.
9. T. J. Sørensen, L. R. Hill and S. Faulkner, Thermodynamics of Self-Assembly of Dicarboxylate Ions with Binuclear Lanthanide Complexes, *ChemistryOpen*, 2015, **4**, 509–515.

10. H. Putz and K. Brandenburg GbR, *Diamond – Crystal and Molecular Structure Visualization*, version 4.6.6, Crystal Impact, Bonn, Germany, 2021.
11. A. Dadabhoy, S. Faulkner and P. G. Sammes, Long wavelength sensitizers for europium(III) luminescence based on acridone derivatives, *J. Chem. Soc., Perkin Trans. 2*, 2002, 348–357.
12. J. D. Moore, R. L. Lord, G. A. Cisneros and M. J. Allen, Concentration-independent pH detection with a luminescent dimetallic Eu(III)-based probe, *J. Am. Chem. Soc*., 2012, **134**, 17372–17375.
13. S. Aime, M. Botta, S. G. Crich, G. B. Giovenzana, R. Pagliarin, M. Piccinini, M. Sisti and E. Terreno, Towards MRI contrast agents of improved efficacy. NMR relaxometric investigations of the binding interaction to HSA of a novel heptadentate macrocyclic triphosphonate Gd(III) complex, *J. Biol. Inorg. Chem*., 1997, **2**, 470–479.
14. A. K. R. Junker, M. Tropiano, S. Faulkner and T. J. Sørensen, Kinetically inert lanthanide complexes as reporter groups for binding of potassium by 18-crown-6, *Inorg. Chem*., 2016, **55**, 12299–12308.
15. (a) Analytical methods committee, Recommendations for the definition, estimation and use of the detection limit, *Analyst*, 1987, **112**, 199–204; (b) J. Mocak, A. M. Bond, S. Mitchell and G. Scollary, A statistical overview of standard (IUPAC and ACS) and new procedures for determining the limits of detection and quantification: application to voltammetric and stripping techniques, *Pure & Appl. Chem*., 1997, **69**, 297–328.
16. (a) I. Ting-Po and G. H, Nancollas, EQUIL: General computational method for the calculation of solution equilibriums, *Anal. Chem*., 1972, **44**, 1940–1950; (b) T. B. Gasa, J. M. Spruell, W. R. Dichtel, T. J. Sørensen, D. Philip, J. F. Stoddart and P. Kuzmič, Complexation between Methyl Viologen (Paraquat) Bis(Hexafluorophosphate) and Dibenzo[24]Crown-8 Revisited, *Chem. Eur. J*., 2009, **15**, 106–116; (c) P. R. Nawrocki, N. Kofod, M. Juelsholt, K. M. Ø Jensen and T. J. Sørensen, The effect of weighted averages when determining the speciation and structure–property relationships of europium(III) dipicolinate complexes, *Phys. Chem. Chem. Phys*., 2020, **22**, 12794–12805.
